# A Decentralized Receiver in Gaussian Interference

**DOI:** 10.3390/e20040269

**Published:** 2018-04-11

**Authors:** Christian D. Chapman, Hans Mittelmann, Adam R. Margetts, Daniel W. Bliss

**Affiliations:** 1 School of Electrical, Computer and Energy Engineering, Arizona State University, Tempe, AZ 85281, USA; 2 School of Mathematical and Statistical Sciences, Arizona State University, Tempe, AZ 85281, USA; 3 MIT Lincoln Laboratory, Lexington, MA 02421, USA

**Keywords:** distributed reception, communications networks, channel capacity, relay channels, interference mitigation

## Abstract

Bounds are developed on the maximum communications rate between a transmitter and a fusion node aided by a cluster of distributed receivers with limited resources for cooperation, all in the presence of an additive Gaussian interferer. The receivers cannot communicate with one another and can only convey processed versions of their observations to the fusion center through a Local Array Network (LAN) with limited total throughput. The effectiveness of each bound’s approach for mitigating a strong interferer is assessed over a wide range of channels. It is seen that, if resources are shared effectively, even a simple quantize-and-forward strategy can mitigate an interferer 20 dB stronger than the signal in a diverse range of spatially Ricean channels. Monte-Carlo experiments for the bounds reveal that, while achievable rates are stable when varying the receiver’s observed scattered-path to line-of-sight signal power, the receivers must adapt how they share resources in response to this change. The bounds analyzed are proven to be achievable and are seen to be tight with capacity when LAN resources are either ample or limited.

## 1. Introduction

**Figure 1:**
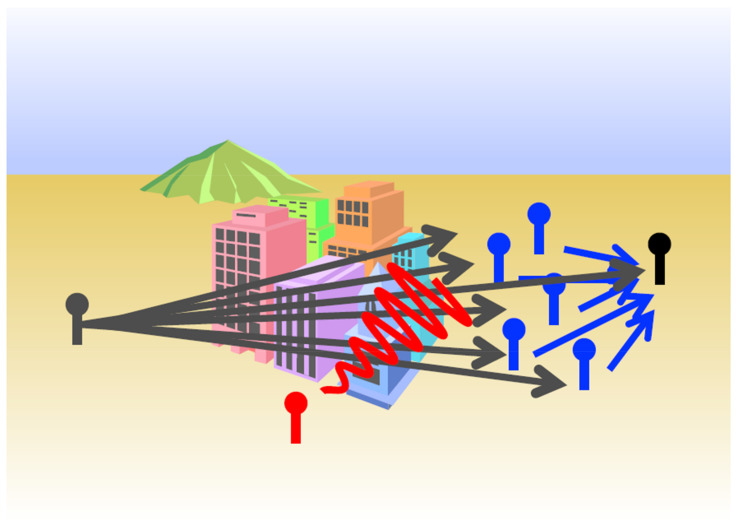
A broadcast node speaks to many helper nodes, which forward limited amounts of information to a base node. Additive interference from neighboring systems is present in the link from broadcaster to helpers.

We are at a remarkable time for radio systems. With the significant reductions in cost and improvements in performance, there has been an explosion in the number of radio systems with a wide range of applications including personal, machine-to-machine, vehicle-to-vehicle, and Internet-of-Things (IoT) communications. In many situations, radios cluster in physically close groups. This has two effects. First, it increases the likelihood of interference between systems and necessitates that systems be designed to maintain good performance in these conditions. Second, it creates an opportunity for groups of independent radios to be used as a distributed array to improve performance. Considering this new setting, we study the problem of receiving a signal in the presence of interference with the help of a distributed array, as illustrated in Figure 1.

We develop bounds on the maximum communications rate from a single transmitter to a base node that is provided side information from a distributed receive array in the presence of interference. The nodes that provide side information to the base are identified as *helpers*, and we assert they do not communicate with one another, but only forward information to the base through a previously established reliable Local Array Network (LAN) link, and that this link only supports a maximum total throughput which all the helpers must share.

This receive system can mitigate a strong interferer in a wide variety of environments. We consider the effects of an overall LAN capability that is parameterized by a total number of bits that helpers can share with the base for each source channel usage. Furthermore, to investigate algorithmic needs as a function of environmental conditions, we consider a spatially Ricean channel and a parametrically defined interference. Finally, we consider both the conditions in which the base node does and does not have its own noisy observation of the source signal. Our bound development in this paper provides significant extensions to and clarifications of the results found in References [1,2], and our preliminary efforts in Reference [3].

### 1.1. Results

We perform an analysis of the achievable rates of the system in the presence of an interferer. The following contributions are provided:

- Upper and lower bounds on the system’s achievable communication rate in correlated Gaussian noise and regimes where these bounds are tight (Section 3). The strongest lower bound is given in Theorem 7.
- Performance characteristics of these rates in the presence of an interferer (Section 4.1).
- Behavior of the strategies in various scattering environments (Section 4.2). The scattering environment is seen to not affect average performance if LAN resource sharing adapts to the channel.
- A strengthening of an existing achievability proof for the system, where the same rate is achieved using less cooperation between nodes (Remark 7).

### 1.2. Background

The problem of communicating with the help of an array of distributed receivers has been studied in a variety of contexts, most generally as an instance of communications over a single-input multiple-output (SIMO) channel. A variety of SIMO channels have been extensively analyzed, but most analyses are subtly different than the problem considered here. Our work is done in the context of information theory and bounds achievable communications rates, while in contrast most existing studies work towards minimizing distortion metrics or bit error rates.

Results presented here are a significant extension and generalization of the work in [3], where there is no treatment of the system’s performance in the presence of an interferer. A tighter inner bound than all those in [3] is included in Theorem 7, and full proofs are provided for all the results.

Achievable communications rates for this network topology were studied in References [1,2], although their bounds do not directly apply to channels with a Gaussian interferer. The studies provide an example demonstrating the sub-optimality of a Gaussian broadcaster when no interference is present, and suggest that non-Gaussian signaling techniques are needed for this sort of network. In contrast, we demonstrate in Section 4 that an achievable rate using Gaussian signaling typically comes quite close to the system’s upper bound in many practical regimes.

The helpers in the system we have described seem like relay nodes but there is an important distinction. Here, the ensemble of links from each helper to the base are like those in a graphical network, available for design within distributional and rate constraints. This is not usual in the context of relay networks, including those studied in very general setting such as Reference [4]. Studies such as References [5,6,7] detailed using a collection of receivers as beam forming relays to a destination node in a network with structure similar to the system considered here. In our situation, each helper-to-base link is orthogonal to the others, so beam forming studies are not directly applicable.

Performance of specific coding schemes for this system were studied in References [8,9,10]. In particular, [10] was able to identify a scheme that can perform to within 1.5 dB of allowing the base and receivers to communicate without constraints. Results in our study further work towards characterizing achievable rates of the system rather than designing and analyzing the performance of specific modulation schemes.

The topology we consider is similar to many-help-one problems such as the “Chief Executive Officer (CEO) problem” posed by Berger in Reference [11]. In this scenario, some node called the CEO seeks to estimate a source by listening to “agent” nodes which communicate to the CEO at fixed total rates. Many variations of this problem have been studied, for instance in Reference [12] in limit with number of agents. The focus of the CEO problem and most of its derivatives are to estimate a source to within some distortion (often mean squared error), whereas the focus here is on finding achievable rates for lossless communications.

The noisy Slepian–Wolf problem [13] may be interpreted as communications in the opposite direction of our system: distributed, cooperative helpers have a message for a base, but their cooperation ability is limited and their modulations must be sent over a noisy channel.

## 2. Problem Setup

Throughout the paper, we use the notation in Table 1. A broadcaster seeks to convey a message to a base at some rate R>0 bits per time period. The message *M* is uniformly distributed along $\left[1:2^{TR}\right]$ over some T∈N time periods. Our goal is to determine the greatest rate *R* at which the base can recover *M* with low probability of error.

Before communicating, the broadcaster and base agree on a modulation scheme $\phi :\left[1:2^{TR}\right]\to C^{T}$, where each possible message $m\in [1:2^{TR}]$ is mapped to a *T*-length signal through ϕ. Each of these signals is denoted $x_{m}=\left(x_{m,1},\ldots ,x_{m,T}\right)=\phi \left(m\right)$, and it is chosen within the broadcaster’s power constraint: $\frac{1}{T}\sum_{\ell =1}^{T}{\left|x_{m,\ell }\right|}^{2}\leq 1$. To send message *M*, the broadcaster transmits $x_{M}$, which we denote $X=\left(X_{1},\ldots ,X_{T}\right)=x_{M}$.

The signal is observed by N+1 single-antenna receive nodes through a static flat fading channel and additive white Gaussian noise. Enumerating these receivers 0–*N*, we identify the base as the “0th” receiver, and call the rest *helpers*. For n∈[0:N], at each time *t*, the nth receiver observes

(1) $$Y_{n,t}=h_{n}X_{t}+W_{n,t},$$

where

- the channel $h≜\left[\begin{array}{c} h_{0}\\ \vdots \\ h_{N} \end{array}\right]\in C^{(N+1)\times 1}$ is constant over time *t*, and
- the noise $\left(W_{0,t},\ldots ,W_{N,t}\right)∼\mathrm{CN}\;\left(0,\Sigma \right)$ has covariance Σ across receivers, and is independent over *t*.

The receiver observations are written as vectors:

(2) $$\begin{array}{cc} Y_{n} & ≜\left(Y_{n,1},\ldots ,Y_{n,T}\right)\in C^{T}, \end{array}$$

(3) $$\begin{array}{cc} \;Y & ≜\left(Y_{0},Y_{1},\ldots ,Y_{N}\right)\in {\left(C^{T}\right)}^{N+1}. \end{array}$$

Helper *n* for n∈[1:N] employs a (possibly randomly chosen) vector quantizer $Q_{n}$ on its received sequence $Y_{n}$ to produce a coarse $r_{n}$-bitrate summary of its observations, $U_{n}≜Q_{n}\left(Y_{n}\right)$. The vector of helper messages is denoted:

(4) $$\begin{array}{cc} U & ≜(U_{1},\ldots ,U_{N}) \end{array}$$

(5) $$\begin{array}{c} \;=(Q_{1}\left(Y_{1}\right),\ldots ,Q_{N}\left(Y_{N}\right)) \end{array}$$

and the quantizers $(Q_{1},\ldots ,Q_{N})=Q$ satisfy two properties:

- Every node in the system is informed of each quantizer’s behavior, and quantizers produce their compression using only local information. Formally, the probability distribution of (Q,X,Y,U) factors:
  (6) $$\begin{array}{c} P_{\left(Q,X,Y,U\right)}=P_{Q}\cdot P_{X|Q}\cdot P_{Y|X}\cdot \prod_{n=1}^{N}P_{U_{n}|Y_{n},Q}. \end{array}$$
- Quantizations are within the LAN constraint:
  (7) $$\frac{1}{T}H\left(U_{n}|Q\right)\leq r_{n},\;n\in \left[1:N\right].$$

The vector of the helper’s quantization rates is denoted:

(8) $$r≜\left(r_{1},\ldots ,r_{N}\right)\in C^{(N+1)\times 1}.$$

Each $U_{n}$ is conveyed precisely to the base over a Local Array Network (LAN). We assume the LAN only supports a limited amount of communication between helpers and the base. For instance, the helpers must all share a small frequency band. We model this by asserting that feasible rate vectors r belong to a bounded set $R_{\mathrm{LAN}}\left(L\right)$ with a sum-capacity L>0:

(9) $$R_{\mathrm{LAN}}\left(L\right)≜\left\{r\left|\sum_{n=1}^{N}r_{n}\leq L,\;r_{n}\geq 0\right.\right\}.$$

**Figure 2:**
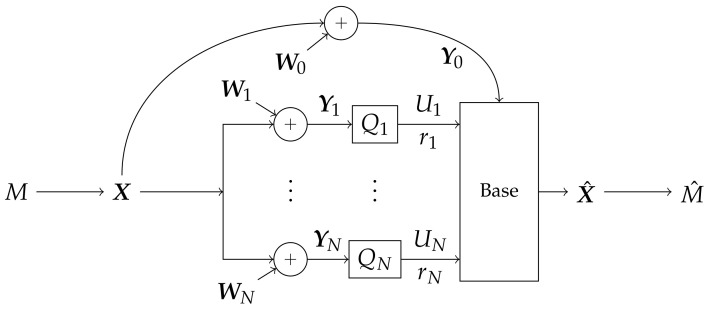
The system in consideration. A message *M* is broadcast as X with average power 1. The signal is received by a base and *N* helper nodes, occluded by correlated AWGN W. Helper *n* for n∈[1:N] quantizes $Y_{n}$ through $Q_{n}$ to produce an $r_{n}$-bit summary $U_{n}$. The quantized observations U and the base’s full-precision reception $Y_{0}$ are combined to produce an estimate $\hat{X}$ of X, then an estimate $\hat{M}$ of *M*.

We refer to the condition that $r\in R_{\mathrm{LAN}}\left(L\right)$ as the *LAN constraint.* The base reconstructs an estimate $\hat{X}$ of X using its own full-precision observation $Y_{0}$ and side information U from the helpers, then recovers an estimate $\hat{M}$ of message *M* from $\hat{X}$. A block diagram of the system is shown in Figure 2.

Channel h and noise covariance Σ are assumed to be static throughout the transmission of each message and are known to all the receive nodes. Both the transmitter and the receivers are assumed to have knowledge of the set of feasible rates, $R_{\mathrm{LAN}}\left(L\right)$. The environment determines:

- channel fades h,
- noise covariance Σ and
- maximum LAN throughput *L*.

Available for design are:

- quantizers Q,
- message modulation ${\left\{x_{m}\right\}}_{m\in [1:2^{TR}]}$,
- rates each helper should send to the base r and
- fusion and decoding methods at the base to produce $\hat{M}$.

We say a communication rate *R* is *achievable* if for any ε>0 then for large enough *T* there is some distribution of quantizers Q satisfying $\frac{1}{T}H\left(Q_{k}\left(Y_{k}\right)|Q\right)\leq r_{k}$ for each *k*, encoders $\phi_{Q}:\left[1:2^{TR}\right]\to X^{T}$ and decoders $f_{Q}$ where:

(10) $$P(f_{Q}\left(U\right)\neq M)<\varepsilon .$$

**Theorem** **1.** *The maximum communications rate achievable with arbitrarily low probability of error is:*

(11) $$C=\underset{T}{\sup}\max_{\begin{array}{c} \left(X,Y,Q,U\right)∼P\in S_{T}:\\ {E|X|}^{2}\leq 1 \end{array}}\frac{1}{T}I\left(X;Y_{0},U|Q\right)$$

*where $S_{T}$ is the collection of joint distributions with block length T that satisfy conditions in Equations (6) and (7) for some $r\in R_{\mathrm{LAN}}\left(L\right)$.*

**Proof.**  *C* is achievable through the usual noisy channel coding argument [14]. For any achievable rate *R*, applying Fano’s inequality to H(M|Q) gives R≤C. □

In the sequel, we bound *C* from Theorem 1 with computable expressions.

## 3. Bounds on Communications Rates

The system’s capacity *C* can be upper bounded by considering a stronger channel where all helpers are informed of each other’s observations. Compute:

(12) $$\begin{array}{cc} I(X;Y_{0},U|Q) & =I\left(X;U|Y_{0},Q\right)+I\left(X;Y_{0}|Q\right) \end{array}$$

(13) $$\begin{array}{c} \;\leq H\left(U|Q\right)+I\left(X;Y_{0}\right) \end{array}$$

(14) $$\begin{array}{c} \;\leq T\cdot \left(L+\log_{2}\left[1+∥h_{0}{∥}^{2}/\Sigma_{0,0}\right]\right), \end{array}$$

where the first inequality follows since $Q\to X\to Y_{0}$ is a Markov chain (Equation (6)). If the LAN constraint were not imposed and the base had access to the helpers’ receptions in full precision, the receive side would be equivalent to a multi-antenna Gaussian receiver. By noisy channel coding [15], the capacity of this stronger channel is $\max_{P_{X}{:E[|X|}^{2}]<1}I\left(X;Y\right)$ (here, (X,Y) are variables at a single timepoint) which, by a derivation given in [16], simplifies to the familiar upper bound:

(15) $$\log_{2}\;\left[\frac{|\Sigma +{\mathrm{hh}}^{†}|}{\left|\Sigma \right|}\right].$$

Taking the minimum of this bound and Equation (14) yields an upper bound for the original system. This is an instance of a general *cut-set upper bound* from [17].

**Remark** **1.** *Any achievable rate R must satisfy*

(16) $$\begin{array}{c} R\leq \min\;\left\{\log_{2}\;\left[\frac{|\Sigma +{\mathrm{hh}}^{†}|}{\left|\Sigma \right|}\right]\;,L+\log_{2}\left[1+∥h_{0}{∥}^{2}/\Sigma_{1,1}\right]\right\}\;. \end{array}$$

**Proof.**  Justified by the preceding discussion. □

The rest of the section is dedicated to four communication strategies for the system in order of increasing complexity and diversity utilization. The strongest bound is presented in Theorem 7. It is described using the context of the preceding two bounds.

### 3.1. Achievable Rate by Decoding and Forwarding

Treating each helper node as a user seeking to receive its own message, the link from the broadcaster to helpers is a scalar Gaussian broadcast channel. The capacity region of this channel was characterized in Reference [18], and in particular its sum-rate was given in Reference [19]. In the scalar case, this sum rate reduces to:

(17) $$\max_{n}\log_{2}\left[\frac{\Sigma_{n,n}+{\left|h_{n}\right|}^{2}}{\Sigma_{n,n}}\right],$$

where only the point-to-point channel between transmitter and the best receiver is used.

**Remark** **2.** *The following rate is achievable*

(18) $$\begin{array}{c} R_{\mathrm{BC}}\left(L\right)=\max\left\{\log_{2}\left[\frac{\Sigma_{0,0}+{\left|h_{0}\right|}^{2}}{\Sigma_{0,0}}\right],\min\left\{L,\max_{n}\log_{2}\left[\frac{\Sigma_{n,n}+{\left|h_{n}\right|}^{2}}{\Sigma_{n,n}}\right]\right\}\right\}. \end{array}$$

*The rate is achievable with the following facilities:*

- Helpers are informed of which helper is the best (best is derived from h,Σ,L).
- The transmitter and best helper coordinate a codebook of appropriate rate and distribution.

**Proof.**  By having the broadcaster send a message to the *n*th receiver, a rate

(19) $$\log_{2}\left[\frac{\Sigma_{n,n}+{\left|h_{n}\right|}^{2}}{\Sigma_{n,n}}\right]$$

is achievable from transmitter-to-receiver. If n=0 (i.e., the transmitter broadcasts to the base), then Equation (19) is achievable from transmitter-to-base. Otherwise, Equation (19) is achievable from transmitter-to-base if it is less than *L* by forwarding the receiver’s decoding over the LAN. □

**Remark** **3.** *When the base does not have its own reception (i.e., $I(X;Y_{0})=0$) and*

(20) $$L\leq \max_{n}\log_{2}\left[\frac{\Sigma_{n,n}+{\left|h_{n}\right|}^{2}}{\Sigma_{n,n}}\right]$$

*then the system’s capacity is L.*

**Proof.**  By assumption, the upper bound in Equation (16) equals L. This is achievable by Remark 2. □

The strategy to achieve this rate requires a strong amount of cooperation from the broadcaster, since the decoding receivers must all share codebooks with the transmitter.

### 3.2. Achievable Rate through Gaussian Distortion

Rate-distortion theory shows that to encode a Gaussian source $Y∼N(0,\sigma^{2})$ with minimum rate such that distortion does not exceed some maximum allowable mean squared error D>0, the rate must be at least

(21) $$R\left(D\right)=\log_{2}\left(\sigma^{2}\right)-\log_{2}\left(D\right).$$

To achieve this rate, the encoding operation must emulate the following test channel (Reference [14]):

(22) $$\underset{W∼\mathrm{CN}\;(0,1)}{Y\overset{\alpha /\beta }{\to }\underset{↑}{⊕}\overset{\beta }{\to }Z}=\alpha Y+\beta W$$

where $\alpha =\sqrt{1-D/\sigma^{2}}$ and $\beta =\sqrt{D}$. Dithered lattice quantization [20] is one method that can be used to realize such a quantizer in limit with block length in addition to having other nice analytic properties. Using this method, in limit with block length, the base’s estimates for a helper’s observation at any single timepoint can be made to approximate

(23) $$Z_{n}\left(r_{n}\right)≜Y_{n}+W_{Q,n}\left(r_{n}\right)$$

where $W_{Q,n}\left(r_{n}\right)$ is independent Gaussian distortion with variance some function of the helper’s rate $r_{n}$. We will now derive this function. Define

(24) $$W_{Q}\left(r\right)≜\left(W_{Q,1}\left(r_{1}\right),\ldots ,W_{Q,N}\left(r_{n}\right)\right)\in C^{N\times 1},$$

and the vector of $Z_{n}$s corresponding to this choice of $W_{Q,n}$ as:

(25) $$Z\left(r\right)≜Y+W_{Q}\left(r\right).$$

The subscript *Q* here is a decoration to distinguish quantization distortion terms $W_{Q},W_{Q,n}$ from environment noise, $W,W_{n}.$

If the *n*th helper is to forward information to the base at rate $r_{n},$ then the amount of distortion in the helper’s encoding under this strategy can be determined by setting Equation (21) equal to $r_{n}$ and solving for D, with $\sigma^{2}$ equal to the helper observation’s variance, $∥h_{n}{∥}^{2}+\Sigma_{n,n}$. Scaling the output of the test channel the helper emulates (Equation (22)) by 1/α causes it to equal the helper’s observation plus independent Gaussian noise with variance ${\left(\beta /\alpha \right)}^{2}=D/\left(1-D/\sigma^{2}\right)$. This means that, in such a system, the distortion from the helpers is equivalent to adding independent additive Gaussian noise with variance:

(26) $$\mathrm{Var}\left(W_{Q,n}\left(r_{n}\right)\right)=\frac{∥h_{n}{∥}^{2}+\Sigma_{n,n}}{2^{r_{n}}-1}.$$

By Equations (1) and (26), all the noise and distortion on the signal present in the helpers’ messages to the base are summarized by the vector $W+W_{Q}$ with covariance matrix

(27) $$D\left(r\right)≜\Sigma +\mathrm{diag}\left(0,\frac{∥h_{1}{∥}^{2}+\Sigma_{1,1}}{2^{r_{1}}-1},\ldots ,\frac{∥h_{N}{∥}^{2}+\Sigma_{N,N}}{2^{r_{N}}-1}\right).$$

**Figure 3:**
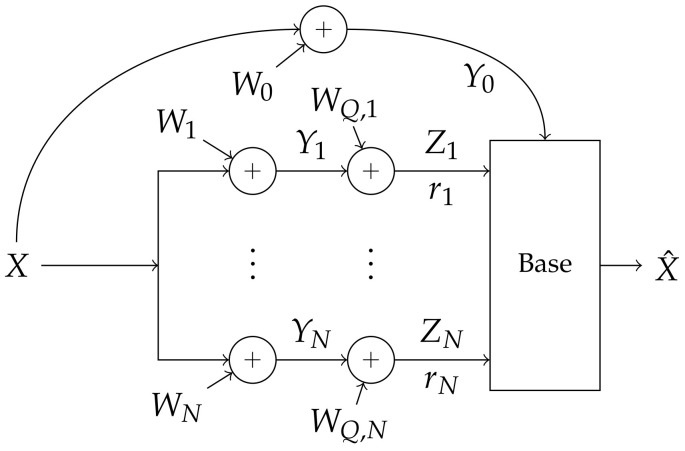
Gaussian distortion System: Quantizer distortion is modeled as additive white Gaussian noise, where enough noise is added so that the capacity across quantizers is some given rate vector r.

We refer to this system as a *Gaussian distortion system*. A block diagram of this system is shown in Figure 3. This system is equivalent to a multi-antenna Gaussian receiver and its achievable rate has a closed form:

(28) $$R_{G}\left(r\right)≜I\left(X;Z\left(r\right)\right)=\log_{2}\left[\frac{|D\left(r\right)+{\mathrm{hh}}^{†}|}{\left|D(r)\right|}\right].$$

**Theorem** **2.** *For a distributed receive system as described in Section 2 with noise covariance matrix* Σ, *LAN constraint L and fixed average helper quantization rates $r\in R_{\mathrm{LAN}}\left(L\right)$, then a rate $R_{G}\left(r\right)$ is achievable with the following facilities:*

- The transmitter and base coordinate a codebook of appropriate rate and distribution.
- Each n-th helper is informed of an encoding rate $r_{n}$ (optimal choice derived from h,Σ,L).
- Each helper coordinates a random dither with the base.
- The base is informed of r,h,Σ,L.

All the details of this derivation are formalized in Appendix B.

Maximizing $R_{G}$ over $R_{LAN}\left(L\right)$ gives a lower bound on the system capacity:

(29) $$R_{G,\max}\left(L\right)≜\max_{r\in R_{\mathrm{LAN}}\left(L\right)}R_{G}\left(r\right).$$

Unfortunately, this expression cannot be simplified much in general. Maximization of $R_{G}$ over $R_{\mathrm{LAN}}\left(\cdot \right)$ can be performed efficiently using quasi-convex optimization algorithms:

**Remark** **4.** The max of $R_{G}\left(r\right)$ follows the maximum of $h^{†}D{\left(r\right)}^{-1}h,$ which is quasi-concave in r.

**Proof.**  The maximum of $R_{G}$ follows the maximum of $-h^{†}D{\left(r\right)}^{-1}h$ by the matrix determinant lemma.

It suffices to show that the restriction of this functional on the intersection of any line with $R_{+}^{N\times 1}$ is quasi-concave (see Reference [21]). Further, a one-dimensional function is quasi-concave if anywhere its derivative is 0 and its second derivative is below 0.

Fix any $a,b\in R^{N\times 1},$ denote $D_{t}{≜D\left(\cdot \right)|}_{ta+b}$ for any *t* where $ta+b\in R_{+}^{N\times 1}$. Define $A_{t}≜\frac{d}{dt}D_{t},$ and $f\left(t\right)≜-h^{†}D_{t}^{-1}h$. Then,

(30) $$\frac{d}{dt}f\left(t\right)=A_{t}D_{t}^{-1}h$$

(31) $$\frac{d^{2}}{dt^{2}}f\left(t\right)={\left(D_{t}^{-1}h\right)}^{†}\left[\frac{d}{dt}A_{t}-2A_{t}D_{t}^{-1}A_{t}\right]\left(D_{t}^{-1}h\right).$$

If $\frac{d}{dt}f\left(t\right)=0,$ then $D_{t}^{-1}h$ is in the null space of $A_{t}$ so the $-2A_{t}D_{t}^{-1}A$ term in Equation (31) vanishes. $\frac{d}{dt}A_{t}$ is negative definite in $R_{+}^{N\times 1}$ so $\frac{d^{2}}{dt^{2}}f\left(t\right)<0$ there, establishing *f*’s quasi-concavity and thus that of $-h^{†}D_{t}^{-1}h^{†}$. Since $-h^{†}D_{t}^{-1}h^{†}$ is quasi-concave along all of $R_{+}^{N\times 1}$, then it is also quasi-concave in any convex restriction of that domain. $R_{LAN}\left(L\right)$ is a simplex, which is convex. □

$R_{G,\max}\left(L\right)$ is tight with the upper bound in Equation (16), in limit with LAN throughput *L*.

**Remark** **5.** $R_{G,\max}\left(L\right)\to C$ as L→∞.

**Proof.**  $R_{G\max}\left(L\right)\geq R_{G}\left(L/N\cdot 1_{N}\right),$ and if L→∞ then

(32) $$R_{G}\left(\;L/N\cdot 1_{N}\;\right)\to \log_{2}\left[\frac{|\Sigma +{\mathrm{hh}}^{†}|}{\left|\Sigma \right|}\right]$$

by continuity of $\log_{2}$, sums, and the matrix determinant (in terms of matrix coordinates). The limit in Equation (32) is greater than or equal to Equation (16), establishing the remark. □

This strategy has the benefit of not needing much coordination between receive nodes. The transmitter does not have to share codes with helpers because here the helpers do not perform any decoding. Each helper needs only its encoding rate and its own channel state to form its messages properly.

### 3.3. Achievable Rate through Distributed Compression

Helper *n* for n∈[1:N] in a realization of the Gaussian distortion system described in Section 3.2, on average and with long codes, will produce an $r_{n}$-bit encoding of its observation that can be decoded to produce the helper observation with additional approximately Gaussian noise. Since all the helper’s quantizations contain the same signal component (and possibly the same interference), they are correlated and can be compressed before forwarding to allow for less noise to be introduced in quantization.

The Slepian–Wolf theorem [22] shows that if the LAN is such that helpers can encode at rates $\rho ≜(\rho_{1},\ldots ,\rho_{N})$, then the helpers can losslessly convey the encodings to the base at lower rates r⪯ρ, as long as r and the encodings U satisfy the conditions that for all subsets S⊆[1:N], then:

(33) $$H\left(U_{S}|U_{S^{C}},Y_{0},Q\right)<\sum_{n\in S}r_{n}.$$

Note that $Y_{0}$ is always included in the conditioning because $Y_{0}$ is available at the base node in full precision.

In the Gaussian distortion setting described right before Equation (23), $H(U_{S}|U_{S^{C}},Y_{0},Q)$ can be made close to $I(Y_{S};Z_{S}\left(\rho \right)|Z_{S^{C}}\left(\rho \right),Y_{0})$ with time expansion. The details of this are given in Lemma 2. Maximizing over the LAN constraint in Equation (9), the following rate is achievable:

(34) $$\begin{array}{c} R_{DC}\left(L\right)≜\max_{r\in R_{\mathrm{LAN}}\left(L\right)}\;\max_{\rho \in R_{DC}\left(r\right)}R_{G}\left(\rho \right) \end{array}$$

where

(35) $$\begin{array}{c} R_{DC}\left(r\right)=\left\{\rho :\forall S\subseteq [1:N],I\left(Y_{S};Z_{S}\left(\rho \right)|Z_{S^{C}}\left(\rho \right),Y_{0}\right)<\sum_{n\in S}r_{n}\right\}. \end{array}$$

Z(·) in Equation (35) is defined as in Equation (25). We can then state the following:

**Theorem** **3.** *For a distributed receive system as described in Section 2 with noise covariance matrix* Σ *and LAN constraint L, $R_{DC}\left(L\right)$ is achievable with the following facilities:*

- The transmitter and base coordinate a codebook of appropriate rate and distribution.
- Each n-th helper is informed of a quantization rate $\rho_{n}$ and binning rate $r_{n}$, (optimal choice derived from h,Σ,L).
- Each helper coordinates a random dither with the base.
- The base is informed of ρ,r,h,Σ,L.

The base can unambiguously decompress the helper’s compressed encodings with low probability of error if and only if ρ is chosen such that Equation (33) is satisfied. However, in analog to Corollary 1 from Reference [1], the rate in 6 can be improved by expanding $R_{DC}\left(r\right)$ to include some of the ρ where the base cannot perform unambiguous decompression. This helps because even if the encoding rates in ρ are chosen outside of $R_{DC}\left(r\right)$ so that helpers cannot convey U to the base unambiguously, some extra correlation with *X* is retained through this distortion.

**Theorem** **4.** *For a distributed receive system as described in Section 2 with noise covariance matrix* Σ *and LAN constraint L, the following rate is achievable:*

(36) $$R_{\bar{DC}}\left(L\right)≜\max_{\begin{array}{c} r\in R_{\mathrm{LAN}}\left(L\right),\\ \lambda >0 \end{array}}\;\max_{\rho \in R_{DC}^{\lambda }\left(r\right)}R_{G}\left(\rho \right)-\lambda$$

*where*

(37) $$\begin{array}{c} R_{DC}^{\lambda }\left(r\right)≜\left\{\rho :\forall S\subseteq [1:N],I\left(Y_{S};Z_{S}\left(\rho \right)|Z_{S^{C}}\left(\rho \right),Y_{0}\right)<\sum_{n\in S}r_{n}+\lambda \right\}. \end{array}$$

*The rate requires the following facilities:*

- The transmitter and base coordinate a codebook of appropriate rate and distribution.
- Each n-th helper is informed of a quantization rate $\rho_{n}$, binning rate $r_{n}$ and hull parameter λ (optimal choice derived from h,Σ,L).
- Each helper coordinates a random dither with the base.
- The base is informed of ρ,r,λ,h,Σ,L.

All the details of Theorems 6 and 7 are shown in Appendix B.

**Remark** **6.** *The capacity of the system is $R_{\bar{DC}}\left(L\right)$ under the following restrictions:*

- Σ *is diagonal (no interference).*
- The base does not have its own full-precision observation of the broadcast ($h_{0}=0$).
- The broadcaster must transmit a Gaussian signal.
- Construction of helper messages is independent of the transmitter’s codebook X.

This is demonstrated in Appendix C. The last three assumptions are necessary:

- Since the base has codebook knowledge, it is possible for the transmitter to send a direct message to the base, which is not accounted for in the compress-and-forward strategy used for $R_{\bar{DC}}\left(L\right)$.
- The Gaussian broadcast assumption is needed because of a counterexample given in Reference [1].
- Codebook independence is necessary because $R_{\bar{DC}}\left(L\right)$ is strictly less than the upper bound in Equation (16), but, by Remark 3, this upper bound is achieved in some regimes.

Theorem 7 and Remark 7 strengthen Corollary 1 and Theorem 5 from Reference [1] since we show that the same rate can be achieved with less cooperation between transmitter and receivers. Sanderovich et al. [1] used a “nomadicity” assumption which asserts that the mapping from transmitter messages to codewords is not present at the helpers, whereas Theorem 7 shows (the same argument applying to the general discrete case) that indeed the same rate is achievable when helpers have *no knowledge at all* of the codewords the transmitter is using.

Similar to the Gaussian distortion achievable rate, the distributed compression technique does not require cooperation between the transmitter and helpers. It does, however, require a priori sharing of codes from the helpers to the base so that the helpers can perform distributed compression.

## 4. Ergodic Bounds

In this section, the bounds are averaged over random channels in various regimes. Each bound tested is a deterministic function of:

- number of helpers *N*,
- LAN constraint *L*,
- noise covariance matrix Σ, and
- channel h (assumed to be static and precisely estimated a priori per each channel use).

In all graphs, the rate from Equation (16) is called “Upper Bound”, the rate from Equation (28) is “Gaussian Distortion”, the rate from Theorem 7 is “Distributed Compression” and the rate from Remark 2 is “Broadcast”. A discussion of the optimizations performed for the Gaussian Distortion and Distributed Compression bounds is in Appendix A.

### 4.1. Performance in the Presence of an Interferer

One motivation for increasing receive diversity is to better mitigate the effect of interference, so it is important to study the extent to which the strategies from Section 3 can do so.

We model interference as a zero-mean Gaussian broadcast independent of our system. Our system’s nodes are informed of nothing more than the scales at which the interferer appears in our receivers’ observations. This assumption prohibits using dirty paper coding [23] and other transmit-side interference mitigation strategies. With this model, the covariance matrix of noise terms associated with a particular interferer seen by the receivers is a matrix $A=aa^{†},$ where $a\in C^{N+1}.$ In all trials run, a single interferer was assumed to be present at all nodes with power α and random phase so that

(38) $$\Sigma =I_{N+1\times N+1}+\alpha \cdot aa^{†}.$$

with $a_{n}=e^{j2\pi \cdot \theta_{n}},\;\theta_{n}∼\mathrm{Unif}\left[0,1\right]$ where Unif is the uniform distribution.

**Figure 4:**
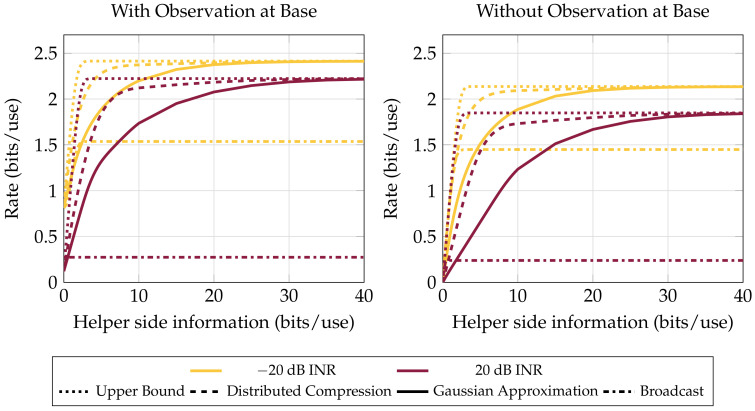
Bound performance versus LAN constraint (that is, the total rate available to hub from all the helpers combined). Four helpers with 0 dB average receiver SNR, averaged over 1000 channels with gains ∼CN (0,I). Interferer present at each receiver with a uniform random phase and specified INR. Single-user decode-and-forward is greatly affected by interference (Compare the Broadcast curves from −20 dB to 20 dB INR). Distributed compression only offers significant benefits over Gaussian compress-and-forward in strong interference and when LAN resources are scarce.

Figure 4 shows the difference in achievable rates with and without interference for different receive-side strategies. Even when a strong rank-one interferer is present at all the nodes, achievable communications rates are comparable to the case without an interferer.

Sanderovich et al. [1] provided an example of a system such as the one in the present work where a Gaussian transmitter is significantly sub-optimal. In contrast, we see in Figure 4 that, over an average of many random channels in a range of settings, achievable rates using Gaussian signaling come quite close to an upper bound on the system. Even the relatively simple Gaussian distortion bound is close in performance to the optimum in all regimes but one with strong interference and little cumulative helper-to-base information. In this regime the LAN constraint limits the helper’s ability to provide the base with the diversity of observations necessary to perform good interference mitigation.

### 4.2. Path Diversity versus Performance

It is reasonable to expect that the best strategy for a distributed receiver to use will change depending on its environment. If the receivers observe the signal through a single line-of-sight path, all else being equal, the signal and noise at each receiver will have similar statistics. In contrast, in environments with many scatterers, the channel statistics will vary more across spatially distributed receivers.

The level of attenuation at a receiver can be modeled with a Rician distribution [24]. The Rician distribution follows the magnitude of a circularly-symmetric complex Gaussian with nonzero mean and can be parameterized by two nonzero values: a scale Ω>0 representing the average receive SNR and a shape K>0 (called a *K-factor* in other literature) denoting the ratio of signal power received from direct paths to the amount of power received from scattered paths. By construction, if K=0, then a Rician distribution is equivalent to a Rayleigh distribution with mean Ω. In contrast, if K→∞, then the distribution approaches a point mass at Ω (Reference [25]).

**Figure 5:**
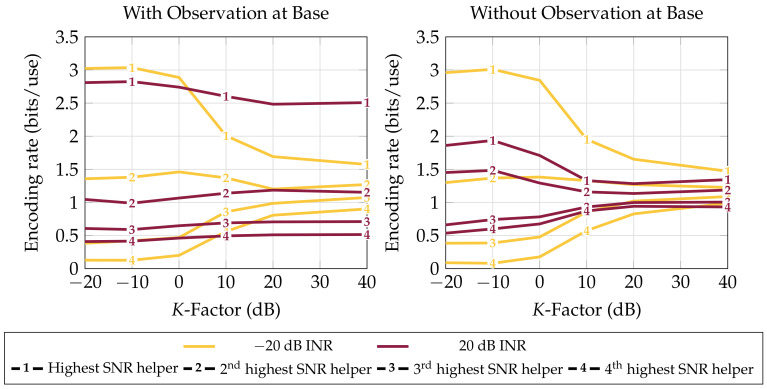
Average rate at which each helper forwards to base in distributed compression (Theorem 7) versus *K*-factor (scattered-to-direct-path received signal power ratio). Four helpers with 0 dB average receive SNR and a LAN constraint of L=5 bits per channel use of side information the helpers share to inform the base of their observations. Averaged over 1000 channels with gains ∼CN (0,I). Interferer present at each receiver with a uniform random phase and specified INR. As the proportion of line-of-sight path power increases, the need for receive diversity increases and the base draws information from more helpers. This does not occur when the base has its own observation, when presumably it is used as an estimate of the interferer to be mitigated from a compression of the highest-SNR helper’s observation.

**Figure A1:**
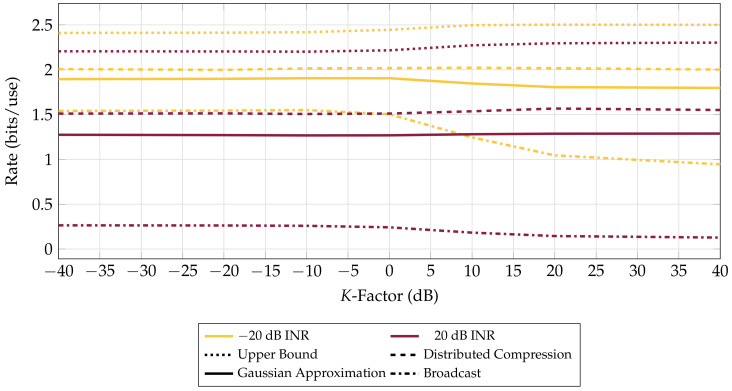
Achievable rates versus *K*. Four helpers with an observation available at the base and LAN constraint L=5 bits shared among helpers. Averaged over 500 channel realizations with uniform independent phase and average receiver SNR fixed at 0 dB. Scattering environment has negligible impact on achievable rates for these bounds, except in the Broadcast bound where receive diversity is not well utilized. Similar results are obtained when the base does not have its own observation.

The type of scattering environment does not greatly affect the average rate achievable by any bound other than the Broadcast bound (Figure A1). This is because the Broadcast bound’s only utilization of receive diversity is in channel variances which is small when the channel is dominated by line-of-sight receptions. Despite the invariance of most bounds to *K*, the profile of helper-to-base bits does change. Figure 5 shows that, no matter the other parameters, in high scattering, it is most helpful for the base to draw most of its information from the highest SNR helper while mostly ignoring low-SNR helpers’ observations. The imbalance is less pronounced in interference and when SNR is less varied across receivers (high *K*), since in these regimes, gaining diverse observations for combining is more beneficial than using the strongest helper’s observation.

When the base collects its own full-precision observation, the helper rate profile is unaffected by interference and scattering, where most helper information is provided by the highest SNR helper. In practice, in this situation, the base might use its observation as an estimate of the interferer and subtract it from a compression of the strongest helper’s observation.

## 5. Conclusions

One upper bound and three lower bounds on communication rate were developed. A simple upper bound (Remark 1) was derived, achievable when the LAN constraint is stringent (small *L*), and achievable in limit as the LAN constraint is relaxed (L→∞). An achievable rate (Theorem 5) considering quantizers which add Gaussian distortion was seen to provide large gains in interference over a rate that does not fully use the distributed receive array (Remark 2). The rate (Theorem 7) achieved when a distributed compression stage is added to the previous technique provides gains over Theorem 5 when interference is strong and helper-to-base communication is limited, but may be more difficult to implement.

Leaving SNR fixed, performance is mostly unchanged in high-scattering versus low-scattering environments, although the profile of helper-to-base communication changes: In high-scattering environments, some helper observations are ignored by the base, while, in line-of-sight environments, each helper informs the base equally. The presence of a strong interferer dampens this effect, since in this regime the base needs more spatial diversity to mitigate the interferer.

An immediate goal for future work is to devise practical implementations of the presented strategies. This may include refining the models of the LAN and helpers to more precisely reflect system capabilities.

## Figures and Tables

**Table 1:** Notation and terminology.

CN (0,Σ)	Complex circularly symmetric normal distribution with zero mean and Hermitian covariance matrix Σ
I(·;·),H(·),h(·)	Mutual information, Shannon entropy and differential entropy
$A^{†},a^{†}$	Conjugate transpose of matrix A, vector a
$a_{S};S\subseteq T$	Vector with components indexed by *S*
|A|	Determinant of a matrix A
[a:b]	Integers from *a* to *b*, inclusive
a⪰0	Vector a with non-negative elements
A⪰0	Positive semidefinite matrix A
$1_{N}$	*N*-row vector of 1 s
$A_{j,k}$	Element on row *j*, column *k* of a matrix A
diag(a)	Diagonal matrix where the (i,i)th diagonal element is the ith element of vector a
$I_{n\times n}$	n×n identity matrix
∥x∥	Vector magnitude/2-norm
$R_{+}$	Nonnegative real numbers

## Supplementary Materials

The following are available online at http://www.mdpi.com/1099-4300/20/4/269/s1.

## Appendix A. Optimization Method

Evaluation of the Gaussian distortion and distributed compression bounds in each environment requires optimization of a quasi-convex objective (Remark 4). In the case of distributed compression, the space of acceptable parameters is not convex: consider compressing the encodings for two highly-correlated sources. By the Slepian–Wolf theorem, if the encodings are high-bitrate, then perturbing the encodings’ bitrate does not greatly affect the region of feasible compression rates, since the encodings have high redundancy. On the other hand, perturbing the bitrate of low-bitrate encodings greatly affects the region of feasible compression rates, since uncorrelated distortion from encoding dominates source redundancies. Then, a compression to an average between minimum feasible compression rates for low-bitrate encodings and ones for high-bitrate encodings is infeasible, and the distance from the feasible set becomes large as the difference between high and low bitrates is made large. This is the mechanism that governs $R_{\bar{DC}}\left(r\right)\subseteq R_{+}^{K},$ hence the domain’s non-convexity.

To overcome this, an iterative interior point method was used to find the maximum: each constraint f(x)<0 is replaced with a stricter constraint, f(x)+β<0 for some β>0 and a minimization of the objective is performed under the new constraint. The optimal point is passed as the initial guess for the next iteration, where the objective function is minimized with updated constraints $f\left(x\right)+\beta^{\prime }<0$ with $0<\beta^{\prime }<\beta$. The method is iterated until the constraint is practically equivalent to f(x)<0 or the optimal point converged. Each individual minimization was performed using sequential least-squares programming (SLSQP) through SciPy [26]. The code for this is included in the Supplementary Materials.

## Appendix B. Achievability of Bounds

Several lemmas are needed to prove bound achievability. The following lemma demonstrates that lattice encodings become close in joint information content to the equivalent random variables in a Gaussian distortion system from Section 3.2. Lemma 1 demonstrates the joint differential entropies between source estimates from the encodings are close to the equivalent differential entropies in the Gaussian distortion system. Lemma 2 demonstrates the encodings themselves have joint Shannon entropies that are close to mutual informations in the Gaussian distortion system.

**Lemma** **1.** *Take helper observations Y and independent dithers ${\left(W_{Q,n}\right)}_{n\in [0:N]}$ where, for each n, the dither vector $W_{Q,n}$ is distributed along the base cell of a regular lattice in $C^{t}$ whose normalized second moment is $\sigma_{Q,n}^{2}$. For any A⊂[0:N], ε>0 and t large enough,*

(A1) $$\left|\frac{1}{t}h\left(Y_{A}-{\left(W_{Q,n}\right)}_{n\in A}\right)-h\left(Y_{A}+W_{A}\right)\right|<\varepsilon$$

**Proof.** The proof is similar to Theorem 6 from [20]. Notice that, for any additive noise channel, x→x+n then I(x;x+n)=h(x+n)−h(n) so that h(x+n)=h(n)+I(x;x+n). Then,

(A2)1th(YA−(WQ,n)n∈A)=1t[h((WQ,n)n∈A)+I(YA;YA−(WQ,n)n∈A)](A3)=1t[h((WQ,n)n∈A)−th(WA)+th(WA)+I(YA;YA−(WQ,n)n∈A)](A4)=1t[∑n∈Ah(WQ,n)−th(Wn)+th(WA)+I(YA;YA−(WQ,n)n∈A)](A5)≥1t[∑n∈Ah(WQ,n)−th(Wn)+th(WA)+tI(YA;YA+WA)](A6)=1t[∑n∈Ah(WQ,n)−th(Wn)+th(YA+WA)](A7)=h(YA+WA)+1t∑n∈AD(PWQ,n∥∏ℓ=1tPWn)→t↑∞h(YA+WA).

where Equation (A4) follows since the terms are independent, Equation (A5) follows by the worst additive noise lemma [27], and the substitution and convergence in Equation (A7) follow by Section III of [20]. In addition:

(A8)1th(YA−(WQ,n)n∈A)=1t[h((WQ,n)n∈A)+I(YA;YA−(WQ,n)n∈A)](A9)≤1th((WQ,n)n∈A)+I(YA;YA+WA)+∑n∈AD(PWQ,n∥∏ℓ=1tPWn)(A10)<I(YA;YA+WA)+∑n∈A1th(WQ,n)+ε/2(A11)≤I(YA;YA+WA)+∑n∈Ah(Wn)+ε(A12)=I(YA;YA+WA)+h(W)+ε=h(YA+WA)+ε

where Equation (A4) follows by Lemma 1 from [28] and Equations (A10) and (A11) follow since the terms in ${\left(W_{Q,n}\right)}_{n\in A}$ are independent and by Section III of [20]. □

**Lemma** **2.** *For length-t helper observations Y, dithered lattice encodings U of Y using dithers ${\left({\tilde{W}}_{Q,n}\right)}_{n\in [0:N]}$ where each $U_{n}$ encodes $Y_{n}$ onto a regular lattice in $C^{t}$ whose cells have normalized second moment $\sigma_{Q,n}^{2}$. Then, for any ε>0 and if t is large enough, any S⊆[0:N] has*

(A13) $$\left|\frac{1}{t}H\left(U_{S}|{\left({\tilde{W}}_{Q,n}\right)}_{n\in S}\right)-I\left(Y_{S};Z_{S}\left(r\right)\right)\right|<\varepsilon .$$

*In Equations (A13), Z(r)=Y+W, with $Y=\left(Y_{0},\ldots ,Y_{N}\right)\in C^{N+1}$ distributed the same as helper observations at a single time point, and*

(A14) $$\begin{array}{c} W=\left(W_{0},\ldots ,W_{N}\right)∼\mathrm{CN}\left(0,\mathrm{diag}\left(\sigma_{Q,1}^{2},\ldots ,\sigma_{Q,N}^{2}\right)\right). \end{array}$$

**Proof.** We prove this by induction. By Theorem 1 in Reference [29], for any n∈[0:N]

(A15) $$H\left(U_{n}|{\tilde{W}}_{Q,n}\right)=I\left(Y_{n};Y_{n}-{\tilde{W}}_{Q,n}\right).$$

Theorem 6 in Reference [20] has that

(A16) $$\frac{1}{t}I\left(Y_{n};Y_{n}-{\tilde{W}}_{Q,n}\right)\underset{t↑\infty }{\to }I\left(Y_{n};\;Y_{n}+W_{Q,n}\right)=I\left(Y_{n};Z_{n}\left(r_{n}\right)\right).$$

Thus, the statement holds for any singleton {n}. Take any disjoint A,B⊂[0:N] for which the claim holds for $\varepsilon^{\prime }>0$. By construction, given ${\left({\tilde{W}}_{Q,n}\right)}_{n\in A\cup B}$, there is a function $f_{A\cup B}$ where $f_{A\cup B}\left(U_{A\cup B}\right)=Y_{A\cup B}-{\left({\tilde{W}}_{Q,n}\right)}_{n\in A\cup B}^{*}$ with ${\left({\tilde{W}}_{Q,n}\right)}_{n\in A\cup B}^{*}$ independent of but distributed like ${\left({\tilde{W}}_{Q,n}\right)}_{n\in A\cup B}$ [20]. Compute:

(A17)1tH(UA∪B|(W˜Q,n)n∈A∪B)≥1tH(fA∪B(UA∪B)|(W˜Q,n)n∈A∪B)(A18)≥1tI(fA∪B(UA∪B);YA∪B|(W˜Q,n)n∈A∪B)(A19)=1tI(fA∪B(UA∪B);YA∪B)(A20)≥I(YA∪B;ZA∪B(r)).

The first inequality follows by the data processing inequality. Equation (A19) follows since ${\left({\tilde{W}}_{Q,n}\right)}_{n\in A\cup B}$ is independent of the other variables in the term. The last inequality follows by the worst additive noise lemma [27]. For *t* large enough,

(A21)1tH(UA∪B|(W˜Q,n)n∈A∪B)=1t[H(UA|(W˜Q,n)n∈A∪B)+H(UB|(W˜Q,n)n∈A∪B)−I(UA;UB|(W˜Q,n)n∈A∪B)](A22)≤I(YA;ZA(r))+I(YB;ZB(r))−1tI(UA;UB|(W˜Q,n)n∈A∪B)+2ε.

Similar to before, given ${\left({\tilde{W}}_{Q,n}\right)}_{n\in S},S=A,B$ there are deterministic functions $f_{A},f_{B}$ where $f_{A}\left(U_{A}\right)=Y_{A}-{\left({\tilde{W}}_{Q,n}\right)}_{n\in A}^{*},$ and $f_{B}\left(U_{B}\right)=Y_{B}-{\left({\tilde{W}}_{Q,n}\right)}_{n\in B}^{*}$ with ${\left({\tilde{W}}_{Q,n}\right)}_{n\in S}^{*}$ iid to ${\left({\tilde{W}}_{Q,n}\right)}_{n\in S}$, S=A,B. Compute:

(A23)1tI(UA;UB|(W˜Q,n)n∈A∪B)≥1tI(fA(UA);fB(UB)|(W˜Q,n)n∈A∪B)(A24)=1tI(fA(UA);fB(UB))(A25)=1t[h(YA−(W˜Q,n)n∈A*)+h(YB−(W˜Q,n)n∈B*)−…h(YA−(W˜Q,n)n∈A*,YB−(W˜Q,n)n∈B*)](A26)≥h(YA+WA)+h(YB+WB)−h(YA+WA,YB+WB)−3ε′(A27)=I(ZA(r);ZB(r))−3ε′

where, again, the first line follows by the data processing inequality and the second line since the conditional is independent of the rest of the mutual information’s terms. For large enough *t*, the second inequality follows by Lemma 1. Combining Equations (A20), (A22) and (A27),

(A28) $$0\leq H\left(U_{A\cup B}^{t}\right)-I\left(Y_{A\cup B}^{t};Z_{A\cup B}^{t}\left(r\right)\right)\leq 5\varepsilon^{\prime }.$$

Since S⊆[1:N], the inductive step will only need to be used up to *N* times. Letting $0<\varepsilon^{\prime }<\varepsilon \cdot 5^{-N}$, the initial statement holds. □

A small result is also needed to show that the base having its own full precision observation is approximately equivalent to receiving a high-bitrate helper message. Recall that the base is associated with helper index 0.

**Lemma** **3.** *If ∀S⊆[1:N], then*

(A29) $$I\left(Y_{S},Z_{S}\left(r\right)|Z_{S^{C}}\left(r\right),Y_{0}\right)<\sum_{m\in S}r_{m},$$

*then, some value $r_{0}$ can be chosen sufficiently large so that for any S⊆[0:N]*

(A30) $$I\left(Y_{S},Z_{S}\left(r\right)|Z_{S^{C}}\left(r\right)\right)<\sum_{m\in S}r_{m}.$$

**Proof.** Since all the conditional mutual information terms in the statement are continuous in r, it is enough to show the statement for r≻0. In this case, with no loss of generality, we can say there is some δ>0 where:

(A31) $$I\left(Y_{S},Z_{S}\left(r\right)|Z_{S^{C}}\left(r\right),Y_{0}\right)<\sum_{m\in S}r_{m}-\delta .$$

Fix $r_{0}$ large enough so that for any S⊆[1:N],

(A32) $$\begin{array}{c} ∥I\left(Y_{S};Z_{S}\left(r\right)|Z_{S^{C}}\left(r\right),Y_{0}\right)-I\left(Y_{S};Z_{S}\left(r\right)|Z_{S^{C}}\left(r\right),Z_{0}\left(r_{0}\right)\right)∥\leq \delta . \end{array}$$

There is guaranteed to be such an $r_{0}$ because the above expression is a continuous function of the covariance matrix of $\left(Y_{0},Y,Z_{0}\left(r_{0}\right),Z\left(r\right)\right)$ component-wise, and as $r_{0}$ is made large, the covariance matrices involved in the second term converge component-wise to those of the first since $\frac{∥h_{0}{∥}^{2}+\Sigma_{1,1}}{2^{r_{0}}-1}\to 0$ as $r_{0}\to \infty$. By Equations (A31) and (A32), the statement holds for any S⊆[1:N], and it remains to show that it also holds for each S∪{0}. Note that, for any S⊆[1:N],

(A33)≤I(YSt,Y0t;ZSt(r),Z0t(r)|Z(S∪{0})Ct(r))(A34)=I(YSt;ZSt(r)|Z(S∪{0})Ct(r))+I(Y0t;Z0t(r)|Z[1:N]t(r))(A35)≤I(YSt;ZSt(r)|Z(S∪{0})Ct(r))+r0(A36)≤∑m∈Srm+r0

where Equation (A35) follows because conditioning reduces mutual information, and Equation (A36) follows from what was shown at the start. □

**Theorem** **5.** *For a distributed receive system as described in Section 2 with noise covariance matrix* Σ, *LAN constraint L and fixed average helper quantization rates $r\in R_{\mathrm{LAN}}\left(L\right)$, a rate $R_{G}\left(r\right)$ is achievable.*

**Proof.** If some n∈[0:N] has $r_{n}=0$, the system is equivalent to the case where the *n*th receiver’s observation is not present, so, without loss of generality, we assert that $r_{n}>\varepsilon$. Fix some rate $R<R_{G}\left(r\right)$ and a block length $T=t^{2}\in N$. Form a message *M* uniform on $[1:2^{tR}].$

- **Outline**

Form a rate-*R* codebook with length-*T* codewords. At receiver n∈[0:N], form a length-*t* regular lattice encoder with lattice chosen coarse enough that its encodings have rate $r_{n}$. Combine encodings at base and find the typical codeword. Observe that probability of error is low by recognizing that each *t*-length encoding from a helper approximately contains the information content of the helper’s observation plus Gaussian distortion.

- **Transmitter setup**

Generate a codebook mapping $\phi :\left[1:2^{TR}\right]\to C^{T\times 1}$ where:

(A37) $$\phi \left(m\right)=\left(x_{m,1},\ldots ,x_{m,T}\right)\in C^{T\times 1}$$

with each component $x_{m,\ell }$ drawn iid from CN (0,1). Reveal ϕ to the transmitter and base.

- **Helper encoder setup**

For each helper n,n∈[1:N], generate a dither vector ${\tilde{W}}_{Q,n}={\left({\tilde{W}}_{Q,n}^{\ell }\right)}_{\ell }\in {\left(C^{t\times 1}\right)}^{t}$, where each successive *t*-segment is uniform in the base region of the Voroni partition $P_{n}^{t}$ of a regular, white *t*-dimensional lattice $L_{n}^{t}$ which has the following normalized-second-moment:

(A38) $$G^{t}\left(L_{n}^{t},P_{n}^{t}\right)=\frac{∥h_{n}{∥}^{2}+\Sigma_{n,n}}{2^{r_{n}-\varepsilon }-1}.$$

These terms are detailed at the beginning of Reference [20]. Reveal ${\tilde{W}}_{Q,n}$ to the base and helper *n*. Similarly, at the base, generate a dither vector ${\tilde{W}}_{Q,0}={\left({\tilde{W}}_{Q,0}^{\ell }\right)}_{\ell }\in {\left(C^{t\times 1}\right)}^{t}$ with $r_{0}$ chosen large enough for Lemma 3 to hold.

- **Transmission**

To send message $M\in [1:2^{TR}],$ have the transmitter broadcast $X=\left(X_{1},\ldots ,X_{T}\right)=\phi \left(M\right).$

- **Helper encoding and forwarding**

Helper *n* (n∈[0:N]) observes a sequence of length *T*, $Y_{n}={\left(Y_{n}^{\ell }\right)}_{\ell }\in {\left(C^{t\times 1}\right)}^{t}$. Now $Y_{n}-{\tilde{W}}_{Q,n}={\left(Y_{n}^{\ell }-{\tilde{W}}_{Q,n}^{\ell }\right)}_{\ell }\in C^{T}={\left(C^{t}\right)}^{t}$ is composed of *t* consecutive length-*t* segments. For the *ℓ*-th length-*t* segment (ℓ∈[1:t]), form a quantization $U_{n}^{\ell }\in L_{n}^{t}$ by finding the point in $L_{n}^{t}$ associated with the region in $P_{n}^{t}$ in which that segment resides. The properties of such $U_{n}$ are the subject of References [20,29]. By the extension of Theorem 1 in Appendix A of Reference [29],

(A39) $$H\left(U_{n}^{\ell }|{\tilde{W}}_{Q,n}\right)=\frac{1}{t}I\left(Y_{n};Y_{n}-{\tilde{W}}_{Q,n}\right).$$

Further, by Theorem 6 in Reference [20],

(A40) $$\frac{1}{t}I\left(Y_{n};Y_{n}-{\tilde{W}}_{Q,n}\right)\underset{t↑\infty }{\to }I\left(Y_{n};\;Y_{n}+W_{Q,n}\right)=r_{n}-\varepsilon$$

where $Y_{n}$ has the distribution of any individual component of $Y_{n}$ and $W_{Q,n}∼\mathrm{CN}\left(0,G^{t}\left(L_{n}^{t},P_{n}^{t}\right)\right)$ (in agreement with notation in Section 3.2). Thus, the *t* encoded messages produced by helper n,${\left(U_{n}^{\ell }\right)}_{\ell }$, are within the LAN constraint for large enough block length *T*. Helper n∈[1:N] forwards ${\left(U_{n}^{\ell }\right)}_{\ell }$ to the base.

- **Decoding**

At the base, receive all of the lattice quantizations,

(A41) $$U={\left({\left(U_{n}^{\ell }\right)}_{\ell \in [1:t]}\right)}_{n\in [0:N]}.$$

Take $A_{\varepsilon }^{T}\left(X,U|W_{Q}\right)$ to be the collection of

(A42) $$\left(x_{m},u\right)\in \mathrm{Range}\left(\phi \right)\times {\left(\prod_{n=0}^{N}L_{n}^{t}\right)}^{t}$$

which are jointly ε-weakly-typical with respect to the joint distribution of (X,U), conditional on all the dithers ${\left({\tilde{W}}_{Q,n}\right)}_{n\in [0:N]}$. Weak- and joint-typicality are defined in Reference [14]. At the base, find a message estimate $\hat{m}\in \left[1:2^{TR}\right]$ where $\left(\phi \left(\hat{m}\right),U\right)\in A_{\varepsilon }^{T}\left(X,U|W_{Q}\right)$. Declare error events $E_{0}$ if *M* is not found to be typical with U, and $E_{1}$ if there is some $\hat{m}\neq M$ where $\left(\phi \left(\hat{m}\right),U\right)\in A_{\varepsilon }^{T}\left(X,U|W_{Q}\right)$.

- **Error analysis**

By typicality and the law of large numbers, $P(E_{0})\to 0$ as t→∞. In addition,

(A43)P(E1)≤∑m^≠MP{ϕ(m^):(ϕ(m^),U)∈AεT(X,U|WQ)}(A44)≤∑m^≠M2−t·(I(X1,…,Xt;(Un1)n∈[0:N]|(W˜Q,n1)n∈[0:N])−3ε)(A45)<2−t·(I(X1,…,Xt;(Un1)n∈[0:N]|(W˜Q,n1)n∈[0:N])−3ε)+TR(A46)=2−t·(h(X1,…,Xt)−h(X1,…,Xt|(Un1)n∈[0:N],(W˜Q,n1)n,ℓ)−3ε−tR)(A47)≤2−t·(h(X1,…,Xt)−h(X1,…,Xt|(Ynℓ−W˜Q,n1)n)−3ε−tR)(A48)≤2−t·(t·I(X;Z(r−1·ε))−4ε−tR)

Z(·) is defined as in Equation (25). Equation (A47) follows by the data processing inequality. Equation (A48) follows by combining the entropies into a mutual information, then using the worst additive noise lemma [27]. Thus, if *R* is chosen less than I(X;Z(r−1·ε))−4ε then $P(E_{0}\cup E_{1})\to 0$ as T→∞. Mutual information is lower semi-continuous so I(X;Z(r−1·ε))−4ε can be made arbitrarily close to $R_{G}\left(r\right)$. □

**Theorem** **6.** *For a distributed receive system as described in Section 2 with noise covariance matrix* Σ *and LAN constraint L, a rate $R_{DC}\left(L\right)$ is achievable.*

**Proof.** Apply Theorem 7 with λ=0 (proof shown below). □

**Theorem** **7.** *For a distributed receive system as described in Section 2 with noise covariance matrix* Σ *and LAN constraint L then $R_{\bar{DC}}\left(L\right)$ is achievable.*

**Proof.** It is enough to show that any component in the maximization from Equation (36) is achievable. Fix λ>0, a helper rate vector $r\in R_{\mathrm{LAN}}\left(L\right)$, and a compression rate vector $\rho \in R_{DC}^{\lambda }\left(r\right)$. If some n∈[0:N] has $r_{n}=0$ or $\rho_{n}=0$, the system is equivalent to the case where the *n*th helper is not present, so, without loss of generality, we assert that each $r_{n},\rho_{n}>\varepsilon$. Fix some rate $R<R_{G}\left(\rho -1\cdot \varepsilon \right)-\lambda$ and a block length $T=t^{2}\in N$. Form a message *M* uniform on $\left[1:2^{tR}\right]$.

- **Outline**

Form a rate-*R* codebook with length-*T* codewords. At receiver n∈[0:N], form a length-*t* regular lattice encoder with lattice chosen coarse enough that its encodings have rate $r_{n}$. Randomly bin the encodings down to rate $\rho_{n}$. At the base find the codeword jointly typical with the binned encodings. Observe that probability of error is low by recognizing that the unbinned *t*-length encodings from helpers approximately contain the joint-information content of the helper’s observations plus Gaussian distortion.

- **Transmitter setup**

Generate a codebook mapping $\phi :\left[1:2^{TR}\right]\to C^{T\times 1}$ where

(A49) $$\phi \left(m\right)=\left(x_{m,1},\ldots ,x_{m,T}\right)\in C^{T\times 1}$$

with each component $x_{m,\ell }$ drawn iid from CN (0,1). Reveal ϕ to the transmitter and base.

- **Helper encoder setup**

For each helper n,n∈[1:N], generate a dither vector ${\tilde{W}}_{Q,n}={\left({\tilde{W}}_{Q,n}^{\ell }\right)}_{\ell }\in {\left(C^{t\times 1}\right)}^{t}$, where each successive *t*-segment is uniform in the base region of the Voroni partition $P_{n}^{t}$ of a regular, white t−dimensional lattice $L_{n}^{t}$ which has the following normalized-second-moment:

(A50) $$G^{t}\left(L_{n}^{t},P_{n}^{t}\right)=\frac{∥h_{n}{∥}^{2}+\Sigma_{n,n}}{2^{\rho_{n}-\varepsilon }-1}.$$

These terms are detailed at the beginning of Reference [20]. Reveal ${\tilde{W}}_{Q,n}$ to the base and helper *n*. Similarly at the base generate a dither vector ${\tilde{W}}_{Q,0}={\left({\tilde{W}}_{Q,0}^{\ell }\right)}_{\ell }\in {\left(C^{t\times 1}\right)}^{t}$ with $\rho_{0}$ chosen large enough for Lemma 3 to hold. For each receiver n∈[0:N], form a random mapping ${\mathrm{Index}}_{n}:L_{n}^{t}\to \left[1:2^{tr_{n}}\right]$ which takes on iid values at each $u_{n}\in L_{n}^{t}$:

(A51) $${\mathrm{Index}}_{n}\left(u_{n}\right)∼\mathrm{Uniform}\left([1:2^{tr_{n}}]\right)$$

${\mathrm{Index}}_{n}$ is the binning scheme used by receiver n. Distribute each ${\mathrm{Index}}_{n}$ to helper *n* and the base.

- **Transmission**

To send a message $M\in [1:2^{TR}],$ have the transmitter broadcast $X=\left(X_{1},\ldots ,X_{T}\right)=\phi \left(M\right).$

- **Helper encoding**

Helper *n* (n∈[0:N]) observes a sequence of length *T*, $Y_{n}={\left(Y_{n}^{\ell }\right)}_{\ell }\in {\left(C^{t\times 1}\right)}^{t}$. Now $Y_{n}-{\tilde{W}}_{Q,n}={\left(Y_{n}^{\ell }-{\tilde{W}}_{Q,n}^{\ell }\right)}_{\ell }\in C^{T}={\left(C^{t}\right)}^{t}$ is composed of *t* consecutive length-*t* segments. For the *ℓ*-th length-*t* segment, ℓ∈[1:t], form a quantization $U_{n}^{\ell }\in L_{n}^{t}$ by finding the point in $L_{n}^{t}$ associated with the region in $P_{n}^{t}$ in which that segment resides. The properties of such $U_{n}$ are the subject of References [20,29]. Applying Lemma 2, then for large enough *t*, any S⊆[0:N] has

(A52) $$\frac{1}{t}H\left(U_{S}^{\ell }|U_{S^{C}}^{\ell },{\left({\tilde{W}}_{Q,n}\right)}_{n\in [0:N]}\right)\underset{t↑\infty }{\to }I\left(Z_{S}\left(\rho \right);Y_{S}|Z_{S^{C}}\left(\rho \right)\right)<\sum_{n\in S}r_{n}$$

where the right-hand inequality comes from choice of ρ.

- **Helper joint-compression and forwarding**

At receiver n∈[0:N], form binned encodings ${\left(V_{n}^{\ell }\right)}_{\ell \in [1:t]}$ where for each *ℓ*, $V_{n}^{\ell }={\mathrm{Index}}_{n}\left(U_{n}^{\ell }\right)$. Note that $|\mathrm{Range}\left({\mathrm{Index}}_{n}\right)|\leq 2^{t\cdot r_{n}}$ so $H\left(V_{n}^{\ell }\right)\leq t\cdot r_{n}$ and $H\left({\left(V_{n}^{\ell }\right)}_{\ell }\right)\leq T\cdot r_{n}$ so that the LAN constraint is satisfied. Each receiver forwards ${\left(V_{n}^{\ell }\right)}_{\ell }$ to the base.

- **Decoding**

At the base, receive all all the binned helper encodings $V={\left({\left(V_{n}^{\ell }\right)}_{\ell }\right)}_{n}.$ Take $A_{\varepsilon }^{T}\left(X,U|W_{Q}\right)$ to be the set of

(A53) $$\left(x_{m},\left(u^{1},\ldots ,u^{t}\right)\right)\in \mathrm{Range}\left(\phi \right)\times {\left(\prod_{n=0}^{N}L_{n}^{t}\right)}^{t}$$

which are jointly ε-weakly-typical with respect to the joint distribution of (X,U), conditional on all the dithers ${\left({\tilde{W}}_{Q,n}\right)}_{n\in [0:N]}$. Weak- and joint-typicality are defined in Reference [14]. For an ensemble of bin indices from all the receivers, $v={\left({\left(v_{n}^{\ell }\right)}_{\ell }\right)}_{n}\in \prod_{n=0}^{N}{\left[1:2^{t\cdot r_{n}}\right]}^{t}$, define:

(A54) $$\begin{array}{cc} B_{v} & ≜\left\{u\in {\left(\prod_{n=0}^{N}L_{n}^{t}\right)}^{t}|{\mathrm{Index}}_{k}\left(u_{k}^{\ell }\right)=v_{k}^{\ell },k\in \left[0:N\right],\ell \in \left[1:t\right]\right\}. \end{array}$$

Each $B_{v}$ represents the set of helper lattice quantizations represented by the ensemble of helper bin indices v. At the base, find $\hat{m}$ for which there is some $\hat{u}\in B_{V}$ where $\left(\phi \left(\hat{m}\right),\hat{u}\right)\in A_{\varepsilon }^{T}\left(X,U|W_{Q}\right)$. Declare the message associated with $\hat{x}$ to be the broadcast.

- **Error analysis**

We have the following error events:

- $E_{JT}$: X is not typical with anything in $B_{V,[0:N]}$.
- $E_{\hat{m},S}$: For S⊆[0:N], there is some $\hat{m}\neq M$ and $\hat{u}=\left({\hat{u}}_{S},{\hat{u}}_{S^{C}}\right)\in B_{V}$ where $\left(\phi \left(\hat{m}\right),\hat{u}\right)\in A_{\varepsilon }^{T}\left(X,U|W_{Q}\right)$ and ${\hat{u}}_{S^{C}}=U_{S^{C}}$, but any n∈S has ${\hat{u}}_{n}\neq U_{n}$. $E_{\hat{m},S}$ denotes the situation where the base identifies the wrong quantizations for all the receivers in *S*.

By the law of large numbers and properties of typical sets, as *t* becomes large, eventually $P(E_{JT})<\varepsilon$. For each S⊆[0:N], take $A_{\varepsilon }^{T}\left(U|W_{Q}\right)$ to be the collection of jointly-typical sequences in ${\left(\prod_{n}L_{n}^{t}\right)}^{t}$ up to the joint distribution on ${\left(U_{n}^{\ell }\right)}_{n\in S}$ (any ℓ∈[1:t]), conditioned on ${\left({\tilde{W}}_{Q,n}\right)}_{n}$. Now compute:

(A55) $$\begin{array}{cc} \sum_{\hat{m}\neq M}P\left(E_{\hat{m},S}\right) & \leq 2^{TR}\cdot \sum_{\begin{array}{c} \tilde{u}\in A_{\varepsilon }^{T}\left(U|W_{Q}\right):\\ {\tilde{u}}_{S^{C}}=U_{S^{C}} \end{array}}1_{B_{V}}\left(\tilde{u}\right)\cdot P\left(\left(\tilde{X},\tilde{u}\right)\in A_{\varepsilon }^{T}\left(X,U|W_{Q}\right)\right) \end{array}$$

where $\tilde{X}$ is a random variable independent of and distributed identically to X. Then, with high probability for large enough *t*,

(A56)∑m^≠MP(Em^,S)≤2TR·∑u˜∈AεT(U|WQ):u˜SC=USC1BV(u˜)·2−t·(I(X;U|(WQ,n)n)−3ε)(A57)≤2TR·{u˜∈AεT(U|WQ)|u˜SC=USC}·(1+ε)·P(U∈BV)·2−t·(I(X;U|(WQ,n)n)−3ε)(A58)≤2TR·2t·(H(US|USC,(WQ,n)n)+ε)·2−t·(t·∑n∈Srn−ε)·2−t·(I(X;U|(WQ,nℓ)n)−3ε)

where Equation (A58) follows by construction of $B_{V}$. Taking the log,

(A59)log∑m^≠MP(Em^,S)(A60)≤TR+t·(H(US|USC,(WQ,n)n)+ε)−t·(t·∑n∈Srn−ε)−t·I(X;U|(WQ,n)n)−3ε+ε(A61)≤t·(H(US|USC,(WQ,n)n)−t·∑n∈Srn+tR−I(X;U|(WQ,n)n)+6ε)(A62)≤t2·(I(YS;ZS(ρ−1·ε)|ZSC(ρ−1·ε))−∑n∈Srn+R−I(X;Z(ρ−1·ε))+8ε/t).

Equation (A62) follows by using Lemma 2 on $H(U_{S}|U_{S^{C}})/t$ and using reasoning identical to Equations (A45)–(A48) on $I(X;U|{\left(W_{Q,n}\right)}_{n})$. Recall that *R* was chosen so that $R\leq R_{G}\left(\rho -1\cdot \varepsilon \right)-\lambda =I\left(X;Z\left(\rho -1\cdot \varepsilon \right)\right)-\lambda$. Then, Equation (A62) will approach −∞ as t→∞ (thereby $P(\cup_{\hat{m},S}E_{\hat{m},S})$ approaches 0) if all S⊆[0:N] satisfy:

(A63) $$I\left(Y_{S};Z_{S}\left(\rho -1\cdot \varepsilon \right)|Z_{S^{C}}\left(\rho -1\cdot \varepsilon \right)\right)<\lambda +\sum_{m\in S}r_{m}-8\varepsilon .$$

By assumption that $\rho \in R_{DC}^{\lambda }\left(r\right)$ and for small enough ε, then Equation (A63) holds for each *S*. Since all error events approach 0, then a rate of $R_{G}\left(\rho -1\cdot \varepsilon \right)-\lambda$ is achievable. For small enough ε, by lower semi-continuity of mutual information $R_{G}\left(\rho -1\cdot \varepsilon \right)-\lambda$ can be made arbitrarily close to $R_{G}\left(\rho \right)-\lambda$. □

## Appendix C. Proof of Conditional Capacity

**Remark** **7.** *The capacity of the system is $R_{\bar{DC}}\left(L\right)$ under the following restrictions:*

- Σ *is diagonal (no interference).*
- The base does not have its own full-precision observation of the broadcast ($h_{0}=0$).
- The broadcaster must transmit a Gaussian signal.
- Construction of helper messages is independent of the transmitter’s codebook X.

**Proof.** By Theorem 5 in Reference [1] (variable names altered, constants adapted for complex variables), the capacity of the system under the assumed restrictions is

(A64) $$\max_{\begin{array}{c} r\in R_{\mathrm{LAN}}\left(L\right),\\ V\in V \end{array}}C_{r,V}$$

(A65) $$C_{r,V}≜\min_{S\subseteq [1:N]}\left\{\sum_{m\in S}\left[r_{m}-I\left(V_{m};Y_{m}|X\right)\right]+I\left(V_{S^{C}};X\right)\right\}.$$

In Equation (A65), V is the collection of random vectors $V=(V_{1},\ldots ,V_{N})$ whose components are of the form $V_{n}=Y_{n}+{\tilde{W}}_{n}$ with independently distributed ${\tilde{W}}_{n}$:

(A66) $${\tilde{W}}_{n}∼\mathrm{CN}\left(0,\sigma_{n}^{2}\cdot \frac{2^{-2v_{n}}}{1-2^{-2v_{n}}}\right)$$

for any $v_{n}\geq 0$. Since both the variance in Equation (A66) as a function of $v_{n}$ and Equation (26) as a function of $r_{n}$ are injective on (0,∞), then for any V∈V there is some ρ (in the context of Equation (37); either inside or outside $\cup_{\lambda \in R}R_{DC}^{\lambda }\left(L\right)$) which will yield a variable Z(ρ) with identical distribution to V. Fixing helper rates r, for any S⊆[1:N], we can instead write $C_{r,V}$ as:

(A67)Cr,V=maxc:∀S⊆[1:N],≤c≤I(VSC;X)+∑m∈Srm−I(Vm;Ym|X)(A68)≤maxc:∀S⊆[1:N],≤c−I(VS;X)+I(VS;YS|X)≤∑m∈Srm(A69)≤maxc:∀S⊆[1:N],≤[c−I(X;V)]+I(YS;VS|VSC)≤∑m∈Srm(A70)=maxλ∈RmaxI(X;V)−λ:∀S⊆[1:N]I(YS;VS|VSC)≤∑m∈Srm+λ

where $I\left(V_{S};Y_{S}|X\right)\leq \sum_{m\in S}I\left(V_{m};Y_{m}|X\right)$ follows because, for each *n*, $V_{n}↔\left(X,Y_{n}\right)↔\left(V_{\left\{j\neq n\right\}},Y_{\left\{j\neq n\right\}}\right)$ is a Markov chain. Maximizing over r, Equation (A70) is identical to the set of rates shown in Theorem 7. □
