# Supplementary material for: A Decentralized Receiver in Gaussian Interference
Source: Entropy (Basel). 2018 Apr 11;20(4):269. doi: 10.3390/e20040269 (PMC7512784; doi:10.3390/e20040269)
Supplement: Supplementary file 1 [file entropy-20-00269-s001.zip › dist_recv/checkdc.html]

Cython: checkdc.pyx


Generated by Cython 0.25.2

Yellow lines hint at Python interaction.  
Click on a line that starts with a "`+`" to see the C code that Cython generated for it.

Raw output: checkdc.c

```
 001: """Check whether a distributed compression configuration violates entropy conditions"""
```

```
+002: import numpy as np
```

```
  __pyx_t_1 = __Pyx_Import(__pyx_n_s_numpy, 0, -1); if (unlikely(!__pyx_t_1)) __PYX_ERR(0, 2, __pyx_L1_error)
  __Pyx_GOTREF(__pyx_t_1);
  if (PyDict_SetItem(__pyx_d, __pyx_n_s_np, __pyx_t_1) < 0) __PYX_ERR(0, 2, __pyx_L1_error)
  __Pyx_DECREF(__pyx_t_1); __pyx_t_1 = 0;
```

```
 003:
```

```
+004: D_MIN_USEFUL_BITRATE = 0.01
```

```
  if (PyDict_SetItem(__pyx_d, __pyx_n_s_D_MIN_USEFUL_BITRATE, __pyx_float_0_01) < 0) __PYX_ERR(0, 4, __pyx_L1_error)
```

```
 005:
```

```
 006:
```

```
+007: def checkdc(v_channel, nda_interf, v_dc_bits, v_ga_bits, d_lambda):
```

```
/* Python wrapper */
static PyObject *__pyx_pw_9dist_recv_7checkdc_1checkdc(PyObject *__pyx_self, PyObject *__pyx_args, PyObject *__pyx_kwds); /*proto*/
static char __pyx_doc_9dist_recv_7checkdc_checkdc[] = " Check whether a distributed compression configuration violates entropy conditions\n        (continuous version)\n         Input:\n             v_channel = Channel vector h (size N+1 x 1)\n             nda_interf = Interference covariance matrix Sigma (size N+1 x N+1)\n             v_dc_bits = Post-compression helper encoding rates. (size N x 1)\n             v_ga_bits = Pre-compression Gaussian encoder rates. (size N x 1)\n             d_lambda = Hull parameter\n         Output:\n             b_is_sane = True only if v_dc_bits is valid given all the other inputs\n     ";
static PyMethodDef __pyx_mdef_9dist_recv_7checkdc_1checkdc = {"checkdc", (PyCFunction)__pyx_pw_9dist_recv_7checkdc_1checkdc, METH_VARARGS|METH_KEYWORDS, __pyx_doc_9dist_recv_7checkdc_checkdc};
static PyObject *__pyx_pw_9dist_recv_7checkdc_1checkdc(PyObject *__pyx_self, PyObject *__pyx_args, PyObject *__pyx_kwds) {
  PyObject *__pyx_v_v_channel = 0;
  PyObject *__pyx_v_nda_interf = 0;
  PyObject *__pyx_v_v_dc_bits = 0;
  PyObject *__pyx_v_v_ga_bits = 0;
  PyObject *__pyx_v_d_lambda = 0;
  PyObject *__pyx_r = 0;
  __Pyx_RefNannyDeclarations
  __Pyx_RefNannySetupContext("checkdc (wrapper)", 0);
  {
    static PyObject **__pyx_pyargnames[] = {&__pyx_n_s_v_channel,&__pyx_n_s_nda_interf,&__pyx_n_s_v_dc_bits,&__pyx_n_s_v_ga_bits,&__pyx_n_s_d_lambda,0};
    PyObject* values[5] = {0,0,0,0,0};
    if (unlikely(__pyx_kwds)) {
      Py_ssize_t kw_args;
      const Py_ssize_t pos_args = PyTuple_GET_SIZE(__pyx_args);
      switch (pos_args) {
        case  5: values[4] = PyTuple_GET_ITEM(__pyx_args, 4);
        case  4: values[3] = PyTuple_GET_ITEM(__pyx_args, 3);
        case  3: values[2] = PyTuple_GET_ITEM(__pyx_args, 2);
        case  2: values[1] = PyTuple_GET_ITEM(__pyx_args, 1);
        case  1: values[0] = PyTuple_GET_ITEM(__pyx_args, 0);
        case  0: break;
        default: goto __pyx_L5_argtuple_error;
      }
      kw_args = PyDict_Size(__pyx_kwds);
      switch (pos_args) {
        case  0:
        if (likely((values[0] = PyDict_GetItem(__pyx_kwds, __pyx_n_s_v_channel)) != 0)) kw_args--;
        else goto __pyx_L5_argtuple_error;
        case  1:
        if (likely((values[1] = PyDict_GetItem(__pyx_kwds, __pyx_n_s_nda_interf)) != 0)) kw_args--;
        else {
          __Pyx_RaiseArgtupleInvalid("checkdc", 1, 5, 5, 1); __PYX_ERR(0, 7, __pyx_L3_error)
        }
        case  2:
        if (likely((values[2] = PyDict_GetItem(__pyx_kwds, __pyx_n_s_v_dc_bits)) != 0)) kw_args--;
        else {
          __Pyx_RaiseArgtupleInvalid("checkdc", 1, 5, 5, 2); __PYX_ERR(0, 7, __pyx_L3_error)
        }
        case  3:
        if (likely((values[3] = PyDict_GetItem(__pyx_kwds, __pyx_n_s_v_ga_bits)) != 0)) kw_args--;
        else {
          __Pyx_RaiseArgtupleInvalid("checkdc", 1, 5, 5, 3); __PYX_ERR(0, 7, __pyx_L3_error)
        }
        case  4:
        if (likely((values[4] = PyDict_GetItem(__pyx_kwds, __pyx_n_s_d_lambda)) != 0)) kw_args--;
        else {
          __Pyx_RaiseArgtupleInvalid("checkdc", 1, 5, 5, 4); __PYX_ERR(0, 7, __pyx_L3_error)
        }
      }
      if (unlikely(kw_args > 0)) {
        if (unlikely(__Pyx_ParseOptionalKeywords(__pyx_kwds, __pyx_pyargnames, 0, values, pos_args, "checkdc") < 0)) __PYX_ERR(0, 7, __pyx_L3_error)
      }
    } else if (PyTuple_GET_SIZE(__pyx_args) != 5) {
      goto __pyx_L5_argtuple_error;
    } else {
      values[0] = PyTuple_GET_ITEM(__pyx_args, 0);
      values[1] = PyTuple_GET_ITEM(__pyx_args, 1);
      values[2] = PyTuple_GET_ITEM(__pyx_args, 2);
      values[3] = PyTuple_GET_ITEM(__pyx_args, 3);
      values[4] = PyTuple_GET_ITEM(__pyx_args, 4);
    }
    __pyx_v_v_channel = values[0];
    __pyx_v_nda_interf = values[1];
    __pyx_v_v_dc_bits = values[2];
    __pyx_v_v_ga_bits = values[3];
    __pyx_v_d_lambda = values[4];
  }
  goto __pyx_L4_argument_unpacking_done;
  __pyx_L5_argtuple_error:;
  __Pyx_RaiseArgtupleInvalid("checkdc", 1, 5, 5, PyTuple_GET_SIZE(__pyx_args)); __PYX_ERR(0, 7, __pyx_L3_error)
  __pyx_L3_error:;
  __Pyx_AddTraceback("dist_recv.checkdc.checkdc", __pyx_clineno, __pyx_lineno, __pyx_filename);
  __Pyx_RefNannyFinishContext();
  return NULL;
  __pyx_L4_argument_unpacking_done:;
  __pyx_r = __pyx_pf_9dist_recv_7checkdc_checkdc(__pyx_self, __pyx_v_v_channel, __pyx_v_nda_interf, __pyx_v_v_dc_bits, __pyx_v_v_ga_bits, __pyx_v_d_lambda);

  /* function exit code */
  __Pyx_RefNannyFinishContext();
  return __pyx_r;
}

static PyObject *__pyx_pf_9dist_recv_7checkdc_checkdc(CYTHON_UNUSED PyObject *__pyx_self, PyObject *__pyx_v_v_channel, PyObject *__pyx_v_nda_interf, PyObject *__pyx_v_v_dc_bits, PyObject *__pyx_v_v_ga_bits, PyObject *__pyx_v_d_lambda) {
  PyObject *__pyx_v_v_badness = NULL;
  PyObject *__pyx_v_b_is_sane = NULL;
  PyObject *__pyx_r = NULL;
  __Pyx_TraceDeclarations
  __Pyx_TraceFrameInit(__pyx_codeobj_)
  __Pyx_RefNannyDeclarations
  __Pyx_RefNannySetupContext("checkdc", 0);
  __Pyx_TraceCall("checkdc", __pyx_f[0], 7, 0, __PYX_ERR(0, 7, __pyx_L1_error));
/* … */
  /* function exit code */
  __pyx_L1_error:;
  __Pyx_XDECREF(__pyx_t_1);
  __Pyx_XDECREF(__pyx_t_2);
  __Pyx_XDECREF(__pyx_t_3);
  __Pyx_XDECREF(__pyx_t_5);
  __Pyx_AddTraceback("dist_recv.checkdc.checkdc", __pyx_clineno, __pyx_lineno, __pyx_filename);
  __pyx_r = NULL;
  __pyx_L0:;
  __Pyx_XDECREF(__pyx_v_v_badness);
  __Pyx_XDECREF(__pyx_v_b_is_sane);
  __Pyx_XGIVEREF(__pyx_r);
  __Pyx_TraceReturn(__pyx_r, 0);
  __Pyx_RefNannyFinishContext();
  return __pyx_r;
}
/* … */
  __pyx_tuple__7 = PyTuple_Pack(7, __pyx_n_s_v_channel, __pyx_n_s_nda_interf, __pyx_n_s_v_dc_bits, __pyx_n_s_v_ga_bits, __pyx_n_s_d_lambda, __pyx_n_s_v_badness, __pyx_n_s_b_is_sane); if (unlikely(!__pyx_tuple__7)) __PYX_ERR(0, 7, __pyx_L1_error)
  __Pyx_GOTREF(__pyx_tuple__7);
  __Pyx_GIVEREF(__pyx_tuple__7);
/* … */
  __pyx_t_1 = PyCFunction_NewEx(&__pyx_mdef_9dist_recv_7checkdc_1checkdc, NULL, __pyx_n_s_dist_recv_checkdc); if (unlikely(!__pyx_t_1)) __PYX_ERR(0, 7, __pyx_L1_error)
  __Pyx_GOTREF(__pyx_t_1);
  if (PyDict_SetItem(__pyx_d, __pyx_n_s_checkdc, __pyx_t_1) < 0) __PYX_ERR(0, 7, __pyx_L1_error)
  __Pyx_DECREF(__pyx_t_1); __pyx_t_1 = 0;
  __pyx_codeobj_ = (PyObject*)__Pyx_PyCode_New(5, 0, 7, 0, 0, __pyx_empty_bytes, __pyx_empty_tuple, __pyx_empty_tuple, __pyx_tuple__7, __pyx_empty_tuple, __pyx_empty_tuple, __pyx_kp_s_Users_cdchapm2_Dropbox_Personal, __pyx_n_s_checkdc, 7, __pyx_empty_bytes); if (unlikely(!__pyx_codeobj_)) __PYX_ERR(0, 7, __pyx_L1_error)
```

```
 008:     """ Check whether a distributed compression configuration violates entropy conditions
```

```
 009:         (continuous version)
```

```
 010:          Input:
```

```
 011:              v_channel = Channel vector h (size N+1 x 1)
```

```
 012:              nda_interf = Interference covariance matrix Sigma (size N+1 x N+1)
```

```
 013:              v_dc_bits = Post-compression helper encoding rates. (size N x 1)
```

```
 014:              v_ga_bits = Pre-compression Gaussian encoder rates. (size N x 1)
```

```
 015:              d_lambda = Hull parameter
```

```
 016:          Output:
```

```
 017:              b_is_sane = True only if v_dc_bits is valid given all the other inputs
```

```
 018:      """
```

```
+019:     v_badness = checkdc_cts(v_channel, nda_interf, v_dc_bits, v_ga_bits, d_lambda)
```

```
  __pyx_t_2 = __Pyx_GetModuleGlobalName(__pyx_n_s_checkdc_cts); if (unlikely(!__pyx_t_2)) __PYX_ERR(0, 19, __pyx_L1_error)
  __Pyx_GOTREF(__pyx_t_2);
  __pyx_t_3 = NULL;
  __pyx_t_4 = 0;
  if (CYTHON_UNPACK_METHODS && unlikely(PyMethod_Check(__pyx_t_2))) {
    __pyx_t_3 = PyMethod_GET_SELF(__pyx_t_2);
    if (likely(__pyx_t_3)) {
      PyObject* function = PyMethod_GET_FUNCTION(__pyx_t_2);
      __Pyx_INCREF(__pyx_t_3);
      __Pyx_INCREF(function);
      __Pyx_DECREF_SET(__pyx_t_2, function);
      __pyx_t_4 = 1;
    }
  }
  #if CYTHON_FAST_PYCALL
  if (PyFunction_Check(__pyx_t_2)) {
    PyObject *__pyx_temp[6] = {__pyx_t_3, __pyx_v_v_channel, __pyx_v_nda_interf, __pyx_v_v_dc_bits, __pyx_v_v_ga_bits, __pyx_v_d_lambda};
    __pyx_t_1 = __Pyx_PyFunction_FastCall(__pyx_t_2, __pyx_temp+1-__pyx_t_4, 5+__pyx_t_4); if (unlikely(!__pyx_t_1)) __PYX_ERR(0, 19, __pyx_L1_error)
    __Pyx_XDECREF(__pyx_t_3); __pyx_t_3 = 0;
    __Pyx_GOTREF(__pyx_t_1);
  } else
  #endif
  #if CYTHON_FAST_PYCCALL
  if (__Pyx_PyFastCFunction_Check(__pyx_t_2)) {
    PyObject *__pyx_temp[6] = {__pyx_t_3, __pyx_v_v_channel, __pyx_v_nda_interf, __pyx_v_v_dc_bits, __pyx_v_v_ga_bits, __pyx_v_d_lambda};
    __pyx_t_1 = __Pyx_PyCFunction_FastCall(__pyx_t_2, __pyx_temp+1-__pyx_t_4, 5+__pyx_t_4); if (unlikely(!__pyx_t_1)) __PYX_ERR(0, 19, __pyx_L1_error)
    __Pyx_XDECREF(__pyx_t_3); __pyx_t_3 = 0;
    __Pyx_GOTREF(__pyx_t_1);
  } else
  #endif
  {
    __pyx_t_5 = PyTuple_New(5+__pyx_t_4); if (unlikely(!__pyx_t_5)) __PYX_ERR(0, 19, __pyx_L1_error)
    __Pyx_GOTREF(__pyx_t_5);
    if (__pyx_t_3) {
      __Pyx_GIVEREF(__pyx_t_3); PyTuple_SET_ITEM(__pyx_t_5, 0, __pyx_t_3); __pyx_t_3 = NULL;
    }
    __Pyx_INCREF(__pyx_v_v_channel);
    __Pyx_GIVEREF(__pyx_v_v_channel);
    PyTuple_SET_ITEM(__pyx_t_5, 0+__pyx_t_4, __pyx_v_v_channel);
    __Pyx_INCREF(__pyx_v_nda_interf);
    __Pyx_GIVEREF(__pyx_v_nda_interf);
    PyTuple_SET_ITEM(__pyx_t_5, 1+__pyx_t_4, __pyx_v_nda_interf);
    __Pyx_INCREF(__pyx_v_v_dc_bits);
    __Pyx_GIVEREF(__pyx_v_v_dc_bits);
    PyTuple_SET_ITEM(__pyx_t_5, 2+__pyx_t_4, __pyx_v_v_dc_bits);
    __Pyx_INCREF(__pyx_v_v_ga_bits);
    __Pyx_GIVEREF(__pyx_v_v_ga_bits);
    PyTuple_SET_ITEM(__pyx_t_5, 3+__pyx_t_4, __pyx_v_v_ga_bits);
    __Pyx_INCREF(__pyx_v_d_lambda);
    __Pyx_GIVEREF(__pyx_v_d_lambda);
    PyTuple_SET_ITEM(__pyx_t_5, 4+__pyx_t_4, __pyx_v_d_lambda);
    __pyx_t_1 = __Pyx_PyObject_Call(__pyx_t_2, __pyx_t_5, NULL); if (unlikely(!__pyx_t_1)) __PYX_ERR(0, 19, __pyx_L1_error)
    __Pyx_GOTREF(__pyx_t_1);
    __Pyx_DECREF(__pyx_t_5); __pyx_t_5 = 0;
  }
  __Pyx_DECREF(__pyx_t_2); __pyx_t_2 = 0;
  __pyx_v_v_badness = __pyx_t_1;
  __pyx_t_1 = 0;
```

```
+020:     b_is_sane = (max(v_badness) <= 0)
```

```
  __pyx_t_1 = PyTuple_New(1); if (unlikely(!__pyx_t_1)) __PYX_ERR(0, 20, __pyx_L1_error)
  __Pyx_GOTREF(__pyx_t_1);
  __Pyx_INCREF(__pyx_v_v_badness);
  __Pyx_GIVEREF(__pyx_v_v_badness);
  PyTuple_SET_ITEM(__pyx_t_1, 0, __pyx_v_v_badness);
  __pyx_t_2 = __Pyx_PyObject_Call(__pyx_builtin_max, __pyx_t_1, NULL); if (unlikely(!__pyx_t_2)) __PYX_ERR(0, 20, __pyx_L1_error)
  __Pyx_GOTREF(__pyx_t_2);
  __Pyx_DECREF(__pyx_t_1); __pyx_t_1 = 0;
  __pyx_t_1 = PyObject_RichCompare(__pyx_t_2, __pyx_int_0, Py_LE); __Pyx_XGOTREF(__pyx_t_1); if (unlikely(!__pyx_t_1)) __PYX_ERR(0, 20, __pyx_L1_error)
  __Pyx_DECREF(__pyx_t_2); __pyx_t_2 = 0;
  __pyx_v_b_is_sane = __pyx_t_1;
  __pyx_t_1 = 0;
```

```
+021:     return b_is_sane
```

```
  __Pyx_XDECREF(__pyx_r);
  __Pyx_INCREF(__pyx_v_b_is_sane);
  __pyx_r = __pyx_v_b_is_sane;
  goto __pyx_L0;
```

```
 022:
```

```
 023:
```

```
+024: def checkdc_cts(v_channel, nda_interf, v_dc_bits, v_ga_bits, d_lambda):
```

```
/* Python wrapper */
static PyObject *__pyx_pw_9dist_recv_7checkdc_3checkdc_cts(PyObject *__pyx_self, PyObject *__pyx_args, PyObject *__pyx_kwds); /*proto*/
static char __pyx_doc_9dist_recv_7checkdc_2checkdc_cts[] = " Check whether a distributed compression configuration violates entropy conditions\n        (continuous version)\n         Input:\n             v_channel = Channel vector h (size N+1 x 1)\n             nda_interf = Interference covariance matrix Sigma (size N+1 x N+1)\n             v_dc_bits = Post-compression helper encoding rates. (size N x 1)\n             v_ga_bits = Pre-compression Gaussian encoder rates. (size N x 1)\n             d_lambda = Hull parameter\n         Output:\n             v_badness = violation amounts\n     ";
static PyMethodDef __pyx_mdef_9dist_recv_7checkdc_3checkdc_cts = {"checkdc_cts", (PyCFunction)__pyx_pw_9dist_recv_7checkdc_3checkdc_cts, METH_VARARGS|METH_KEYWORDS, __pyx_doc_9dist_recv_7checkdc_2checkdc_cts};
static PyObject *__pyx_pw_9dist_recv_7checkdc_3checkdc_cts(PyObject *__pyx_self, PyObject *__pyx_args, PyObject *__pyx_kwds) {
  PyObject *__pyx_v_v_channel = 0;
  PyObject *__pyx_v_nda_interf = 0;
  PyObject *__pyx_v_v_dc_bits = 0;
  PyObject *__pyx_v_v_ga_bits = 0;
  PyObject *__pyx_v_d_lambda = 0;
  PyObject *__pyx_r = 0;
  __Pyx_RefNannyDeclarations
  __Pyx_RefNannySetupContext("checkdc_cts (wrapper)", 0);
  {
    static PyObject **__pyx_pyargnames[] = {&__pyx_n_s_v_channel,&__pyx_n_s_nda_interf,&__pyx_n_s_v_dc_bits,&__pyx_n_s_v_ga_bits,&__pyx_n_s_d_lambda,0};
    PyObject* values[5] = {0,0,0,0,0};
    if (unlikely(__pyx_kwds)) {
      Py_ssize_t kw_args;
      const Py_ssize_t pos_args = PyTuple_GET_SIZE(__pyx_args);
      switch (pos_args) {
        case  5: values[4] = PyTuple_GET_ITEM(__pyx_args, 4);
        case  4: values[3] = PyTuple_GET_ITEM(__pyx_args, 3);
        case  3: values[2] = PyTuple_GET_ITEM(__pyx_args, 2);
        case  2: values[1] = PyTuple_GET_ITEM(__pyx_args, 1);
        case  1: values[0] = PyTuple_GET_ITEM(__pyx_args, 0);
        case  0: break;
        default: goto __pyx_L5_argtuple_error;
      }
      kw_args = PyDict_Size(__pyx_kwds);
      switch (pos_args) {
        case  0:
        if (likely((values[0] = PyDict_GetItem(__pyx_kwds, __pyx_n_s_v_channel)) != 0)) kw_args--;
        else goto __pyx_L5_argtuple_error;
        case  1:
        if (likely((values[1] = PyDict_GetItem(__pyx_kwds, __pyx_n_s_nda_interf)) != 0)) kw_args--;
        else {
          __Pyx_RaiseArgtupleInvalid("checkdc_cts", 1, 5, 5, 1); __PYX_ERR(0, 24, __pyx_L3_error)
        }
        case  2:
        if (likely((values[2] = PyDict_GetItem(__pyx_kwds, __pyx_n_s_v_dc_bits)) != 0)) kw_args--;
        else {
          __Pyx_RaiseArgtupleInvalid("checkdc_cts", 1, 5, 5, 2); __PYX_ERR(0, 24, __pyx_L3_error)
        }
        case  3:
        if (likely((values[3] = PyDict_GetItem(__pyx_kwds, __pyx_n_s_v_ga_bits)) != 0)) kw_args--;
        else {
          __Pyx_RaiseArgtupleInvalid("checkdc_cts", 1, 5, 5, 3); __PYX_ERR(0, 24, __pyx_L3_error)
        }
        case  4:
        if (likely((values[4] = PyDict_GetItem(__pyx_kwds, __pyx_n_s_d_lambda)) != 0)) kw_args--;
        else {
          __Pyx_RaiseArgtupleInvalid("checkdc_cts", 1, 5, 5, 4); __PYX_ERR(0, 24, __pyx_L3_error)
        }
      }
      if (unlikely(kw_args > 0)) {
        if (unlikely(__Pyx_ParseOptionalKeywords(__pyx_kwds, __pyx_pyargnames, 0, values, pos_args, "checkdc_cts") < 0)) __PYX_ERR(0, 24, __pyx_L3_error)
      }
    } else if (PyTuple_GET_SIZE(__pyx_args) != 5) {
      goto __pyx_L5_argtuple_error;
    } else {
      values[0] = PyTuple_GET_ITEM(__pyx_args, 0);
      values[1] = PyTuple_GET_ITEM(__pyx_args, 1);
      values[2] = PyTuple_GET_ITEM(__pyx_args, 2);
      values[3] = PyTuple_GET_ITEM(__pyx_args, 3);
      values[4] = PyTuple_GET_ITEM(__pyx_args, 4);
    }
    __pyx_v_v_channel = values[0];
    __pyx_v_nda_interf = values[1];
    __pyx_v_v_dc_bits = values[2];
    __pyx_v_v_ga_bits = values[3];
    __pyx_v_d_lambda = values[4];
  }
  goto __pyx_L4_argument_unpacking_done;
  __pyx_L5_argtuple_error:;
  __Pyx_RaiseArgtupleInvalid("checkdc_cts", 1, 5, 5, PyTuple_GET_SIZE(__pyx_args)); __PYX_ERR(0, 24, __pyx_L3_error)
  __pyx_L3_error:;
  __Pyx_AddTraceback("dist_recv.checkdc.checkdc_cts", __pyx_clineno, __pyx_lineno, __pyx_filename);
  __Pyx_RefNannyFinishContext();
  return NULL;
  __pyx_L4_argument_unpacking_done:;
  __pyx_r = __pyx_pf_9dist_recv_7checkdc_2checkdc_cts(__pyx_self, __pyx_v_v_channel, __pyx_v_nda_interf, __pyx_v_v_dc_bits, __pyx_v_v_ga_bits, __pyx_v_d_lambda);

  /* function exit code */
  __Pyx_RefNannyFinishContext();
  return __pyx_r;
}

static PyObject *__pyx_pf_9dist_recv_7checkdc_2checkdc_cts(CYTHON_UNUSED PyObject *__pyx_self, PyObject *__pyx_v_v_channel, PyObject *__pyx_v_nda_interf, PyObject *__pyx_v_v_dc_bits, PyObject *__pyx_v_v_ga_bits, PyObject *__pyx_v_d_lambda) {
  PyObject *__pyx_v_v_dc_bits_u = NULL;
  PyObject *__pyx_v_v_ga_bits_u = NULL;
  PyObject *__pyx_v_bv_using = NULL;
  PyObject *__pyx_v_n_using = NULL;
  PyObject *__pyx_v_v_channel_u = NULL;
  PyObject *__pyx_v_nda_interf_u = NULL;
  PyObject *__pyx_v_nda_receiver_noise = NULL;
  PyObject *__pyx_v_nda_receiver_signal = NULL;
  PyObject *__pyx_v_v_helper_distortion = NULL;
  PyObject *__pyx_v_nda_total_noise = NULL;
  PyObject *__pyx_v_v_s_n_dets = NULL;
  PyObject *__pyx_v_v_cond_ents = NULL;
  PyObject *__pyx_v_v_badness = NULL;
  PyObject *__pyx_v_n_set = NULL;
  PyObject *__pyx_v_n_set_c = NULL;
  PyObject *__pyx_v_bv_set = NULL;
  PyObject *__pyx_v_d_entropy_include = NULL;
  PyObject *__pyx_v_d_entropy_exclude = NULL;
  PyObject *__pyx_v_n_in_set = NULL;
  PyObject *__pyx_v_n_set_kliii = NULL;
  PyObject *__pyx_v_iii = NULL;
  PyObject *__pyx_v_n_set_iii = NULL;
  PyObject *__pyx_v_n_set_kleqiii = NULL;
  PyObject *__pyx_v_d_mi = NULL;
  PyObject *__pyx_v_d_cond_ent = NULL;
  PyObject *__pyx_r = NULL;
  __Pyx_TraceDeclarations
  __Pyx_TraceFrameInit(__pyx_codeobj__2)
  __Pyx_RefNannyDeclarations
  __Pyx_RefNannySetupContext("checkdc_cts", 0);
  __Pyx_TraceCall("checkdc_cts", __pyx_f[0], 24, 0, __PYX_ERR(0, 24, __pyx_L1_error));
/* … */
  /* function exit code */
  __pyx_L1_error:;
  __Pyx_XDECREF(__pyx_t_1);
  __Pyx_XDECREF(__pyx_t_2);
  __Pyx_XDECREF(__pyx_t_3);
  __Pyx_XDECREF(__pyx_t_4);
  __Pyx_XDECREF(__pyx_t_6);
  __Pyx_XDECREF(__pyx_t_8);
  __Pyx_XDECREF(__pyx_t_9);
  __Pyx_AddTraceback("dist_recv.checkdc.checkdc_cts", __pyx_clineno, __pyx_lineno, __pyx_filename);
  __pyx_r = NULL;
  __pyx_L0:;
  __Pyx_XDECREF(__pyx_v_v_dc_bits_u);
  __Pyx_XDECREF(__pyx_v_v_ga_bits_u);
  __Pyx_XDECREF(__pyx_v_bv_using);
  __Pyx_XDECREF(__pyx_v_n_using);
  __Pyx_XDECREF(__pyx_v_v_channel_u);
  __Pyx_XDECREF(__pyx_v_nda_interf_u);
  __Pyx_XDECREF(__pyx_v_nda_receiver_noise);
  __Pyx_XDECREF(__pyx_v_nda_receiver_signal);
  __Pyx_XDECREF(__pyx_v_v_helper_distortion);
  __Pyx_XDECREF(__pyx_v_nda_total_noise);
  __Pyx_XDECREF(__pyx_v_v_s_n_dets);
  __Pyx_XDECREF(__pyx_v_v_cond_ents);
  __Pyx_XDECREF(__pyx_v_v_badness);
  __Pyx_XDECREF(__pyx_v_n_set);
  __Pyx_XDECREF(__pyx_v_n_set_c);
  __Pyx_XDECREF(__pyx_v_bv_set);
  __Pyx_XDECREF(__pyx_v_d_entropy_include);
  __Pyx_XDECREF(__pyx_v_d_entropy_exclude);
  __Pyx_XDECREF(__pyx_v_n_in_set);
  __Pyx_XDECREF(__pyx_v_n_set_kliii);
  __Pyx_XDECREF(__pyx_v_iii);
  __Pyx_XDECREF(__pyx_v_n_set_iii);
  __Pyx_XDECREF(__pyx_v_n_set_kleqiii);
  __Pyx_XDECREF(__pyx_v_d_mi);
  __Pyx_XDECREF(__pyx_v_d_cond_ent);
  __Pyx_XGIVEREF(__pyx_r);
  __Pyx_TraceReturn(__pyx_r, 0);
  __Pyx_RefNannyFinishContext();
  return __pyx_r;
}
/* … */
  __pyx_tuple__8 = PyTuple_Pack(30, __pyx_n_s_v_channel, __pyx_n_s_nda_interf, __pyx_n_s_v_dc_bits, __pyx_n_s_v_ga_bits, __pyx_n_s_d_lambda, __pyx_n_s_v_dc_bits_u, __pyx_n_s_v_ga_bits_u, __pyx_n_s_bv_using, __pyx_n_s_n_using, __pyx_n_s_v_channel_u, __pyx_n_s_nda_interf_u, __pyx_n_s_nda_receiver_noise, __pyx_n_s_nda_receiver_signal, __pyx_n_s_v_helper_distortion, __pyx_n_s_nda_total_noise, __pyx_n_s_v_s_n_dets, __pyx_n_s_v_cond_ents, __pyx_n_s_v_badness, __pyx_n_s_n_set, __pyx_n_s_n_set_c, __pyx_n_s_bv_set, __pyx_n_s_d_entropy_include, __pyx_n_s_d_entropy_exclude, __pyx_n_s_n_in_set, __pyx_n_s_n_set_kliii, __pyx_n_s_iii, __pyx_n_s_n_set_iii, __pyx_n_s_n_set_kleqiii, __pyx_n_s_d_mi, __pyx_n_s_d_cond_ent); if (unlikely(!__pyx_tuple__8)) __PYX_ERR(0, 24, __pyx_L1_error)
  __Pyx_GOTREF(__pyx_tuple__8);
  __Pyx_GIVEREF(__pyx_tuple__8);
/* … */
  __pyx_t_1 = PyCFunction_NewEx(&__pyx_mdef_9dist_recv_7checkdc_3checkdc_cts, NULL, __pyx_n_s_dist_recv_checkdc); if (unlikely(!__pyx_t_1)) __PYX_ERR(0, 24, __pyx_L1_error)
  __Pyx_GOTREF(__pyx_t_1);
  if (PyDict_SetItem(__pyx_d, __pyx_n_s_checkdc_cts, __pyx_t_1) < 0) __PYX_ERR(0, 24, __pyx_L1_error)
  __Pyx_DECREF(__pyx_t_1); __pyx_t_1 = 0;
  __pyx_codeobj__2 = (PyObject*)__Pyx_PyCode_New(5, 0, 30, 0, 0, __pyx_empty_bytes, __pyx_empty_tuple, __pyx_empty_tuple, __pyx_tuple__8, __pyx_empty_tuple, __pyx_empty_tuple, __pyx_kp_s_Users_cdchapm2_Dropbox_Personal, __pyx_n_s_checkdc_cts, 24, __pyx_empty_bytes); if (unlikely(!__pyx_codeobj__2)) __PYX_ERR(0, 24, __pyx_L1_error)
```

```
 025:     """ Check whether a distributed compression configuration violates entropy conditions
```

```
 026:         (continuous version)
```

```
 027:          Input:
```

```
 028:              v_channel = Channel vector h (size N+1 x 1)
```

```
 029:              nda_interf = Interference covariance matrix Sigma (size N+1 x N+1)
```

```
 030:              v_dc_bits = Post-compression helper encoding rates. (size N x 1)
```

```
 031:              v_ga_bits = Pre-compression Gaussian encoder rates. (size N x 1)
```

```
 032:              d_lambda = Hull parameter
```

```
 033:          Output:
```

```
 034:              v_badness = violation amounts
```

```
 035:      """
```

```
 036:
```

```
 037:     # Clip to useful receivers
```

```
+038:     v_dc_bits_u = np.append(float('inf'), v_dc_bits)
```

```
  __pyx_t_2 = __Pyx_GetModuleGlobalName(__pyx_n_s_np); if (unlikely(!__pyx_t_2)) __PYX_ERR(0, 38, __pyx_L1_error)
  __Pyx_GOTREF(__pyx_t_2);
  __pyx_t_3 = __Pyx_PyObject_GetAttrStr(__pyx_t_2, __pyx_n_s_append); if (unlikely(!__pyx_t_3)) __PYX_ERR(0, 38, __pyx_L1_error)
  __Pyx_GOTREF(__pyx_t_3);
  __Pyx_DECREF(__pyx_t_2); __pyx_t_2 = 0;
  __pyx_t_2 = __Pyx_PyNumber_Float(__pyx_n_s_inf); if (unlikely(!__pyx_t_2)) __PYX_ERR(0, 38, __pyx_L1_error)
  __Pyx_GOTREF(__pyx_t_2);
  __pyx_t_4 = NULL;
  __pyx_t_5 = 0;
  if (CYTHON_UNPACK_METHODS && unlikely(PyMethod_Check(__pyx_t_3))) {
    __pyx_t_4 = PyMethod_GET_SELF(__pyx_t_3);
    if (likely(__pyx_t_4)) {
      PyObject* function = PyMethod_GET_FUNCTION(__pyx_t_3);
      __Pyx_INCREF(__pyx_t_4);
      __Pyx_INCREF(function);
      __Pyx_DECREF_SET(__pyx_t_3, function);
      __pyx_t_5 = 1;
    }
  }
  #if CYTHON_FAST_PYCALL
  if (PyFunction_Check(__pyx_t_3)) {
    PyObject *__pyx_temp[3] = {__pyx_t_4, __pyx_t_2, __pyx_v_v_dc_bits};
    __pyx_t_1 = __Pyx_PyFunction_FastCall(__pyx_t_3, __pyx_temp+1-__pyx_t_5, 2+__pyx_t_5); if (unlikely(!__pyx_t_1)) __PYX_ERR(0, 38, __pyx_L1_error)
    __Pyx_XDECREF(__pyx_t_4); __pyx_t_4 = 0;
    __Pyx_GOTREF(__pyx_t_1);
    __Pyx_DECREF(__pyx_t_2); __pyx_t_2 = 0;
  } else
  #endif
  #if CYTHON_FAST_PYCCALL
  if (__Pyx_PyFastCFunction_Check(__pyx_t_3)) {
    PyObject *__pyx_temp[3] = {__pyx_t_4, __pyx_t_2, __pyx_v_v_dc_bits};
    __pyx_t_1 = __Pyx_PyCFunction_FastCall(__pyx_t_3, __pyx_temp+1-__pyx_t_5, 2+__pyx_t_5); if (unlikely(!__pyx_t_1)) __PYX_ERR(0, 38, __pyx_L1_error)
    __Pyx_XDECREF(__pyx_t_4); __pyx_t_4 = 0;
    __Pyx_GOTREF(__pyx_t_1);
    __Pyx_DECREF(__pyx_t_2); __pyx_t_2 = 0;
  } else
  #endif
  {
    __pyx_t_6 = PyTuple_New(2+__pyx_t_5); if (unlikely(!__pyx_t_6)) __PYX_ERR(0, 38, __pyx_L1_error)
    __Pyx_GOTREF(__pyx_t_6);
    if (__pyx_t_4) {
      __Pyx_GIVEREF(__pyx_t_4); PyTuple_SET_ITEM(__pyx_t_6, 0, __pyx_t_4); __pyx_t_4 = NULL;
    }
    __Pyx_GIVEREF(__pyx_t_2);
    PyTuple_SET_ITEM(__pyx_t_6, 0+__pyx_t_5, __pyx_t_2);
    __Pyx_INCREF(__pyx_v_v_dc_bits);
    __Pyx_GIVEREF(__pyx_v_v_dc_bits);
    PyTuple_SET_ITEM(__pyx_t_6, 1+__pyx_t_5, __pyx_v_v_dc_bits);
    __pyx_t_2 = 0;
    __pyx_t_1 = __Pyx_PyObject_Call(__pyx_t_3, __pyx_t_6, NULL); if (unlikely(!__pyx_t_1)) __PYX_ERR(0, 38, __pyx_L1_error)
    __Pyx_GOTREF(__pyx_t_1);
    __Pyx_DECREF(__pyx_t_6); __pyx_t_6 = 0;
  }
  __Pyx_DECREF(__pyx_t_3); __pyx_t_3 = 0;
  __pyx_v_v_dc_bits_u = __pyx_t_1;
  __pyx_t_1 = 0;
```

```
+039:     v_ga_bits_u = np.append(float('inf'), v_ga_bits)
```

```
  __pyx_t_3 = __Pyx_GetModuleGlobalName(__pyx_n_s_np); if (unlikely(!__pyx_t_3)) __PYX_ERR(0, 39, __pyx_L1_error)
  __Pyx_GOTREF(__pyx_t_3);
  __pyx_t_6 = __Pyx_PyObject_GetAttrStr(__pyx_t_3, __pyx_n_s_append); if (unlikely(!__pyx_t_6)) __PYX_ERR(0, 39, __pyx_L1_error)
  __Pyx_GOTREF(__pyx_t_6);
  __Pyx_DECREF(__pyx_t_3); __pyx_t_3 = 0;
  __pyx_t_3 = __Pyx_PyNumber_Float(__pyx_n_s_inf); if (unlikely(!__pyx_t_3)) __PYX_ERR(0, 39, __pyx_L1_error)
  __Pyx_GOTREF(__pyx_t_3);
  __pyx_t_2 = NULL;
  __pyx_t_5 = 0;
  if (CYTHON_UNPACK_METHODS && unlikely(PyMethod_Check(__pyx_t_6))) {
    __pyx_t_2 = PyMethod_GET_SELF(__pyx_t_6);
    if (likely(__pyx_t_2)) {
      PyObject* function = PyMethod_GET_FUNCTION(__pyx_t_6);
      __Pyx_INCREF(__pyx_t_2);
      __Pyx_INCREF(function);
      __Pyx_DECREF_SET(__pyx_t_6, function);
      __pyx_t_5 = 1;
    }
  }
  #if CYTHON_FAST_PYCALL
  if (PyFunction_Check(__pyx_t_6)) {
    PyObject *__pyx_temp[3] = {__pyx_t_2, __pyx_t_3, __pyx_v_v_ga_bits};
    __pyx_t_1 = __Pyx_PyFunction_FastCall(__pyx_t_6, __pyx_temp+1-__pyx_t_5, 2+__pyx_t_5); if (unlikely(!__pyx_t_1)) __PYX_ERR(0, 39, __pyx_L1_error)
    __Pyx_XDECREF(__pyx_t_2); __pyx_t_2 = 0;
    __Pyx_GOTREF(__pyx_t_1);
    __Pyx_DECREF(__pyx_t_3); __pyx_t_3 = 0;
  } else
  #endif
  #if CYTHON_FAST_PYCCALL
  if (__Pyx_PyFastCFunction_Check(__pyx_t_6)) {
    PyObject *__pyx_temp[3] = {__pyx_t_2, __pyx_t_3, __pyx_v_v_ga_bits};
    __pyx_t_1 = __Pyx_PyCFunction_FastCall(__pyx_t_6, __pyx_temp+1-__pyx_t_5, 2+__pyx_t_5); if (unlikely(!__pyx_t_1)) __PYX_ERR(0, 39, __pyx_L1_error)
    __Pyx_XDECREF(__pyx_t_2); __pyx_t_2 = 0;
    __Pyx_GOTREF(__pyx_t_1);
    __Pyx_DECREF(__pyx_t_3); __pyx_t_3 = 0;
  } else
  #endif
  {
    __pyx_t_4 = PyTuple_New(2+__pyx_t_5); if (unlikely(!__pyx_t_4)) __PYX_ERR(0, 39, __pyx_L1_error)
    __Pyx_GOTREF(__pyx_t_4);
    if (__pyx_t_2) {
      __Pyx_GIVEREF(__pyx_t_2); PyTuple_SET_ITEM(__pyx_t_4, 0, __pyx_t_2); __pyx_t_2 = NULL;
    }
    __Pyx_GIVEREF(__pyx_t_3);
    PyTuple_SET_ITEM(__pyx_t_4, 0+__pyx_t_5, __pyx_t_3);
    __Pyx_INCREF(__pyx_v_v_ga_bits);
    __Pyx_GIVEREF(__pyx_v_v_ga_bits);
    PyTuple_SET_ITEM(__pyx_t_4, 1+__pyx_t_5, __pyx_v_v_ga_bits);
    __pyx_t_3 = 0;
    __pyx_t_1 = __Pyx_PyObject_Call(__pyx_t_6, __pyx_t_4, NULL); if (unlikely(!__pyx_t_1)) __PYX_ERR(0, 39, __pyx_L1_error)
    __Pyx_GOTREF(__pyx_t_1);
    __Pyx_DECREF(__pyx_t_4); __pyx_t_4 = 0;
  }
  __Pyx_DECREF(__pyx_t_6); __pyx_t_6 = 0;
  __pyx_v_v_ga_bits_u = __pyx_t_1;
  __pyx_t_1 = 0;
```

```
+040:     bv_using = (v_dc_bits_u > D_MIN_USEFUL_BITRATE) & (v_ga_bits_u > D_MIN_USEFUL_BITRATE)
```

```
  __pyx_t_1 = __Pyx_GetModuleGlobalName(__pyx_n_s_D_MIN_USEFUL_BITRATE); if (unlikely(!__pyx_t_1)) __PYX_ERR(0, 40, __pyx_L1_error)
  __Pyx_GOTREF(__pyx_t_1);
  __pyx_t_6 = PyObject_RichCompare(__pyx_v_v_dc_bits_u, __pyx_t_1, Py_GT); __Pyx_XGOTREF(__pyx_t_6); if (unlikely(!__pyx_t_6)) __PYX_ERR(0, 40, __pyx_L1_error)
  __Pyx_DECREF(__pyx_t_1); __pyx_t_1 = 0;
  __pyx_t_1 = __Pyx_GetModuleGlobalName(__pyx_n_s_D_MIN_USEFUL_BITRATE); if (unlikely(!__pyx_t_1)) __PYX_ERR(0, 40, __pyx_L1_error)
  __Pyx_GOTREF(__pyx_t_1);
  __pyx_t_4 = PyObject_RichCompare(__pyx_v_v_ga_bits_u, __pyx_t_1, Py_GT); __Pyx_XGOTREF(__pyx_t_4); if (unlikely(!__pyx_t_4)) __PYX_ERR(0, 40, __pyx_L1_error)
  __Pyx_DECREF(__pyx_t_1); __pyx_t_1 = 0;
  __pyx_t_1 = PyNumber_And(__pyx_t_6, __pyx_t_4); if (unlikely(!__pyx_t_1)) __PYX_ERR(0, 40, __pyx_L1_error)
  __Pyx_GOTREF(__pyx_t_1);
  __Pyx_DECREF(__pyx_t_6); __pyx_t_6 = 0;
  __Pyx_DECREF(__pyx_t_4); __pyx_t_4 = 0;
  __pyx_v_bv_using = __pyx_t_1;
  __pyx_t_1 = 0;
```

```
+041:     n_using = sum(bv_using)
```

```
  __pyx_t_1 = PyTuple_New(1); if (unlikely(!__pyx_t_1)) __PYX_ERR(0, 41, __pyx_L1_error)
  __Pyx_GOTREF(__pyx_t_1);
  __Pyx_INCREF(__pyx_v_bv_using);
  __Pyx_GIVEREF(__pyx_v_bv_using);
  PyTuple_SET_ITEM(__pyx_t_1, 0, __pyx_v_bv_using);
  __pyx_t_4 = __Pyx_PyObject_Call(__pyx_builtin_sum, __pyx_t_1, NULL); if (unlikely(!__pyx_t_4)) __PYX_ERR(0, 41, __pyx_L1_error)
  __Pyx_GOTREF(__pyx_t_4);
  __Pyx_DECREF(__pyx_t_1); __pyx_t_1 = 0;
  __pyx_v_n_using = __pyx_t_4;
  __pyx_t_4 = 0;
```

```
+042:     if n_using == 0:
```

```
  __pyx_t_4 = __Pyx_PyInt_EqObjC(__pyx_v_n_using, __pyx_int_0, 0, 0); if (unlikely(!__pyx_t_4)) __PYX_ERR(0, 42, __pyx_L1_error)
  __Pyx_GOTREF(__pyx_t_4);
  __pyx_t_7 = __Pyx_PyObject_IsTrue(__pyx_t_4); if (unlikely(__pyx_t_7 < 0)) __PYX_ERR(0, 42, __pyx_L1_error)
  __Pyx_DECREF(__pyx_t_4); __pyx_t_4 = 0;
  if (__pyx_t_7) {
/* … */
  }
```

```
+043:         return 0
```

```
    __Pyx_XDECREF(__pyx_r);
    __Pyx_INCREF(__pyx_int_0);
    __pyx_r = __pyx_int_0;
    goto __pyx_L0;
```

```
 044:
```

```
+045:     v_channel_u = v_channel[bv_using]
```

```
  __pyx_t_4 = PyObject_GetItem(__pyx_v_v_channel, __pyx_v_bv_using); if (unlikely(!__pyx_t_4)) __PYX_ERR(0, 45, __pyx_L1_error)
  __Pyx_GOTREF(__pyx_t_4);
  __pyx_v_v_channel_u = __pyx_t_4;
  __pyx_t_4 = 0;
```

```
+046:     nda_interf_u = nda_interf[bv_using][:, bv_using]
```

```
  __pyx_t_4 = PyObject_GetItem(__pyx_v_nda_interf, __pyx_v_bv_using); if (unlikely(!__pyx_t_4)) __PYX_ERR(0, 46, __pyx_L1_error)
  __Pyx_GOTREF(__pyx_t_4);
  __pyx_t_1 = PyTuple_New(2); if (unlikely(!__pyx_t_1)) __PYX_ERR(0, 46, __pyx_L1_error)
  __Pyx_GOTREF(__pyx_t_1);
  __Pyx_INCREF(__pyx_slice__3);
  __Pyx_GIVEREF(__pyx_slice__3);
  PyTuple_SET_ITEM(__pyx_t_1, 0, __pyx_slice__3);
  __Pyx_INCREF(__pyx_v_bv_using);
  __Pyx_GIVEREF(__pyx_v_bv_using);
  PyTuple_SET_ITEM(__pyx_t_1, 1, __pyx_v_bv_using);
  __pyx_t_6 = PyObject_GetItem(__pyx_t_4, __pyx_t_1); if (unlikely(!__pyx_t_6)) __PYX_ERR(0, 46, __pyx_L1_error)
  __Pyx_GOTREF(__pyx_t_6);
  __Pyx_DECREF(__pyx_t_4); __pyx_t_4 = 0;
  __Pyx_DECREF(__pyx_t_1); __pyx_t_1 = 0;
  __pyx_v_nda_interf_u = __pyx_t_6;
  __pyx_t_6 = 0;
/* … */
  __pyx_slice__3 = PySlice_New(Py_None, Py_None, Py_None); if (unlikely(!__pyx_slice__3)) __PYX_ERR(0, 46, __pyx_L1_error)
  __Pyx_GOTREF(__pyx_slice__3);
  __Pyx_GIVEREF(__pyx_slice__3);
```

```
+047:     v_dc_bits_u = v_dc_bits_u[bv_using]
```

```
  __pyx_t_6 = PyObject_GetItem(__pyx_v_v_dc_bits_u, __pyx_v_bv_using); if (unlikely(!__pyx_t_6)) __PYX_ERR(0, 47, __pyx_L1_error)
  __Pyx_GOTREF(__pyx_t_6);
  __Pyx_DECREF_SET(__pyx_v_v_dc_bits_u, __pyx_t_6);
  __pyx_t_6 = 0;
```

```
+048:     v_ga_bits_u = v_ga_bits_u[bv_using]
```

```
  __pyx_t_6 = PyObject_GetItem(__pyx_v_v_ga_bits_u, __pyx_v_bv_using); if (unlikely(!__pyx_t_6)) __PYX_ERR(0, 48, __pyx_L1_error)
  __Pyx_GOTREF(__pyx_t_6);
  __Pyx_DECREF_SET(__pyx_v_v_ga_bits_u, __pyx_t_6);
  __pyx_t_6 = 0;
```

```
 049:
```

```
 050:     # Receiver thermal noise + interference covariance
```

```
+051:     nda_receiver_noise = nda_interf_u + np.identity(n_using)
```

```
  __pyx_t_1 = __Pyx_GetModuleGlobalName(__pyx_n_s_np); if (unlikely(!__pyx_t_1)) __PYX_ERR(0, 51, __pyx_L1_error)
  __Pyx_GOTREF(__pyx_t_1);
  __pyx_t_4 = __Pyx_PyObject_GetAttrStr(__pyx_t_1, __pyx_n_s_identity); if (unlikely(!__pyx_t_4)) __PYX_ERR(0, 51, __pyx_L1_error)
  __Pyx_GOTREF(__pyx_t_4);
  __Pyx_DECREF(__pyx_t_1); __pyx_t_1 = 0;
  __pyx_t_1 = NULL;
  if (CYTHON_UNPACK_METHODS && unlikely(PyMethod_Check(__pyx_t_4))) {
    __pyx_t_1 = PyMethod_GET_SELF(__pyx_t_4);
    if (likely(__pyx_t_1)) {
      PyObject* function = PyMethod_GET_FUNCTION(__pyx_t_4);
      __Pyx_INCREF(__pyx_t_1);
      __Pyx_INCREF(function);
      __Pyx_DECREF_SET(__pyx_t_4, function);
    }
  }
  if (!__pyx_t_1) {
    __pyx_t_6 = __Pyx_PyObject_CallOneArg(__pyx_t_4, __pyx_v_n_using); if (unlikely(!__pyx_t_6)) __PYX_ERR(0, 51, __pyx_L1_error)
    __Pyx_GOTREF(__pyx_t_6);
  } else {
    #if CYTHON_FAST_PYCALL
    if (PyFunction_Check(__pyx_t_4)) {
      PyObject *__pyx_temp[2] = {__pyx_t_1, __pyx_v_n_using};
      __pyx_t_6 = __Pyx_PyFunction_FastCall(__pyx_t_4, __pyx_temp+1-1, 1+1); if (unlikely(!__pyx_t_6)) __PYX_ERR(0, 51, __pyx_L1_error)
      __Pyx_XDECREF(__pyx_t_1); __pyx_t_1 = 0;
      __Pyx_GOTREF(__pyx_t_6);
    } else
    #endif
    #if CYTHON_FAST_PYCCALL
    if (__Pyx_PyFastCFunction_Check(__pyx_t_4)) {
      PyObject *__pyx_temp[2] = {__pyx_t_1, __pyx_v_n_using};
      __pyx_t_6 = __Pyx_PyCFunction_FastCall(__pyx_t_4, __pyx_temp+1-1, 1+1); if (unlikely(!__pyx_t_6)) __PYX_ERR(0, 51, __pyx_L1_error)
      __Pyx_XDECREF(__pyx_t_1); __pyx_t_1 = 0;
      __Pyx_GOTREF(__pyx_t_6);
    } else
    #endif
    {
      __pyx_t_3 = PyTuple_New(1+1); if (unlikely(!__pyx_t_3)) __PYX_ERR(0, 51, __pyx_L1_error)
      __Pyx_GOTREF(__pyx_t_3);
      __Pyx_GIVEREF(__pyx_t_1); PyTuple_SET_ITEM(__pyx_t_3, 0, __pyx_t_1); __pyx_t_1 = NULL;
      __Pyx_INCREF(__pyx_v_n_using);
      __Pyx_GIVEREF(__pyx_v_n_using);
      PyTuple_SET_ITEM(__pyx_t_3, 0+1, __pyx_v_n_using);
      __pyx_t_6 = __Pyx_PyObject_Call(__pyx_t_4, __pyx_t_3, NULL); if (unlikely(!__pyx_t_6)) __PYX_ERR(0, 51, __pyx_L1_error)
      __Pyx_GOTREF(__pyx_t_6);
      __Pyx_DECREF(__pyx_t_3); __pyx_t_3 = 0;
    }
  }
  __Pyx_DECREF(__pyx_t_4); __pyx_t_4 = 0;
  __pyx_t_4 = PyNumber_Add(__pyx_v_nda_interf_u, __pyx_t_6); if (unlikely(!__pyx_t_4)) __PYX_ERR(0, 51, __pyx_L1_error)
  __Pyx_GOTREF(__pyx_t_4);
  __Pyx_DECREF(__pyx_t_6); __pyx_t_6 = 0;
  __pyx_v_nda_receiver_noise = __pyx_t_4;
  __pyx_t_4 = 0;
```

```
 052:
```

```
 053:     # Receive signal covariance
```

```
+054:     nda_receiver_signal = np.outer(v_channel_u, v_channel_u.conj())
```

```
  __pyx_t_6 = __Pyx_GetModuleGlobalName(__pyx_n_s_np); if (unlikely(!__pyx_t_6)) __PYX_ERR(0, 54, __pyx_L1_error)
  __Pyx_GOTREF(__pyx_t_6);
  __pyx_t_3 = __Pyx_PyObject_GetAttrStr(__pyx_t_6, __pyx_n_s_outer); if (unlikely(!__pyx_t_3)) __PYX_ERR(0, 54, __pyx_L1_error)
  __Pyx_GOTREF(__pyx_t_3);
  __Pyx_DECREF(__pyx_t_6); __pyx_t_6 = 0;
  __pyx_t_1 = __Pyx_PyObject_GetAttrStr(__pyx_v_v_channel_u, __pyx_n_s_conj); if (unlikely(!__pyx_t_1)) __PYX_ERR(0, 54, __pyx_L1_error)
  __Pyx_GOTREF(__pyx_t_1);
  __pyx_t_2 = NULL;
  if (CYTHON_UNPACK_METHODS && likely(PyMethod_Check(__pyx_t_1))) {
    __pyx_t_2 = PyMethod_GET_SELF(__pyx_t_1);
    if (likely(__pyx_t_2)) {
      PyObject* function = PyMethod_GET_FUNCTION(__pyx_t_1);
      __Pyx_INCREF(__pyx_t_2);
      __Pyx_INCREF(function);
      __Pyx_DECREF_SET(__pyx_t_1, function);
    }
  }
  if (__pyx_t_2) {
    __pyx_t_6 = __Pyx_PyObject_CallOneArg(__pyx_t_1, __pyx_t_2); if (unlikely(!__pyx_t_6)) __PYX_ERR(0, 54, __pyx_L1_error)
    __Pyx_DECREF(__pyx_t_2); __pyx_t_2 = 0;
  } else {
    __pyx_t_6 = __Pyx_PyObject_CallNoArg(__pyx_t_1); if (unlikely(!__pyx_t_6)) __PYX_ERR(0, 54, __pyx_L1_error)
  }
  __Pyx_GOTREF(__pyx_t_6);
  __Pyx_DECREF(__pyx_t_1); __pyx_t_1 = 0;
  __pyx_t_1 = NULL;
  __pyx_t_5 = 0;
  if (CYTHON_UNPACK_METHODS && unlikely(PyMethod_Check(__pyx_t_3))) {
    __pyx_t_1 = PyMethod_GET_SELF(__pyx_t_3);
    if (likely(__pyx_t_1)) {
      PyObject* function = PyMethod_GET_FUNCTION(__pyx_t_3);
      __Pyx_INCREF(__pyx_t_1);
      __Pyx_INCREF(function);
      __Pyx_DECREF_SET(__pyx_t_3, function);
      __pyx_t_5 = 1;
    }
  }
  #if CYTHON_FAST_PYCALL
  if (PyFunction_Check(__pyx_t_3)) {
    PyObject *__pyx_temp[3] = {__pyx_t_1, __pyx_v_v_channel_u, __pyx_t_6};
    __pyx_t_4 = __Pyx_PyFunction_FastCall(__pyx_t_3, __pyx_temp+1-__pyx_t_5, 2+__pyx_t_5); if (unlikely(!__pyx_t_4)) __PYX_ERR(0, 54, __pyx_L1_error)
    __Pyx_XDECREF(__pyx_t_1); __pyx_t_1 = 0;
    __Pyx_GOTREF(__pyx_t_4);
    __Pyx_DECREF(__pyx_t_6); __pyx_t_6 = 0;
  } else
  #endif
  #if CYTHON_FAST_PYCCALL
  if (__Pyx_PyFastCFunction_Check(__pyx_t_3)) {
    PyObject *__pyx_temp[3] = {__pyx_t_1, __pyx_v_v_channel_u, __pyx_t_6};
    __pyx_t_4 = __Pyx_PyCFunction_FastCall(__pyx_t_3, __pyx_temp+1-__pyx_t_5, 2+__pyx_t_5); if (unlikely(!__pyx_t_4)) __PYX_ERR(0, 54, __pyx_L1_error)
    __Pyx_XDECREF(__pyx_t_1); __pyx_t_1 = 0;
    __Pyx_GOTREF(__pyx_t_4);
    __Pyx_DECREF(__pyx_t_6); __pyx_t_6 = 0;
  } else
  #endif
  {
    __pyx_t_2 = PyTuple_New(2+__pyx_t_5); if (unlikely(!__pyx_t_2)) __PYX_ERR(0, 54, __pyx_L1_error)
    __Pyx_GOTREF(__pyx_t_2);
    if (__pyx_t_1) {
      __Pyx_GIVEREF(__pyx_t_1); PyTuple_SET_ITEM(__pyx_t_2, 0, __pyx_t_1); __pyx_t_1 = NULL;
    }
    __Pyx_INCREF(__pyx_v_v_channel_u);
    __Pyx_GIVEREF(__pyx_v_v_channel_u);
    PyTuple_SET_ITEM(__pyx_t_2, 0+__pyx_t_5, __pyx_v_v_channel_u);
    __Pyx_GIVEREF(__pyx_t_6);
    PyTuple_SET_ITEM(__pyx_t_2, 1+__pyx_t_5, __pyx_t_6);
    __pyx_t_6 = 0;
    __pyx_t_4 = __Pyx_PyObject_Call(__pyx_t_3, __pyx_t_2, NULL); if (unlikely(!__pyx_t_4)) __PYX_ERR(0, 54, __pyx_L1_error)
    __Pyx_GOTREF(__pyx_t_4);
    __Pyx_DECREF(__pyx_t_2); __pyx_t_2 = 0;
  }
  __Pyx_DECREF(__pyx_t_3); __pyx_t_3 = 0;
  __pyx_v_nda_receiver_signal = __pyx_t_4;
  __pyx_t_4 = 0;
```

```
 055:
```

```
 056:     # Power of distortion due to quantization
```

```
+057:     v_helper_distortion = np.real(np.diag(nda_receiver_signal + nda_receiver_noise))
```

```
  __pyx_t_3 = __Pyx_GetModuleGlobalName(__pyx_n_s_np); if (unlikely(!__pyx_t_3)) __PYX_ERR(0, 57, __pyx_L1_error)
  __Pyx_GOTREF(__pyx_t_3);
  __pyx_t_2 = __Pyx_PyObject_GetAttrStr(__pyx_t_3, __pyx_n_s_real); if (unlikely(!__pyx_t_2)) __PYX_ERR(0, 57, __pyx_L1_error)
  __Pyx_GOTREF(__pyx_t_2);
  __Pyx_DECREF(__pyx_t_3); __pyx_t_3 = 0;
  __pyx_t_6 = __Pyx_GetModuleGlobalName(__pyx_n_s_np); if (unlikely(!__pyx_t_6)) __PYX_ERR(0, 57, __pyx_L1_error)
  __Pyx_GOTREF(__pyx_t_6);
  __pyx_t_1 = __Pyx_PyObject_GetAttrStr(__pyx_t_6, __pyx_n_s_diag); if (unlikely(!__pyx_t_1)) __PYX_ERR(0, 57, __pyx_L1_error)
  __Pyx_GOTREF(__pyx_t_1);
  __Pyx_DECREF(__pyx_t_6); __pyx_t_6 = 0;
  __pyx_t_6 = PyNumber_Add(__pyx_v_nda_receiver_signal, __pyx_v_nda_receiver_noise); if (unlikely(!__pyx_t_6)) __PYX_ERR(0, 57, __pyx_L1_error)
  __Pyx_GOTREF(__pyx_t_6);
  __pyx_t_8 = NULL;
  if (CYTHON_UNPACK_METHODS && unlikely(PyMethod_Check(__pyx_t_1))) {
    __pyx_t_8 = PyMethod_GET_SELF(__pyx_t_1);
    if (likely(__pyx_t_8)) {
      PyObject* function = PyMethod_GET_FUNCTION(__pyx_t_1);
      __Pyx_INCREF(__pyx_t_8);
      __Pyx_INCREF(function);
      __Pyx_DECREF_SET(__pyx_t_1, function);
    }
  }
  if (!__pyx_t_8) {
    __pyx_t_3 = __Pyx_PyObject_CallOneArg(__pyx_t_1, __pyx_t_6); if (unlikely(!__pyx_t_3)) __PYX_ERR(0, 57, __pyx_L1_error)
    __Pyx_DECREF(__pyx_t_6); __pyx_t_6 = 0;
    __Pyx_GOTREF(__pyx_t_3);
  } else {
    #if CYTHON_FAST_PYCALL
    if (PyFunction_Check(__pyx_t_1)) {
      PyObject *__pyx_temp[2] = {__pyx_t_8, __pyx_t_6};
      __pyx_t_3 = __Pyx_PyFunction_FastCall(__pyx_t_1, __pyx_temp+1-1, 1+1); if (unlikely(!__pyx_t_3)) __PYX_ERR(0, 57, __pyx_L1_error)
      __Pyx_XDECREF(__pyx_t_8); __pyx_t_8 = 0;
      __Pyx_GOTREF(__pyx_t_3);
      __Pyx_DECREF(__pyx_t_6); __pyx_t_6 = 0;
    } else
    #endif
    #if CYTHON_FAST_PYCCALL
    if (__Pyx_PyFastCFunction_Check(__pyx_t_1)) {
      PyObject *__pyx_temp[2] = {__pyx_t_8, __pyx_t_6};
      __pyx_t_3 = __Pyx_PyCFunction_FastCall(__pyx_t_1, __pyx_temp+1-1, 1+1); if (unlikely(!__pyx_t_3)) __PYX_ERR(0, 57, __pyx_L1_error)
      __Pyx_XDECREF(__pyx_t_8); __pyx_t_8 = 0;
      __Pyx_GOTREF(__pyx_t_3);
      __Pyx_DECREF(__pyx_t_6); __pyx_t_6 = 0;
    } else
    #endif
    {
      __pyx_t_9 = PyTuple_New(1+1); if (unlikely(!__pyx_t_9)) __PYX_ERR(0, 57, __pyx_L1_error)
      __Pyx_GOTREF(__pyx_t_9);
      __Pyx_GIVEREF(__pyx_t_8); PyTuple_SET_ITEM(__pyx_t_9, 0, __pyx_t_8); __pyx_t_8 = NULL;
      __Pyx_GIVEREF(__pyx_t_6);
      PyTuple_SET_ITEM(__pyx_t_9, 0+1, __pyx_t_6);
      __pyx_t_6 = 0;
      __pyx_t_3 = __Pyx_PyObject_Call(__pyx_t_1, __pyx_t_9, NULL); if (unlikely(!__pyx_t_3)) __PYX_ERR(0, 57, __pyx_L1_error)
      __Pyx_GOTREF(__pyx_t_3);
      __Pyx_DECREF(__pyx_t_9); __pyx_t_9 = 0;
    }
  }
  __Pyx_DECREF(__pyx_t_1); __pyx_t_1 = 0;
  __pyx_t_1 = NULL;
  if (CYTHON_UNPACK_METHODS && unlikely(PyMethod_Check(__pyx_t_2))) {
    __pyx_t_1 = PyMethod_GET_SELF(__pyx_t_2);
    if (likely(__pyx_t_1)) {
      PyObject* function = PyMethod_GET_FUNCTION(__pyx_t_2);
      __Pyx_INCREF(__pyx_t_1);
      __Pyx_INCREF(function);
      __Pyx_DECREF_SET(__pyx_t_2, function);
    }
  }
  if (!__pyx_t_1) {
    __pyx_t_4 = __Pyx_PyObject_CallOneArg(__pyx_t_2, __pyx_t_3); if (unlikely(!__pyx_t_4)) __PYX_ERR(0, 57, __pyx_L1_error)
    __Pyx_DECREF(__pyx_t_3); __pyx_t_3 = 0;
    __Pyx_GOTREF(__pyx_t_4);
  } else {
    #if CYTHON_FAST_PYCALL
    if (PyFunction_Check(__pyx_t_2)) {
      PyObject *__pyx_temp[2] = {__pyx_t_1, __pyx_t_3};
      __pyx_t_4 = __Pyx_PyFunction_FastCall(__pyx_t_2, __pyx_temp+1-1, 1+1); if (unlikely(!__pyx_t_4)) __PYX_ERR(0, 57, __pyx_L1_error)
      __Pyx_XDECREF(__pyx_t_1); __pyx_t_1 = 0;
      __Pyx_GOTREF(__pyx_t_4);
      __Pyx_DECREF(__pyx_t_3); __pyx_t_3 = 0;
    } else
    #endif
    #if CYTHON_FAST_PYCCALL
    if (__Pyx_PyFastCFunction_Check(__pyx_t_2)) {
      PyObject *__pyx_temp[2] = {__pyx_t_1, __pyx_t_3};
      __pyx_t_4 = __Pyx_PyCFunction_FastCall(__pyx_t_2, __pyx_temp+1-1, 1+1); if (unlikely(!__pyx_t_4)) __PYX_ERR(0, 57, __pyx_L1_error)
      __Pyx_XDECREF(__pyx_t_1); __pyx_t_1 = 0;
      __Pyx_GOTREF(__pyx_t_4);
      __Pyx_DECREF(__pyx_t_3); __pyx_t_3 = 0;
    } else
    #endif
    {
      __pyx_t_9 = PyTuple_New(1+1); if (unlikely(!__pyx_t_9)) __PYX_ERR(0, 57, __pyx_L1_error)
      __Pyx_GOTREF(__pyx_t_9);
      __Pyx_GIVEREF(__pyx_t_1); PyTuple_SET_ITEM(__pyx_t_9, 0, __pyx_t_1); __pyx_t_1 = NULL;
      __Pyx_GIVEREF(__pyx_t_3);
      PyTuple_SET_ITEM(__pyx_t_9, 0+1, __pyx_t_3);
      __pyx_t_3 = 0;
      __pyx_t_4 = __Pyx_PyObject_Call(__pyx_t_2, __pyx_t_9, NULL); if (unlikely(!__pyx_t_4)) __PYX_ERR(0, 57, __pyx_L1_error)
      __Pyx_GOTREF(__pyx_t_4);
      __Pyx_DECREF(__pyx_t_9); __pyx_t_9 = 0;
    }
  }
  __Pyx_DECREF(__pyx_t_2); __pyx_t_2 = 0;
  __pyx_v_v_helper_distortion = __pyx_t_4;
  __pyx_t_4 = 0;
```

```
+058:     v_helper_distortion = np.divide(v_helper_distortion, np.power(2, v_ga_bits_u)-1)
```

```
  __pyx_t_2 = __Pyx_GetModuleGlobalName(__pyx_n_s_np); if (unlikely(!__pyx_t_2)) __PYX_ERR(0, 58, __pyx_L1_error)
  __Pyx_GOTREF(__pyx_t_2);
  __pyx_t_9 = __Pyx_PyObject_GetAttrStr(__pyx_t_2, __pyx_n_s_divide); if (unlikely(!__pyx_t_9)) __PYX_ERR(0, 58, __pyx_L1_error)
  __Pyx_GOTREF(__pyx_t_9);
  __Pyx_DECREF(__pyx_t_2); __pyx_t_2 = 0;
  __pyx_t_3 = __Pyx_GetModuleGlobalName(__pyx_n_s_np); if (unlikely(!__pyx_t_3)) __PYX_ERR(0, 58, __pyx_L1_error)
  __Pyx_GOTREF(__pyx_t_3);
  __pyx_t_1 = __Pyx_PyObject_GetAttrStr(__pyx_t_3, __pyx_n_s_power); if (unlikely(!__pyx_t_1)) __PYX_ERR(0, 58, __pyx_L1_error)
  __Pyx_GOTREF(__pyx_t_1);
  __Pyx_DECREF(__pyx_t_3); __pyx_t_3 = 0;
  __pyx_t_3 = NULL;
  __pyx_t_5 = 0;
  if (CYTHON_UNPACK_METHODS && unlikely(PyMethod_Check(__pyx_t_1))) {
    __pyx_t_3 = PyMethod_GET_SELF(__pyx_t_1);
    if (likely(__pyx_t_3)) {
      PyObject* function = PyMethod_GET_FUNCTION(__pyx_t_1);
      __Pyx_INCREF(__pyx_t_3);
      __Pyx_INCREF(function);
      __Pyx_DECREF_SET(__pyx_t_1, function);
      __pyx_t_5 = 1;
    }
  }
  #if CYTHON_FAST_PYCALL
  if (PyFunction_Check(__pyx_t_1)) {
    PyObject *__pyx_temp[3] = {__pyx_t_3, __pyx_int_2, __pyx_v_v_ga_bits_u};
    __pyx_t_2 = __Pyx_PyFunction_FastCall(__pyx_t_1, __pyx_temp+1-__pyx_t_5, 2+__pyx_t_5); if (unlikely(!__pyx_t_2)) __PYX_ERR(0, 58, __pyx_L1_error)
    __Pyx_XDECREF(__pyx_t_3); __pyx_t_3 = 0;
    __Pyx_GOTREF(__pyx_t_2);
  } else
  #endif
  #if CYTHON_FAST_PYCCALL
  if (__Pyx_PyFastCFunction_Check(__pyx_t_1)) {
    PyObject *__pyx_temp[3] = {__pyx_t_3, __pyx_int_2, __pyx_v_v_ga_bits_u};
    __pyx_t_2 = __Pyx_PyCFunction_FastCall(__pyx_t_1, __pyx_temp+1-__pyx_t_5, 2+__pyx_t_5); if (unlikely(!__pyx_t_2)) __PYX_ERR(0, 58, __pyx_L1_error)
    __Pyx_XDECREF(__pyx_t_3); __pyx_t_3 = 0;
    __Pyx_GOTREF(__pyx_t_2);
  } else
  #endif
  {
    __pyx_t_6 = PyTuple_New(2+__pyx_t_5); if (unlikely(!__pyx_t_6)) __PYX_ERR(0, 58, __pyx_L1_error)
    __Pyx_GOTREF(__pyx_t_6);
    if (__pyx_t_3) {
      __Pyx_GIVEREF(__pyx_t_3); PyTuple_SET_ITEM(__pyx_t_6, 0, __pyx_t_3); __pyx_t_3 = NULL;
    }
    __Pyx_INCREF(__pyx_int_2);
    __Pyx_GIVEREF(__pyx_int_2);
    PyTuple_SET_ITEM(__pyx_t_6, 0+__pyx_t_5, __pyx_int_2);
    __Pyx_INCREF(__pyx_v_v_ga_bits_u);
    __Pyx_GIVEREF(__pyx_v_v_ga_bits_u);
    PyTuple_SET_ITEM(__pyx_t_6, 1+__pyx_t_5, __pyx_v_v_ga_bits_u);
    __pyx_t_2 = __Pyx_PyObject_Call(__pyx_t_1, __pyx_t_6, NULL); if (unlikely(!__pyx_t_2)) __PYX_ERR(0, 58, __pyx_L1_error)
    __Pyx_GOTREF(__pyx_t_2);
    __Pyx_DECREF(__pyx_t_6); __pyx_t_6 = 0;
  }
  __Pyx_DECREF(__pyx_t_1); __pyx_t_1 = 0;
  __pyx_t_1 = __Pyx_PyInt_SubtractObjC(__pyx_t_2, __pyx_int_1, 1, 0); if (unlikely(!__pyx_t_1)) __PYX_ERR(0, 58, __pyx_L1_error)
  __Pyx_GOTREF(__pyx_t_1);
  __Pyx_DECREF(__pyx_t_2); __pyx_t_2 = 0;
  __pyx_t_2 = NULL;
  __pyx_t_5 = 0;
  if (CYTHON_UNPACK_METHODS && unlikely(PyMethod_Check(__pyx_t_9))) {
    __pyx_t_2 = PyMethod_GET_SELF(__pyx_t_9);
    if (likely(__pyx_t_2)) {
      PyObject* function = PyMethod_GET_FUNCTION(__pyx_t_9);
      __Pyx_INCREF(__pyx_t_2);
      __Pyx_INCREF(function);
      __Pyx_DECREF_SET(__pyx_t_9, function);
      __pyx_t_5 = 1;
    }
  }
  #if CYTHON_FAST_PYCALL
  if (PyFunction_Check(__pyx_t_9)) {
    PyObject *__pyx_temp[3] = {__pyx_t_2, __pyx_v_v_helper_distortion, __pyx_t_1};
    __pyx_t_4 = __Pyx_PyFunction_FastCall(__pyx_t_9, __pyx_temp+1-__pyx_t_5, 2+__pyx_t_5); if (unlikely(!__pyx_t_4)) __PYX_ERR(0, 58, __pyx_L1_error)
    __Pyx_XDECREF(__pyx_t_2); __pyx_t_2 = 0;
    __Pyx_GOTREF(__pyx_t_4);
    __Pyx_DECREF(__pyx_t_1); __pyx_t_1 = 0;
  } else
  #endif
  #if CYTHON_FAST_PYCCALL
  if (__Pyx_PyFastCFunction_Check(__pyx_t_9)) {
    PyObject *__pyx_temp[3] = {__pyx_t_2, __pyx_v_v_helper_distortion, __pyx_t_1};
    __pyx_t_4 = __Pyx_PyCFunction_FastCall(__pyx_t_9, __pyx_temp+1-__pyx_t_5, 2+__pyx_t_5); if (unlikely(!__pyx_t_4)) __PYX_ERR(0, 58, __pyx_L1_error)
    __Pyx_XDECREF(__pyx_t_2); __pyx_t_2 = 0;
    __Pyx_GOTREF(__pyx_t_4);
    __Pyx_DECREF(__pyx_t_1); __pyx_t_1 = 0;
  } else
  #endif
  {
    __pyx_t_6 = PyTuple_New(2+__pyx_t_5); if (unlikely(!__pyx_t_6)) __PYX_ERR(0, 58, __pyx_L1_error)
    __Pyx_GOTREF(__pyx_t_6);
    if (__pyx_t_2) {
      __Pyx_GIVEREF(__pyx_t_2); PyTuple_SET_ITEM(__pyx_t_6, 0, __pyx_t_2); __pyx_t_2 = NULL;
    }
    __Pyx_INCREF(__pyx_v_v_helper_distortion);
    __Pyx_GIVEREF(__pyx_v_v_helper_distortion);
    PyTuple_SET_ITEM(__pyx_t_6, 0+__pyx_t_5, __pyx_v_v_helper_distortion);
    __Pyx_GIVEREF(__pyx_t_1);
    PyTuple_SET_ITEM(__pyx_t_6, 1+__pyx_t_5, __pyx_t_1);
    __pyx_t_1 = 0;
    __pyx_t_4 = __Pyx_PyObject_Call(__pyx_t_9, __pyx_t_6, NULL); if (unlikely(!__pyx_t_4)) __PYX_ERR(0, 58, __pyx_L1_error)
    __Pyx_GOTREF(__pyx_t_4);
    __Pyx_DECREF(__pyx_t_6); __pyx_t_6 = 0;
  }
  __Pyx_DECREF(__pyx_t_9); __pyx_t_9 = 0;
  __Pyx_DECREF_SET(__pyx_v_v_helper_distortion, __pyx_t_4);
  __pyx_t_4 = 0;
```

```
 059:
```

```
 060:     # Thermal + interference + distortion covariance
```

```
+061:     nda_total_noise = nda_receiver_noise + np.diag(v_helper_distortion)
```

```
  __pyx_t_9 = __Pyx_GetModuleGlobalName(__pyx_n_s_np); if (unlikely(!__pyx_t_9)) __PYX_ERR(0, 61, __pyx_L1_error)
  __Pyx_GOTREF(__pyx_t_9);
  __pyx_t_6 = __Pyx_PyObject_GetAttrStr(__pyx_t_9, __pyx_n_s_diag); if (unlikely(!__pyx_t_6)) __PYX_ERR(0, 61, __pyx_L1_error)
  __Pyx_GOTREF(__pyx_t_6);
  __Pyx_DECREF(__pyx_t_9); __pyx_t_9 = 0;
  __pyx_t_9 = NULL;
  if (CYTHON_UNPACK_METHODS && unlikely(PyMethod_Check(__pyx_t_6))) {
    __pyx_t_9 = PyMethod_GET_SELF(__pyx_t_6);
    if (likely(__pyx_t_9)) {
      PyObject* function = PyMethod_GET_FUNCTION(__pyx_t_6);
      __Pyx_INCREF(__pyx_t_9);
      __Pyx_INCREF(function);
      __Pyx_DECREF_SET(__pyx_t_6, function);
    }
  }
  if (!__pyx_t_9) {
    __pyx_t_4 = __Pyx_PyObject_CallOneArg(__pyx_t_6, __pyx_v_v_helper_distortion); if (unlikely(!__pyx_t_4)) __PYX_ERR(0, 61, __pyx_L1_error)
    __Pyx_GOTREF(__pyx_t_4);
  } else {
    #if CYTHON_FAST_PYCALL
    if (PyFunction_Check(__pyx_t_6)) {
      PyObject *__pyx_temp[2] = {__pyx_t_9, __pyx_v_v_helper_distortion};
      __pyx_t_4 = __Pyx_PyFunction_FastCall(__pyx_t_6, __pyx_temp+1-1, 1+1); if (unlikely(!__pyx_t_4)) __PYX_ERR(0, 61, __pyx_L1_error)
      __Pyx_XDECREF(__pyx_t_9); __pyx_t_9 = 0;
      __Pyx_GOTREF(__pyx_t_4);
    } else
    #endif
    #if CYTHON_FAST_PYCCALL
    if (__Pyx_PyFastCFunction_Check(__pyx_t_6)) {
      PyObject *__pyx_temp[2] = {__pyx_t_9, __pyx_v_v_helper_distortion};
      __pyx_t_4 = __Pyx_PyCFunction_FastCall(__pyx_t_6, __pyx_temp+1-1, 1+1); if (unlikely(!__pyx_t_4)) __PYX_ERR(0, 61, __pyx_L1_error)
      __Pyx_XDECREF(__pyx_t_9); __pyx_t_9 = 0;
      __Pyx_GOTREF(__pyx_t_4);
    } else
    #endif
    {
      __pyx_t_1 = PyTuple_New(1+1); if (unlikely(!__pyx_t_1)) __PYX_ERR(0, 61, __pyx_L1_error)
      __Pyx_GOTREF(__pyx_t_1);
      __Pyx_GIVEREF(__pyx_t_9); PyTuple_SET_ITEM(__pyx_t_1, 0, __pyx_t_9); __pyx_t_9 = NULL;
      __Pyx_INCREF(__pyx_v_v_helper_distortion);
      __Pyx_GIVEREF(__pyx_v_v_helper_distortion);
      PyTuple_SET_ITEM(__pyx_t_1, 0+1, __pyx_v_v_helper_distortion);
      __pyx_t_4 = __Pyx_PyObject_Call(__pyx_t_6, __pyx_t_1, NULL); if (unlikely(!__pyx_t_4)) __PYX_ERR(0, 61, __pyx_L1_error)
      __Pyx_GOTREF(__pyx_t_4);
      __Pyx_DECREF(__pyx_t_1); __pyx_t_1 = 0;
    }
  }
  __Pyx_DECREF(__pyx_t_6); __pyx_t_6 = 0;
  __pyx_t_6 = PyNumber_Add(__pyx_v_nda_receiver_noise, __pyx_t_4); if (unlikely(!__pyx_t_6)) __PYX_ERR(0, 61, __pyx_L1_error)
  __Pyx_GOTREF(__pyx_t_6);
  __Pyx_DECREF(__pyx_t_4); __pyx_t_4 = 0;
  __pyx_v_nda_total_noise = __pyx_t_6;
  __pyx_t_6 = 0;
```

```
 062:
```

```
 063:     """
```

```
 064:     We want to compute H(Z_S|Z_{S^C}).
```

```
 065:     ...but we only know:
```

```
 066:      * H(Z_n) = v_ga_bits_u[n]
```

```
 067:      * I(Z_S ; Z_{S^C}) = log2(|cov(Z_S)|*|cov(Z_{S^C})| / |cov(Z)|)
```

```
 068:
```

```
 069:     Strategy:
```

```
 070:     H(Z_S|Z_{S^C}) = H(Z_S) - I(Z_S;Z_{S^C})
```

```
 071:     and:
```

```
 072:     H(Z_S) = sum_{n in S} H(Z_n)
```

```
 073:              - sum_{all n} I(Z_sn ; Z_{sk, k<n})
```

```
 074:     """
```

```
 075:
```

```
+076:     v_s_n_dets = det_minors(nda_receiver_signal + nda_total_noise)
```

```
  __pyx_t_4 = __Pyx_GetModuleGlobalName(__pyx_n_s_det_minors); if (unlikely(!__pyx_t_4)) __PYX_ERR(0, 76, __pyx_L1_error)
  __Pyx_GOTREF(__pyx_t_4);
  __pyx_t_1 = PyNumber_Add(__pyx_v_nda_receiver_signal, __pyx_v_nda_total_noise); if (unlikely(!__pyx_t_1)) __PYX_ERR(0, 76, __pyx_L1_error)
  __Pyx_GOTREF(__pyx_t_1);
  __pyx_t_9 = NULL;
  if (CYTHON_UNPACK_METHODS && unlikely(PyMethod_Check(__pyx_t_4))) {
    __pyx_t_9 = PyMethod_GET_SELF(__pyx_t_4);
    if (likely(__pyx_t_9)) {
      PyObject* function = PyMethod_GET_FUNCTION(__pyx_t_4);
      __Pyx_INCREF(__pyx_t_9);
      __Pyx_INCREF(function);
      __Pyx_DECREF_SET(__pyx_t_4, function);
    }
  }
  if (!__pyx_t_9) {
    __pyx_t_6 = __Pyx_PyObject_CallOneArg(__pyx_t_4, __pyx_t_1); if (unlikely(!__pyx_t_6)) __PYX_ERR(0, 76, __pyx_L1_error)
    __Pyx_DECREF(__pyx_t_1); __pyx_t_1 = 0;
    __Pyx_GOTREF(__pyx_t_6);
  } else {
    #if CYTHON_FAST_PYCALL
    if (PyFunction_Check(__pyx_t_4)) {
      PyObject *__pyx_temp[2] = {__pyx_t_9, __pyx_t_1};
      __pyx_t_6 = __Pyx_PyFunction_FastCall(__pyx_t_4, __pyx_temp+1-1, 1+1); if (unlikely(!__pyx_t_6)) __PYX_ERR(0, 76, __pyx_L1_error)
      __Pyx_XDECREF(__pyx_t_9); __pyx_t_9 = 0;
      __Pyx_GOTREF(__pyx_t_6);
      __Pyx_DECREF(__pyx_t_1); __pyx_t_1 = 0;
    } else
    #endif
    #if CYTHON_FAST_PYCCALL
    if (__Pyx_PyFastCFunction_Check(__pyx_t_4)) {
      PyObject *__pyx_temp[2] = {__pyx_t_9, __pyx_t_1};
      __pyx_t_6 = __Pyx_PyCFunction_FastCall(__pyx_t_4, __pyx_temp+1-1, 1+1); if (unlikely(!__pyx_t_6)) __PYX_ERR(0, 76, __pyx_L1_error)
      __Pyx_XDECREF(__pyx_t_9); __pyx_t_9 = 0;
      __Pyx_GOTREF(__pyx_t_6);
      __Pyx_DECREF(__pyx_t_1); __pyx_t_1 = 0;
    } else
    #endif
    {
      __pyx_t_2 = PyTuple_New(1+1); if (unlikely(!__pyx_t_2)) __PYX_ERR(0, 76, __pyx_L1_error)
      __Pyx_GOTREF(__pyx_t_2);
      __Pyx_GIVEREF(__pyx_t_9); PyTuple_SET_ITEM(__pyx_t_2, 0, __pyx_t_9); __pyx_t_9 = NULL;
      __Pyx_GIVEREF(__pyx_t_1);
      PyTuple_SET_ITEM(__pyx_t_2, 0+1, __pyx_t_1);
      __pyx_t_1 = 0;
      __pyx_t_6 = __Pyx_PyObject_Call(__pyx_t_4, __pyx_t_2, NULL); if (unlikely(!__pyx_t_6)) __PYX_ERR(0, 76, __pyx_L1_error)
      __Pyx_GOTREF(__pyx_t_6);
      __Pyx_DECREF(__pyx_t_2); __pyx_t_2 = 0;
    }
  }
  __Pyx_DECREF(__pyx_t_4); __pyx_t_4 = 0;
  __pyx_v_v_s_n_dets = __pyx_t_6;
  __pyx_t_6 = 0;
```

```
+077:     v_cond_ents = np.zeros(2**n_using, dtype=np.complex64)
```

```
  __pyx_t_6 = __Pyx_GetModuleGlobalName(__pyx_n_s_np); if (unlikely(!__pyx_t_6)) __PYX_ERR(0, 77, __pyx_L1_error)
  __Pyx_GOTREF(__pyx_t_6);
  __pyx_t_4 = __Pyx_PyObject_GetAttrStr(__pyx_t_6, __pyx_n_s_zeros); if (unlikely(!__pyx_t_4)) __PYX_ERR(0, 77, __pyx_L1_error)
  __Pyx_GOTREF(__pyx_t_4);
  __Pyx_DECREF(__pyx_t_6); __pyx_t_6 = 0;
  __pyx_t_6 = __Pyx_PyNumber_PowerOf2(__pyx_int_2, __pyx_v_n_using, Py_None); if (unlikely(!__pyx_t_6)) __PYX_ERR(0, 77, __pyx_L1_error)
  __Pyx_GOTREF(__pyx_t_6);
  __pyx_t_2 = PyTuple_New(1); if (unlikely(!__pyx_t_2)) __PYX_ERR(0, 77, __pyx_L1_error)
  __Pyx_GOTREF(__pyx_t_2);
  __Pyx_GIVEREF(__pyx_t_6);
  PyTuple_SET_ITEM(__pyx_t_2, 0, __pyx_t_6);
  __pyx_t_6 = 0;
  __pyx_t_6 = PyDict_New(); if (unlikely(!__pyx_t_6)) __PYX_ERR(0, 77, __pyx_L1_error)
  __Pyx_GOTREF(__pyx_t_6);
  __pyx_t_1 = __Pyx_GetModuleGlobalName(__pyx_n_s_np); if (unlikely(!__pyx_t_1)) __PYX_ERR(0, 77, __pyx_L1_error)
  __Pyx_GOTREF(__pyx_t_1);
  __pyx_t_9 = __Pyx_PyObject_GetAttrStr(__pyx_t_1, __pyx_n_s_complex64); if (unlikely(!__pyx_t_9)) __PYX_ERR(0, 77, __pyx_L1_error)
  __Pyx_GOTREF(__pyx_t_9);
  __Pyx_DECREF(__pyx_t_1); __pyx_t_1 = 0;
  if (PyDict_SetItem(__pyx_t_6, __pyx_n_s_dtype, __pyx_t_9) < 0) __PYX_ERR(0, 77, __pyx_L1_error)
  __Pyx_DECREF(__pyx_t_9); __pyx_t_9 = 0;
  __pyx_t_9 = __Pyx_PyObject_Call(__pyx_t_4, __pyx_t_2, __pyx_t_6); if (unlikely(!__pyx_t_9)) __PYX_ERR(0, 77, __pyx_L1_error)
  __Pyx_GOTREF(__pyx_t_9);
  __Pyx_DECREF(__pyx_t_4); __pyx_t_4 = 0;
  __Pyx_DECREF(__pyx_t_2); __pyx_t_2 = 0;
  __Pyx_DECREF(__pyx_t_6); __pyx_t_6 = 0;
  __pyx_v_v_cond_ents = __pyx_t_9;
  __pyx_t_9 = 0;
```

```
 078:
```

```
+079:     v_badness = np.zeros(2**n_using, dtype=np.complex64)
```

```
  __pyx_t_9 = __Pyx_GetModuleGlobalName(__pyx_n_s_np); if (unlikely(!__pyx_t_9)) __PYX_ERR(0, 79, __pyx_L1_error)
  __Pyx_GOTREF(__pyx_t_9);
  __pyx_t_6 = __Pyx_PyObject_GetAttrStr(__pyx_t_9, __pyx_n_s_zeros); if (unlikely(!__pyx_t_6)) __PYX_ERR(0, 79, __pyx_L1_error)
  __Pyx_GOTREF(__pyx_t_6);
  __Pyx_DECREF(__pyx_t_9); __pyx_t_9 = 0;
  __pyx_t_9 = __Pyx_PyNumber_PowerOf2(__pyx_int_2, __pyx_v_n_using, Py_None); if (unlikely(!__pyx_t_9)) __PYX_ERR(0, 79, __pyx_L1_error)
  __Pyx_GOTREF(__pyx_t_9);
  __pyx_t_2 = PyTuple_New(1); if (unlikely(!__pyx_t_2)) __PYX_ERR(0, 79, __pyx_L1_error)
  __Pyx_GOTREF(__pyx_t_2);
  __Pyx_GIVEREF(__pyx_t_9);
  PyTuple_SET_ITEM(__pyx_t_2, 0, __pyx_t_9);
  __pyx_t_9 = 0;
  __pyx_t_9 = PyDict_New(); if (unlikely(!__pyx_t_9)) __PYX_ERR(0, 79, __pyx_L1_error)
  __Pyx_GOTREF(__pyx_t_9);
  __pyx_t_4 = __Pyx_GetModuleGlobalName(__pyx_n_s_np); if (unlikely(!__pyx_t_4)) __PYX_ERR(0, 79, __pyx_L1_error)
  __Pyx_GOTREF(__pyx_t_4);
  __pyx_t_1 = __Pyx_PyObject_GetAttrStr(__pyx_t_4, __pyx_n_s_complex64); if (unlikely(!__pyx_t_1)) __PYX_ERR(0, 79, __pyx_L1_error)
  __Pyx_GOTREF(__pyx_t_1);
  __Pyx_DECREF(__pyx_t_4); __pyx_t_4 = 0;
  if (PyDict_SetItem(__pyx_t_9, __pyx_n_s_dtype, __pyx_t_1) < 0) __PYX_ERR(0, 79, __pyx_L1_error)
  __Pyx_DECREF(__pyx_t_1); __pyx_t_1 = 0;
  __pyx_t_1 = __Pyx_PyObject_Call(__pyx_t_6, __pyx_t_2, __pyx_t_9); if (unlikely(!__pyx_t_1)) __PYX_ERR(0, 79, __pyx_L1_error)
  __Pyx_GOTREF(__pyx_t_1);
  __Pyx_DECREF(__pyx_t_6); __pyx_t_6 = 0;
  __Pyx_DECREF(__pyx_t_2); __pyx_t_2 = 0;
  __Pyx_DECREF(__pyx_t_9); __pyx_t_9 = 0;
  __pyx_v_v_badness = __pyx_t_1;
  __pyx_t_1 = 0;
```

```
 080:
```

```
 081:     # skip empty set, since that is equivalent to the condition: lambda>0
```

```
 082:     # skip sets that include the base; give vacuous conditions since the base has infinite bits
```

```
+083:     for n_set in range(1, 2**(n_using-1)):
```

```
  __pyx_t_1 = __Pyx_PyInt_SubtractObjC(__pyx_v_n_using, __pyx_int_1, 1, 0); if (unlikely(!__pyx_t_1)) __PYX_ERR(0, 83, __pyx_L1_error)
  __Pyx_GOTREF(__pyx_t_1);
  __pyx_t_9 = __Pyx_PyNumber_PowerOf2(__pyx_int_2, __pyx_t_1, Py_None); if (unlikely(!__pyx_t_9)) __PYX_ERR(0, 83, __pyx_L1_error)
  __Pyx_GOTREF(__pyx_t_9);
  __Pyx_DECREF(__pyx_t_1); __pyx_t_1 = 0;
  __pyx_t_1 = PyTuple_New(2); if (unlikely(!__pyx_t_1)) __PYX_ERR(0, 83, __pyx_L1_error)
  __Pyx_GOTREF(__pyx_t_1);
  __Pyx_INCREF(__pyx_int_1);
  __Pyx_GIVEREF(__pyx_int_1);
  PyTuple_SET_ITEM(__pyx_t_1, 0, __pyx_int_1);
  __Pyx_GIVEREF(__pyx_t_9);
  PyTuple_SET_ITEM(__pyx_t_1, 1, __pyx_t_9);
  __pyx_t_9 = 0;
  __pyx_t_9 = __Pyx_PyObject_Call(__pyx_builtin_range, __pyx_t_1, NULL); if (unlikely(!__pyx_t_9)) __PYX_ERR(0, 83, __pyx_L1_error)
  __Pyx_GOTREF(__pyx_t_9);
  __Pyx_DECREF(__pyx_t_1); __pyx_t_1 = 0;
  if (likely(PyList_CheckExact(__pyx_t_9)) || PyTuple_CheckExact(__pyx_t_9)) {
    __pyx_t_1 = __pyx_t_9; __Pyx_INCREF(__pyx_t_1); __pyx_t_10 = 0;
    __pyx_t_11 = NULL;
  } else {
    __pyx_t_10 = -1; __pyx_t_1 = PyObject_GetIter(__pyx_t_9); if (unlikely(!__pyx_t_1)) __PYX_ERR(0, 83, __pyx_L1_error)
    __Pyx_GOTREF(__pyx_t_1);
    __pyx_t_11 = Py_TYPE(__pyx_t_1)->tp_iternext; if (unlikely(!__pyx_t_11)) __PYX_ERR(0, 83, __pyx_L1_error)
  }
  __Pyx_DECREF(__pyx_t_9); __pyx_t_9 = 0;
  for (;;) {
    if (likely(!__pyx_t_11)) {
      if (likely(PyList_CheckExact(__pyx_t_1))) {
        if (__pyx_t_10 >= PyList_GET_SIZE(__pyx_t_1)) break;
        #if CYTHON_ASSUME_SAFE_MACROS && !CYTHON_AVOID_BORROWED_REFS
        __pyx_t_9 = PyList_GET_ITEM(__pyx_t_1, __pyx_t_10); __Pyx_INCREF(__pyx_t_9); __pyx_t_10++; if (unlikely(0 < 0)) __PYX_ERR(0, 83, __pyx_L1_error)
        #else
        __pyx_t_9 = PySequence_ITEM(__pyx_t_1, __pyx_t_10); __pyx_t_10++; if (unlikely(!__pyx_t_9)) __PYX_ERR(0, 83, __pyx_L1_error)
        __Pyx_GOTREF(__pyx_t_9);
        #endif
      } else {
        if (__pyx_t_10 >= PyTuple_GET_SIZE(__pyx_t_1)) break;
        #if CYTHON_ASSUME_SAFE_MACROS && !CYTHON_AVOID_BORROWED_REFS
        __pyx_t_9 = PyTuple_GET_ITEM(__pyx_t_1, __pyx_t_10); __Pyx_INCREF(__pyx_t_9); __pyx_t_10++; if (unlikely(0 < 0)) __PYX_ERR(0, 83, __pyx_L1_error)
        #else
        __pyx_t_9 = PySequence_ITEM(__pyx_t_1, __pyx_t_10); __pyx_t_10++; if (unlikely(!__pyx_t_9)) __PYX_ERR(0, 83, __pyx_L1_error)
        __Pyx_GOTREF(__pyx_t_9);
        #endif
      }
    } else {
      __pyx_t_9 = __pyx_t_11(__pyx_t_1);
      if (unlikely(!__pyx_t_9)) {
        PyObject* exc_type = PyErr_Occurred();
        if (exc_type) {
          if (likely(exc_type == PyExc_StopIteration || PyErr_GivenExceptionMatches(exc_type, PyExc_StopIteration))) PyErr_Clear();
          else __PYX_ERR(0, 83, __pyx_L1_error)
        }
        break;
      }
      __Pyx_GOTREF(__pyx_t_9);
    }
    __Pyx_XDECREF_SET(__pyx_v_n_set, __pyx_t_9);
    __pyx_t_9 = 0;
/* … */
  }
  __Pyx_DECREF(__pyx_t_1); __pyx_t_1 = 0;
```

```
+084:         n_set_c = 2**n_using - 1 - n_set
```

```
    __pyx_t_9 = __Pyx_PyNumber_PowerOf2(__pyx_int_2, __pyx_v_n_using, Py_None); if (unlikely(!__pyx_t_9)) __PYX_ERR(0, 84, __pyx_L1_error)
    __Pyx_GOTREF(__pyx_t_9);
    __pyx_t_2 = __Pyx_PyInt_SubtractObjC(__pyx_t_9, __pyx_int_1, 1, 0); if (unlikely(!__pyx_t_2)) __PYX_ERR(0, 84, __pyx_L1_error)
    __Pyx_GOTREF(__pyx_t_2);
    __Pyx_DECREF(__pyx_t_9); __pyx_t_9 = 0;
    __pyx_t_9 = PyNumber_Subtract(__pyx_t_2, __pyx_v_n_set); if (unlikely(!__pyx_t_9)) __PYX_ERR(0, 84, __pyx_L1_error)
    __Pyx_GOTREF(__pyx_t_9);
    __Pyx_DECREF(__pyx_t_2); __pyx_t_2 = 0;
    __Pyx_XDECREF_SET(__pyx_v_n_set_c, __pyx_t_9);
    __pyx_t_9 = 0;
```

```
 085:
```

```
 086:         # sum_{n in set} H(Z_n)
```

```
+087:         bv_set = num2bv(n_set, n_using)
```

```
    __pyx_t_2 = __Pyx_GetModuleGlobalName(__pyx_n_s_num2bv); if (unlikely(!__pyx_t_2)) __PYX_ERR(0, 87, __pyx_L1_error)
    __Pyx_GOTREF(__pyx_t_2);
    __pyx_t_6 = NULL;
    __pyx_t_5 = 0;
    if (CYTHON_UNPACK_METHODS && unlikely(PyMethod_Check(__pyx_t_2))) {
      __pyx_t_6 = PyMethod_GET_SELF(__pyx_t_2);
      if (likely(__pyx_t_6)) {
        PyObject* function = PyMethod_GET_FUNCTION(__pyx_t_2);
        __Pyx_INCREF(__pyx_t_6);
        __Pyx_INCREF(function);
        __Pyx_DECREF_SET(__pyx_t_2, function);
        __pyx_t_5 = 1;
      }
    }
    #if CYTHON_FAST_PYCALL
    if (PyFunction_Check(__pyx_t_2)) {
      PyObject *__pyx_temp[3] = {__pyx_t_6, __pyx_v_n_set, __pyx_v_n_using};
      __pyx_t_9 = __Pyx_PyFunction_FastCall(__pyx_t_2, __pyx_temp+1-__pyx_t_5, 2+__pyx_t_5); if (unlikely(!__pyx_t_9)) __PYX_ERR(0, 87, __pyx_L1_error)
      __Pyx_XDECREF(__pyx_t_6); __pyx_t_6 = 0;
      __Pyx_GOTREF(__pyx_t_9);
    } else
    #endif
    #if CYTHON_FAST_PYCCALL
    if (__Pyx_PyFastCFunction_Check(__pyx_t_2)) {
      PyObject *__pyx_temp[3] = {__pyx_t_6, __pyx_v_n_set, __pyx_v_n_using};
      __pyx_t_9 = __Pyx_PyCFunction_FastCall(__pyx_t_2, __pyx_temp+1-__pyx_t_5, 2+__pyx_t_5); if (unlikely(!__pyx_t_9)) __PYX_ERR(0, 87, __pyx_L1_error)
      __Pyx_XDECREF(__pyx_t_6); __pyx_t_6 = 0;
      __Pyx_GOTREF(__pyx_t_9);
    } else
    #endif
    {
      __pyx_t_4 = PyTuple_New(2+__pyx_t_5); if (unlikely(!__pyx_t_4)) __PYX_ERR(0, 87, __pyx_L1_error)
      __Pyx_GOTREF(__pyx_t_4);
      if (__pyx_t_6) {
        __Pyx_GIVEREF(__pyx_t_6); PyTuple_SET_ITEM(__pyx_t_4, 0, __pyx_t_6); __pyx_t_6 = NULL;
      }
      __Pyx_INCREF(__pyx_v_n_set);
      __Pyx_GIVEREF(__pyx_v_n_set);
      PyTuple_SET_ITEM(__pyx_t_4, 0+__pyx_t_5, __pyx_v_n_set);
      __Pyx_INCREF(__pyx_v_n_using);
      __Pyx_GIVEREF(__pyx_v_n_using);
      PyTuple_SET_ITEM(__pyx_t_4, 1+__pyx_t_5, __pyx_v_n_using);
      __pyx_t_9 = __Pyx_PyObject_Call(__pyx_t_2, __pyx_t_4, NULL); if (unlikely(!__pyx_t_9)) __PYX_ERR(0, 87, __pyx_L1_error)
      __Pyx_GOTREF(__pyx_t_9);
      __Pyx_DECREF(__pyx_t_4); __pyx_t_4 = 0;
    }
    __Pyx_DECREF(__pyx_t_2); __pyx_t_2 = 0;
    __Pyx_XDECREF_SET(__pyx_v_bv_set, __pyx_t_9);
    __pyx_t_9 = 0;
```

```
+088:         d_entropy_include = sum(v_ga_bits_u[bv_set])
```

```
    __pyx_t_9 = PyObject_GetItem(__pyx_v_v_ga_bits_u, __pyx_v_bv_set); if (unlikely(!__pyx_t_9)) __PYX_ERR(0, 88, __pyx_L1_error)
    __Pyx_GOTREF(__pyx_t_9);
    __pyx_t_2 = PyTuple_New(1); if (unlikely(!__pyx_t_2)) __PYX_ERR(0, 88, __pyx_L1_error)
    __Pyx_GOTREF(__pyx_t_2);
    __Pyx_GIVEREF(__pyx_t_9);
    PyTuple_SET_ITEM(__pyx_t_2, 0, __pyx_t_9);
    __pyx_t_9 = 0;
    __pyx_t_9 = __Pyx_PyObject_Call(__pyx_builtin_sum, __pyx_t_2, NULL); if (unlikely(!__pyx_t_9)) __PYX_ERR(0, 88, __pyx_L1_error)
    __Pyx_GOTREF(__pyx_t_9);
    __Pyx_DECREF(__pyx_t_2); __pyx_t_2 = 0;
    __Pyx_XDECREF_SET(__pyx_v_d_entropy_include, __pyx_t_9);
    __pyx_t_9 = 0;
```

```
 089:
```

```
 090:         # sum_{all n} I(Z_sn ; Z_{sk: k<n})
```

```
+091:         d_entropy_exclude = 0
```

```
    __Pyx_INCREF(__pyx_int_0);
    __Pyx_XDECREF_SET(__pyx_v_d_entropy_exclude, __pyx_int_0);
```

```
+092:         n_in_set = sum(bv_set)
```

```
    __pyx_t_9 = PyTuple_New(1); if (unlikely(!__pyx_t_9)) __PYX_ERR(0, 92, __pyx_L1_error)
    __Pyx_GOTREF(__pyx_t_9);
    __Pyx_INCREF(__pyx_v_bv_set);
    __Pyx_GIVEREF(__pyx_v_bv_set);
    PyTuple_SET_ITEM(__pyx_t_9, 0, __pyx_v_bv_set);
    __pyx_t_2 = __Pyx_PyObject_Call(__pyx_builtin_sum, __pyx_t_9, NULL); if (unlikely(!__pyx_t_2)) __PYX_ERR(0, 92, __pyx_L1_error)
    __Pyx_GOTREF(__pyx_t_2);
    __Pyx_DECREF(__pyx_t_9); __pyx_t_9 = 0;
    __Pyx_XDECREF_SET(__pyx_v_n_in_set, __pyx_t_2);
    __pyx_t_2 = 0;
```

```
+093:         n_set_kliii = 1
```

```
    __Pyx_INCREF(__pyx_int_1);
    __Pyx_XDECREF_SET(__pyx_v_n_set_kliii, __pyx_int_1);
```

```
+094:         for iii in range(1, n_in_set):
```

```
    __pyx_t_2 = PyTuple_New(2); if (unlikely(!__pyx_t_2)) __PYX_ERR(0, 94, __pyx_L1_error)
    __Pyx_GOTREF(__pyx_t_2);
    __Pyx_INCREF(__pyx_int_1);
    __Pyx_GIVEREF(__pyx_int_1);
    PyTuple_SET_ITEM(__pyx_t_2, 0, __pyx_int_1);
    __Pyx_INCREF(__pyx_v_n_in_set);
    __Pyx_GIVEREF(__pyx_v_n_in_set);
    PyTuple_SET_ITEM(__pyx_t_2, 1, __pyx_v_n_in_set);
    __pyx_t_9 = __Pyx_PyObject_Call(__pyx_builtin_range, __pyx_t_2, NULL); if (unlikely(!__pyx_t_9)) __PYX_ERR(0, 94, __pyx_L1_error)
    __Pyx_GOTREF(__pyx_t_9);
    __Pyx_DECREF(__pyx_t_2); __pyx_t_2 = 0;
    if (likely(PyList_CheckExact(__pyx_t_9)) || PyTuple_CheckExact(__pyx_t_9)) {
      __pyx_t_2 = __pyx_t_9; __Pyx_INCREF(__pyx_t_2); __pyx_t_12 = 0;
      __pyx_t_13 = NULL;
    } else {
      __pyx_t_12 = -1; __pyx_t_2 = PyObject_GetIter(__pyx_t_9); if (unlikely(!__pyx_t_2)) __PYX_ERR(0, 94, __pyx_L1_error)
      __Pyx_GOTREF(__pyx_t_2);
      __pyx_t_13 = Py_TYPE(__pyx_t_2)->tp_iternext; if (unlikely(!__pyx_t_13)) __PYX_ERR(0, 94, __pyx_L1_error)
    }
    __Pyx_DECREF(__pyx_t_9); __pyx_t_9 = 0;
    for (;;) {
      if (likely(!__pyx_t_13)) {
        if (likely(PyList_CheckExact(__pyx_t_2))) {
          if (__pyx_t_12 >= PyList_GET_SIZE(__pyx_t_2)) break;
          #if CYTHON_ASSUME_SAFE_MACROS && !CYTHON_AVOID_BORROWED_REFS
          __pyx_t_9 = PyList_GET_ITEM(__pyx_t_2, __pyx_t_12); __Pyx_INCREF(__pyx_t_9); __pyx_t_12++; if (unlikely(0 < 0)) __PYX_ERR(0, 94, __pyx_L1_error)
          #else
          __pyx_t_9 = PySequence_ITEM(__pyx_t_2, __pyx_t_12); __pyx_t_12++; if (unlikely(!__pyx_t_9)) __PYX_ERR(0, 94, __pyx_L1_error)
          __Pyx_GOTREF(__pyx_t_9);
          #endif
        } else {
          if (__pyx_t_12 >= PyTuple_GET_SIZE(__pyx_t_2)) break;
          #if CYTHON_ASSUME_SAFE_MACROS && !CYTHON_AVOID_BORROWED_REFS
          __pyx_t_9 = PyTuple_GET_ITEM(__pyx_t_2, __pyx_t_12); __Pyx_INCREF(__pyx_t_9); __pyx_t_12++; if (unlikely(0 < 0)) __PYX_ERR(0, 94, __pyx_L1_error)
          #else
          __pyx_t_9 = PySequence_ITEM(__pyx_t_2, __pyx_t_12); __pyx_t_12++; if (unlikely(!__pyx_t_9)) __PYX_ERR(0, 94, __pyx_L1_error)
          __Pyx_GOTREF(__pyx_t_9);
          #endif
        }
      } else {
        __pyx_t_9 = __pyx_t_13(__pyx_t_2);
        if (unlikely(!__pyx_t_9)) {
          PyObject* exc_type = PyErr_Occurred();
          if (exc_type) {
            if (likely(exc_type == PyExc_StopIteration || PyErr_GivenExceptionMatches(exc_type, PyExc_StopIteration))) PyErr_Clear();
            else __PYX_ERR(0, 94, __pyx_L1_error)
          }
          break;
        }
        __Pyx_GOTREF(__pyx_t_9);
      }
      __Pyx_XDECREF_SET(__pyx_v_iii, __pyx_t_9);
      __pyx_t_9 = 0;
/* … */
    }
    __Pyx_DECREF(__pyx_t_2); __pyx_t_2 = 0;
```

```
+095:             n_set_iii = 2**iii
```

```
      __pyx_t_9 = __Pyx_PyNumber_PowerOf2(__pyx_int_2, __pyx_v_iii, Py_None); if (unlikely(!__pyx_t_9)) __PYX_ERR(0, 95, __pyx_L1_error)
      __Pyx_GOTREF(__pyx_t_9);
      __Pyx_XDECREF_SET(__pyx_v_n_set_iii, __pyx_t_9);
      __pyx_t_9 = 0;
```

```
+096:             n_set_kleqiii = n_set_kliii + n_set_iii
```

```
      __pyx_t_9 = PyNumber_Add(__pyx_v_n_set_kliii, __pyx_v_n_set_iii); if (unlikely(!__pyx_t_9)) __PYX_ERR(0, 96, __pyx_L1_error)
      __Pyx_GOTREF(__pyx_t_9);
      __Pyx_XDECREF_SET(__pyx_v_n_set_kleqiii, __pyx_t_9);
      __pyx_t_9 = 0;
```

```
+097:             d_entropy_exclude = d_entropy_exclude + \
```

```
      __pyx_t_6 = PyNumber_Add(__pyx_v_d_entropy_exclude, __pyx_t_9); if (unlikely(!__pyx_t_6)) __PYX_ERR(0, 97, __pyx_L1_error)
      __Pyx_GOTREF(__pyx_t_6);
      __Pyx_DECREF(__pyx_t_9); __pyx_t_9 = 0;
      __Pyx_DECREF_SET(__pyx_v_d_entropy_exclude, __pyx_t_6);
      __pyx_t_6 = 0;
```

```
+098:                 np.log2(v_s_n_dets[n_set_iii]*v_s_n_dets[n_set_kliii] /
```

```
      __pyx_t_4 = __Pyx_GetModuleGlobalName(__pyx_n_s_np); if (unlikely(!__pyx_t_4)) __PYX_ERR(0, 98, __pyx_L1_error)
      __Pyx_GOTREF(__pyx_t_4);
      __pyx_t_6 = __Pyx_PyObject_GetAttrStr(__pyx_t_4, __pyx_n_s_log2); if (unlikely(!__pyx_t_6)) __PYX_ERR(0, 98, __pyx_L1_error)
      __Pyx_GOTREF(__pyx_t_6);
      __Pyx_DECREF(__pyx_t_4); __pyx_t_4 = 0;
      __pyx_t_4 = PyObject_GetItem(__pyx_v_v_s_n_dets, __pyx_v_n_set_iii); if (unlikely(!__pyx_t_4)) __PYX_ERR(0, 98, __pyx_L1_error)
      __Pyx_GOTREF(__pyx_t_4);
      __pyx_t_3 = PyObject_GetItem(__pyx_v_v_s_n_dets, __pyx_v_n_set_kliii); if (unlikely(!__pyx_t_3)) __PYX_ERR(0, 98, __pyx_L1_error)
      __Pyx_GOTREF(__pyx_t_3);
      __pyx_t_8 = PyNumber_Multiply(__pyx_t_4, __pyx_t_3); if (unlikely(!__pyx_t_8)) __PYX_ERR(0, 98, __pyx_L1_error)
      __Pyx_GOTREF(__pyx_t_8);
      __Pyx_DECREF(__pyx_t_4); __pyx_t_4 = 0;
      __Pyx_DECREF(__pyx_t_3); __pyx_t_3 = 0;
/* … */
      __pyx_t_4 = __Pyx_PyNumber_Divide(__pyx_t_8, __pyx_t_3); if (unlikely(!__pyx_t_4)) __PYX_ERR(0, 98, __pyx_L1_error)
      __Pyx_GOTREF(__pyx_t_4);
      __Pyx_DECREF(__pyx_t_8); __pyx_t_8 = 0;
      __Pyx_DECREF(__pyx_t_3); __pyx_t_3 = 0;
      __pyx_t_3 = NULL;
      if (CYTHON_UNPACK_METHODS && unlikely(PyMethod_Check(__pyx_t_6))) {
        __pyx_t_3 = PyMethod_GET_SELF(__pyx_t_6);
        if (likely(__pyx_t_3)) {
          PyObject* function = PyMethod_GET_FUNCTION(__pyx_t_6);
          __Pyx_INCREF(__pyx_t_3);
          __Pyx_INCREF(function);
          __Pyx_DECREF_SET(__pyx_t_6, function);
        }
      }
      if (!__pyx_t_3) {
        __pyx_t_9 = __Pyx_PyObject_CallOneArg(__pyx_t_6, __pyx_t_4); if (unlikely(!__pyx_t_9)) __PYX_ERR(0, 98, __pyx_L1_error)
        __Pyx_DECREF(__pyx_t_4); __pyx_t_4 = 0;
        __Pyx_GOTREF(__pyx_t_9);
      } else {
        #if CYTHON_FAST_PYCALL
        if (PyFunction_Check(__pyx_t_6)) {
          PyObject *__pyx_temp[2] = {__pyx_t_3, __pyx_t_4};
          __pyx_t_9 = __Pyx_PyFunction_FastCall(__pyx_t_6, __pyx_temp+1-1, 1+1); if (unlikely(!__pyx_t_9)) __PYX_ERR(0, 98, __pyx_L1_error)
          __Pyx_XDECREF(__pyx_t_3); __pyx_t_3 = 0;
          __Pyx_GOTREF(__pyx_t_9);
          __Pyx_DECREF(__pyx_t_4); __pyx_t_4 = 0;
        } else
        #endif
        #if CYTHON_FAST_PYCCALL
        if (__Pyx_PyFastCFunction_Check(__pyx_t_6)) {
          PyObject *__pyx_temp[2] = {__pyx_t_3, __pyx_t_4};
          __pyx_t_9 = __Pyx_PyCFunction_FastCall(__pyx_t_6, __pyx_temp+1-1, 1+1); if (unlikely(!__pyx_t_9)) __PYX_ERR(0, 98, __pyx_L1_error)
          __Pyx_XDECREF(__pyx_t_3); __pyx_t_3 = 0;
          __Pyx_GOTREF(__pyx_t_9);
          __Pyx_DECREF(__pyx_t_4); __pyx_t_4 = 0;
        } else
        #endif
        {
          __pyx_t_8 = PyTuple_New(1+1); if (unlikely(!__pyx_t_8)) __PYX_ERR(0, 98, __pyx_L1_error)
          __Pyx_GOTREF(__pyx_t_8);
          __Pyx_GIVEREF(__pyx_t_3); PyTuple_SET_ITEM(__pyx_t_8, 0, __pyx_t_3); __pyx_t_3 = NULL;
          __Pyx_GIVEREF(__pyx_t_4);
          PyTuple_SET_ITEM(__pyx_t_8, 0+1, __pyx_t_4);
          __pyx_t_4 = 0;
          __pyx_t_9 = __Pyx_PyObject_Call(__pyx_t_6, __pyx_t_8, NULL); if (unlikely(!__pyx_t_9)) __PYX_ERR(0, 98, __pyx_L1_error)
          __Pyx_GOTREF(__pyx_t_9);
          __Pyx_DECREF(__pyx_t_8); __pyx_t_8 = 0;
        }
      }
      __Pyx_DECREF(__pyx_t_6); __pyx_t_6 = 0;
```

```
+099:                         v_s_n_dets[n_set_kleqiii])
```

```
      __pyx_t_3 = PyObject_GetItem(__pyx_v_v_s_n_dets, __pyx_v_n_set_kleqiii); if (unlikely(!__pyx_t_3)) __PYX_ERR(0, 99, __pyx_L1_error)
      __Pyx_GOTREF(__pyx_t_3);
```

```
+100:             n_set_kliii = n_set_kleqiii
```

```
      __Pyx_INCREF(__pyx_v_n_set_kleqiii);
      __Pyx_DECREF_SET(__pyx_v_n_set_kliii, __pyx_v_n_set_kleqiii);
```

```
 101:
```

```
 102:         # I(Z_S;Z_{S^C})
```

```
+103:         d_mi = 0
```

```
    __Pyx_INCREF(__pyx_int_0);
    __Pyx_XDECREF_SET(__pyx_v_d_mi, __pyx_int_0);
```

```
+104:         if not (n_set == 2**n_using - 1):
```

```
    __pyx_t_2 = __Pyx_PyNumber_PowerOf2(__pyx_int_2, __pyx_v_n_using, Py_None); if (unlikely(!__pyx_t_2)) __PYX_ERR(0, 104, __pyx_L1_error)
    __Pyx_GOTREF(__pyx_t_2);
    __pyx_t_6 = __Pyx_PyInt_SubtractObjC(__pyx_t_2, __pyx_int_1, 1, 0); if (unlikely(!__pyx_t_6)) __PYX_ERR(0, 104, __pyx_L1_error)
    __Pyx_GOTREF(__pyx_t_6);
    __Pyx_DECREF(__pyx_t_2); __pyx_t_2 = 0;
    __pyx_t_2 = PyObject_RichCompare(__pyx_v_n_set, __pyx_t_6, Py_EQ); __Pyx_XGOTREF(__pyx_t_2); if (unlikely(!__pyx_t_2)) __PYX_ERR(0, 104, __pyx_L1_error)
    __Pyx_DECREF(__pyx_t_6); __pyx_t_6 = 0;
    __pyx_t_7 = __Pyx_PyObject_IsTrue(__pyx_t_2); if (unlikely(__pyx_t_7 < 0)) __PYX_ERR(0, 104, __pyx_L1_error)
    __Pyx_DECREF(__pyx_t_2); __pyx_t_2 = 0;
    __pyx_t_14 = ((!__pyx_t_7) != 0);
    if (__pyx_t_14) {
/* … */
    }
```

```
+105:             d_mi = np.log2(v_s_n_dets[n_set]*v_s_n_dets[n_set_c] /
```

```
      __pyx_t_6 = __Pyx_GetModuleGlobalName(__pyx_n_s_np); if (unlikely(!__pyx_t_6)) __PYX_ERR(0, 105, __pyx_L1_error)
      __Pyx_GOTREF(__pyx_t_6);
      __pyx_t_9 = __Pyx_PyObject_GetAttrStr(__pyx_t_6, __pyx_n_s_log2); if (unlikely(!__pyx_t_9)) __PYX_ERR(0, 105, __pyx_L1_error)
      __Pyx_GOTREF(__pyx_t_9);
      __Pyx_DECREF(__pyx_t_6); __pyx_t_6 = 0;
      __pyx_t_6 = PyObject_GetItem(__pyx_v_v_s_n_dets, __pyx_v_n_set); if (unlikely(!__pyx_t_6)) __PYX_ERR(0, 105, __pyx_L1_error)
      __Pyx_GOTREF(__pyx_t_6);
      __pyx_t_8 = PyObject_GetItem(__pyx_v_v_s_n_dets, __pyx_v_n_set_c); if (unlikely(!__pyx_t_8)) __PYX_ERR(0, 105, __pyx_L1_error)
      __Pyx_GOTREF(__pyx_t_8);
      __pyx_t_4 = PyNumber_Multiply(__pyx_t_6, __pyx_t_8); if (unlikely(!__pyx_t_4)) __PYX_ERR(0, 105, __pyx_L1_error)
      __Pyx_GOTREF(__pyx_t_4);
      __Pyx_DECREF(__pyx_t_6); __pyx_t_6 = 0;
      __Pyx_DECREF(__pyx_t_8); __pyx_t_8 = 0;
/* … */
      __pyx_t_6 = __Pyx_PyNumber_Divide(__pyx_t_4, __pyx_t_8); if (unlikely(!__pyx_t_6)) __PYX_ERR(0, 105, __pyx_L1_error)
      __Pyx_GOTREF(__pyx_t_6);
      __Pyx_DECREF(__pyx_t_4); __pyx_t_4 = 0;
      __Pyx_DECREF(__pyx_t_8); __pyx_t_8 = 0;
      __pyx_t_8 = NULL;
      if (CYTHON_UNPACK_METHODS && unlikely(PyMethod_Check(__pyx_t_9))) {
        __pyx_t_8 = PyMethod_GET_SELF(__pyx_t_9);
        if (likely(__pyx_t_8)) {
          PyObject* function = PyMethod_GET_FUNCTION(__pyx_t_9);
          __Pyx_INCREF(__pyx_t_8);
          __Pyx_INCREF(function);
          __Pyx_DECREF_SET(__pyx_t_9, function);
        }
      }
      if (!__pyx_t_8) {
        __pyx_t_2 = __Pyx_PyObject_CallOneArg(__pyx_t_9, __pyx_t_6); if (unlikely(!__pyx_t_2)) __PYX_ERR(0, 105, __pyx_L1_error)
        __Pyx_DECREF(__pyx_t_6); __pyx_t_6 = 0;
        __Pyx_GOTREF(__pyx_t_2);
      } else {
        #if CYTHON_FAST_PYCALL
        if (PyFunction_Check(__pyx_t_9)) {
          PyObject *__pyx_temp[2] = {__pyx_t_8, __pyx_t_6};
          __pyx_t_2 = __Pyx_PyFunction_FastCall(__pyx_t_9, __pyx_temp+1-1, 1+1); if (unlikely(!__pyx_t_2)) __PYX_ERR(0, 105, __pyx_L1_error)
          __Pyx_XDECREF(__pyx_t_8); __pyx_t_8 = 0;
          __Pyx_GOTREF(__pyx_t_2);
          __Pyx_DECREF(__pyx_t_6); __pyx_t_6 = 0;
        } else
        #endif
        #if CYTHON_FAST_PYCCALL
        if (__Pyx_PyFastCFunction_Check(__pyx_t_9)) {
          PyObject *__pyx_temp[2] = {__pyx_t_8, __pyx_t_6};
          __pyx_t_2 = __Pyx_PyCFunction_FastCall(__pyx_t_9, __pyx_temp+1-1, 1+1); if (unlikely(!__pyx_t_2)) __PYX_ERR(0, 105, __pyx_L1_error)
          __Pyx_XDECREF(__pyx_t_8); __pyx_t_8 = 0;
          __Pyx_GOTREF(__pyx_t_2);
          __Pyx_DECREF(__pyx_t_6); __pyx_t_6 = 0;
        } else
        #endif
        {
          __pyx_t_4 = PyTuple_New(1+1); if (unlikely(!__pyx_t_4)) __PYX_ERR(0, 105, __pyx_L1_error)
          __Pyx_GOTREF(__pyx_t_4);
          __Pyx_GIVEREF(__pyx_t_8); PyTuple_SET_ITEM(__pyx_t_4, 0, __pyx_t_8); __pyx_t_8 = NULL;
          __Pyx_GIVEREF(__pyx_t_6);
          PyTuple_SET_ITEM(__pyx_t_4, 0+1, __pyx_t_6);
          __pyx_t_6 = 0;
          __pyx_t_2 = __Pyx_PyObject_Call(__pyx_t_9, __pyx_t_4, NULL); if (unlikely(!__pyx_t_2)) __PYX_ERR(0, 105, __pyx_L1_error)
          __Pyx_GOTREF(__pyx_t_2);
          __Pyx_DECREF(__pyx_t_4); __pyx_t_4 = 0;
        }
      }
      __Pyx_DECREF(__pyx_t_9); __pyx_t_9 = 0;
      __Pyx_DECREF_SET(__pyx_v_d_mi, __pyx_t_2);
      __pyx_t_2 = 0;
```

```
+106:                            v_s_n_dets[-1])
```

```
      __pyx_t_8 = __Pyx_GetItemInt(__pyx_v_v_s_n_dets, -1L, long, 1, __Pyx_PyInt_From_long, 0, 1, 1); if (unlikely(!__pyx_t_8)) __PYX_ERR(0, 106, __pyx_L1_error)
      __Pyx_GOTREF(__pyx_t_8);
```

```
 107:
```

```
 108:         # entropy condition: v_badness[n_set] < 0
```

```
+109:         d_cond_ent = d_entropy_include - d_entropy_exclude - d_mi
```

```
    __pyx_t_2 = PyNumber_Subtract(__pyx_v_d_entropy_include, __pyx_v_d_entropy_exclude); if (unlikely(!__pyx_t_2)) __PYX_ERR(0, 109, __pyx_L1_error)
    __Pyx_GOTREF(__pyx_t_2);
    __pyx_t_9 = PyNumber_Subtract(__pyx_t_2, __pyx_v_d_mi); if (unlikely(!__pyx_t_9)) __PYX_ERR(0, 109, __pyx_L1_error)
    __Pyx_GOTREF(__pyx_t_9);
    __Pyx_DECREF(__pyx_t_2); __pyx_t_2 = 0;
    __Pyx_XDECREF_SET(__pyx_v_d_cond_ent, __pyx_t_9);
    __pyx_t_9 = 0;
```

```
+110:         v_cond_ents[n_set] = d_cond_ent
```

```
    if (unlikely(PyObject_SetItem(__pyx_v_v_cond_ents, __pyx_v_n_set, __pyx_v_d_cond_ent) < 0)) __PYX_ERR(0, 110, __pyx_L1_error)
```

```
+111:         v_badness[n_set] = np.real(d_cond_ent)-(sum(v_dc_bits_u[bv_set]) + d_lambda)
```

```
    __pyx_t_2 = __Pyx_GetModuleGlobalName(__pyx_n_s_np); if (unlikely(!__pyx_t_2)) __PYX_ERR(0, 111, __pyx_L1_error)
    __Pyx_GOTREF(__pyx_t_2);
    __pyx_t_4 = __Pyx_PyObject_GetAttrStr(__pyx_t_2, __pyx_n_s_real); if (unlikely(!__pyx_t_4)) __PYX_ERR(0, 111, __pyx_L1_error)
    __Pyx_GOTREF(__pyx_t_4);
    __Pyx_DECREF(__pyx_t_2); __pyx_t_2 = 0;
    __pyx_t_2 = NULL;
    if (CYTHON_UNPACK_METHODS && unlikely(PyMethod_Check(__pyx_t_4))) {
      __pyx_t_2 = PyMethod_GET_SELF(__pyx_t_4);
      if (likely(__pyx_t_2)) {
        PyObject* function = PyMethod_GET_FUNCTION(__pyx_t_4);
        __Pyx_INCREF(__pyx_t_2);
        __Pyx_INCREF(function);
        __Pyx_DECREF_SET(__pyx_t_4, function);
      }
    }
    if (!__pyx_t_2) {
      __pyx_t_9 = __Pyx_PyObject_CallOneArg(__pyx_t_4, __pyx_v_d_cond_ent); if (unlikely(!__pyx_t_9)) __PYX_ERR(0, 111, __pyx_L1_error)
      __Pyx_GOTREF(__pyx_t_9);
    } else {
      #if CYTHON_FAST_PYCALL
      if (PyFunction_Check(__pyx_t_4)) {
        PyObject *__pyx_temp[2] = {__pyx_t_2, __pyx_v_d_cond_ent};
        __pyx_t_9 = __Pyx_PyFunction_FastCall(__pyx_t_4, __pyx_temp+1-1, 1+1); if (unlikely(!__pyx_t_9)) __PYX_ERR(0, 111, __pyx_L1_error)
        __Pyx_XDECREF(__pyx_t_2); __pyx_t_2 = 0;
        __Pyx_GOTREF(__pyx_t_9);
      } else
      #endif
      #if CYTHON_FAST_PYCCALL
      if (__Pyx_PyFastCFunction_Check(__pyx_t_4)) {
        PyObject *__pyx_temp[2] = {__pyx_t_2, __pyx_v_d_cond_ent};
        __pyx_t_9 = __Pyx_PyCFunction_FastCall(__pyx_t_4, __pyx_temp+1-1, 1+1); if (unlikely(!__pyx_t_9)) __PYX_ERR(0, 111, __pyx_L1_error)
        __Pyx_XDECREF(__pyx_t_2); __pyx_t_2 = 0;
        __Pyx_GOTREF(__pyx_t_9);
      } else
      #endif
      {
        __pyx_t_6 = PyTuple_New(1+1); if (unlikely(!__pyx_t_6)) __PYX_ERR(0, 111, __pyx_L1_error)
        __Pyx_GOTREF(__pyx_t_6);
        __Pyx_GIVEREF(__pyx_t_2); PyTuple_SET_ITEM(__pyx_t_6, 0, __pyx_t_2); __pyx_t_2 = NULL;
        __Pyx_INCREF(__pyx_v_d_cond_ent);
        __Pyx_GIVEREF(__pyx_v_d_cond_ent);
        PyTuple_SET_ITEM(__pyx_t_6, 0+1, __pyx_v_d_cond_ent);
        __pyx_t_9 = __Pyx_PyObject_Call(__pyx_t_4, __pyx_t_6, NULL); if (unlikely(!__pyx_t_9)) __PYX_ERR(0, 111, __pyx_L1_error)
        __Pyx_GOTREF(__pyx_t_9);
        __Pyx_DECREF(__pyx_t_6); __pyx_t_6 = 0;
      }
    }
    __Pyx_DECREF(__pyx_t_4); __pyx_t_4 = 0;
    __pyx_t_4 = PyObject_GetItem(__pyx_v_v_dc_bits_u, __pyx_v_bv_set); if (unlikely(!__pyx_t_4)) __PYX_ERR(0, 111, __pyx_L1_error)
    __Pyx_GOTREF(__pyx_t_4);
    __pyx_t_6 = PyTuple_New(1); if (unlikely(!__pyx_t_6)) __PYX_ERR(0, 111, __pyx_L1_error)
    __Pyx_GOTREF(__pyx_t_6);
    __Pyx_GIVEREF(__pyx_t_4);
    PyTuple_SET_ITEM(__pyx_t_6, 0, __pyx_t_4);
    __pyx_t_4 = 0;
    __pyx_t_4 = __Pyx_PyObject_Call(__pyx_builtin_sum, __pyx_t_6, NULL); if (unlikely(!__pyx_t_4)) __PYX_ERR(0, 111, __pyx_L1_error)
    __Pyx_GOTREF(__pyx_t_4);
    __Pyx_DECREF(__pyx_t_6); __pyx_t_6 = 0;
    __pyx_t_6 = PyNumber_Add(__pyx_t_4, __pyx_v_d_lambda); if (unlikely(!__pyx_t_6)) __PYX_ERR(0, 111, __pyx_L1_error)
    __Pyx_GOTREF(__pyx_t_6);
    __Pyx_DECREF(__pyx_t_4); __pyx_t_4 = 0;
    __pyx_t_4 = PyNumber_Subtract(__pyx_t_9, __pyx_t_6); if (unlikely(!__pyx_t_4)) __PYX_ERR(0, 111, __pyx_L1_error)
    __Pyx_GOTREF(__pyx_t_4);
    __Pyx_DECREF(__pyx_t_9); __pyx_t_9 = 0;
    __Pyx_DECREF(__pyx_t_6); __pyx_t_6 = 0;
    if (unlikely(PyObject_SetItem(__pyx_v_v_badness, __pyx_v_n_set, __pyx_t_4) < 0)) __PYX_ERR(0, 111, __pyx_L1_error)
    __Pyx_DECREF(__pyx_t_4); __pyx_t_4 = 0;
```

```
 112:
```

```
+113:     return v_badness
```

```
  __Pyx_XDECREF(__pyx_r);
  __Pyx_INCREF(__pyx_v_v_badness);
  __pyx_r = __pyx_v_v_badness;
  goto __pyx_L0;
```

```
 114:
```

```
 115:
```

```
+116: def det_minors(nda_m):
```

```
/* Python wrapper */
static PyObject *__pyx_pw_9dist_recv_7checkdc_5det_minors(PyObject *__pyx_self, PyObject *__pyx_v_nda_m); /*proto*/
static char __pyx_doc_9dist_recv_7checkdc_4det_minors[] = " Compute the determinants of all the symmetric minors of a square matrix.\n\n        Input:\n            nda_m = N x N complex square np.ndarray\n        Output:\n            v_dets = vector of length 2^N where v_dets[iii] equals:\n                            det( nda_m( bv_iii, bv_iii ) ),\n                    where bv_iii is a binary vector representation of iii.\n    ";
static PyMethodDef __pyx_mdef_9dist_recv_7checkdc_5det_minors = {"det_minors", (PyCFunction)__pyx_pw_9dist_recv_7checkdc_5det_minors, METH_O, __pyx_doc_9dist_recv_7checkdc_4det_minors};
static PyObject *__pyx_pw_9dist_recv_7checkdc_5det_minors(PyObject *__pyx_self, PyObject *__pyx_v_nda_m) {
  PyObject *__pyx_r = 0;
  __Pyx_RefNannyDeclarations
  __Pyx_RefNannySetupContext("det_minors (wrapper)", 0);
  __pyx_r = __pyx_pf_9dist_recv_7checkdc_4det_minors(__pyx_self, ((PyObject *)__pyx_v_nda_m));

  /* function exit code */
  __Pyx_RefNannyFinishContext();
  return __pyx_r;
}

static PyObject *__pyx_pf_9dist_recv_7checkdc_4det_minors(CYTHON_UNUSED PyObject *__pyx_self, PyObject *__pyx_v_nda_m) {
  PyObject *__pyx_v_n_dim = NULL;
  PyObject *__pyx_v_v_dets = NULL;
  PyObject *__pyx_v_iii = NULL;
  PyObject *__pyx_v_bv_iii = NULL;
  PyObject *__pyx_r = NULL;
  __Pyx_TraceDeclarations
  __Pyx_TraceFrameInit(__pyx_codeobj__4)
  __Pyx_RefNannyDeclarations
  __Pyx_RefNannySetupContext("det_minors", 0);
  __Pyx_TraceCall("det_minors", __pyx_f[0], 116, 0, __PYX_ERR(0, 116, __pyx_L1_error));
/* … */
  /* function exit code */
  __pyx_L1_error:;
  __Pyx_XDECREF(__pyx_t_1);
  __Pyx_XDECREF(__pyx_t_2);
  __Pyx_XDECREF(__pyx_t_3);
  __Pyx_XDECREF(__pyx_t_4);
  __Pyx_XDECREF(__pyx_t_5);
  __Pyx_XDECREF(__pyx_t_9);
  __Pyx_AddTraceback("dist_recv.checkdc.det_minors", __pyx_clineno, __pyx_lineno, __pyx_filename);
  __pyx_r = NULL;
  __pyx_L0:;
  __Pyx_XDECREF(__pyx_v_n_dim);
  __Pyx_XDECREF(__pyx_v_v_dets);
  __Pyx_XDECREF(__pyx_v_iii);
  __Pyx_XDECREF(__pyx_v_bv_iii);
  __Pyx_XGIVEREF(__pyx_r);
  __Pyx_TraceReturn(__pyx_r, 0);
  __Pyx_RefNannyFinishContext();
  return __pyx_r;
}
/* … */
  __pyx_tuple__9 = PyTuple_Pack(5, __pyx_n_s_nda_m, __pyx_n_s_n_dim, __pyx_n_s_v_dets, __pyx_n_s_iii, __pyx_n_s_bv_iii); if (unlikely(!__pyx_tuple__9)) __PYX_ERR(0, 116, __pyx_L1_error)
  __Pyx_GOTREF(__pyx_tuple__9);
  __Pyx_GIVEREF(__pyx_tuple__9);
/* … */
  __pyx_t_1 = PyCFunction_NewEx(&__pyx_mdef_9dist_recv_7checkdc_5det_minors, NULL, __pyx_n_s_dist_recv_checkdc); if (unlikely(!__pyx_t_1)) __PYX_ERR(0, 116, __pyx_L1_error)
  __Pyx_GOTREF(__pyx_t_1);
  if (PyDict_SetItem(__pyx_d, __pyx_n_s_det_minors, __pyx_t_1) < 0) __PYX_ERR(0, 116, __pyx_L1_error)
  __Pyx_DECREF(__pyx_t_1); __pyx_t_1 = 0;
  __pyx_codeobj__4 = (PyObject*)__Pyx_PyCode_New(1, 0, 5, 0, 0, __pyx_empty_bytes, __pyx_empty_tuple, __pyx_empty_tuple, __pyx_tuple__9, __pyx_empty_tuple, __pyx_empty_tuple, __pyx_kp_s_Users_cdchapm2_Dropbox_Personal, __pyx_n_s_det_minors, 116, __pyx_empty_bytes); if (unlikely(!__pyx_codeobj__4)) __PYX_ERR(0, 116, __pyx_L1_error)
```

```
 117:     """ Compute the determinants of all the symmetric minors of a square matrix.
```

```
 118:
```

```
 119:         Input:
```

```
 120:             nda_m = N x N complex square np.ndarray
```

```
 121:         Output:
```

```
 122:             v_dets = vector of length 2^N where v_dets[iii] equals:
```

```
 123:                             det( nda_m( bv_iii, bv_iii ) ),
```

```
 124:                     where bv_iii is a binary vector representation of iii.
```

```
 125:     """
```

```
 126:
```

```
+127:     n_dim = nda_m.shape[0]
```

```
  __pyx_t_1 = __Pyx_PyObject_GetAttrStr(__pyx_v_nda_m, __pyx_n_s_shape); if (unlikely(!__pyx_t_1)) __PYX_ERR(0, 127, __pyx_L1_error)
  __Pyx_GOTREF(__pyx_t_1);
  __pyx_t_2 = __Pyx_GetItemInt(__pyx_t_1, 0, long, 1, __Pyx_PyInt_From_long, 0, 0, 1); if (unlikely(!__pyx_t_2)) __PYX_ERR(0, 127, __pyx_L1_error)
  __Pyx_GOTREF(__pyx_t_2);
  __Pyx_DECREF(__pyx_t_1); __pyx_t_1 = 0;
  __pyx_v_n_dim = __pyx_t_2;
  __pyx_t_2 = 0;
```

```
+128:     v_dets = np.zeros(2**n_dim, dtype=np.complex64)
```

```
  __pyx_t_2 = __Pyx_GetModuleGlobalName(__pyx_n_s_np); if (unlikely(!__pyx_t_2)) __PYX_ERR(0, 128, __pyx_L1_error)
  __Pyx_GOTREF(__pyx_t_2);
  __pyx_t_1 = __Pyx_PyObject_GetAttrStr(__pyx_t_2, __pyx_n_s_zeros); if (unlikely(!__pyx_t_1)) __PYX_ERR(0, 128, __pyx_L1_error)
  __Pyx_GOTREF(__pyx_t_1);
  __Pyx_DECREF(__pyx_t_2); __pyx_t_2 = 0;
  __pyx_t_2 = __Pyx_PyNumber_PowerOf2(__pyx_int_2, __pyx_v_n_dim, Py_None); if (unlikely(!__pyx_t_2)) __PYX_ERR(0, 128, __pyx_L1_error)
  __Pyx_GOTREF(__pyx_t_2);
  __pyx_t_3 = PyTuple_New(1); if (unlikely(!__pyx_t_3)) __PYX_ERR(0, 128, __pyx_L1_error)
  __Pyx_GOTREF(__pyx_t_3);
  __Pyx_GIVEREF(__pyx_t_2);
  PyTuple_SET_ITEM(__pyx_t_3, 0, __pyx_t_2);
  __pyx_t_2 = 0;
  __pyx_t_2 = PyDict_New(); if (unlikely(!__pyx_t_2)) __PYX_ERR(0, 128, __pyx_L1_error)
  __Pyx_GOTREF(__pyx_t_2);
  __pyx_t_4 = __Pyx_GetModuleGlobalName(__pyx_n_s_np); if (unlikely(!__pyx_t_4)) __PYX_ERR(0, 128, __pyx_L1_error)
  __Pyx_GOTREF(__pyx_t_4);
  __pyx_t_5 = __Pyx_PyObject_GetAttrStr(__pyx_t_4, __pyx_n_s_complex64); if (unlikely(!__pyx_t_5)) __PYX_ERR(0, 128, __pyx_L1_error)
  __Pyx_GOTREF(__pyx_t_5);
  __Pyx_DECREF(__pyx_t_4); __pyx_t_4 = 0;
  if (PyDict_SetItem(__pyx_t_2, __pyx_n_s_dtype, __pyx_t_5) < 0) __PYX_ERR(0, 128, __pyx_L1_error)
  __Pyx_DECREF(__pyx_t_5); __pyx_t_5 = 0;
  __pyx_t_5 = __Pyx_PyObject_Call(__pyx_t_1, __pyx_t_3, __pyx_t_2); if (unlikely(!__pyx_t_5)) __PYX_ERR(0, 128, __pyx_L1_error)
  __Pyx_GOTREF(__pyx_t_5);
  __Pyx_DECREF(__pyx_t_1); __pyx_t_1 = 0;
  __Pyx_DECREF(__pyx_t_3); __pyx_t_3 = 0;
  __Pyx_DECREF(__pyx_t_2); __pyx_t_2 = 0;
  __pyx_v_v_dets = __pyx_t_5;
  __pyx_t_5 = 0;
```

```
+129:     for iii in range(1, 2**n_dim):
```

```
  __pyx_t_5 = __Pyx_PyNumber_PowerOf2(__pyx_int_2, __pyx_v_n_dim, Py_None); if (unlikely(!__pyx_t_5)) __PYX_ERR(0, 129, __pyx_L1_error)
  __Pyx_GOTREF(__pyx_t_5);
  __pyx_t_2 = PyTuple_New(2); if (unlikely(!__pyx_t_2)) __PYX_ERR(0, 129, __pyx_L1_error)
  __Pyx_GOTREF(__pyx_t_2);
  __Pyx_INCREF(__pyx_int_1);
  __Pyx_GIVEREF(__pyx_int_1);
  PyTuple_SET_ITEM(__pyx_t_2, 0, __pyx_int_1);
  __Pyx_GIVEREF(__pyx_t_5);
  PyTuple_SET_ITEM(__pyx_t_2, 1, __pyx_t_5);
  __pyx_t_5 = 0;
  __pyx_t_5 = __Pyx_PyObject_Call(__pyx_builtin_range, __pyx_t_2, NULL); if (unlikely(!__pyx_t_5)) __PYX_ERR(0, 129, __pyx_L1_error)
  __Pyx_GOTREF(__pyx_t_5);
  __Pyx_DECREF(__pyx_t_2); __pyx_t_2 = 0;
  if (likely(PyList_CheckExact(__pyx_t_5)) || PyTuple_CheckExact(__pyx_t_5)) {
    __pyx_t_2 = __pyx_t_5; __Pyx_INCREF(__pyx_t_2); __pyx_t_6 = 0;
    __pyx_t_7 = NULL;
  } else {
    __pyx_t_6 = -1; __pyx_t_2 = PyObject_GetIter(__pyx_t_5); if (unlikely(!__pyx_t_2)) __PYX_ERR(0, 129, __pyx_L1_error)
    __Pyx_GOTREF(__pyx_t_2);
    __pyx_t_7 = Py_TYPE(__pyx_t_2)->tp_iternext; if (unlikely(!__pyx_t_7)) __PYX_ERR(0, 129, __pyx_L1_error)
  }
  __Pyx_DECREF(__pyx_t_5); __pyx_t_5 = 0;
  for (;;) {
    if (likely(!__pyx_t_7)) {
      if (likely(PyList_CheckExact(__pyx_t_2))) {
        if (__pyx_t_6 >= PyList_GET_SIZE(__pyx_t_2)) break;
        #if CYTHON_ASSUME_SAFE_MACROS && !CYTHON_AVOID_BORROWED_REFS
        __pyx_t_5 = PyList_GET_ITEM(__pyx_t_2, __pyx_t_6); __Pyx_INCREF(__pyx_t_5); __pyx_t_6++; if (unlikely(0 < 0)) __PYX_ERR(0, 129, __pyx_L1_error)
        #else
        __pyx_t_5 = PySequence_ITEM(__pyx_t_2, __pyx_t_6); __pyx_t_6++; if (unlikely(!__pyx_t_5)) __PYX_ERR(0, 129, __pyx_L1_error)
        __Pyx_GOTREF(__pyx_t_5);
        #endif
      } else {
        if (__pyx_t_6 >= PyTuple_GET_SIZE(__pyx_t_2)) break;
        #if CYTHON_ASSUME_SAFE_MACROS && !CYTHON_AVOID_BORROWED_REFS
        __pyx_t_5 = PyTuple_GET_ITEM(__pyx_t_2, __pyx_t_6); __Pyx_INCREF(__pyx_t_5); __pyx_t_6++; if (unlikely(0 < 0)) __PYX_ERR(0, 129, __pyx_L1_error)
        #else
        __pyx_t_5 = PySequence_ITEM(__pyx_t_2, __pyx_t_6); __pyx_t_6++; if (unlikely(!__pyx_t_5)) __PYX_ERR(0, 129, __pyx_L1_error)
        __Pyx_GOTREF(__pyx_t_5);
        #endif
      }
    } else {
      __pyx_t_5 = __pyx_t_7(__pyx_t_2);
      if (unlikely(!__pyx_t_5)) {
        PyObject* exc_type = PyErr_Occurred();
        if (exc_type) {
          if (likely(exc_type == PyExc_StopIteration || PyErr_GivenExceptionMatches(exc_type, PyExc_StopIteration))) PyErr_Clear();
          else __PYX_ERR(0, 129, __pyx_L1_error)
        }
        break;
      }
      __Pyx_GOTREF(__pyx_t_5);
    }
    __Pyx_XDECREF_SET(__pyx_v_iii, __pyx_t_5);
    __pyx_t_5 = 0;
/* … */
  }
  __Pyx_DECREF(__pyx_t_2); __pyx_t_2 = 0;
```

```
+130:         bv_iii = num2bv(iii, n_dim)
```

```
    __pyx_t_3 = __Pyx_GetModuleGlobalName(__pyx_n_s_num2bv); if (unlikely(!__pyx_t_3)) __PYX_ERR(0, 130, __pyx_L1_error)
    __Pyx_GOTREF(__pyx_t_3);
    __pyx_t_1 = NULL;
    __pyx_t_8 = 0;
    if (CYTHON_UNPACK_METHODS && unlikely(PyMethod_Check(__pyx_t_3))) {
      __pyx_t_1 = PyMethod_GET_SELF(__pyx_t_3);
      if (likely(__pyx_t_1)) {
        PyObject* function = PyMethod_GET_FUNCTION(__pyx_t_3);
        __Pyx_INCREF(__pyx_t_1);
        __Pyx_INCREF(function);
        __Pyx_DECREF_SET(__pyx_t_3, function);
        __pyx_t_8 = 1;
      }
    }
    #if CYTHON_FAST_PYCALL
    if (PyFunction_Check(__pyx_t_3)) {
      PyObject *__pyx_temp[3] = {__pyx_t_1, __pyx_v_iii, __pyx_v_n_dim};
      __pyx_t_5 = __Pyx_PyFunction_FastCall(__pyx_t_3, __pyx_temp+1-__pyx_t_8, 2+__pyx_t_8); if (unlikely(!__pyx_t_5)) __PYX_ERR(0, 130, __pyx_L1_error)
      __Pyx_XDECREF(__pyx_t_1); __pyx_t_1 = 0;
      __Pyx_GOTREF(__pyx_t_5);
    } else
    #endif
    #if CYTHON_FAST_PYCCALL
    if (__Pyx_PyFastCFunction_Check(__pyx_t_3)) {
      PyObject *__pyx_temp[3] = {__pyx_t_1, __pyx_v_iii, __pyx_v_n_dim};
      __pyx_t_5 = __Pyx_PyCFunction_FastCall(__pyx_t_3, __pyx_temp+1-__pyx_t_8, 2+__pyx_t_8); if (unlikely(!__pyx_t_5)) __PYX_ERR(0, 130, __pyx_L1_error)
      __Pyx_XDECREF(__pyx_t_1); __pyx_t_1 = 0;
      __Pyx_GOTREF(__pyx_t_5);
    } else
    #endif
    {
      __pyx_t_4 = PyTuple_New(2+__pyx_t_8); if (unlikely(!__pyx_t_4)) __PYX_ERR(0, 130, __pyx_L1_error)
      __Pyx_GOTREF(__pyx_t_4);
      if (__pyx_t_1) {
        __Pyx_GIVEREF(__pyx_t_1); PyTuple_SET_ITEM(__pyx_t_4, 0, __pyx_t_1); __pyx_t_1 = NULL;
      }
      __Pyx_INCREF(__pyx_v_iii);
      __Pyx_GIVEREF(__pyx_v_iii);
      PyTuple_SET_ITEM(__pyx_t_4, 0+__pyx_t_8, __pyx_v_iii);
      __Pyx_INCREF(__pyx_v_n_dim);
      __Pyx_GIVEREF(__pyx_v_n_dim);
      PyTuple_SET_ITEM(__pyx_t_4, 1+__pyx_t_8, __pyx_v_n_dim);
      __pyx_t_5 = __Pyx_PyObject_Call(__pyx_t_3, __pyx_t_4, NULL); if (unlikely(!__pyx_t_5)) __PYX_ERR(0, 130, __pyx_L1_error)
      __Pyx_GOTREF(__pyx_t_5);
      __Pyx_DECREF(__pyx_t_4); __pyx_t_4 = 0;
    }
    __Pyx_DECREF(__pyx_t_3); __pyx_t_3 = 0;
    __Pyx_XDECREF_SET(__pyx_v_bv_iii, __pyx_t_5);
    __pyx_t_5 = 0;
```

```
+131:         v_dets[iii] = np.linalg.det(nda_m[bv_iii][:, bv_iii])
```

```
    __pyx_t_3 = __Pyx_GetModuleGlobalName(__pyx_n_s_np); if (unlikely(!__pyx_t_3)) __PYX_ERR(0, 131, __pyx_L1_error)
    __Pyx_GOTREF(__pyx_t_3);
    __pyx_t_4 = __Pyx_PyObject_GetAttrStr(__pyx_t_3, __pyx_n_s_linalg); if (unlikely(!__pyx_t_4)) __PYX_ERR(0, 131, __pyx_L1_error)
    __Pyx_GOTREF(__pyx_t_4);
    __Pyx_DECREF(__pyx_t_3); __pyx_t_3 = 0;
    __pyx_t_3 = __Pyx_PyObject_GetAttrStr(__pyx_t_4, __pyx_n_s_det); if (unlikely(!__pyx_t_3)) __PYX_ERR(0, 131, __pyx_L1_error)
    __Pyx_GOTREF(__pyx_t_3);
    __Pyx_DECREF(__pyx_t_4); __pyx_t_4 = 0;
    __pyx_t_4 = PyObject_GetItem(__pyx_v_nda_m, __pyx_v_bv_iii); if (unlikely(!__pyx_t_4)) __PYX_ERR(0, 131, __pyx_L1_error)
    __Pyx_GOTREF(__pyx_t_4);
    __pyx_t_1 = PyTuple_New(2); if (unlikely(!__pyx_t_1)) __PYX_ERR(0, 131, __pyx_L1_error)
    __Pyx_GOTREF(__pyx_t_1);
    __Pyx_INCREF(__pyx_slice__5);
    __Pyx_GIVEREF(__pyx_slice__5);
    PyTuple_SET_ITEM(__pyx_t_1, 0, __pyx_slice__5);
    __Pyx_INCREF(__pyx_v_bv_iii);
    __Pyx_GIVEREF(__pyx_v_bv_iii);
    PyTuple_SET_ITEM(__pyx_t_1, 1, __pyx_v_bv_iii);
    __pyx_t_9 = PyObject_GetItem(__pyx_t_4, __pyx_t_1); if (unlikely(!__pyx_t_9)) __PYX_ERR(0, 131, __pyx_L1_error)
    __Pyx_GOTREF(__pyx_t_9);
    __Pyx_DECREF(__pyx_t_4); __pyx_t_4 = 0;
    __Pyx_DECREF(__pyx_t_1); __pyx_t_1 = 0;
    __pyx_t_1 = NULL;
    if (CYTHON_UNPACK_METHODS && likely(PyMethod_Check(__pyx_t_3))) {
      __pyx_t_1 = PyMethod_GET_SELF(__pyx_t_3);
      if (likely(__pyx_t_1)) {
        PyObject* function = PyMethod_GET_FUNCTION(__pyx_t_3);
        __Pyx_INCREF(__pyx_t_1);
        __Pyx_INCREF(function);
        __Pyx_DECREF_SET(__pyx_t_3, function);
      }
    }
    if (!__pyx_t_1) {
      __pyx_t_5 = __Pyx_PyObject_CallOneArg(__pyx_t_3, __pyx_t_9); if (unlikely(!__pyx_t_5)) __PYX_ERR(0, 131, __pyx_L1_error)
      __Pyx_DECREF(__pyx_t_9); __pyx_t_9 = 0;
      __Pyx_GOTREF(__pyx_t_5);
    } else {
      #if CYTHON_FAST_PYCALL
      if (PyFunction_Check(__pyx_t_3)) {
        PyObject *__pyx_temp[2] = {__pyx_t_1, __pyx_t_9};
        __pyx_t_5 = __Pyx_PyFunction_FastCall(__pyx_t_3, __pyx_temp+1-1, 1+1); if (unlikely(!__pyx_t_5)) __PYX_ERR(0, 131, __pyx_L1_error)
        __Pyx_XDECREF(__pyx_t_1); __pyx_t_1 = 0;
        __Pyx_GOTREF(__pyx_t_5);
        __Pyx_DECREF(__pyx_t_9); __pyx_t_9 = 0;
      } else
      #endif
      #if CYTHON_FAST_PYCCALL
      if (__Pyx_PyFastCFunction_Check(__pyx_t_3)) {
        PyObject *__pyx_temp[2] = {__pyx_t_1, __pyx_t_9};
        __pyx_t_5 = __Pyx_PyCFunction_FastCall(__pyx_t_3, __pyx_temp+1-1, 1+1); if (unlikely(!__pyx_t_5)) __PYX_ERR(0, 131, __pyx_L1_error)
        __Pyx_XDECREF(__pyx_t_1); __pyx_t_1 = 0;
        __Pyx_GOTREF(__pyx_t_5);
        __Pyx_DECREF(__pyx_t_9); __pyx_t_9 = 0;
      } else
      #endif
      {
        __pyx_t_4 = PyTuple_New(1+1); if (unlikely(!__pyx_t_4)) __PYX_ERR(0, 131, __pyx_L1_error)
        __Pyx_GOTREF(__pyx_t_4);
        __Pyx_GIVEREF(__pyx_t_1); PyTuple_SET_ITEM(__pyx_t_4, 0, __pyx_t_1); __pyx_t_1 = NULL;
        __Pyx_GIVEREF(__pyx_t_9);
        PyTuple_SET_ITEM(__pyx_t_4, 0+1, __pyx_t_9);
        __pyx_t_9 = 0;
        __pyx_t_5 = __Pyx_PyObject_Call(__pyx_t_3, __pyx_t_4, NULL); if (unlikely(!__pyx_t_5)) __PYX_ERR(0, 131, __pyx_L1_error)
        __Pyx_GOTREF(__pyx_t_5);
        __Pyx_DECREF(__pyx_t_4); __pyx_t_4 = 0;
      }
    }
    __Pyx_DECREF(__pyx_t_3); __pyx_t_3 = 0;
    if (unlikely(PyObject_SetItem(__pyx_v_v_dets, __pyx_v_iii, __pyx_t_5) < 0)) __PYX_ERR(0, 131, __pyx_L1_error)
    __Pyx_DECREF(__pyx_t_5); __pyx_t_5 = 0;
/* … */
  __pyx_slice__5 = PySlice_New(Py_None, Py_None, Py_None); if (unlikely(!__pyx_slice__5)) __PYX_ERR(0, 131, __pyx_L1_error)
  __Pyx_GOTREF(__pyx_slice__5);
  __Pyx_GIVEREF(__pyx_slice__5);
```

```
+132:     return v_dets
```

```
  __Pyx_XDECREF(__pyx_r);
  __Pyx_INCREF(__pyx_v_v_dets);
  __pyx_r = __pyx_v_v_dets;
  goto __pyx_L0;
```

```
 133:
```

```
 134:
```

```
+135: def num2bv(num, n_len):
```

```
/* Python wrapper */
static PyObject *__pyx_pw_9dist_recv_7checkdc_7num2bv(PyObject *__pyx_self, PyObject *__pyx_args, PyObject *__pyx_kwds); /*proto*/
static char __pyx_doc_9dist_recv_7checkdc_6num2bv[] = "Convert a number to a binary vector of some length";
static PyMethodDef __pyx_mdef_9dist_recv_7checkdc_7num2bv = {"num2bv", (PyCFunction)__pyx_pw_9dist_recv_7checkdc_7num2bv, METH_VARARGS|METH_KEYWORDS, __pyx_doc_9dist_recv_7checkdc_6num2bv};
static PyObject *__pyx_pw_9dist_recv_7checkdc_7num2bv(PyObject *__pyx_self, PyObject *__pyx_args, PyObject *__pyx_kwds) {
  PyObject *__pyx_v_num = 0;
  PyObject *__pyx_v_n_len = 0;
  PyObject *__pyx_r = 0;
  __Pyx_RefNannyDeclarations
  __Pyx_RefNannySetupContext("num2bv (wrapper)", 0);
  {
    static PyObject **__pyx_pyargnames[] = {&__pyx_n_s_num,&__pyx_n_s_n_len,0};
    PyObject* values[2] = {0,0};
    if (unlikely(__pyx_kwds)) {
      Py_ssize_t kw_args;
      const Py_ssize_t pos_args = PyTuple_GET_SIZE(__pyx_args);
      switch (pos_args) {
        case  2: values[1] = PyTuple_GET_ITEM(__pyx_args, 1);
        case  1: values[0] = PyTuple_GET_ITEM(__pyx_args, 0);
        case  0: break;
        default: goto __pyx_L5_argtuple_error;
      }
      kw_args = PyDict_Size(__pyx_kwds);
      switch (pos_args) {
        case  0:
        if (likely((values[0] = PyDict_GetItem(__pyx_kwds, __pyx_n_s_num)) != 0)) kw_args--;
        else goto __pyx_L5_argtuple_error;
        case  1:
        if (likely((values[1] = PyDict_GetItem(__pyx_kwds, __pyx_n_s_n_len)) != 0)) kw_args--;
        else {
          __Pyx_RaiseArgtupleInvalid("num2bv", 1, 2, 2, 1); __PYX_ERR(0, 135, __pyx_L3_error)
        }
      }
      if (unlikely(kw_args > 0)) {
        if (unlikely(__Pyx_ParseOptionalKeywords(__pyx_kwds, __pyx_pyargnames, 0, values, pos_args, "num2bv") < 0)) __PYX_ERR(0, 135, __pyx_L3_error)
      }
    } else if (PyTuple_GET_SIZE(__pyx_args) != 2) {
      goto __pyx_L5_argtuple_error;
    } else {
      values[0] = PyTuple_GET_ITEM(__pyx_args, 0);
      values[1] = PyTuple_GET_ITEM(__pyx_args, 1);
    }
    __pyx_v_num = values[0];
    __pyx_v_n_len = values[1];
  }
  goto __pyx_L4_argument_unpacking_done;
  __pyx_L5_argtuple_error:;
  __Pyx_RaiseArgtupleInvalid("num2bv", 1, 2, 2, PyTuple_GET_SIZE(__pyx_args)); __PYX_ERR(0, 135, __pyx_L3_error)
  __pyx_L3_error:;
  __Pyx_AddTraceback("dist_recv.checkdc.num2bv", __pyx_clineno, __pyx_lineno, __pyx_filename);
  __Pyx_RefNannyFinishContext();
  return NULL;
  __pyx_L4_argument_unpacking_done:;
  __pyx_r = __pyx_pf_9dist_recv_7checkdc_6num2bv(__pyx_self, __pyx_v_num, __pyx_v_n_len);

  /* function exit code */
  __Pyx_RefNannyFinishContext();
  return __pyx_r;
}

static PyObject *__pyx_pf_9dist_recv_7checkdc_6num2bv(CYTHON_UNUSED PyObject *__pyx_self, PyObject *__pyx_v_num, PyObject *__pyx_v_n_len) {
  PyObject *__pyx_v_ii = NULL;
  PyObject *__pyx_r = NULL;
  __Pyx_TraceDeclarations
  __Pyx_TraceFrameInit(__pyx_codeobj__6)
  __Pyx_RefNannyDeclarations
  __Pyx_RefNannySetupContext("num2bv", 0);
  __Pyx_TraceCall("num2bv", __pyx_f[0], 135, 0, __PYX_ERR(0, 135, __pyx_L1_error));
/* … */
  /* function exit code */
  __pyx_L1_error:;
  __Pyx_XDECREF(__pyx_t_1);
  __Pyx_XDECREF(__pyx_t_2);
  __Pyx_XDECREF(__pyx_t_3);
  __Pyx_XDECREF(__pyx_t_6);
  __Pyx_XDECREF(__pyx_t_7);
  __Pyx_AddTraceback("dist_recv.checkdc.num2bv", __pyx_clineno, __pyx_lineno, __pyx_filename);
  __pyx_r = NULL;
  __pyx_L0:;
  __Pyx_XDECREF(__pyx_v_ii);
  __Pyx_XGIVEREF(__pyx_r);
  __Pyx_TraceReturn(__pyx_r, 0);
  __Pyx_RefNannyFinishContext();
  return __pyx_r;
}
/* … */
  __pyx_tuple__10 = PyTuple_Pack(3, __pyx_n_s_num, __pyx_n_s_n_len, __pyx_n_s_ii); if (unlikely(!__pyx_tuple__10)) __PYX_ERR(0, 135, __pyx_L1_error)
  __Pyx_GOTREF(__pyx_tuple__10);
  __Pyx_GIVEREF(__pyx_tuple__10);
/* … */
  __pyx_t_1 = PyCFunction_NewEx(&__pyx_mdef_9dist_recv_7checkdc_7num2bv, NULL, __pyx_n_s_dist_recv_checkdc); if (unlikely(!__pyx_t_1)) __PYX_ERR(0, 135, __pyx_L1_error)
  __Pyx_GOTREF(__pyx_t_1);
  if (PyDict_SetItem(__pyx_d, __pyx_n_s_num2bv, __pyx_t_1) < 0) __PYX_ERR(0, 135, __pyx_L1_error)
  __Pyx_DECREF(__pyx_t_1); __pyx_t_1 = 0;
```

```
 136:     """Convert a number to a binary vector of some length"""
```

```
+137:     return [bool((2**ii & num)//(2**ii)) for ii in range(n_len-1, -1, -1)]
```

```
  __Pyx_XDECREF(__pyx_r);
  __pyx_t_1 = PyList_New(0); if (unlikely(!__pyx_t_1)) __PYX_ERR(0, 137, __pyx_L1_error)
  __Pyx_GOTREF(__pyx_t_1);
  __pyx_t_2 = __Pyx_PyInt_SubtractObjC(__pyx_v_n_len, __pyx_int_1, 1, 0); if (unlikely(!__pyx_t_2)) __PYX_ERR(0, 137, __pyx_L1_error)
  __Pyx_GOTREF(__pyx_t_2);
  __pyx_t_3 = PyTuple_New(3); if (unlikely(!__pyx_t_3)) __PYX_ERR(0, 137, __pyx_L1_error)
  __Pyx_GOTREF(__pyx_t_3);
  __Pyx_GIVEREF(__pyx_t_2);
  PyTuple_SET_ITEM(__pyx_t_3, 0, __pyx_t_2);
  __Pyx_INCREF(__pyx_int_neg_1);
  __Pyx_GIVEREF(__pyx_int_neg_1);
  PyTuple_SET_ITEM(__pyx_t_3, 1, __pyx_int_neg_1);
  __Pyx_INCREF(__pyx_int_neg_1);
  __Pyx_GIVEREF(__pyx_int_neg_1);
  PyTuple_SET_ITEM(__pyx_t_3, 2, __pyx_int_neg_1);
  __pyx_t_2 = 0;
  __pyx_t_2 = __Pyx_PyObject_Call(__pyx_builtin_range, __pyx_t_3, NULL); if (unlikely(!__pyx_t_2)) __PYX_ERR(0, 137, __pyx_L1_error)
  __Pyx_GOTREF(__pyx_t_2);
  __Pyx_DECREF(__pyx_t_3); __pyx_t_3 = 0;
  if (likely(PyList_CheckExact(__pyx_t_2)) || PyTuple_CheckExact(__pyx_t_2)) {
    __pyx_t_3 = __pyx_t_2; __Pyx_INCREF(__pyx_t_3); __pyx_t_4 = 0;
    __pyx_t_5 = NULL;
  } else {
    __pyx_t_4 = -1; __pyx_t_3 = PyObject_GetIter(__pyx_t_2); if (unlikely(!__pyx_t_3)) __PYX_ERR(0, 137, __pyx_L1_error)
    __Pyx_GOTREF(__pyx_t_3);
    __pyx_t_5 = Py_TYPE(__pyx_t_3)->tp_iternext; if (unlikely(!__pyx_t_5)) __PYX_ERR(0, 137, __pyx_L1_error)
  }
  __Pyx_DECREF(__pyx_t_2); __pyx_t_2 = 0;
  for (;;) {
    if (likely(!__pyx_t_5)) {
      if (likely(PyList_CheckExact(__pyx_t_3))) {
        if (__pyx_t_4 >= PyList_GET_SIZE(__pyx_t_3)) break;
        #if CYTHON_ASSUME_SAFE_MACROS && !CYTHON_AVOID_BORROWED_REFS
        __pyx_t_2 = PyList_GET_ITEM(__pyx_t_3, __pyx_t_4); __Pyx_INCREF(__pyx_t_2); __pyx_t_4++; if (unlikely(0 < 0)) __PYX_ERR(0, 137, __pyx_L1_error)
        #else
        __pyx_t_2 = PySequence_ITEM(__pyx_t_3, __pyx_t_4); __pyx_t_4++; if (unlikely(!__pyx_t_2)) __PYX_ERR(0, 137, __pyx_L1_error)
        __Pyx_GOTREF(__pyx_t_2);
        #endif
      } else {
        if (__pyx_t_4 >= PyTuple_GET_SIZE(__pyx_t_3)) break;
        #if CYTHON_ASSUME_SAFE_MACROS && !CYTHON_AVOID_BORROWED_REFS
        __pyx_t_2 = PyTuple_GET_ITEM(__pyx_t_3, __pyx_t_4); __Pyx_INCREF(__pyx_t_2); __pyx_t_4++; if (unlikely(0 < 0)) __PYX_ERR(0, 137, __pyx_L1_error)
        #else
        __pyx_t_2 = PySequence_ITEM(__pyx_t_3, __pyx_t_4); __pyx_t_4++; if (unlikely(!__pyx_t_2)) __PYX_ERR(0, 137, __pyx_L1_error)
        __Pyx_GOTREF(__pyx_t_2);
        #endif
      }
    } else {
      __pyx_t_2 = __pyx_t_5(__pyx_t_3);
      if (unlikely(!__pyx_t_2)) {
        PyObject* exc_type = PyErr_Occurred();
        if (exc_type) {
          if (likely(exc_type == PyExc_StopIteration || PyErr_GivenExceptionMatches(exc_type, PyExc_StopIteration))) PyErr_Clear();
          else __PYX_ERR(0, 137, __pyx_L1_error)
        }
        break;
      }
      __Pyx_GOTREF(__pyx_t_2);
    }
    __Pyx_XDECREF_SET(__pyx_v_ii, __pyx_t_2);
    __pyx_t_2 = 0;
    __pyx_t_2 = __Pyx_PyNumber_PowerOf2(__pyx_int_2, __pyx_v_ii, Py_None); if (unlikely(!__pyx_t_2)) __PYX_ERR(0, 137, __pyx_L1_error)
    __Pyx_GOTREF(__pyx_t_2);
    __pyx_t_6 = PyNumber_And(__pyx_t_2, __pyx_v_num); if (unlikely(!__pyx_t_6)) __PYX_ERR(0, 137, __pyx_L1_error)
    __Pyx_GOTREF(__pyx_t_6);
    __Pyx_DECREF(__pyx_t_2); __pyx_t_2 = 0;
    __pyx_t_2 = __Pyx_PyNumber_PowerOf2(__pyx_int_2, __pyx_v_ii, Py_None); if (unlikely(!__pyx_t_2)) __PYX_ERR(0, 137, __pyx_L1_error)
    __Pyx_GOTREF(__pyx_t_2);
    __pyx_t_7 = PyNumber_FloorDivide(__pyx_t_6, __pyx_t_2); if (unlikely(!__pyx_t_7)) __PYX_ERR(0, 137, __pyx_L1_error)
    __Pyx_GOTREF(__pyx_t_7);
    __Pyx_DECREF(__pyx_t_6); __pyx_t_6 = 0;
    __Pyx_DECREF(__pyx_t_2); __pyx_t_2 = 0;
    __pyx_t_8 = __Pyx_PyObject_IsTrue(__pyx_t_7); if (unlikely(__pyx_t_8 < 0)) __PYX_ERR(0, 137, __pyx_L1_error)
    __Pyx_DECREF(__pyx_t_7); __pyx_t_7 = 0;
    __pyx_t_7 = __Pyx_PyBool_FromLong((!(!__pyx_t_8))); if (unlikely(!__pyx_t_7)) __PYX_ERR(0, 137, __pyx_L1_error)
    __Pyx_GOTREF(__pyx_t_7);
    if (unlikely(__Pyx_ListComp_Append(__pyx_t_1, (PyObject*)__pyx_t_7))) __PYX_ERR(0, 137, __pyx_L1_error)
    __Pyx_DECREF(__pyx_t_7); __pyx_t_7 = 0;
  }
  __Pyx_DECREF(__pyx_t_3); __pyx_t_3 = 0;
  __pyx_r = __pyx_t_1;
  __pyx_t_1 = 0;
  goto __pyx_L0;
```
