# Supplementary material for: A Decentralized Receiver in Gaussian Interference
Source: Entropy (Basel). 2018 Apr 11;20(4):269. doi: 10.3390/e20040269 (PMC7512784; doi:10.3390/e20040269)
Supplement: Supplementary file 1 [file entropy-20-00269-s001.zip › dist_recv/opt_rates.html]

Cython: opt\_rates.pyx


Generated by Cython 0.25.2

Yellow lines hint at Python interaction.  
Click on a line that starts with a "`+`" to see the C code that Cython generated for it.

Raw output: opt\_rates.c

```
 001: """Optimize rates over a sum-bits constraint"""
```

```
+002: from scipy import optimize
```

```
  __pyx_t_1 = PyList_New(1); if (unlikely(!__pyx_t_1)) __PYX_ERR(0, 2, __pyx_L1_error)
  __Pyx_GOTREF(__pyx_t_1);
  __Pyx_INCREF(__pyx_n_s_optimize);
  __Pyx_GIVEREF(__pyx_n_s_optimize);
  PyList_SET_ITEM(__pyx_t_1, 0, __pyx_n_s_optimize);
  __pyx_t_2 = __Pyx_Import(__pyx_n_s_scipy, __pyx_t_1, -1); if (unlikely(!__pyx_t_2)) __PYX_ERR(0, 2, __pyx_L1_error)
  __Pyx_GOTREF(__pyx_t_2);
  __Pyx_DECREF(__pyx_t_1); __pyx_t_1 = 0;
  __pyx_t_1 = __Pyx_ImportFrom(__pyx_t_2, __pyx_n_s_optimize); if (unlikely(!__pyx_t_1)) __PYX_ERR(0, 2, __pyx_L1_error)
  __Pyx_GOTREF(__pyx_t_1);
  if (PyDict_SetItem(__pyx_d, __pyx_n_s_optimize, __pyx_t_1) < 0) __PYX_ERR(0, 2, __pyx_L1_error)
  __Pyx_DECREF(__pyx_t_1); __pyx_t_1 = 0;
  __Pyx_DECREF(__pyx_t_2); __pyx_t_2 = 0;
```

```
+003: import numpy as np
```

```
  __pyx_t_2 = __Pyx_Import(__pyx_n_s_numpy, 0, -1); if (unlikely(!__pyx_t_2)) __PYX_ERR(0, 3, __pyx_L1_error)
  __Pyx_GOTREF(__pyx_t_2);
  if (PyDict_SetItem(__pyx_d, __pyx_n_s_np, __pyx_t_2) < 0) __PYX_ERR(0, 3, __pyx_L1_error)
  __Pyx_DECREF(__pyx_t_2); __pyx_t_2 = 0;
```

```
+004: from dist_recv import rates
```

```
  __pyx_t_2 = PyList_New(1); if (unlikely(!__pyx_t_2)) __PYX_ERR(0, 4, __pyx_L1_error)
  __Pyx_GOTREF(__pyx_t_2);
  __Pyx_INCREF(__pyx_n_s_rates);
  __Pyx_GIVEREF(__pyx_n_s_rates);
  PyList_SET_ITEM(__pyx_t_2, 0, __pyx_n_s_rates);
  __pyx_t_1 = __Pyx_Import(__pyx_n_s_dist_recv, __pyx_t_2, -1); if (unlikely(!__pyx_t_1)) __PYX_ERR(0, 4, __pyx_L1_error)
  __Pyx_GOTREF(__pyx_t_1);
  __Pyx_DECREF(__pyx_t_2); __pyx_t_2 = 0;
  __pyx_t_2 = __Pyx_ImportFrom(__pyx_t_1, __pyx_n_s_rates); if (unlikely(!__pyx_t_2)) __PYX_ERR(0, 4, __pyx_L1_error)
  __Pyx_GOTREF(__pyx_t_2);
  if (PyDict_SetItem(__pyx_d, __pyx_n_s_rates, __pyx_t_2) < 0) __PYX_ERR(0, 4, __pyx_L1_error)
  __Pyx_DECREF(__pyx_t_2); __pyx_t_2 = 0;
  __Pyx_DECREF(__pyx_t_1); __pyx_t_1 = 0;
```

```
+005: from dist_recv import checkdc
```

```
  __pyx_t_1 = PyList_New(1); if (unlikely(!__pyx_t_1)) __PYX_ERR(0, 5, __pyx_L1_error)
  __Pyx_GOTREF(__pyx_t_1);
  __Pyx_INCREF(__pyx_n_s_checkdc);
  __Pyx_GIVEREF(__pyx_n_s_checkdc);
  PyList_SET_ITEM(__pyx_t_1, 0, __pyx_n_s_checkdc);
  __pyx_t_2 = __Pyx_Import(__pyx_n_s_dist_recv, __pyx_t_1, -1); if (unlikely(!__pyx_t_2)) __PYX_ERR(0, 5, __pyx_L1_error)
  __Pyx_GOTREF(__pyx_t_2);
  __Pyx_DECREF(__pyx_t_1); __pyx_t_1 = 0;
  __pyx_t_1 = __Pyx_ImportFrom(__pyx_t_2, __pyx_n_s_checkdc); if (unlikely(!__pyx_t_1)) __PYX_ERR(0, 5, __pyx_L1_error)
  __Pyx_GOTREF(__pyx_t_1);
  if (PyDict_SetItem(__pyx_d, __pyx_n_s_checkdc, __pyx_t_1) < 0) __PYX_ERR(0, 5, __pyx_L1_error)
  __Pyx_DECREF(__pyx_t_1); __pyx_t_1 = 0;
  __Pyx_DECREF(__pyx_t_2); __pyx_t_2 = 0;
```

```
 006:
```

```
 007:
```

```
+008: def opt_ga(v_channel, nda_interf, d_max_bits):
```

```
/* Python wrapper */
static PyObject *__pyx_pw_9dist_recv_9opt_rates_1opt_ga(PyObject *__pyx_self, PyObject *__pyx_args, PyObject *__pyx_kwds); /*proto*/
static char __pyx_doc_9dist_recv_9opt_rates_opt_ga[] = " Optimize rates.rate_ga for a given channel, interference, over a sum-bits constraint\n    Input:\n        v_channel = Channel vector h (size N+1 x 1)\n        nda_interf = Interference covariance matrix Sigma (size N+1 x N+1)\n        d_max_bits = Max total helper encoding rates.\n    Output:\n        opt_result = Optimization output (v_bits)\n    ";
static PyMethodDef __pyx_mdef_9dist_recv_9opt_rates_1opt_ga = {"opt_ga", (PyCFunction)__pyx_pw_9dist_recv_9opt_rates_1opt_ga, METH_VARARGS|METH_KEYWORDS, __pyx_doc_9dist_recv_9opt_rates_opt_ga};
static PyObject *__pyx_pw_9dist_recv_9opt_rates_1opt_ga(PyObject *__pyx_self, PyObject *__pyx_args, PyObject *__pyx_kwds) {
  PyObject *__pyx_v_v_channel = 0;
  PyObject *__pyx_v_nda_interf = 0;
  PyObject *__pyx_v_d_max_bits = 0;
  PyObject *__pyx_r = 0;
  __Pyx_RefNannyDeclarations
  __Pyx_RefNannySetupContext("opt_ga (wrapper)", 0);
  {
    static PyObject **__pyx_pyargnames[] = {&__pyx_n_s_v_channel,&__pyx_n_s_nda_interf,&__pyx_n_s_d_max_bits,0};
    PyObject* values[3] = {0,0,0};
    if (unlikely(__pyx_kwds)) {
      Py_ssize_t kw_args;
      const Py_ssize_t pos_args = PyTuple_GET_SIZE(__pyx_args);
      switch (pos_args) {
        case  3: values[2] = PyTuple_GET_ITEM(__pyx_args, 2);
        case  2: values[1] = PyTuple_GET_ITEM(__pyx_args, 1);
        case  1: values[0] = PyTuple_GET_ITEM(__pyx_args, 0);
        case  0: break;
        default: goto __pyx_L5_argtuple_error;
      }
      kw_args = PyDict_Size(__pyx_kwds);
      switch (pos_args) {
        case  0:
        if (likely((values[0] = PyDict_GetItem(__pyx_kwds, __pyx_n_s_v_channel)) != 0)) kw_args--;
        else goto __pyx_L5_argtuple_error;
        case  1:
        if (likely((values[1] = PyDict_GetItem(__pyx_kwds, __pyx_n_s_nda_interf)) != 0)) kw_args--;
        else {
          __Pyx_RaiseArgtupleInvalid("opt_ga", 1, 3, 3, 1); __PYX_ERR(0, 8, __pyx_L3_error)
        }
        case  2:
        if (likely((values[2] = PyDict_GetItem(__pyx_kwds, __pyx_n_s_d_max_bits)) != 0)) kw_args--;
        else {
          __Pyx_RaiseArgtupleInvalid("opt_ga", 1, 3, 3, 2); __PYX_ERR(0, 8, __pyx_L3_error)
        }
      }
      if (unlikely(kw_args > 0)) {
        if (unlikely(__Pyx_ParseOptionalKeywords(__pyx_kwds, __pyx_pyargnames, 0, values, pos_args, "opt_ga") < 0)) __PYX_ERR(0, 8, __pyx_L3_error)
      }
    } else if (PyTuple_GET_SIZE(__pyx_args) != 3) {
      goto __pyx_L5_argtuple_error;
    } else {
      values[0] = PyTuple_GET_ITEM(__pyx_args, 0);
      values[1] = PyTuple_GET_ITEM(__pyx_args, 1);
      values[2] = PyTuple_GET_ITEM(__pyx_args, 2);
    }
    __pyx_v_v_channel = values[0];
    __pyx_v_nda_interf = values[1];
    __pyx_v_d_max_bits = values[2];
  }
  goto __pyx_L4_argument_unpacking_done;
  __pyx_L5_argtuple_error:;
  __Pyx_RaiseArgtupleInvalid("opt_ga", 1, 3, 3, PyTuple_GET_SIZE(__pyx_args)); __PYX_ERR(0, 8, __pyx_L3_error)
  __pyx_L3_error:;
  __Pyx_AddTraceback("dist_recv.opt_rates.opt_ga", __pyx_clineno, __pyx_lineno, __pyx_filename);
  __Pyx_RefNannyFinishContext();
  return NULL;
  __pyx_L4_argument_unpacking_done:;
  __pyx_r = __pyx_pf_9dist_recv_9opt_rates_opt_ga(__pyx_self, __pyx_v_v_channel, __pyx_v_nda_interf, __pyx_v_d_max_bits);

  /* function exit code */
  __Pyx_RefNannyFinishContext();
  return __pyx_r;
}
/* … */
static PyObject *__pyx_pf_9dist_recv_9opt_rates_opt_ga(CYTHON_UNUSED PyObject *__pyx_self, PyObject *__pyx_v_v_channel, PyObject *__pyx_v_nda_interf, PyObject *__pyx_v_d_max_bits) {
  struct __pyx_obj_9dist_recv_9opt_rates___pyx_scope_struct__opt_ga *__pyx_cur_scope;
  PyObject *__pyx_v_n_helpers = NULL;
  PyObject *__pyx_v_fn_objective = NULL;
  PyObject *__pyx_v_v_intl_guess = NULL;
  PyObject *__pyx_v_nda_bounds = NULL;
  PyObject *__pyx_v_fn_sum_constraint = NULL;
  PyObject *__pyx_v_dict_constraints = NULL;
  PyObject *__pyx_v_opt_result = NULL;
  PyObject *__pyx_r = NULL;
  __Pyx_TraceDeclarations
  __Pyx_TraceFrameInit(__pyx_codeobj_)
  __Pyx_RefNannyDeclarations
  __Pyx_RefNannySetupContext("opt_ga", 0);
  __pyx_cur_scope = (struct __pyx_obj_9dist_recv_9opt_rates___pyx_scope_struct__opt_ga *)__pyx_tp_new_9dist_recv_9opt_rates___pyx_scope_struct__opt_ga(__pyx_ptype_9dist_recv_9opt_rates___pyx_scope_struct__opt_ga, __pyx_empty_tuple, NULL);
  if (unlikely(!__pyx_cur_scope)) {
    __pyx_cur_scope = ((struct __pyx_obj_9dist_recv_9opt_rates___pyx_scope_struct__opt_ga *)Py_None);
    __Pyx_INCREF(Py_None);
    __PYX_ERR(0, 8, __pyx_L1_error)
  } else {
    __Pyx_GOTREF(__pyx_cur_scope);
  }
  __Pyx_TraceCall("opt_ga", __pyx_f[0], 8, 0, __PYX_ERR(0, 8, __pyx_L1_error));
  __pyx_cur_scope->__pyx_v_v_channel = __pyx_v_v_channel;
  __Pyx_INCREF(__pyx_cur_scope->__pyx_v_v_channel);
  __Pyx_GIVEREF(__pyx_cur_scope->__pyx_v_v_channel);
  __pyx_cur_scope->__pyx_v_nda_interf = __pyx_v_nda_interf;
  __Pyx_INCREF(__pyx_cur_scope->__pyx_v_nda_interf);
  __Pyx_GIVEREF(__pyx_cur_scope->__pyx_v_nda_interf);
  __pyx_cur_scope->__pyx_v_d_max_bits = __pyx_v_d_max_bits;
  __Pyx_INCREF(__pyx_cur_scope->__pyx_v_d_max_bits);
  __Pyx_GIVEREF(__pyx_cur_scope->__pyx_v_d_max_bits);
/* … */
  /* function exit code */
  __pyx_L1_error:;
  __Pyx_XDECREF(__pyx_t_1);
  __Pyx_XDECREF(__pyx_t_3);
  __Pyx_XDECREF(__pyx_t_4);
  __Pyx_XDECREF(__pyx_t_5);
  __Pyx_XDECREF(__pyx_t_7);
  __Pyx_AddTraceback("dist_recv.opt_rates.opt_ga", __pyx_clineno, __pyx_lineno, __pyx_filename);
  __pyx_r = NULL;
  __pyx_L0:;
  __Pyx_XDECREF(__pyx_v_n_helpers);
  __Pyx_XDECREF(__pyx_v_fn_objective);
  __Pyx_XDECREF(__pyx_v_v_intl_guess);
  __Pyx_XDECREF(__pyx_v_nda_bounds);
  __Pyx_XDECREF(__pyx_v_fn_sum_constraint);
  __Pyx_XDECREF(__pyx_v_dict_constraints);
  __Pyx_XDECREF(__pyx_v_opt_result);
  __Pyx_DECREF(((PyObject *)__pyx_cur_scope));
  __Pyx_XGIVEREF(__pyx_r);
  __Pyx_TraceReturn(__pyx_r, 0);
  __Pyx_RefNannyFinishContext();
  return __pyx_r;
}
/* … */
  __pyx_tuple__7 = PyTuple_Pack(10, __pyx_n_s_v_channel, __pyx_n_s_nda_interf, __pyx_n_s_d_max_bits, __pyx_n_s_n_helpers, __pyx_n_s_fn_objective, __pyx_n_s_v_intl_guess, __pyx_n_s_nda_bounds, __pyx_n_s_fn_sum_constraint, __pyx_n_s_dict_constraints, __pyx_n_s_opt_result); if (unlikely(!__pyx_tuple__7)) __PYX_ERR(0, 8, __pyx_L1_error)
  __Pyx_GOTREF(__pyx_tuple__7);
  __Pyx_GIVEREF(__pyx_tuple__7);
/* … */
  __pyx_t_2 = PyCFunction_NewEx(&__pyx_mdef_9dist_recv_9opt_rates_1opt_ga, NULL, __pyx_n_s_dist_recv_opt_rates); if (unlikely(!__pyx_t_2)) __PYX_ERR(0, 8, __pyx_L1_error)
  __Pyx_GOTREF(__pyx_t_2);
  if (PyDict_SetItem(__pyx_d, __pyx_n_s_opt_ga, __pyx_t_2) < 0) __PYX_ERR(0, 8, __pyx_L1_error)
  __Pyx_DECREF(__pyx_t_2); __pyx_t_2 = 0;
  __pyx_codeobj_ = (PyObject*)__Pyx_PyCode_New(3, 0, 10, 0, 0, __pyx_empty_bytes, __pyx_empty_tuple, __pyx_empty_tuple, __pyx_tuple__7, __pyx_empty_tuple, __pyx_empty_tuple, __pyx_kp_s_Users_cdchapm2_Dropbox_Personal, __pyx_n_s_opt_ga, 8, __pyx_empty_bytes); if (unlikely(!__pyx_codeobj_)) __PYX_ERR(0, 8, __pyx_L1_error)
/* … */
struct __pyx_obj_9dist_recv_9opt_rates___pyx_scope_struct__opt_ga {
  PyObject_HEAD
  PyObject *__pyx_v_d_max_bits;
  PyObject *__pyx_v_nda_interf;
  PyObject *__pyx_v_v_channel;
};
```

```
 009:     """ Optimize rates.rate_ga for a given channel, interference, over a sum-bits constraint
```

```
 010:     Input:
```

```
 011:         v_channel = Channel vector h (size N+1 x 1)
```

```
 012:         nda_interf = Interference covariance matrix Sigma (size N+1 x N+1)
```

```
 013:         d_max_bits = Max total helper encoding rates.
```

```
 014:     Output:
```

```
 015:         opt_result = Optimization output (v_bits)
```

```
 016:     """
```

```
+017:     n_helpers = len(v_channel)-1
```

```
  __pyx_t_1 = __pyx_cur_scope->__pyx_v_v_channel;
  __Pyx_INCREF(__pyx_t_1);
  __pyx_t_2 = PyObject_Length(__pyx_t_1); if (unlikely(__pyx_t_2 == -1)) __PYX_ERR(0, 17, __pyx_L1_error)
  __Pyx_DECREF(__pyx_t_1); __pyx_t_1 = 0;
  __pyx_t_1 = PyInt_FromSsize_t((__pyx_t_2 - 1)); if (unlikely(!__pyx_t_1)) __PYX_ERR(0, 17, __pyx_L1_error)
  __Pyx_GOTREF(__pyx_t_1);
  __pyx_v_n_helpers = __pyx_t_1;
  __pyx_t_1 = 0;
```

```
 018:
```

```
+019:     fn_objective = lambda v_bits: -rates.rate_ga(v_channel, nda_interf, v_bits)
```

```
/* Python wrapper */
static PyObject *__pyx_pw_9dist_recv_9opt_rates_6opt_ga_lambda(PyObject *__pyx_self, PyObject *__pyx_v_v_bits); /*proto*/
static PyMethodDef __pyx_mdef_9dist_recv_9opt_rates_6opt_ga_lambda = {"lambda", (PyCFunction)__pyx_pw_9dist_recv_9opt_rates_6opt_ga_lambda, METH_O, 0};
static PyObject *__pyx_pw_9dist_recv_9opt_rates_6opt_ga_lambda(PyObject *__pyx_self, PyObject *__pyx_v_v_bits) {
  PyObject *__pyx_r = 0;
  __Pyx_RefNannyDeclarations
  __Pyx_RefNannySetupContext("lambda (wrapper)", 0);
  __pyx_r = __pyx_lambda_funcdef_lambda(__pyx_self, ((PyObject *)__pyx_v_v_bits));

  /* function exit code */
  __Pyx_RefNannyFinishContext();
  return __pyx_r;
}

static PyObject *__pyx_lambda_funcdef_lambda(PyObject *__pyx_self, PyObject *__pyx_v_v_bits) {
  struct __pyx_obj_9dist_recv_9opt_rates___pyx_scope_struct__opt_ga *__pyx_cur_scope;
  struct __pyx_obj_9dist_recv_9opt_rates___pyx_scope_struct__opt_ga *__pyx_outer_scope;
  PyObject *__pyx_r = NULL;
  __Pyx_TraceDeclarations
  __Pyx_RefNannyDeclarations
  __Pyx_RefNannySetupContext("lambda", 0);
  __pyx_outer_scope = (struct __pyx_obj_9dist_recv_9opt_rates___pyx_scope_struct__opt_ga *) __Pyx_CyFunction_GetClosure(__pyx_self);
  __pyx_cur_scope = __pyx_outer_scope;
  __Pyx_TraceCall("lambda", __pyx_f[0], 19, 0, __PYX_ERR(0, 19, __pyx_L1_error));
  __Pyx_XDECREF(__pyx_r);
  __pyx_t_2 = __Pyx_GetModuleGlobalName(__pyx_n_s_rates); if (unlikely(!__pyx_t_2)) __PYX_ERR(0, 19, __pyx_L1_error)
  __Pyx_GOTREF(__pyx_t_2);
  __pyx_t_3 = __Pyx_PyObject_GetAttrStr(__pyx_t_2, __pyx_n_s_rate_ga); if (unlikely(!__pyx_t_3)) __PYX_ERR(0, 19, __pyx_L1_error)
  __Pyx_GOTREF(__pyx_t_3);
  __Pyx_DECREF(__pyx_t_2); __pyx_t_2 = 0;
  if (unlikely(!__pyx_cur_scope->__pyx_v_v_channel)) { __Pyx_RaiseClosureNameError("v_channel"); __PYX_ERR(0, 19, __pyx_L1_error) }
  if (unlikely(!__pyx_cur_scope->__pyx_v_nda_interf)) { __Pyx_RaiseClosureNameError("nda_interf"); __PYX_ERR(0, 19, __pyx_L1_error) }
  __pyx_t_2 = NULL;
  __pyx_t_4 = 0;
  if (CYTHON_UNPACK_METHODS && unlikely(PyMethod_Check(__pyx_t_3))) {
    __pyx_t_2 = PyMethod_GET_SELF(__pyx_t_3);
    if (likely(__pyx_t_2)) {
      PyObject* function = PyMethod_GET_FUNCTION(__pyx_t_3);
      __Pyx_INCREF(__pyx_t_2);
      __Pyx_INCREF(function);
      __Pyx_DECREF_SET(__pyx_t_3, function);
      __pyx_t_4 = 1;
    }
  }
  #if CYTHON_FAST_PYCALL
  if (PyFunction_Check(__pyx_t_3)) {
    PyObject *__pyx_temp[4] = {__pyx_t_2, __pyx_cur_scope->__pyx_v_v_channel, __pyx_cur_scope->__pyx_v_nda_interf, __pyx_v_v_bits};
    __pyx_t_1 = __Pyx_PyFunction_FastCall(__pyx_t_3, __pyx_temp+1-__pyx_t_4, 3+__pyx_t_4); if (unlikely(!__pyx_t_1)) __PYX_ERR(0, 19, __pyx_L1_error)
    __Pyx_XDECREF(__pyx_t_2); __pyx_t_2 = 0;
    __Pyx_GOTREF(__pyx_t_1);
  } else
  #endif
  #if CYTHON_FAST_PYCCALL
  if (__Pyx_PyFastCFunction_Check(__pyx_t_3)) {
    PyObject *__pyx_temp[4] = {__pyx_t_2, __pyx_cur_scope->__pyx_v_v_channel, __pyx_cur_scope->__pyx_v_nda_interf, __pyx_v_v_bits};
    __pyx_t_1 = __Pyx_PyCFunction_FastCall(__pyx_t_3, __pyx_temp+1-__pyx_t_4, 3+__pyx_t_4); if (unlikely(!__pyx_t_1)) __PYX_ERR(0, 19, __pyx_L1_error)
    __Pyx_XDECREF(__pyx_t_2); __pyx_t_2 = 0;
    __Pyx_GOTREF(__pyx_t_1);
  } else
  #endif
  {
    __pyx_t_5 = PyTuple_New(3+__pyx_t_4); if (unlikely(!__pyx_t_5)) __PYX_ERR(0, 19, __pyx_L1_error)
    __Pyx_GOTREF(__pyx_t_5);
    if (__pyx_t_2) {
      __Pyx_GIVEREF(__pyx_t_2); PyTuple_SET_ITEM(__pyx_t_5, 0, __pyx_t_2); __pyx_t_2 = NULL;
    }
    __Pyx_INCREF(__pyx_cur_scope->__pyx_v_v_channel);
    __Pyx_GIVEREF(__pyx_cur_scope->__pyx_v_v_channel);
    PyTuple_SET_ITEM(__pyx_t_5, 0+__pyx_t_4, __pyx_cur_scope->__pyx_v_v_channel);
    __Pyx_INCREF(__pyx_cur_scope->__pyx_v_nda_interf);
    __Pyx_GIVEREF(__pyx_cur_scope->__pyx_v_nda_interf);
    PyTuple_SET_ITEM(__pyx_t_5, 1+__pyx_t_4, __pyx_cur_scope->__pyx_v_nda_interf);
    __Pyx_INCREF(__pyx_v_v_bits);
    __Pyx_GIVEREF(__pyx_v_v_bits);
    PyTuple_SET_ITEM(__pyx_t_5, 2+__pyx_t_4, __pyx_v_v_bits);
    __pyx_t_1 = __Pyx_PyObject_Call(__pyx_t_3, __pyx_t_5, NULL); if (unlikely(!__pyx_t_1)) __PYX_ERR(0, 19, __pyx_L1_error)
    __Pyx_GOTREF(__pyx_t_1);
    __Pyx_DECREF(__pyx_t_5); __pyx_t_5 = 0;
  }
  __Pyx_DECREF(__pyx_t_3); __pyx_t_3 = 0;
  __pyx_t_3 = PyNumber_Negative(__pyx_t_1); if (unlikely(!__pyx_t_3)) __PYX_ERR(0, 19, __pyx_L1_error)
  __Pyx_GOTREF(__pyx_t_3);
  __Pyx_DECREF(__pyx_t_1); __pyx_t_1 = 0;
  __pyx_r = __pyx_t_3;
  __pyx_t_3 = 0;
  goto __pyx_L0;

  /* function exit code */
  __pyx_L1_error:;
  __Pyx_XDECREF(__pyx_t_1);
  __Pyx_XDECREF(__pyx_t_2);
  __Pyx_XDECREF(__pyx_t_3);
  __Pyx_XDECREF(__pyx_t_5);
  __Pyx_AddTraceback("dist_recv.opt_rates.opt_ga.lambda", __pyx_clineno, __pyx_lineno, __pyx_filename);
  __pyx_r = NULL;
  __pyx_L0:;
  __Pyx_XGIVEREF(__pyx_r);
  __Pyx_TraceReturn(__pyx_r, 0);
  __Pyx_RefNannyFinishContext();
  return __pyx_r;
}
/* … */
  __pyx_t_1 = __Pyx_CyFunction_NewEx(&__pyx_mdef_9dist_recv_9opt_rates_6opt_ga_lambda, 0, __pyx_n_s_opt_ga_locals_lambda, ((PyObject*)__pyx_cur_scope), __pyx_n_s_dist_recv_opt_rates, __pyx_d, NULL); if (unlikely(!__pyx_t_1)) __PYX_ERR(0, 19, __pyx_L1_error)
  __Pyx_GOTREF(__pyx_t_1);
  __pyx_v_fn_objective = __pyx_t_1;
  __pyx_t_1 = 0;
```

```
+020:     v_intl_guess = 1*np.ones(n_helpers)*d_max_bits/n_helpers
```

```
  __pyx_t_3 = __Pyx_GetModuleGlobalName(__pyx_n_s_np); if (unlikely(!__pyx_t_3)) __PYX_ERR(0, 20, __pyx_L1_error)
  __Pyx_GOTREF(__pyx_t_3);
  __pyx_t_4 = __Pyx_PyObject_GetAttrStr(__pyx_t_3, __pyx_n_s_ones); if (unlikely(!__pyx_t_4)) __PYX_ERR(0, 20, __pyx_L1_error)
  __Pyx_GOTREF(__pyx_t_4);
  __Pyx_DECREF(__pyx_t_3); __pyx_t_3 = 0;
  __pyx_t_3 = NULL;
  if (CYTHON_UNPACK_METHODS && unlikely(PyMethod_Check(__pyx_t_4))) {
    __pyx_t_3 = PyMethod_GET_SELF(__pyx_t_4);
    if (likely(__pyx_t_3)) {
      PyObject* function = PyMethod_GET_FUNCTION(__pyx_t_4);
      __Pyx_INCREF(__pyx_t_3);
      __Pyx_INCREF(function);
      __Pyx_DECREF_SET(__pyx_t_4, function);
    }
  }
  if (!__pyx_t_3) {
    __pyx_t_1 = __Pyx_PyObject_CallOneArg(__pyx_t_4, __pyx_v_n_helpers); if (unlikely(!__pyx_t_1)) __PYX_ERR(0, 20, __pyx_L1_error)
    __Pyx_GOTREF(__pyx_t_1);
  } else {
    #if CYTHON_FAST_PYCALL
    if (PyFunction_Check(__pyx_t_4)) {
      PyObject *__pyx_temp[2] = {__pyx_t_3, __pyx_v_n_helpers};
      __pyx_t_1 = __Pyx_PyFunction_FastCall(__pyx_t_4, __pyx_temp+1-1, 1+1); if (unlikely(!__pyx_t_1)) __PYX_ERR(0, 20, __pyx_L1_error)
      __Pyx_XDECREF(__pyx_t_3); __pyx_t_3 = 0;
      __Pyx_GOTREF(__pyx_t_1);
    } else
    #endif
    #if CYTHON_FAST_PYCCALL
    if (__Pyx_PyFastCFunction_Check(__pyx_t_4)) {
      PyObject *__pyx_temp[2] = {__pyx_t_3, __pyx_v_n_helpers};
      __pyx_t_1 = __Pyx_PyCFunction_FastCall(__pyx_t_4, __pyx_temp+1-1, 1+1); if (unlikely(!__pyx_t_1)) __PYX_ERR(0, 20, __pyx_L1_error)
      __Pyx_XDECREF(__pyx_t_3); __pyx_t_3 = 0;
      __Pyx_GOTREF(__pyx_t_1);
    } else
    #endif
    {
      __pyx_t_5 = PyTuple_New(1+1); if (unlikely(!__pyx_t_5)) __PYX_ERR(0, 20, __pyx_L1_error)
      __Pyx_GOTREF(__pyx_t_5);
      __Pyx_GIVEREF(__pyx_t_3); PyTuple_SET_ITEM(__pyx_t_5, 0, __pyx_t_3); __pyx_t_3 = NULL;
      __Pyx_INCREF(__pyx_v_n_helpers);
      __Pyx_GIVEREF(__pyx_v_n_helpers);
      PyTuple_SET_ITEM(__pyx_t_5, 0+1, __pyx_v_n_helpers);
      __pyx_t_1 = __Pyx_PyObject_Call(__pyx_t_4, __pyx_t_5, NULL); if (unlikely(!__pyx_t_1)) __PYX_ERR(0, 20, __pyx_L1_error)
      __Pyx_GOTREF(__pyx_t_1);
      __Pyx_DECREF(__pyx_t_5); __pyx_t_5 = 0;
    }
  }
  __Pyx_DECREF(__pyx_t_4); __pyx_t_4 = 0;
  __pyx_t_4 = PyNumber_Multiply(__pyx_int_1, __pyx_t_1); if (unlikely(!__pyx_t_4)) __PYX_ERR(0, 20, __pyx_L1_error)
  __Pyx_GOTREF(__pyx_t_4);
  __Pyx_DECREF(__pyx_t_1); __pyx_t_1 = 0;
  __pyx_t_1 = PyNumber_Multiply(__pyx_t_4, __pyx_cur_scope->__pyx_v_d_max_bits); if (unlikely(!__pyx_t_1)) __PYX_ERR(0, 20, __pyx_L1_error)
  __Pyx_GOTREF(__pyx_t_1);
  __Pyx_DECREF(__pyx_t_4); __pyx_t_4 = 0;
  __pyx_t_4 = __Pyx_PyNumber_Divide(__pyx_t_1, __pyx_v_n_helpers); if (unlikely(!__pyx_t_4)) __PYX_ERR(0, 20, __pyx_L1_error)
  __Pyx_GOTREF(__pyx_t_4);
  __Pyx_DECREF(__pyx_t_1); __pyx_t_1 = 0;
  __pyx_v_v_intl_guess = __pyx_t_4;
  __pyx_t_4 = 0;
```

```
+021:     nda_bounds = np.tile((0, None), (n_helpers, 1))
```

```
  __pyx_t_1 = __Pyx_GetModuleGlobalName(__pyx_n_s_np); if (unlikely(!__pyx_t_1)) __PYX_ERR(0, 21, __pyx_L1_error)
  __Pyx_GOTREF(__pyx_t_1);
  __pyx_t_5 = __Pyx_PyObject_GetAttrStr(__pyx_t_1, __pyx_n_s_tile); if (unlikely(!__pyx_t_5)) __PYX_ERR(0, 21, __pyx_L1_error)
  __Pyx_GOTREF(__pyx_t_5);
  __Pyx_DECREF(__pyx_t_1); __pyx_t_1 = 0;
  __pyx_t_1 = PyTuple_New(2); if (unlikely(!__pyx_t_1)) __PYX_ERR(0, 21, __pyx_L1_error)
  __Pyx_GOTREF(__pyx_t_1);
  __Pyx_INCREF(__pyx_v_n_helpers);
  __Pyx_GIVEREF(__pyx_v_n_helpers);
  PyTuple_SET_ITEM(__pyx_t_1, 0, __pyx_v_n_helpers);
  __Pyx_INCREF(__pyx_int_1);
  __Pyx_GIVEREF(__pyx_int_1);
  PyTuple_SET_ITEM(__pyx_t_1, 1, __pyx_int_1);
  __pyx_t_3 = NULL;
  __pyx_t_6 = 0;
  if (CYTHON_UNPACK_METHODS && unlikely(PyMethod_Check(__pyx_t_5))) {
    __pyx_t_3 = PyMethod_GET_SELF(__pyx_t_5);
    if (likely(__pyx_t_3)) {
      PyObject* function = PyMethod_GET_FUNCTION(__pyx_t_5);
      __Pyx_INCREF(__pyx_t_3);
      __Pyx_INCREF(function);
      __Pyx_DECREF_SET(__pyx_t_5, function);
      __pyx_t_6 = 1;
    }
  }
  #if CYTHON_FAST_PYCALL
  if (PyFunction_Check(__pyx_t_5)) {
    PyObject *__pyx_temp[3] = {__pyx_t_3, __pyx_tuple__2, __pyx_t_1};
    __pyx_t_4 = __Pyx_PyFunction_FastCall(__pyx_t_5, __pyx_temp+1-__pyx_t_6, 2+__pyx_t_6); if (unlikely(!__pyx_t_4)) __PYX_ERR(0, 21, __pyx_L1_error)
    __Pyx_XDECREF(__pyx_t_3); __pyx_t_3 = 0;
    __Pyx_GOTREF(__pyx_t_4);
    __Pyx_DECREF(__pyx_t_1); __pyx_t_1 = 0;
  } else
  #endif
  #if CYTHON_FAST_PYCCALL
  if (__Pyx_PyFastCFunction_Check(__pyx_t_5)) {
    PyObject *__pyx_temp[3] = {__pyx_t_3, __pyx_tuple__2, __pyx_t_1};
    __pyx_t_4 = __Pyx_PyCFunction_FastCall(__pyx_t_5, __pyx_temp+1-__pyx_t_6, 2+__pyx_t_6); if (unlikely(!__pyx_t_4)) __PYX_ERR(0, 21, __pyx_L1_error)
    __Pyx_XDECREF(__pyx_t_3); __pyx_t_3 = 0;
    __Pyx_GOTREF(__pyx_t_4);
    __Pyx_DECREF(__pyx_t_1); __pyx_t_1 = 0;
  } else
  #endif
  {
    __pyx_t_7 = PyTuple_New(2+__pyx_t_6); if (unlikely(!__pyx_t_7)) __PYX_ERR(0, 21, __pyx_L1_error)
    __Pyx_GOTREF(__pyx_t_7);
    if (__pyx_t_3) {
      __Pyx_GIVEREF(__pyx_t_3); PyTuple_SET_ITEM(__pyx_t_7, 0, __pyx_t_3); __pyx_t_3 = NULL;
    }
    __Pyx_INCREF(__pyx_tuple__2);
    __Pyx_GIVEREF(__pyx_tuple__2);
    PyTuple_SET_ITEM(__pyx_t_7, 0+__pyx_t_6, __pyx_tuple__2);
    __Pyx_GIVEREF(__pyx_t_1);
    PyTuple_SET_ITEM(__pyx_t_7, 1+__pyx_t_6, __pyx_t_1);
    __pyx_t_1 = 0;
    __pyx_t_4 = __Pyx_PyObject_Call(__pyx_t_5, __pyx_t_7, NULL); if (unlikely(!__pyx_t_4)) __PYX_ERR(0, 21, __pyx_L1_error)
    __Pyx_GOTREF(__pyx_t_4);
    __Pyx_DECREF(__pyx_t_7); __pyx_t_7 = 0;
  }
  __Pyx_DECREF(__pyx_t_5); __pyx_t_5 = 0;
  __pyx_v_nda_bounds = __pyx_t_4;
  __pyx_t_4 = 0;
/* … */
  __pyx_tuple__2 = PyTuple_Pack(2, __pyx_int_0, Py_None); if (unlikely(!__pyx_tuple__2)) __PYX_ERR(0, 21, __pyx_L1_error)
  __Pyx_GOTREF(__pyx_tuple__2);
  __Pyx_GIVEREF(__pyx_tuple__2);
```

```
+022:     fn_sum_constraint = lambda v_bits: d_max_bits - sum(v_bits)
```

```
/* Python wrapper */
static PyObject *__pyx_pw_9dist_recv_9opt_rates_6opt_ga_1lambda1(PyObject *__pyx_self, PyObject *__pyx_v_v_bits); /*proto*/
static PyMethodDef __pyx_mdef_9dist_recv_9opt_rates_6opt_ga_1lambda1 = {"lambda1", (PyCFunction)__pyx_pw_9dist_recv_9opt_rates_6opt_ga_1lambda1, METH_O, 0};
static PyObject *__pyx_pw_9dist_recv_9opt_rates_6opt_ga_1lambda1(PyObject *__pyx_self, PyObject *__pyx_v_v_bits) {
  PyObject *__pyx_r = 0;
  __Pyx_RefNannyDeclarations
  __Pyx_RefNannySetupContext("lambda1 (wrapper)", 0);
  __pyx_r = __pyx_lambda_funcdef_lambda1(__pyx_self, ((PyObject *)__pyx_v_v_bits));

  /* function exit code */
  __Pyx_RefNannyFinishContext();
  return __pyx_r;
}

static PyObject *__pyx_lambda_funcdef_lambda1(PyObject *__pyx_self, PyObject *__pyx_v_v_bits) {
  struct __pyx_obj_9dist_recv_9opt_rates___pyx_scope_struct__opt_ga *__pyx_cur_scope;
  struct __pyx_obj_9dist_recv_9opt_rates___pyx_scope_struct__opt_ga *__pyx_outer_scope;
  PyObject *__pyx_r = NULL;
  __Pyx_TraceDeclarations
  __Pyx_RefNannyDeclarations
  __Pyx_RefNannySetupContext("lambda1", 0);
  __pyx_outer_scope = (struct __pyx_obj_9dist_recv_9opt_rates___pyx_scope_struct__opt_ga *) __Pyx_CyFunction_GetClosure(__pyx_self);
  __pyx_cur_scope = __pyx_outer_scope;
  __Pyx_TraceCall("lambda1", __pyx_f[0], 22, 0, __PYX_ERR(0, 22, __pyx_L1_error));
  __Pyx_XDECREF(__pyx_r);
  if (unlikely(!__pyx_cur_scope->__pyx_v_d_max_bits)) { __Pyx_RaiseClosureNameError("d_max_bits"); __PYX_ERR(0, 22, __pyx_L1_error) }
  __pyx_t_1 = PyTuple_New(1); if (unlikely(!__pyx_t_1)) __PYX_ERR(0, 22, __pyx_L1_error)
  __Pyx_GOTREF(__pyx_t_1);
  __Pyx_INCREF(__pyx_v_v_bits);
  __Pyx_GIVEREF(__pyx_v_v_bits);
  PyTuple_SET_ITEM(__pyx_t_1, 0, __pyx_v_v_bits);
  __pyx_t_2 = __Pyx_PyObject_Call(__pyx_builtin_sum, __pyx_t_1, NULL); if (unlikely(!__pyx_t_2)) __PYX_ERR(0, 22, __pyx_L1_error)
  __Pyx_GOTREF(__pyx_t_2);
  __Pyx_DECREF(__pyx_t_1); __pyx_t_1 = 0;
  __pyx_t_1 = PyNumber_Subtract(__pyx_cur_scope->__pyx_v_d_max_bits, __pyx_t_2); if (unlikely(!__pyx_t_1)) __PYX_ERR(0, 22, __pyx_L1_error)
  __Pyx_GOTREF(__pyx_t_1);
  __Pyx_DECREF(__pyx_t_2); __pyx_t_2 = 0;
  __pyx_r = __pyx_t_1;
  __pyx_t_1 = 0;
  goto __pyx_L0;

  /* function exit code */
  __pyx_L1_error:;
  __Pyx_XDECREF(__pyx_t_1);
  __Pyx_XDECREF(__pyx_t_2);
  __Pyx_AddTraceback("dist_recv.opt_rates.opt_ga.lambda1", __pyx_clineno, __pyx_lineno, __pyx_filename);
  __pyx_r = NULL;
  __pyx_L0:;
  __Pyx_XGIVEREF(__pyx_r);
  __Pyx_TraceReturn(__pyx_r, 0);
  __Pyx_RefNannyFinishContext();
  return __pyx_r;
}
/* … */
  __pyx_t_4 = __Pyx_CyFunction_NewEx(&__pyx_mdef_9dist_recv_9opt_rates_6opt_ga_1lambda1, 0, __pyx_n_s_opt_ga_locals_lambda, ((PyObject*)__pyx_cur_scope), __pyx_n_s_dist_recv_opt_rates, __pyx_d, NULL); if (unlikely(!__pyx_t_4)) __PYX_ERR(0, 22, __pyx_L1_error)
  __Pyx_GOTREF(__pyx_t_4);
  __pyx_v_fn_sum_constraint = __pyx_t_4;
  __pyx_t_4 = 0;
```

```
+023:     dict_constraints = dict(type='ineq', fun=fn_sum_constraint)
```

```
  __pyx_t_4 = PyDict_New(); if (unlikely(!__pyx_t_4)) __PYX_ERR(0, 23, __pyx_L1_error)
  __Pyx_GOTREF(__pyx_t_4);
  if (PyDict_SetItem(__pyx_t_4, __pyx_n_s_type, __pyx_n_s_ineq) < 0) __PYX_ERR(0, 23, __pyx_L1_error)
  if (PyDict_SetItem(__pyx_t_4, __pyx_n_s_fun, __pyx_v_fn_sum_constraint) < 0) __PYX_ERR(0, 23, __pyx_L1_error)
  __pyx_v_dict_constraints = ((PyObject*)__pyx_t_4);
  __pyx_t_4 = 0;
```

```
+024:     opt_result = optimize.minimize(fn_objective,
```

```
  __pyx_t_4 = __Pyx_GetModuleGlobalName(__pyx_n_s_optimize); if (unlikely(!__pyx_t_4)) __PYX_ERR(0, 24, __pyx_L1_error)
  __Pyx_GOTREF(__pyx_t_4);
  __pyx_t_5 = __Pyx_PyObject_GetAttrStr(__pyx_t_4, __pyx_n_s_minimize); if (unlikely(!__pyx_t_5)) __PYX_ERR(0, 24, __pyx_L1_error)
  __Pyx_GOTREF(__pyx_t_5);
  __Pyx_DECREF(__pyx_t_4); __pyx_t_4 = 0;
/* … */
  __pyx_t_4 = PyTuple_New(2); if (unlikely(!__pyx_t_4)) __PYX_ERR(0, 24, __pyx_L1_error)
  __Pyx_GOTREF(__pyx_t_4);
  __Pyx_INCREF(__pyx_v_fn_objective);
  __Pyx_GIVEREF(__pyx_v_fn_objective);
  PyTuple_SET_ITEM(__pyx_t_4, 0, __pyx_v_fn_objective);
  __Pyx_INCREF(__pyx_v_v_intl_guess);
  __Pyx_GIVEREF(__pyx_v_v_intl_guess);
  PyTuple_SET_ITEM(__pyx_t_4, 1, __pyx_v_v_intl_guess);
/* … */
  __pyx_t_1 = __Pyx_PyObject_Call(__pyx_t_5, __pyx_t_4, __pyx_t_7); if (unlikely(!__pyx_t_1)) __PYX_ERR(0, 24, __pyx_L1_error)
  __Pyx_GOTREF(__pyx_t_1);
  __Pyx_DECREF(__pyx_t_5); __pyx_t_5 = 0;
  __Pyx_DECREF(__pyx_t_4); __pyx_t_4 = 0;
  __Pyx_DECREF(__pyx_t_7); __pyx_t_7 = 0;
  __pyx_v_opt_result = __pyx_t_1;
  __pyx_t_1 = 0;
```

```
 025:                                    v_intl_guess,
```

```
+026:                                    bounds=nda_bounds,
```

```
  __pyx_t_7 = PyDict_New(); if (unlikely(!__pyx_t_7)) __PYX_ERR(0, 26, __pyx_L1_error)
  __Pyx_GOTREF(__pyx_t_7);
  if (PyDict_SetItem(__pyx_t_7, __pyx_n_s_bounds, __pyx_v_nda_bounds) < 0) __PYX_ERR(0, 26, __pyx_L1_error)
```

```
+027:                                    constraints=dict_constraints)
```

```
  if (PyDict_SetItem(__pyx_t_7, __pyx_n_s_constraints, __pyx_v_dict_constraints) < 0) __PYX_ERR(0, 26, __pyx_L1_error)
```

```
+028:     return opt_result
```

```
  __Pyx_XDECREF(__pyx_r);
  __Pyx_INCREF(__pyx_v_opt_result);
  __pyx_r = __pyx_v_opt_result;
  goto __pyx_L0;
```

```
 029:
```

```
 030:
```

```
+031: def rate_opt_ga(v_channel, nda_interf, opt_result_ga):
```

```
/* Python wrapper */
static PyObject *__pyx_pw_9dist_recv_9opt_rates_3rate_opt_ga(PyObject *__pyx_self, PyObject *__pyx_args, PyObject *__pyx_kwds); /*proto*/
static char __pyx_doc_9dist_recv_9opt_rates_2rate_opt_ga[] = "Given an opt_ga optimization result, return the associated ga rate it achieves";
static PyMethodDef __pyx_mdef_9dist_recv_9opt_rates_3rate_opt_ga = {"rate_opt_ga", (PyCFunction)__pyx_pw_9dist_recv_9opt_rates_3rate_opt_ga, METH_VARARGS|METH_KEYWORDS, __pyx_doc_9dist_recv_9opt_rates_2rate_opt_ga};
static PyObject *__pyx_pw_9dist_recv_9opt_rates_3rate_opt_ga(PyObject *__pyx_self, PyObject *__pyx_args, PyObject *__pyx_kwds) {
  PyObject *__pyx_v_v_channel = 0;
  PyObject *__pyx_v_nda_interf = 0;
  PyObject *__pyx_v_opt_result_ga = 0;
  PyObject *__pyx_r = 0;
  __Pyx_RefNannyDeclarations
  __Pyx_RefNannySetupContext("rate_opt_ga (wrapper)", 0);
  {
    static PyObject **__pyx_pyargnames[] = {&__pyx_n_s_v_channel,&__pyx_n_s_nda_interf,&__pyx_n_s_opt_result_ga,0};
    PyObject* values[3] = {0,0,0};
    if (unlikely(__pyx_kwds)) {
      Py_ssize_t kw_args;
      const Py_ssize_t pos_args = PyTuple_GET_SIZE(__pyx_args);
      switch (pos_args) {
        case  3: values[2] = PyTuple_GET_ITEM(__pyx_args, 2);
        case  2: values[1] = PyTuple_GET_ITEM(__pyx_args, 1);
        case  1: values[0] = PyTuple_GET_ITEM(__pyx_args, 0);
        case  0: break;
        default: goto __pyx_L5_argtuple_error;
      }
      kw_args = PyDict_Size(__pyx_kwds);
      switch (pos_args) {
        case  0:
        if (likely((values[0] = PyDict_GetItem(__pyx_kwds, __pyx_n_s_v_channel)) != 0)) kw_args--;
        else goto __pyx_L5_argtuple_error;
        case  1:
        if (likely((values[1] = PyDict_GetItem(__pyx_kwds, __pyx_n_s_nda_interf)) != 0)) kw_args--;
        else {
          __Pyx_RaiseArgtupleInvalid("rate_opt_ga", 1, 3, 3, 1); __PYX_ERR(0, 31, __pyx_L3_error)
        }
        case  2:
        if (likely((values[2] = PyDict_GetItem(__pyx_kwds, __pyx_n_s_opt_result_ga)) != 0)) kw_args--;
        else {
          __Pyx_RaiseArgtupleInvalid("rate_opt_ga", 1, 3, 3, 2); __PYX_ERR(0, 31, __pyx_L3_error)
        }
      }
      if (unlikely(kw_args > 0)) {
        if (unlikely(__Pyx_ParseOptionalKeywords(__pyx_kwds, __pyx_pyargnames, 0, values, pos_args, "rate_opt_ga") < 0)) __PYX_ERR(0, 31, __pyx_L3_error)
      }
    } else if (PyTuple_GET_SIZE(__pyx_args) != 3) {
      goto __pyx_L5_argtuple_error;
    } else {
      values[0] = PyTuple_GET_ITEM(__pyx_args, 0);
      values[1] = PyTuple_GET_ITEM(__pyx_args, 1);
      values[2] = PyTuple_GET_ITEM(__pyx_args, 2);
    }
    __pyx_v_v_channel = values[0];
    __pyx_v_nda_interf = values[1];
    __pyx_v_opt_result_ga = values[2];
  }
  goto __pyx_L4_argument_unpacking_done;
  __pyx_L5_argtuple_error:;
  __Pyx_RaiseArgtupleInvalid("rate_opt_ga", 1, 3, 3, PyTuple_GET_SIZE(__pyx_args)); __PYX_ERR(0, 31, __pyx_L3_error)
  __pyx_L3_error:;
  __Pyx_AddTraceback("dist_recv.opt_rates.rate_opt_ga", __pyx_clineno, __pyx_lineno, __pyx_filename);
  __Pyx_RefNannyFinishContext();
  return NULL;
  __pyx_L4_argument_unpacking_done:;
  __pyx_r = __pyx_pf_9dist_recv_9opt_rates_2rate_opt_ga(__pyx_self, __pyx_v_v_channel, __pyx_v_nda_interf, __pyx_v_opt_result_ga);

  /* function exit code */
  __Pyx_RefNannyFinishContext();
  return __pyx_r;
}

static PyObject *__pyx_pf_9dist_recv_9opt_rates_2rate_opt_ga(CYTHON_UNUSED PyObject *__pyx_self, PyObject *__pyx_v_v_channel, PyObject *__pyx_v_nda_interf, PyObject *__pyx_v_opt_result_ga) {
  PyObject *__pyx_v_v_bits = NULL;
  PyObject *__pyx_r = NULL;
  __Pyx_TraceDeclarations
  __Pyx_TraceFrameInit(__pyx_codeobj__3)
  __Pyx_RefNannyDeclarations
  __Pyx_RefNannySetupContext("rate_opt_ga", 0);
  __Pyx_TraceCall("rate_opt_ga", __pyx_f[0], 31, 0, __PYX_ERR(0, 31, __pyx_L1_error));
/* … */
  /* function exit code */
  __pyx_L1_error:;
  __Pyx_XDECREF(__pyx_t_1);
  __Pyx_XDECREF(__pyx_t_2);
  __Pyx_XDECREF(__pyx_t_3);
  __Pyx_XDECREF(__pyx_t_5);
  __Pyx_AddTraceback("dist_recv.opt_rates.rate_opt_ga", __pyx_clineno, __pyx_lineno, __pyx_filename);
  __pyx_r = NULL;
  __pyx_L0:;
  __Pyx_XDECREF(__pyx_v_v_bits);
  __Pyx_XGIVEREF(__pyx_r);
  __Pyx_TraceReturn(__pyx_r, 0);
  __Pyx_RefNannyFinishContext();
  return __pyx_r;
}
/* … */
  __pyx_tuple__8 = PyTuple_Pack(4, __pyx_n_s_v_channel, __pyx_n_s_nda_interf, __pyx_n_s_opt_result_ga, __pyx_n_s_v_bits); if (unlikely(!__pyx_tuple__8)) __PYX_ERR(0, 31, __pyx_L1_error)
  __Pyx_GOTREF(__pyx_tuple__8);
  __Pyx_GIVEREF(__pyx_tuple__8);
/* … */
  __pyx_t_2 = PyCFunction_NewEx(&__pyx_mdef_9dist_recv_9opt_rates_3rate_opt_ga, NULL, __pyx_n_s_dist_recv_opt_rates); if (unlikely(!__pyx_t_2)) __PYX_ERR(0, 31, __pyx_L1_error)
  __Pyx_GOTREF(__pyx_t_2);
  if (PyDict_SetItem(__pyx_d, __pyx_n_s_rate_opt_ga, __pyx_t_2) < 0) __PYX_ERR(0, 31, __pyx_L1_error)
  __Pyx_DECREF(__pyx_t_2); __pyx_t_2 = 0;
  __pyx_codeobj__3 = (PyObject*)__Pyx_PyCode_New(3, 0, 4, 0, 0, __pyx_empty_bytes, __pyx_empty_tuple, __pyx_empty_tuple, __pyx_tuple__8, __pyx_empty_tuple, __pyx_empty_tuple, __pyx_kp_s_Users_cdchapm2_Dropbox_Personal, __pyx_n_s_rate_opt_ga, 31, __pyx_empty_bytes); if (unlikely(!__pyx_codeobj__3)) __PYX_ERR(0, 31, __pyx_L1_error)
```

```
 032:     """Given an opt_ga optimization result, return the associated ga rate it achieves"""
```

```
+033:     v_bits = opt_result_ga.x
```

```
  __pyx_t_1 = __Pyx_PyObject_GetAttrStr(__pyx_v_opt_result_ga, __pyx_n_s_x); if (unlikely(!__pyx_t_1)) __PYX_ERR(0, 33, __pyx_L1_error)
  __Pyx_GOTREF(__pyx_t_1);
  __pyx_v_v_bits = __pyx_t_1;
  __pyx_t_1 = 0;
```

```
+034:     return rates.rate_ga(v_channel, nda_interf, v_bits)
```

```
  __Pyx_XDECREF(__pyx_r);
  __pyx_t_2 = __Pyx_GetModuleGlobalName(__pyx_n_s_rates); if (unlikely(!__pyx_t_2)) __PYX_ERR(0, 34, __pyx_L1_error)
  __Pyx_GOTREF(__pyx_t_2);
  __pyx_t_3 = __Pyx_PyObject_GetAttrStr(__pyx_t_2, __pyx_n_s_rate_ga); if (unlikely(!__pyx_t_3)) __PYX_ERR(0, 34, __pyx_L1_error)
  __Pyx_GOTREF(__pyx_t_3);
  __Pyx_DECREF(__pyx_t_2); __pyx_t_2 = 0;
  __pyx_t_2 = NULL;
  __pyx_t_4 = 0;
  if (CYTHON_UNPACK_METHODS && unlikely(PyMethod_Check(__pyx_t_3))) {
    __pyx_t_2 = PyMethod_GET_SELF(__pyx_t_3);
    if (likely(__pyx_t_2)) {
      PyObject* function = PyMethod_GET_FUNCTION(__pyx_t_3);
      __Pyx_INCREF(__pyx_t_2);
      __Pyx_INCREF(function);
      __Pyx_DECREF_SET(__pyx_t_3, function);
      __pyx_t_4 = 1;
    }
  }
  #if CYTHON_FAST_PYCALL
  if (PyFunction_Check(__pyx_t_3)) {
    PyObject *__pyx_temp[4] = {__pyx_t_2, __pyx_v_v_channel, __pyx_v_nda_interf, __pyx_v_v_bits};
    __pyx_t_1 = __Pyx_PyFunction_FastCall(__pyx_t_3, __pyx_temp+1-__pyx_t_4, 3+__pyx_t_4); if (unlikely(!__pyx_t_1)) __PYX_ERR(0, 34, __pyx_L1_error)
    __Pyx_XDECREF(__pyx_t_2); __pyx_t_2 = 0;
    __Pyx_GOTREF(__pyx_t_1);
  } else
  #endif
  #if CYTHON_FAST_PYCCALL
  if (__Pyx_PyFastCFunction_Check(__pyx_t_3)) {
    PyObject *__pyx_temp[4] = {__pyx_t_2, __pyx_v_v_channel, __pyx_v_nda_interf, __pyx_v_v_bits};
    __pyx_t_1 = __Pyx_PyCFunction_FastCall(__pyx_t_3, __pyx_temp+1-__pyx_t_4, 3+__pyx_t_4); if (unlikely(!__pyx_t_1)) __PYX_ERR(0, 34, __pyx_L1_error)
    __Pyx_XDECREF(__pyx_t_2); __pyx_t_2 = 0;
    __Pyx_GOTREF(__pyx_t_1);
  } else
  #endif
  {
    __pyx_t_5 = PyTuple_New(3+__pyx_t_4); if (unlikely(!__pyx_t_5)) __PYX_ERR(0, 34, __pyx_L1_error)
    __Pyx_GOTREF(__pyx_t_5);
    if (__pyx_t_2) {
      __Pyx_GIVEREF(__pyx_t_2); PyTuple_SET_ITEM(__pyx_t_5, 0, __pyx_t_2); __pyx_t_2 = NULL;
    }
    __Pyx_INCREF(__pyx_v_v_channel);
    __Pyx_GIVEREF(__pyx_v_v_channel);
    PyTuple_SET_ITEM(__pyx_t_5, 0+__pyx_t_4, __pyx_v_v_channel);
    __Pyx_INCREF(__pyx_v_nda_interf);
    __Pyx_GIVEREF(__pyx_v_nda_interf);
    PyTuple_SET_ITEM(__pyx_t_5, 1+__pyx_t_4, __pyx_v_nda_interf);
    __Pyx_INCREF(__pyx_v_v_bits);
    __Pyx_GIVEREF(__pyx_v_v_bits);
    PyTuple_SET_ITEM(__pyx_t_5, 2+__pyx_t_4, __pyx_v_v_bits);
    __pyx_t_1 = __Pyx_PyObject_Call(__pyx_t_3, __pyx_t_5, NULL); if (unlikely(!__pyx_t_1)) __PYX_ERR(0, 34, __pyx_L1_error)
    __Pyx_GOTREF(__pyx_t_1);
    __Pyx_DECREF(__pyx_t_5); __pyx_t_5 = 0;
  }
  __Pyx_DECREF(__pyx_t_3); __pyx_t_3 = 0;
  __pyx_r = __pyx_t_1;
  __pyx_t_1 = 0;
  goto __pyx_L0;
```

```
 035:
```

```
 036:
```

```
+037: def opt_dc(v_channel, nda_interf, d_max_bits):
```

```
/* Python wrapper */
static PyObject *__pyx_pw_9dist_recv_9opt_rates_5opt_dc(PyObject *__pyx_self, PyObject *__pyx_args, PyObject *__pyx_kwds); /*proto*/
static char __pyx_doc_9dist_recv_9opt_rates_4opt_dc[] = " \n    Optimize rates.rate_dc for a given channel, interference, over a sum-bits constraint\n        (barrier relaxation)\n    Input:\n        v_channel = Channel vector h (size N+1 x 1)\n        nda_interf = Interference covariance matrix Sigma (size N+1 x N+1)\n        d_max_bits = Max total helper encoding rates.\n    Output:\n        opt_result = Optimization output:\n                     opt_result.x[0:N] = v_dc_bits\n                     opt_result.x[N:-1] = v_ga_bits\n                     opt_result.x[-1] = d_lambda\n    ";
static PyMethodDef __pyx_mdef_9dist_recv_9opt_rates_5opt_dc = {"opt_dc", (PyCFunction)__pyx_pw_9dist_recv_9opt_rates_5opt_dc, METH_VARARGS|METH_KEYWORDS, __pyx_doc_9dist_recv_9opt_rates_4opt_dc};
static PyObject *__pyx_pw_9dist_recv_9opt_rates_5opt_dc(PyObject *__pyx_self, PyObject *__pyx_args, PyObject *__pyx_kwds) {
  PyObject *__pyx_v_v_channel = 0;
  PyObject *__pyx_v_nda_interf = 0;
  PyObject *__pyx_v_d_max_bits = 0;
  PyObject *__pyx_r = 0;
  __Pyx_RefNannyDeclarations
  __Pyx_RefNannySetupContext("opt_dc (wrapper)", 0);
  {
    static PyObject **__pyx_pyargnames[] = {&__pyx_n_s_v_channel,&__pyx_n_s_nda_interf,&__pyx_n_s_d_max_bits,0};
    PyObject* values[3] = {0,0,0};
    if (unlikely(__pyx_kwds)) {
      Py_ssize_t kw_args;
      const Py_ssize_t pos_args = PyTuple_GET_SIZE(__pyx_args);
      switch (pos_args) {
        case  3: values[2] = PyTuple_GET_ITEM(__pyx_args, 2);
        case  2: values[1] = PyTuple_GET_ITEM(__pyx_args, 1);
        case  1: values[0] = PyTuple_GET_ITEM(__pyx_args, 0);
        case  0: break;
        default: goto __pyx_L5_argtuple_error;
      }
      kw_args = PyDict_Size(__pyx_kwds);
      switch (pos_args) {
        case  0:
        if (likely((values[0] = PyDict_GetItem(__pyx_kwds, __pyx_n_s_v_channel)) != 0)) kw_args--;
        else goto __pyx_L5_argtuple_error;
        case  1:
        if (likely((values[1] = PyDict_GetItem(__pyx_kwds, __pyx_n_s_nda_interf)) != 0)) kw_args--;
        else {
          __Pyx_RaiseArgtupleInvalid("opt_dc", 1, 3, 3, 1); __PYX_ERR(0, 37, __pyx_L3_error)
        }
        case  2:
        if (likely((values[2] = PyDict_GetItem(__pyx_kwds, __pyx_n_s_d_max_bits)) != 0)) kw_args--;
        else {
          __Pyx_RaiseArgtupleInvalid("opt_dc", 1, 3, 3, 2); __PYX_ERR(0, 37, __pyx_L3_error)
        }
      }
      if (unlikely(kw_args > 0)) {
        if (unlikely(__Pyx_ParseOptionalKeywords(__pyx_kwds, __pyx_pyargnames, 0, values, pos_args, "opt_dc") < 0)) __PYX_ERR(0, 37, __pyx_L3_error)
      }
    } else if (PyTuple_GET_SIZE(__pyx_args) != 3) {
      goto __pyx_L5_argtuple_error;
    } else {
      values[0] = PyTuple_GET_ITEM(__pyx_args, 0);
      values[1] = PyTuple_GET_ITEM(__pyx_args, 1);
      values[2] = PyTuple_GET_ITEM(__pyx_args, 2);
    }
    __pyx_v_v_channel = values[0];
    __pyx_v_nda_interf = values[1];
    __pyx_v_d_max_bits = values[2];
  }
  goto __pyx_L4_argument_unpacking_done;
  __pyx_L5_argtuple_error:;
  __Pyx_RaiseArgtupleInvalid("opt_dc", 1, 3, 3, PyTuple_GET_SIZE(__pyx_args)); __PYX_ERR(0, 37, __pyx_L3_error)
  __pyx_L3_error:;
  __Pyx_AddTraceback("dist_recv.opt_rates.opt_dc", __pyx_clineno, __pyx_lineno, __pyx_filename);
  __Pyx_RefNannyFinishContext();
  return NULL;
  __pyx_L4_argument_unpacking_done:;
  __pyx_r = __pyx_pf_9dist_recv_9opt_rates_4opt_dc(__pyx_self, __pyx_v_v_channel, __pyx_v_nda_interf, __pyx_v_d_max_bits);

  /* function exit code */
  __Pyx_RefNannyFinishContext();
  return __pyx_r;
}
/* … */
static PyObject *__pyx_pf_9dist_recv_9opt_rates_4opt_dc(CYTHON_UNUSED PyObject *__pyx_self, PyObject *__pyx_v_v_channel, PyObject *__pyx_v_nda_interf, PyObject *__pyx_v_d_max_bits) {
  struct __pyx_obj_9dist_recv_9opt_rates___pyx_scope_struct_1_opt_dc *__pyx_cur_scope;
  PyObject *__pyx_v_v_opt_ga = NULL;
  PyObject *__pyx_v_d_opt_ga = NULL;
  PyObject *__pyx_v_v_intl_dc = NULL;
  PyObject *__pyx_v_v_intl_ga = NULL;
  PyObject *__pyx_v_d_intl_lambda = NULL;
  PyObject *__pyx_v_v_best = NULL;
  PyObject *__pyx_v_nda_bounds = NULL;
  PyObject *__pyx_v_fn_sum_bd = NULL;
  PyObject *__pyx_v_constraint_sum = NULL;
  PyObject *__pyx_v_fn_objective = NULL;
  PyObject *__pyx_v_opt_result = NULL;
  PyObject *__pyx_v_fn_dc_bd = NULL;
  PyObject *__pyx_v_constraint_dc = NULL;
  PyObject *__pyx_v_v_constraints = NULL;
  PyObject *__pyx_v_b_is_worse = NULL;
  PyObject *__pyx_r = NULL;
  __Pyx_TraceDeclarations
  __Pyx_TraceFrameInit(__pyx_codeobj__4)
  __Pyx_RefNannyDeclarations
  __Pyx_RefNannySetupContext("opt_dc", 0);
  __pyx_cur_scope = (struct __pyx_obj_9dist_recv_9opt_rates___pyx_scope_struct_1_opt_dc *)__pyx_tp_new_9dist_recv_9opt_rates___pyx_scope_struct_1_opt_dc(__pyx_ptype_9dist_recv_9opt_rates___pyx_scope_struct_1_opt_dc, __pyx_empty_tuple, NULL);
  if (unlikely(!__pyx_cur_scope)) {
    __pyx_cur_scope = ((struct __pyx_obj_9dist_recv_9opt_rates___pyx_scope_struct_1_opt_dc *)Py_None);
    __Pyx_INCREF(Py_None);
    __PYX_ERR(0, 37, __pyx_L1_error)
  } else {
    __Pyx_GOTREF(__pyx_cur_scope);
  }
  __Pyx_TraceCall("opt_dc", __pyx_f[0], 37, 0, __PYX_ERR(0, 37, __pyx_L1_error));
  __pyx_cur_scope->__pyx_v_v_channel = __pyx_v_v_channel;
  __Pyx_INCREF(__pyx_cur_scope->__pyx_v_v_channel);
  __Pyx_GIVEREF(__pyx_cur_scope->__pyx_v_v_channel);
  __pyx_cur_scope->__pyx_v_nda_interf = __pyx_v_nda_interf;
  __Pyx_INCREF(__pyx_cur_scope->__pyx_v_nda_interf);
  __Pyx_GIVEREF(__pyx_cur_scope->__pyx_v_nda_interf);
  __pyx_cur_scope->__pyx_v_d_max_bits = __pyx_v_d_max_bits;
  __Pyx_INCREF(__pyx_cur_scope->__pyx_v_d_max_bits);
  __Pyx_GIVEREF(__pyx_cur_scope->__pyx_v_d_max_bits);
/* … */
  /* function exit code */
  __pyx_L1_error:;
  __Pyx_XDECREF(__pyx_t_1);
  __Pyx_XDECREF(__pyx_t_3);
  __Pyx_XDECREF(__pyx_t_4);
  __Pyx_XDECREF(__pyx_t_6);
  __Pyx_XDECREF(__pyx_t_7);
  __Pyx_XDECREF(__pyx_t_8);
  __Pyx_XDECREF(__pyx_t_9);
  __Pyx_XDECREF(__pyx_t_10);
  __Pyx_AddTraceback("dist_recv.opt_rates.opt_dc", __pyx_clineno, __pyx_lineno, __pyx_filename);
  __pyx_r = NULL;
  __pyx_L0:;
  __Pyx_XDECREF(__pyx_v_v_opt_ga);
  __Pyx_XDECREF(__pyx_v_d_opt_ga);
  __Pyx_XDECREF(__pyx_v_v_intl_dc);
  __Pyx_XDECREF(__pyx_v_v_intl_ga);
  __Pyx_XDECREF(__pyx_v_d_intl_lambda);
  __Pyx_XDECREF(__pyx_v_v_best);
  __Pyx_XDECREF(__pyx_v_nda_bounds);
  __Pyx_XDECREF(__pyx_v_fn_sum_bd);
  __Pyx_XDECREF(__pyx_v_constraint_sum);
  __Pyx_XDECREF(__pyx_v_fn_objective);
  __Pyx_XDECREF(__pyx_v_opt_result);
  __Pyx_XDECREF(__pyx_v_fn_dc_bd);
  __Pyx_XDECREF(__pyx_v_constraint_dc);
  __Pyx_XDECREF(__pyx_v_v_constraints);
  __Pyx_XDECREF(__pyx_v_b_is_worse);
  __Pyx_DECREF(((PyObject *)__pyx_cur_scope));
  __Pyx_XGIVEREF(__pyx_r);
  __Pyx_TraceReturn(__pyx_r, 0);
  __Pyx_RefNannyFinishContext();
  return __pyx_r;
}
/* … */
  __pyx_tuple__9 = PyTuple_Pack(20, __pyx_n_s_v_channel, __pyx_n_s_nda_interf, __pyx_n_s_d_max_bits, __pyx_n_s_n_helpers, __pyx_n_s_v_opt_ga, __pyx_n_s_d_opt_ga, __pyx_n_s_v_intl_dc, __pyx_n_s_v_intl_ga, __pyx_n_s_d_intl_lambda, __pyx_n_s_v_best, __pyx_n_s_nda_bounds, __pyx_n_s_fn_sum_bd, __pyx_n_s_constraint_sum, __pyx_n_s_fn_objective, __pyx_n_s_opt_result, __pyx_n_s_d_brlx, __pyx_n_s_fn_dc_bd, __pyx_n_s_constraint_dc, __pyx_n_s_v_constraints, __pyx_n_s_b_is_worse); if (unlikely(!__pyx_tuple__9)) __PYX_ERR(0, 37, __pyx_L1_error)
  __Pyx_GOTREF(__pyx_tuple__9);
  __Pyx_GIVEREF(__pyx_tuple__9);
/* … */
  __pyx_t_2 = PyCFunction_NewEx(&__pyx_mdef_9dist_recv_9opt_rates_5opt_dc, NULL, __pyx_n_s_dist_recv_opt_rates); if (unlikely(!__pyx_t_2)) __PYX_ERR(0, 37, __pyx_L1_error)
  __Pyx_GOTREF(__pyx_t_2);
  if (PyDict_SetItem(__pyx_d, __pyx_n_s_opt_dc, __pyx_t_2) < 0) __PYX_ERR(0, 37, __pyx_L1_error)
  __Pyx_DECREF(__pyx_t_2); __pyx_t_2 = 0;
  __pyx_codeobj__4 = (PyObject*)__Pyx_PyCode_New(3, 0, 20, 0, 0, __pyx_empty_bytes, __pyx_empty_tuple, __pyx_empty_tuple, __pyx_tuple__9, __pyx_empty_tuple, __pyx_empty_tuple, __pyx_kp_s_Users_cdchapm2_Dropbox_Personal, __pyx_n_s_opt_dc, 37, __pyx_empty_bytes); if (unlikely(!__pyx_codeobj__4)) __PYX_ERR(0, 37, __pyx_L1_error)
```

```
 038:     """
```

```
 039:     Optimize rates.rate_dc for a given channel, interference, over a sum-bits constraint
```

```
 040:         (barrier relaxation)
```

```
 041:     Input:
```

```
 042:         v_channel = Channel vector h (size N+1 x 1)
```

```
 043:         nda_interf = Interference covariance matrix Sigma (size N+1 x N+1)
```

```
 044:         d_max_bits = Max total helper encoding rates.
```

```
 045:     Output:
```

```
 046:         opt_result = Optimization output:
```

```
 047:                      opt_result.x[0:N] = v_dc_bits
```

```
 048:                      opt_result.x[N:-1] = v_ga_bits
```

```
 049:                      opt_result.x[-1] = d_lambda
```

```
 050:     """
```

```
+051:     n_helpers = len(v_channel)-1
```

```
  __pyx_t_1 = __pyx_cur_scope->__pyx_v_v_channel;
  __Pyx_INCREF(__pyx_t_1);
  __pyx_t_2 = PyObject_Length(__pyx_t_1); if (unlikely(__pyx_t_2 == -1)) __PYX_ERR(0, 51, __pyx_L1_error)
  __Pyx_DECREF(__pyx_t_1); __pyx_t_1 = 0;
  __pyx_t_1 = PyInt_FromSsize_t((__pyx_t_2 - 1)); if (unlikely(!__pyx_t_1)) __PYX_ERR(0, 51, __pyx_L1_error)
  __Pyx_GOTREF(__pyx_t_1);
  __Pyx_GIVEREF(__pyx_t_1);
  __pyx_cur_scope->__pyx_v_n_helpers = __pyx_t_1;
  __pyx_t_1 = 0;
```

```
 052:
```

```
+053:     v_opt_ga = opt_ga(v_channel, nda_interf, d_max_bits).x
```

```
  __pyx_t_3 = __Pyx_GetModuleGlobalName(__pyx_n_s_opt_ga); if (unlikely(!__pyx_t_3)) __PYX_ERR(0, 53, __pyx_L1_error)
  __Pyx_GOTREF(__pyx_t_3);
  __pyx_t_4 = NULL;
  __pyx_t_5 = 0;
  if (CYTHON_UNPACK_METHODS && unlikely(PyMethod_Check(__pyx_t_3))) {
    __pyx_t_4 = PyMethod_GET_SELF(__pyx_t_3);
    if (likely(__pyx_t_4)) {
      PyObject* function = PyMethod_GET_FUNCTION(__pyx_t_3);
      __Pyx_INCREF(__pyx_t_4);
      __Pyx_INCREF(function);
      __Pyx_DECREF_SET(__pyx_t_3, function);
      __pyx_t_5 = 1;
    }
  }
  #if CYTHON_FAST_PYCALL
  if (PyFunction_Check(__pyx_t_3)) {
    PyObject *__pyx_temp[4] = {__pyx_t_4, __pyx_cur_scope->__pyx_v_v_channel, __pyx_cur_scope->__pyx_v_nda_interf, __pyx_cur_scope->__pyx_v_d_max_bits};
    __pyx_t_1 = __Pyx_PyFunction_FastCall(__pyx_t_3, __pyx_temp+1-__pyx_t_5, 3+__pyx_t_5); if (unlikely(!__pyx_t_1)) __PYX_ERR(0, 53, __pyx_L1_error)
    __Pyx_XDECREF(__pyx_t_4); __pyx_t_4 = 0;
    __Pyx_GOTREF(__pyx_t_1);
  } else
  #endif
  #if CYTHON_FAST_PYCCALL
  if (__Pyx_PyFastCFunction_Check(__pyx_t_3)) {
    PyObject *__pyx_temp[4] = {__pyx_t_4, __pyx_cur_scope->__pyx_v_v_channel, __pyx_cur_scope->__pyx_v_nda_interf, __pyx_cur_scope->__pyx_v_d_max_bits};
    __pyx_t_1 = __Pyx_PyCFunction_FastCall(__pyx_t_3, __pyx_temp+1-__pyx_t_5, 3+__pyx_t_5); if (unlikely(!__pyx_t_1)) __PYX_ERR(0, 53, __pyx_L1_error)
    __Pyx_XDECREF(__pyx_t_4); __pyx_t_4 = 0;
    __Pyx_GOTREF(__pyx_t_1);
  } else
  #endif
  {
    __pyx_t_6 = PyTuple_New(3+__pyx_t_5); if (unlikely(!__pyx_t_6)) __PYX_ERR(0, 53, __pyx_L1_error)
    __Pyx_GOTREF(__pyx_t_6);
    if (__pyx_t_4) {
      __Pyx_GIVEREF(__pyx_t_4); PyTuple_SET_ITEM(__pyx_t_6, 0, __pyx_t_4); __pyx_t_4 = NULL;
    }
    __Pyx_INCREF(__pyx_cur_scope->__pyx_v_v_channel);
    __Pyx_GIVEREF(__pyx_cur_scope->__pyx_v_v_channel);
    PyTuple_SET_ITEM(__pyx_t_6, 0+__pyx_t_5, __pyx_cur_scope->__pyx_v_v_channel);
    __Pyx_INCREF(__pyx_cur_scope->__pyx_v_nda_interf);
    __Pyx_GIVEREF(__pyx_cur_scope->__pyx_v_nda_interf);
    PyTuple_SET_ITEM(__pyx_t_6, 1+__pyx_t_5, __pyx_cur_scope->__pyx_v_nda_interf);
    __Pyx_INCREF(__pyx_cur_scope->__pyx_v_d_max_bits);
    __Pyx_GIVEREF(__pyx_cur_scope->__pyx_v_d_max_bits);
    PyTuple_SET_ITEM(__pyx_t_6, 2+__pyx_t_5, __pyx_cur_scope->__pyx_v_d_max_bits);
    __pyx_t_1 = __Pyx_PyObject_Call(__pyx_t_3, __pyx_t_6, NULL); if (unlikely(!__pyx_t_1)) __PYX_ERR(0, 53, __pyx_L1_error)
    __Pyx_GOTREF(__pyx_t_1);
    __Pyx_DECREF(__pyx_t_6); __pyx_t_6 = 0;
  }
  __Pyx_DECREF(__pyx_t_3); __pyx_t_3 = 0;
  __pyx_t_3 = __Pyx_PyObject_GetAttrStr(__pyx_t_1, __pyx_n_s_x); if (unlikely(!__pyx_t_3)) __PYX_ERR(0, 53, __pyx_L1_error)
  __Pyx_GOTREF(__pyx_t_3);
  __Pyx_DECREF(__pyx_t_1); __pyx_t_1 = 0;
  __pyx_v_v_opt_ga = __pyx_t_3;
  __pyx_t_3 = 0;
```

```
+054:     d_opt_ga = rates.rate_ga(v_channel, nda_interf, v_opt_ga)
```

```
  __pyx_t_1 = __Pyx_GetModuleGlobalName(__pyx_n_s_rates); if (unlikely(!__pyx_t_1)) __PYX_ERR(0, 54, __pyx_L1_error)
  __Pyx_GOTREF(__pyx_t_1);
  __pyx_t_6 = __Pyx_PyObject_GetAttrStr(__pyx_t_1, __pyx_n_s_rate_ga); if (unlikely(!__pyx_t_6)) __PYX_ERR(0, 54, __pyx_L1_error)
  __Pyx_GOTREF(__pyx_t_6);
  __Pyx_DECREF(__pyx_t_1); __pyx_t_1 = 0;
  __pyx_t_1 = NULL;
  __pyx_t_5 = 0;
  if (CYTHON_UNPACK_METHODS && unlikely(PyMethod_Check(__pyx_t_6))) {
    __pyx_t_1 = PyMethod_GET_SELF(__pyx_t_6);
    if (likely(__pyx_t_1)) {
      PyObject* function = PyMethod_GET_FUNCTION(__pyx_t_6);
      __Pyx_INCREF(__pyx_t_1);
      __Pyx_INCREF(function);
      __Pyx_DECREF_SET(__pyx_t_6, function);
      __pyx_t_5 = 1;
    }
  }
  #if CYTHON_FAST_PYCALL
  if (PyFunction_Check(__pyx_t_6)) {
    PyObject *__pyx_temp[4] = {__pyx_t_1, __pyx_cur_scope->__pyx_v_v_channel, __pyx_cur_scope->__pyx_v_nda_interf, __pyx_v_v_opt_ga};
    __pyx_t_3 = __Pyx_PyFunction_FastCall(__pyx_t_6, __pyx_temp+1-__pyx_t_5, 3+__pyx_t_5); if (unlikely(!__pyx_t_3)) __PYX_ERR(0, 54, __pyx_L1_error)
    __Pyx_XDECREF(__pyx_t_1); __pyx_t_1 = 0;
    __Pyx_GOTREF(__pyx_t_3);
  } else
  #endif
  #if CYTHON_FAST_PYCCALL
  if (__Pyx_PyFastCFunction_Check(__pyx_t_6)) {
    PyObject *__pyx_temp[4] = {__pyx_t_1, __pyx_cur_scope->__pyx_v_v_channel, __pyx_cur_scope->__pyx_v_nda_interf, __pyx_v_v_opt_ga};
    __pyx_t_3 = __Pyx_PyCFunction_FastCall(__pyx_t_6, __pyx_temp+1-__pyx_t_5, 3+__pyx_t_5); if (unlikely(!__pyx_t_3)) __PYX_ERR(0, 54, __pyx_L1_error)
    __Pyx_XDECREF(__pyx_t_1); __pyx_t_1 = 0;
    __Pyx_GOTREF(__pyx_t_3);
  } else
  #endif
  {
    __pyx_t_4 = PyTuple_New(3+__pyx_t_5); if (unlikely(!__pyx_t_4)) __PYX_ERR(0, 54, __pyx_L1_error)
    __Pyx_GOTREF(__pyx_t_4);
    if (__pyx_t_1) {
      __Pyx_GIVEREF(__pyx_t_1); PyTuple_SET_ITEM(__pyx_t_4, 0, __pyx_t_1); __pyx_t_1 = NULL;
    }
    __Pyx_INCREF(__pyx_cur_scope->__pyx_v_v_channel);
    __Pyx_GIVEREF(__pyx_cur_scope->__pyx_v_v_channel);
    PyTuple_SET_ITEM(__pyx_t_4, 0+__pyx_t_5, __pyx_cur_scope->__pyx_v_v_channel);
    __Pyx_INCREF(__pyx_cur_scope->__pyx_v_nda_interf);
    __Pyx_GIVEREF(__pyx_cur_scope->__pyx_v_nda_interf);
    PyTuple_SET_ITEM(__pyx_t_4, 1+__pyx_t_5, __pyx_cur_scope->__pyx_v_nda_interf);
    __Pyx_INCREF(__pyx_v_v_opt_ga);
    __Pyx_GIVEREF(__pyx_v_v_opt_ga);
    PyTuple_SET_ITEM(__pyx_t_4, 2+__pyx_t_5, __pyx_v_v_opt_ga);
    __pyx_t_3 = __Pyx_PyObject_Call(__pyx_t_6, __pyx_t_4, NULL); if (unlikely(!__pyx_t_3)) __PYX_ERR(0, 54, __pyx_L1_error)
    __Pyx_GOTREF(__pyx_t_3);
    __Pyx_DECREF(__pyx_t_4); __pyx_t_4 = 0;
  }
  __Pyx_DECREF(__pyx_t_6); __pyx_t_6 = 0;
  __pyx_v_d_opt_ga = __pyx_t_3;
  __pyx_t_3 = 0;
```

```
 055:
```

```
+056:     v_intl_dc = np.ones(n_helpers)*d_max_bits/n_helpers
```

```
  __pyx_t_6 = __Pyx_GetModuleGlobalName(__pyx_n_s_np); if (unlikely(!__pyx_t_6)) __PYX_ERR(0, 56, __pyx_L1_error)
  __Pyx_GOTREF(__pyx_t_6);
  __pyx_t_4 = __Pyx_PyObject_GetAttrStr(__pyx_t_6, __pyx_n_s_ones); if (unlikely(!__pyx_t_4)) __PYX_ERR(0, 56, __pyx_L1_error)
  __Pyx_GOTREF(__pyx_t_4);
  __Pyx_DECREF(__pyx_t_6); __pyx_t_6 = 0;
  __pyx_t_6 = NULL;
  if (CYTHON_UNPACK_METHODS && unlikely(PyMethod_Check(__pyx_t_4))) {
    __pyx_t_6 = PyMethod_GET_SELF(__pyx_t_4);
    if (likely(__pyx_t_6)) {
      PyObject* function = PyMethod_GET_FUNCTION(__pyx_t_4);
      __Pyx_INCREF(__pyx_t_6);
      __Pyx_INCREF(function);
      __Pyx_DECREF_SET(__pyx_t_4, function);
    }
  }
  if (!__pyx_t_6) {
    __pyx_t_3 = __Pyx_PyObject_CallOneArg(__pyx_t_4, __pyx_cur_scope->__pyx_v_n_helpers); if (unlikely(!__pyx_t_3)) __PYX_ERR(0, 56, __pyx_L1_error)
    __Pyx_GOTREF(__pyx_t_3);
  } else {
    #if CYTHON_FAST_PYCALL
    if (PyFunction_Check(__pyx_t_4)) {
      PyObject *__pyx_temp[2] = {__pyx_t_6, __pyx_cur_scope->__pyx_v_n_helpers};
      __pyx_t_3 = __Pyx_PyFunction_FastCall(__pyx_t_4, __pyx_temp+1-1, 1+1); if (unlikely(!__pyx_t_3)) __PYX_ERR(0, 56, __pyx_L1_error)
      __Pyx_XDECREF(__pyx_t_6); __pyx_t_6 = 0;
      __Pyx_GOTREF(__pyx_t_3);
    } else
    #endif
    #if CYTHON_FAST_PYCCALL
    if (__Pyx_PyFastCFunction_Check(__pyx_t_4)) {
      PyObject *__pyx_temp[2] = {__pyx_t_6, __pyx_cur_scope->__pyx_v_n_helpers};
      __pyx_t_3 = __Pyx_PyCFunction_FastCall(__pyx_t_4, __pyx_temp+1-1, 1+1); if (unlikely(!__pyx_t_3)) __PYX_ERR(0, 56, __pyx_L1_error)
      __Pyx_XDECREF(__pyx_t_6); __pyx_t_6 = 0;
      __Pyx_GOTREF(__pyx_t_3);
    } else
    #endif
    {
      __pyx_t_1 = PyTuple_New(1+1); if (unlikely(!__pyx_t_1)) __PYX_ERR(0, 56, __pyx_L1_error)
      __Pyx_GOTREF(__pyx_t_1);
      __Pyx_GIVEREF(__pyx_t_6); PyTuple_SET_ITEM(__pyx_t_1, 0, __pyx_t_6); __pyx_t_6 = NULL;
      __Pyx_INCREF(__pyx_cur_scope->__pyx_v_n_helpers);
      __Pyx_GIVEREF(__pyx_cur_scope->__pyx_v_n_helpers);
      PyTuple_SET_ITEM(__pyx_t_1, 0+1, __pyx_cur_scope->__pyx_v_n_helpers);
      __pyx_t_3 = __Pyx_PyObject_Call(__pyx_t_4, __pyx_t_1, NULL); if (unlikely(!__pyx_t_3)) __PYX_ERR(0, 56, __pyx_L1_error)
      __Pyx_GOTREF(__pyx_t_3);
      __Pyx_DECREF(__pyx_t_1); __pyx_t_1 = 0;
    }
  }
  __Pyx_DECREF(__pyx_t_4); __pyx_t_4 = 0;
  __pyx_t_4 = PyNumber_Multiply(__pyx_t_3, __pyx_cur_scope->__pyx_v_d_max_bits); if (unlikely(!__pyx_t_4)) __PYX_ERR(0, 56, __pyx_L1_error)
  __Pyx_GOTREF(__pyx_t_4);
  __Pyx_DECREF(__pyx_t_3); __pyx_t_3 = 0;
  __pyx_t_3 = __Pyx_PyNumber_Divide(__pyx_t_4, __pyx_cur_scope->__pyx_v_n_helpers); if (unlikely(!__pyx_t_3)) __PYX_ERR(0, 56, __pyx_L1_error)
  __Pyx_GOTREF(__pyx_t_3);
  __Pyx_DECREF(__pyx_t_4); __pyx_t_4 = 0;
  __pyx_v_v_intl_dc = __pyx_t_3;
  __pyx_t_3 = 0;
```

```
+057:     v_intl_ga = v_intl_dc
```

```
  __Pyx_INCREF(__pyx_v_v_intl_dc);
  __pyx_v_v_intl_ga = __pyx_v_v_intl_dc;
```

```
+058:     d_intl_lambda = 0
```

```
  __Pyx_INCREF(__pyx_int_0);
  __pyx_v_d_intl_lambda = __pyx_int_0;
```

```
+059:     v_best = np.append([v_intl_dc, v_intl_ga], np.array([d_intl_lambda]))
```

```
  __pyx_t_4 = __Pyx_GetModuleGlobalName(__pyx_n_s_np); if (unlikely(!__pyx_t_4)) __PYX_ERR(0, 59, __pyx_L1_error)
  __Pyx_GOTREF(__pyx_t_4);
  __pyx_t_1 = __Pyx_PyObject_GetAttrStr(__pyx_t_4, __pyx_n_s_append); if (unlikely(!__pyx_t_1)) __PYX_ERR(0, 59, __pyx_L1_error)
  __Pyx_GOTREF(__pyx_t_1);
  __Pyx_DECREF(__pyx_t_4); __pyx_t_4 = 0;
  __pyx_t_4 = PyList_New(2); if (unlikely(!__pyx_t_4)) __PYX_ERR(0, 59, __pyx_L1_error)
  __Pyx_GOTREF(__pyx_t_4);
  __Pyx_INCREF(__pyx_v_v_intl_dc);
  __Pyx_GIVEREF(__pyx_v_v_intl_dc);
  PyList_SET_ITEM(__pyx_t_4, 0, __pyx_v_v_intl_dc);
  __Pyx_INCREF(__pyx_v_v_intl_ga);
  __Pyx_GIVEREF(__pyx_v_v_intl_ga);
  PyList_SET_ITEM(__pyx_t_4, 1, __pyx_v_v_intl_ga);
  __pyx_t_7 = __Pyx_GetModuleGlobalName(__pyx_n_s_np); if (unlikely(!__pyx_t_7)) __PYX_ERR(0, 59, __pyx_L1_error)
  __Pyx_GOTREF(__pyx_t_7);
  __pyx_t_8 = __Pyx_PyObject_GetAttrStr(__pyx_t_7, __pyx_n_s_array); if (unlikely(!__pyx_t_8)) __PYX_ERR(0, 59, __pyx_L1_error)
  __Pyx_GOTREF(__pyx_t_8);
  __Pyx_DECREF(__pyx_t_7); __pyx_t_7 = 0;
  __pyx_t_7 = PyList_New(1); if (unlikely(!__pyx_t_7)) __PYX_ERR(0, 59, __pyx_L1_error)
  __Pyx_GOTREF(__pyx_t_7);
  __Pyx_INCREF(__pyx_v_d_intl_lambda);
  __Pyx_GIVEREF(__pyx_v_d_intl_lambda);
  PyList_SET_ITEM(__pyx_t_7, 0, __pyx_v_d_intl_lambda);
  __pyx_t_9 = NULL;
  if (CYTHON_UNPACK_METHODS && unlikely(PyMethod_Check(__pyx_t_8))) {
    __pyx_t_9 = PyMethod_GET_SELF(__pyx_t_8);
    if (likely(__pyx_t_9)) {
      PyObject* function = PyMethod_GET_FUNCTION(__pyx_t_8);
      __Pyx_INCREF(__pyx_t_9);
      __Pyx_INCREF(function);
      __Pyx_DECREF_SET(__pyx_t_8, function);
    }
  }
  if (!__pyx_t_9) {
    __pyx_t_6 = __Pyx_PyObject_CallOneArg(__pyx_t_8, __pyx_t_7); if (unlikely(!__pyx_t_6)) __PYX_ERR(0, 59, __pyx_L1_error)
    __Pyx_DECREF(__pyx_t_7); __pyx_t_7 = 0;
    __Pyx_GOTREF(__pyx_t_6);
  } else {
    #if CYTHON_FAST_PYCALL
    if (PyFunction_Check(__pyx_t_8)) {
      PyObject *__pyx_temp[2] = {__pyx_t_9, __pyx_t_7};
      __pyx_t_6 = __Pyx_PyFunction_FastCall(__pyx_t_8, __pyx_temp+1-1, 1+1); if (unlikely(!__pyx_t_6)) __PYX_ERR(0, 59, __pyx_L1_error)
      __Pyx_XDECREF(__pyx_t_9); __pyx_t_9 = 0;
      __Pyx_GOTREF(__pyx_t_6);
      __Pyx_DECREF(__pyx_t_7); __pyx_t_7 = 0;
    } else
    #endif
    #if CYTHON_FAST_PYCCALL
    if (__Pyx_PyFastCFunction_Check(__pyx_t_8)) {
      PyObject *__pyx_temp[2] = {__pyx_t_9, __pyx_t_7};
      __pyx_t_6 = __Pyx_PyCFunction_FastCall(__pyx_t_8, __pyx_temp+1-1, 1+1); if (unlikely(!__pyx_t_6)) __PYX_ERR(0, 59, __pyx_L1_error)
      __Pyx_XDECREF(__pyx_t_9); __pyx_t_9 = 0;
      __Pyx_GOTREF(__pyx_t_6);
      __Pyx_DECREF(__pyx_t_7); __pyx_t_7 = 0;
    } else
    #endif
    {
      __pyx_t_10 = PyTuple_New(1+1); if (unlikely(!__pyx_t_10)) __PYX_ERR(0, 59, __pyx_L1_error)
      __Pyx_GOTREF(__pyx_t_10);
      __Pyx_GIVEREF(__pyx_t_9); PyTuple_SET_ITEM(__pyx_t_10, 0, __pyx_t_9); __pyx_t_9 = NULL;
      __Pyx_GIVEREF(__pyx_t_7);
      PyTuple_SET_ITEM(__pyx_t_10, 0+1, __pyx_t_7);
      __pyx_t_7 = 0;
      __pyx_t_6 = __Pyx_PyObject_Call(__pyx_t_8, __pyx_t_10, NULL); if (unlikely(!__pyx_t_6)) __PYX_ERR(0, 59, __pyx_L1_error)
      __Pyx_GOTREF(__pyx_t_6);
      __Pyx_DECREF(__pyx_t_10); __pyx_t_10 = 0;
    }
  }
  __Pyx_DECREF(__pyx_t_8); __pyx_t_8 = 0;
  __pyx_t_8 = NULL;
  __pyx_t_5 = 0;
  if (CYTHON_UNPACK_METHODS && unlikely(PyMethod_Check(__pyx_t_1))) {
    __pyx_t_8 = PyMethod_GET_SELF(__pyx_t_1);
    if (likely(__pyx_t_8)) {
      PyObject* function = PyMethod_GET_FUNCTION(__pyx_t_1);
      __Pyx_INCREF(__pyx_t_8);
      __Pyx_INCREF(function);
      __Pyx_DECREF_SET(__pyx_t_1, function);
      __pyx_t_5 = 1;
    }
  }
  #if CYTHON_FAST_PYCALL
  if (PyFunction_Check(__pyx_t_1)) {
    PyObject *__pyx_temp[3] = {__pyx_t_8, __pyx_t_4, __pyx_t_6};
    __pyx_t_3 = __Pyx_PyFunction_FastCall(__pyx_t_1, __pyx_temp+1-__pyx_t_5, 2+__pyx_t_5); if (unlikely(!__pyx_t_3)) __PYX_ERR(0, 59, __pyx_L1_error)
    __Pyx_XDECREF(__pyx_t_8); __pyx_t_8 = 0;
    __Pyx_GOTREF(__pyx_t_3);
    __Pyx_DECREF(__pyx_t_4); __pyx_t_4 = 0;
    __Pyx_DECREF(__pyx_t_6); __pyx_t_6 = 0;
  } else
  #endif
  #if CYTHON_FAST_PYCCALL
  if (__Pyx_PyFastCFunction_Check(__pyx_t_1)) {
    PyObject *__pyx_temp[3] = {__pyx_t_8, __pyx_t_4, __pyx_t_6};
    __pyx_t_3 = __Pyx_PyCFunction_FastCall(__pyx_t_1, __pyx_temp+1-__pyx_t_5, 2+__pyx_t_5); if (unlikely(!__pyx_t_3)) __PYX_ERR(0, 59, __pyx_L1_error)
    __Pyx_XDECREF(__pyx_t_8); __pyx_t_8 = 0;
    __Pyx_GOTREF(__pyx_t_3);
    __Pyx_DECREF(__pyx_t_4); __pyx_t_4 = 0;
    __Pyx_DECREF(__pyx_t_6); __pyx_t_6 = 0;
  } else
  #endif
  {
    __pyx_t_10 = PyTuple_New(2+__pyx_t_5); if (unlikely(!__pyx_t_10)) __PYX_ERR(0, 59, __pyx_L1_error)
    __Pyx_GOTREF(__pyx_t_10);
    if (__pyx_t_8) {
      __Pyx_GIVEREF(__pyx_t_8); PyTuple_SET_ITEM(__pyx_t_10, 0, __pyx_t_8); __pyx_t_8 = NULL;
    }
    __Pyx_GIVEREF(__pyx_t_4);
    PyTuple_SET_ITEM(__pyx_t_10, 0+__pyx_t_5, __pyx_t_4);
    __Pyx_GIVEREF(__pyx_t_6);
    PyTuple_SET_ITEM(__pyx_t_10, 1+__pyx_t_5, __pyx_t_6);
    __pyx_t_4 = 0;
    __pyx_t_6 = 0;
    __pyx_t_3 = __Pyx_PyObject_Call(__pyx_t_1, __pyx_t_10, NULL); if (unlikely(!__pyx_t_3)) __PYX_ERR(0, 59, __pyx_L1_error)
    __Pyx_GOTREF(__pyx_t_3);
    __Pyx_DECREF(__pyx_t_10); __pyx_t_10 = 0;
  }
  __Pyx_DECREF(__pyx_t_1); __pyx_t_1 = 0;
  __pyx_v_v_best = __pyx_t_3;
  __pyx_t_3 = 0;
```

```
 060:
```

```
+061:     nda_bounds = np.tile([0, d_max_bits], (n_helpers*2+1, 1))
```

```
  __pyx_t_1 = __Pyx_GetModuleGlobalName(__pyx_n_s_np); if (unlikely(!__pyx_t_1)) __PYX_ERR(0, 61, __pyx_L1_error)
  __Pyx_GOTREF(__pyx_t_1);
  __pyx_t_10 = __Pyx_PyObject_GetAttrStr(__pyx_t_1, __pyx_n_s_tile); if (unlikely(!__pyx_t_10)) __PYX_ERR(0, 61, __pyx_L1_error)
  __Pyx_GOTREF(__pyx_t_10);
  __Pyx_DECREF(__pyx_t_1); __pyx_t_1 = 0;
  __pyx_t_1 = PyList_New(2); if (unlikely(!__pyx_t_1)) __PYX_ERR(0, 61, __pyx_L1_error)
  __Pyx_GOTREF(__pyx_t_1);
  __Pyx_INCREF(__pyx_int_0);
  __Pyx_GIVEREF(__pyx_int_0);
  PyList_SET_ITEM(__pyx_t_1, 0, __pyx_int_0);
  __Pyx_INCREF(__pyx_cur_scope->__pyx_v_d_max_bits);
  __Pyx_GIVEREF(__pyx_cur_scope->__pyx_v_d_max_bits);
  PyList_SET_ITEM(__pyx_t_1, 1, __pyx_cur_scope->__pyx_v_d_max_bits);
  __pyx_t_6 = PyNumber_Multiply(__pyx_cur_scope->__pyx_v_n_helpers, __pyx_int_2); if (unlikely(!__pyx_t_6)) __PYX_ERR(0, 61, __pyx_L1_error)
  __Pyx_GOTREF(__pyx_t_6);
  __pyx_t_4 = __Pyx_PyInt_AddObjC(__pyx_t_6, __pyx_int_1, 1, 0); if (unlikely(!__pyx_t_4)) __PYX_ERR(0, 61, __pyx_L1_error)
  __Pyx_GOTREF(__pyx_t_4);
  __Pyx_DECREF(__pyx_t_6); __pyx_t_6 = 0;
  __pyx_t_6 = PyTuple_New(2); if (unlikely(!__pyx_t_6)) __PYX_ERR(0, 61, __pyx_L1_error)
  __Pyx_GOTREF(__pyx_t_6);
  __Pyx_GIVEREF(__pyx_t_4);
  PyTuple_SET_ITEM(__pyx_t_6, 0, __pyx_t_4);
  __Pyx_INCREF(__pyx_int_1);
  __Pyx_GIVEREF(__pyx_int_1);
  PyTuple_SET_ITEM(__pyx_t_6, 1, __pyx_int_1);
  __pyx_t_4 = 0;
  __pyx_t_4 = NULL;
  __pyx_t_5 = 0;
  if (CYTHON_UNPACK_METHODS && unlikely(PyMethod_Check(__pyx_t_10))) {
    __pyx_t_4 = PyMethod_GET_SELF(__pyx_t_10);
    if (likely(__pyx_t_4)) {
      PyObject* function = PyMethod_GET_FUNCTION(__pyx_t_10);
      __Pyx_INCREF(__pyx_t_4);
      __Pyx_INCREF(function);
      __Pyx_DECREF_SET(__pyx_t_10, function);
      __pyx_t_5 = 1;
    }
  }
  #if CYTHON_FAST_PYCALL
  if (PyFunction_Check(__pyx_t_10)) {
    PyObject *__pyx_temp[3] = {__pyx_t_4, __pyx_t_1, __pyx_t_6};
    __pyx_t_3 = __Pyx_PyFunction_FastCall(__pyx_t_10, __pyx_temp+1-__pyx_t_5, 2+__pyx_t_5); if (unlikely(!__pyx_t_3)) __PYX_ERR(0, 61, __pyx_L1_error)
    __Pyx_XDECREF(__pyx_t_4); __pyx_t_4 = 0;
    __Pyx_GOTREF(__pyx_t_3);
    __Pyx_DECREF(__pyx_t_1); __pyx_t_1 = 0;
    __Pyx_DECREF(__pyx_t_6); __pyx_t_6 = 0;
  } else
  #endif
  #if CYTHON_FAST_PYCCALL
  if (__Pyx_PyFastCFunction_Check(__pyx_t_10)) {
    PyObject *__pyx_temp[3] = {__pyx_t_4, __pyx_t_1, __pyx_t_6};
    __pyx_t_3 = __Pyx_PyCFunction_FastCall(__pyx_t_10, __pyx_temp+1-__pyx_t_5, 2+__pyx_t_5); if (unlikely(!__pyx_t_3)) __PYX_ERR(0, 61, __pyx_L1_error)
    __Pyx_XDECREF(__pyx_t_4); __pyx_t_4 = 0;
    __Pyx_GOTREF(__pyx_t_3);
    __Pyx_DECREF(__pyx_t_1); __pyx_t_1 = 0;
    __Pyx_DECREF(__pyx_t_6); __pyx_t_6 = 0;
  } else
  #endif
  {
    __pyx_t_8 = PyTuple_New(2+__pyx_t_5); if (unlikely(!__pyx_t_8)) __PYX_ERR(0, 61, __pyx_L1_error)
    __Pyx_GOTREF(__pyx_t_8);
    if (__pyx_t_4) {
      __Pyx_GIVEREF(__pyx_t_4); PyTuple_SET_ITEM(__pyx_t_8, 0, __pyx_t_4); __pyx_t_4 = NULL;
    }
    __Pyx_GIVEREF(__pyx_t_1);
    PyTuple_SET_ITEM(__pyx_t_8, 0+__pyx_t_5, __pyx_t_1);
    __Pyx_GIVEREF(__pyx_t_6);
    PyTuple_SET_ITEM(__pyx_t_8, 1+__pyx_t_5, __pyx_t_6);
    __pyx_t_1 = 0;
    __pyx_t_6 = 0;
    __pyx_t_3 = __Pyx_PyObject_Call(__pyx_t_10, __pyx_t_8, NULL); if (unlikely(!__pyx_t_3)) __PYX_ERR(0, 61, __pyx_L1_error)
    __Pyx_GOTREF(__pyx_t_3);
    __Pyx_DECREF(__pyx_t_8); __pyx_t_8 = 0;
  }
  __Pyx_DECREF(__pyx_t_10); __pyx_t_10 = 0;
  __pyx_v_nda_bounds = __pyx_t_3;
  __pyx_t_3 = 0;
```

```
 062:
```

```
+063:     fn_sum_bd = lambda v_config: d_max_bits - sum(v_config[0:n_helpers])
```

```
/* Python wrapper */
static PyObject *__pyx_pw_9dist_recv_9opt_rates_6opt_dc_lambda2(PyObject *__pyx_self, PyObject *__pyx_v_v_config); /*proto*/
static PyMethodDef __pyx_mdef_9dist_recv_9opt_rates_6opt_dc_lambda2 = {"lambda2", (PyCFunction)__pyx_pw_9dist_recv_9opt_rates_6opt_dc_lambda2, METH_O, 0};
static PyObject *__pyx_pw_9dist_recv_9opt_rates_6opt_dc_lambda2(PyObject *__pyx_self, PyObject *__pyx_v_v_config) {
  PyObject *__pyx_r = 0;
  __Pyx_RefNannyDeclarations
  __Pyx_RefNannySetupContext("lambda2 (wrapper)", 0);
  __pyx_r = __pyx_lambda_funcdef_lambda2(__pyx_self, ((PyObject *)__pyx_v_v_config));

  /* function exit code */
  __Pyx_RefNannyFinishContext();
  return __pyx_r;
}

static PyObject *__pyx_lambda_funcdef_lambda2(PyObject *__pyx_self, PyObject *__pyx_v_v_config) {
  struct __pyx_obj_9dist_recv_9opt_rates___pyx_scope_struct_1_opt_dc *__pyx_cur_scope;
  struct __pyx_obj_9dist_recv_9opt_rates___pyx_scope_struct_1_opt_dc *__pyx_outer_scope;
  PyObject *__pyx_r = NULL;
  __Pyx_TraceDeclarations
  __Pyx_RefNannyDeclarations
  __Pyx_RefNannySetupContext("lambda2", 0);
  __pyx_outer_scope = (struct __pyx_obj_9dist_recv_9opt_rates___pyx_scope_struct_1_opt_dc *) __Pyx_CyFunction_GetClosure(__pyx_self);
  __pyx_cur_scope = __pyx_outer_scope;
  __Pyx_TraceCall("lambda2", __pyx_f[0], 63, 0, __PYX_ERR(0, 63, __pyx_L1_error));
  __Pyx_XDECREF(__pyx_r);
  if (unlikely(!__pyx_cur_scope->__pyx_v_d_max_bits)) { __Pyx_RaiseClosureNameError("d_max_bits"); __PYX_ERR(0, 63, __pyx_L1_error) }
  if (unlikely(!__pyx_cur_scope->__pyx_v_n_helpers)) { __Pyx_RaiseClosureNameError("n_helpers"); __PYX_ERR(0, 63, __pyx_L1_error) }
  __pyx_t_1 = __Pyx_PyObject_GetSlice(__pyx_v_v_config, 0, 0, NULL, &__pyx_cur_scope->__pyx_v_n_helpers, NULL, 1, 0, 1); if (unlikely(!__pyx_t_1)) __PYX_ERR(0, 63, __pyx_L1_error)
  __Pyx_GOTREF(__pyx_t_1);
  __pyx_t_2 = PyTuple_New(1); if (unlikely(!__pyx_t_2)) __PYX_ERR(0, 63, __pyx_L1_error)
  __Pyx_GOTREF(__pyx_t_2);
  __Pyx_GIVEREF(__pyx_t_1);
  PyTuple_SET_ITEM(__pyx_t_2, 0, __pyx_t_1);
  __pyx_t_1 = 0;
  __pyx_t_1 = __Pyx_PyObject_Call(__pyx_builtin_sum, __pyx_t_2, NULL); if (unlikely(!__pyx_t_1)) __PYX_ERR(0, 63, __pyx_L1_error)
  __Pyx_GOTREF(__pyx_t_1);
  __Pyx_DECREF(__pyx_t_2); __pyx_t_2 = 0;
  __pyx_t_2 = PyNumber_Subtract(__pyx_cur_scope->__pyx_v_d_max_bits, __pyx_t_1); if (unlikely(!__pyx_t_2)) __PYX_ERR(0, 63, __pyx_L1_error)
  __Pyx_GOTREF(__pyx_t_2);
  __Pyx_DECREF(__pyx_t_1); __pyx_t_1 = 0;
  __pyx_r = __pyx_t_2;
  __pyx_t_2 = 0;
  goto __pyx_L0;

  /* function exit code */
  __pyx_L1_error:;
  __Pyx_XDECREF(__pyx_t_1);
  __Pyx_XDECREF(__pyx_t_2);
  __Pyx_AddTraceback("dist_recv.opt_rates.opt_dc.lambda2", __pyx_clineno, __pyx_lineno, __pyx_filename);
  __pyx_r = NULL;
  __pyx_L0:;
  __Pyx_XGIVEREF(__pyx_r);
  __Pyx_TraceReturn(__pyx_r, 0);
  __Pyx_RefNannyFinishContext();
  return __pyx_r;
}
/* … */
  __pyx_t_3 = __Pyx_CyFunction_NewEx(&__pyx_mdef_9dist_recv_9opt_rates_6opt_dc_lambda2, 0, __pyx_n_s_opt_dc_locals_lambda, ((PyObject*)__pyx_cur_scope), __pyx_n_s_dist_recv_opt_rates, __pyx_d, NULL); if (unlikely(!__pyx_t_3)) __PYX_ERR(0, 63, __pyx_L1_error)
  __Pyx_GOTREF(__pyx_t_3);
  __pyx_v_fn_sum_bd = __pyx_t_3;
  __pyx_t_3 = 0;
```

```
+064:     constraint_sum = {'type': 'ineq', 'fun': fn_sum_bd}
```

```
  __pyx_t_3 = PyDict_New(); if (unlikely(!__pyx_t_3)) __PYX_ERR(0, 64, __pyx_L1_error)
  __Pyx_GOTREF(__pyx_t_3);
  if (PyDict_SetItem(__pyx_t_3, __pyx_n_s_type, __pyx_n_s_ineq) < 0) __PYX_ERR(0, 64, __pyx_L1_error)
  if (PyDict_SetItem(__pyx_t_3, __pyx_n_s_fun, __pyx_v_fn_sum_bd) < 0) __PYX_ERR(0, 64, __pyx_L1_error)
  __pyx_v_constraint_sum = ((PyObject*)__pyx_t_3);
  __pyx_t_3 = 0;
```

```
 065:
```

```
+066:     fn_objective = lambda v_config: fn_dc_objective(v_channel, nda_interf, v_config)
```

```
/* Python wrapper */
static PyObject *__pyx_pw_9dist_recv_9opt_rates_6opt_dc_1lambda3(PyObject *__pyx_self, PyObject *__pyx_v_v_config); /*proto*/
static PyMethodDef __pyx_mdef_9dist_recv_9opt_rates_6opt_dc_1lambda3 = {"lambda3", (PyCFunction)__pyx_pw_9dist_recv_9opt_rates_6opt_dc_1lambda3, METH_O, 0};
static PyObject *__pyx_pw_9dist_recv_9opt_rates_6opt_dc_1lambda3(PyObject *__pyx_self, PyObject *__pyx_v_v_config) {
  PyObject *__pyx_r = 0;
  __Pyx_RefNannyDeclarations
  __Pyx_RefNannySetupContext("lambda3 (wrapper)", 0);
  __pyx_r = __pyx_lambda_funcdef_lambda3(__pyx_self, ((PyObject *)__pyx_v_v_config));

  /* function exit code */
  __Pyx_RefNannyFinishContext();
  return __pyx_r;
}

static PyObject *__pyx_lambda_funcdef_lambda3(PyObject *__pyx_self, PyObject *__pyx_v_v_config) {
  struct __pyx_obj_9dist_recv_9opt_rates___pyx_scope_struct_1_opt_dc *__pyx_cur_scope;
  struct __pyx_obj_9dist_recv_9opt_rates___pyx_scope_struct_1_opt_dc *__pyx_outer_scope;
  PyObject *__pyx_r = NULL;
  __Pyx_TraceDeclarations
  __Pyx_RefNannyDeclarations
  __Pyx_RefNannySetupContext("lambda3", 0);
  __pyx_outer_scope = (struct __pyx_obj_9dist_recv_9opt_rates___pyx_scope_struct_1_opt_dc *) __Pyx_CyFunction_GetClosure(__pyx_self);
  __pyx_cur_scope = __pyx_outer_scope;
  __Pyx_TraceCall("lambda3", __pyx_f[0], 66, 0, __PYX_ERR(0, 66, __pyx_L1_error));
  __Pyx_XDECREF(__pyx_r);
  __pyx_t_2 = __Pyx_GetModuleGlobalName(__pyx_n_s_fn_dc_objective); if (unlikely(!__pyx_t_2)) __PYX_ERR(0, 66, __pyx_L1_error)
  __Pyx_GOTREF(__pyx_t_2);
  if (unlikely(!__pyx_cur_scope->__pyx_v_v_channel)) { __Pyx_RaiseClosureNameError("v_channel"); __PYX_ERR(0, 66, __pyx_L1_error) }
  if (unlikely(!__pyx_cur_scope->__pyx_v_nda_interf)) { __Pyx_RaiseClosureNameError("nda_interf"); __PYX_ERR(0, 66, __pyx_L1_error) }
  __pyx_t_3 = NULL;
  __pyx_t_4 = 0;
  if (CYTHON_UNPACK_METHODS && unlikely(PyMethod_Check(__pyx_t_2))) {
    __pyx_t_3 = PyMethod_GET_SELF(__pyx_t_2);
    if (likely(__pyx_t_3)) {
      PyObject* function = PyMethod_GET_FUNCTION(__pyx_t_2);
      __Pyx_INCREF(__pyx_t_3);
      __Pyx_INCREF(function);
      __Pyx_DECREF_SET(__pyx_t_2, function);
      __pyx_t_4 = 1;
    }
  }
  #if CYTHON_FAST_PYCALL
  if (PyFunction_Check(__pyx_t_2)) {
    PyObject *__pyx_temp[4] = {__pyx_t_3, __pyx_cur_scope->__pyx_v_v_channel, __pyx_cur_scope->__pyx_v_nda_interf, __pyx_v_v_config};
    __pyx_t_1 = __Pyx_PyFunction_FastCall(__pyx_t_2, __pyx_temp+1-__pyx_t_4, 3+__pyx_t_4); if (unlikely(!__pyx_t_1)) __PYX_ERR(0, 66, __pyx_L1_error)
    __Pyx_XDECREF(__pyx_t_3); __pyx_t_3 = 0;
    __Pyx_GOTREF(__pyx_t_1);
  } else
  #endif
  #if CYTHON_FAST_PYCCALL
  if (__Pyx_PyFastCFunction_Check(__pyx_t_2)) {
    PyObject *__pyx_temp[4] = {__pyx_t_3, __pyx_cur_scope->__pyx_v_v_channel, __pyx_cur_scope->__pyx_v_nda_interf, __pyx_v_v_config};
    __pyx_t_1 = __Pyx_PyCFunction_FastCall(__pyx_t_2, __pyx_temp+1-__pyx_t_4, 3+__pyx_t_4); if (unlikely(!__pyx_t_1)) __PYX_ERR(0, 66, __pyx_L1_error)
    __Pyx_XDECREF(__pyx_t_3); __pyx_t_3 = 0;
    __Pyx_GOTREF(__pyx_t_1);
  } else
  #endif
  {
    __pyx_t_5 = PyTuple_New(3+__pyx_t_4); if (unlikely(!__pyx_t_5)) __PYX_ERR(0, 66, __pyx_L1_error)
    __Pyx_GOTREF(__pyx_t_5);
    if (__pyx_t_3) {
      __Pyx_GIVEREF(__pyx_t_3); PyTuple_SET_ITEM(__pyx_t_5, 0, __pyx_t_3); __pyx_t_3 = NULL;
    }
    __Pyx_INCREF(__pyx_cur_scope->__pyx_v_v_channel);
    __Pyx_GIVEREF(__pyx_cur_scope->__pyx_v_v_channel);
    PyTuple_SET_ITEM(__pyx_t_5, 0+__pyx_t_4, __pyx_cur_scope->__pyx_v_v_channel);
    __Pyx_INCREF(__pyx_cur_scope->__pyx_v_nda_interf);
    __Pyx_GIVEREF(__pyx_cur_scope->__pyx_v_nda_interf);
    PyTuple_SET_ITEM(__pyx_t_5, 1+__pyx_t_4, __pyx_cur_scope->__pyx_v_nda_interf);
    __Pyx_INCREF(__pyx_v_v_config);
    __Pyx_GIVEREF(__pyx_v_v_config);
    PyTuple_SET_ITEM(__pyx_t_5, 2+__pyx_t_4, __pyx_v_v_config);
    __pyx_t_1 = __Pyx_PyObject_Call(__pyx_t_2, __pyx_t_5, NULL); if (unlikely(!__pyx_t_1)) __PYX_ERR(0, 66, __pyx_L1_error)
    __Pyx_GOTREF(__pyx_t_1);
    __Pyx_DECREF(__pyx_t_5); __pyx_t_5 = 0;
  }
  __Pyx_DECREF(__pyx_t_2); __pyx_t_2 = 0;
  __pyx_r = __pyx_t_1;
  __pyx_t_1 = 0;
  goto __pyx_L0;

  /* function exit code */
  __pyx_L1_error:;
  __Pyx_XDECREF(__pyx_t_1);
  __Pyx_XDECREF(__pyx_t_2);
  __Pyx_XDECREF(__pyx_t_3);
  __Pyx_XDECREF(__pyx_t_5);
  __Pyx_AddTraceback("dist_recv.opt_rates.opt_dc.lambda3", __pyx_clineno, __pyx_lineno, __pyx_filename);
  __pyx_r = NULL;
  __pyx_L0:;
  __Pyx_XGIVEREF(__pyx_r);
  __Pyx_TraceReturn(__pyx_r, 0);
  __Pyx_RefNannyFinishContext();
  return __pyx_r;
}
/* … */
  __pyx_t_3 = __Pyx_CyFunction_NewEx(&__pyx_mdef_9dist_recv_9opt_rates_6opt_dc_1lambda3, 0, __pyx_n_s_opt_dc_locals_lambda, ((PyObject*)__pyx_cur_scope), __pyx_n_s_dist_recv_opt_rates, __pyx_d, NULL); if (unlikely(!__pyx_t_3)) __PYX_ERR(0, 66, __pyx_L1_error)
  __Pyx_GOTREF(__pyx_t_3);
  __pyx_v_fn_objective = __pyx_t_3;
  __pyx_t_3 = 0;
```

```
 067:
```

```
+068:     opt_result = None
```

```
  __Pyx_INCREF(Py_None);
  __pyx_v_opt_result = Py_None;
```

```
+069:     d_brlx = -2
```

```
  __Pyx_INCREF(__pyx_int_neg_2);
  __Pyx_GIVEREF(__pyx_int_neg_2);
  __pyx_cur_scope->__pyx_v_d_brlx = __pyx_int_neg_2;
```

```
+070:     while True:
```

```
  while (1) {
```

```
+071:         fn_dc_bd = lambda v_config: -max(checkdc.checkdc_cts(v_channel,
```

```
/* Python wrapper */
static PyObject *__pyx_pw_9dist_recv_9opt_rates_6opt_dc_2lambda4(PyObject *__pyx_self, PyObject *__pyx_v_v_config); /*proto*/
static PyMethodDef __pyx_mdef_9dist_recv_9opt_rates_6opt_dc_2lambda4 = {"lambda4", (PyCFunction)__pyx_pw_9dist_recv_9opt_rates_6opt_dc_2lambda4, METH_O, 0};
static PyObject *__pyx_pw_9dist_recv_9opt_rates_6opt_dc_2lambda4(PyObject *__pyx_self, PyObject *__pyx_v_v_config) {
  PyObject *__pyx_r = 0;
  __Pyx_RefNannyDeclarations
  __Pyx_RefNannySetupContext("lambda4 (wrapper)", 0);
  __pyx_r = __pyx_lambda_funcdef_lambda4(__pyx_self, ((PyObject *)__pyx_v_v_config));

  /* function exit code */
  __Pyx_RefNannyFinishContext();
  return __pyx_r;
}

static PyObject *__pyx_lambda_funcdef_lambda4(PyObject *__pyx_self, PyObject *__pyx_v_v_config) {
  struct __pyx_obj_9dist_recv_9opt_rates___pyx_scope_struct_1_opt_dc *__pyx_cur_scope;
  struct __pyx_obj_9dist_recv_9opt_rates___pyx_scope_struct_1_opt_dc *__pyx_outer_scope;
  PyObject *__pyx_r = NULL;
  __Pyx_TraceDeclarations
  __Pyx_RefNannyDeclarations
  __Pyx_RefNannySetupContext("lambda4", 0);
  __pyx_outer_scope = (struct __pyx_obj_9dist_recv_9opt_rates___pyx_scope_struct_1_opt_dc *) __Pyx_CyFunction_GetClosure(__pyx_self);
  __pyx_cur_scope = __pyx_outer_scope;
  __Pyx_TraceCall("lambda4", __pyx_f[0], 71, 0, __PYX_ERR(0, 71, __pyx_L1_error));
  __Pyx_XDECREF(__pyx_r);
  __pyx_t_1 = __Pyx_GetModuleGlobalName(__pyx_n_s_checkdc); if (unlikely(!__pyx_t_1)) __PYX_ERR(0, 71, __pyx_L1_error)
  __Pyx_GOTREF(__pyx_t_1);
  __pyx_t_2 = __Pyx_PyObject_GetAttrStr(__pyx_t_1, __pyx_n_s_checkdc_cts); if (unlikely(!__pyx_t_2)) __PYX_ERR(0, 71, __pyx_L1_error)
  __Pyx_GOTREF(__pyx_t_2);
  __Pyx_DECREF(__pyx_t_1); __pyx_t_1 = 0;
  if (unlikely(!__pyx_cur_scope->__pyx_v_v_channel)) { __Pyx_RaiseClosureNameError("v_channel"); __PYX_ERR(0, 71, __pyx_L1_error) }
/* … */
  __pyx_t_1 = PyTuple_New(2); if (unlikely(!__pyx_t_1)) __PYX_ERR(0, 71, __pyx_L1_error)
  __Pyx_GOTREF(__pyx_t_1);
  __Pyx_INCREF(__pyx_cur_scope->__pyx_v_v_channel);
  __Pyx_GIVEREF(__pyx_cur_scope->__pyx_v_v_channel);
  PyTuple_SET_ITEM(__pyx_t_1, 0, __pyx_cur_scope->__pyx_v_v_channel);
  __Pyx_INCREF(__pyx_cur_scope->__pyx_v_nda_interf);
  __Pyx_GIVEREF(__pyx_cur_scope->__pyx_v_nda_interf);
  PyTuple_SET_ITEM(__pyx_t_1, 1, __pyx_cur_scope->__pyx_v_nda_interf);
/* … */
  __pyx_t_4 = __Pyx_PyObject_Call(__pyx_t_2, __pyx_t_1, __pyx_t_3); if (unlikely(!__pyx_t_4)) __PYX_ERR(0, 71, __pyx_L1_error)
  __Pyx_GOTREF(__pyx_t_4);
  __Pyx_DECREF(__pyx_t_2); __pyx_t_2 = 0;
  __Pyx_DECREF(__pyx_t_1); __pyx_t_1 = 0;
  __Pyx_DECREF(__pyx_t_3); __pyx_t_3 = 0;
/* … */
  __pyx_t_4 = PyTuple_New(1); if (unlikely(!__pyx_t_4)) __PYX_ERR(0, 71, __pyx_L1_error)
  __Pyx_GOTREF(__pyx_t_4);
  __Pyx_GIVEREF(__pyx_t_3);
  PyTuple_SET_ITEM(__pyx_t_4, 0, __pyx_t_3);
  __pyx_t_3 = 0;
  __pyx_t_3 = __Pyx_PyObject_Call(__pyx_builtin_max, __pyx_t_4, NULL); if (unlikely(!__pyx_t_3)) __PYX_ERR(0, 71, __pyx_L1_error)
  __Pyx_GOTREF(__pyx_t_3);
  __Pyx_DECREF(__pyx_t_4); __pyx_t_4 = 0;
  __pyx_t_4 = PyNumber_Negative(__pyx_t_3); if (unlikely(!__pyx_t_4)) __PYX_ERR(0, 71, __pyx_L1_error)
  __Pyx_GOTREF(__pyx_t_4);
  __Pyx_DECREF(__pyx_t_3); __pyx_t_3 = 0;
  __pyx_r = __pyx_t_4;
  __pyx_t_4 = 0;
  goto __pyx_L0;

  /* function exit code */
  __pyx_L1_error:;
  __Pyx_XDECREF(__pyx_t_1);
  __Pyx_XDECREF(__pyx_t_2);
  __Pyx_XDECREF(__pyx_t_3);
  __Pyx_XDECREF(__pyx_t_4);
  __Pyx_AddTraceback("dist_recv.opt_rates.opt_dc.lambda4", __pyx_clineno, __pyx_lineno, __pyx_filename);
  __pyx_r = NULL;
  __pyx_L0:;
  __Pyx_XGIVEREF(__pyx_r);
  __Pyx_TraceReturn(__pyx_r, 0);
  __Pyx_RefNannyFinishContext();
  return __pyx_r;
}
/* … */
    __pyx_t_3 = __Pyx_CyFunction_NewEx(&__pyx_mdef_9dist_recv_9opt_rates_6opt_dc_2lambda4, 0, __pyx_n_s_opt_dc_locals_lambda, ((PyObject*)__pyx_cur_scope), __pyx_n_s_dist_recv_opt_rates, __pyx_d, NULL); if (unlikely(!__pyx_t_3)) __PYX_ERR(0, 71, __pyx_L1_error)
    __Pyx_GOTREF(__pyx_t_3);
    __Pyx_XDECREF_SET(__pyx_v_fn_dc_bd, __pyx_t_3);
    __pyx_t_3 = 0;
```

```
+072:                                                              nda_interf,
```

```
  if (unlikely(!__pyx_cur_scope->__pyx_v_nda_interf)) { __Pyx_RaiseClosureNameError("nda_interf"); __PYX_ERR(0, 72, __pyx_L1_error) }
```

```
+073:                                                              v_dc_bits=v_config[0:n_helpers],
```

```
  __pyx_t_3 = PyDict_New(); if (unlikely(!__pyx_t_3)) __PYX_ERR(0, 73, __pyx_L1_error)
  __Pyx_GOTREF(__pyx_t_3);
  if (unlikely(!__pyx_cur_scope->__pyx_v_n_helpers)) { __Pyx_RaiseClosureNameError("n_helpers"); __PYX_ERR(0, 73, __pyx_L1_error) }
  __pyx_t_4 = __Pyx_PyObject_GetSlice(__pyx_v_v_config, 0, 0, NULL, &__pyx_cur_scope->__pyx_v_n_helpers, NULL, 1, 0, 1); if (unlikely(!__pyx_t_4)) __PYX_ERR(0, 73, __pyx_L1_error)
  __Pyx_GOTREF(__pyx_t_4);
  if (PyDict_SetItem(__pyx_t_3, __pyx_n_s_v_dc_bits, __pyx_t_4) < 0) __PYX_ERR(0, 73, __pyx_L1_error)
  __Pyx_DECREF(__pyx_t_4); __pyx_t_4 = 0;
```

```
+074:                                                              v_ga_bits=v_config[n_helpers:-1],
```

```
  if (unlikely(!__pyx_cur_scope->__pyx_v_n_helpers)) { __Pyx_RaiseClosureNameError("n_helpers"); __PYX_ERR(0, 74, __pyx_L1_error) }
  __pyx_t_4 = __Pyx_PyObject_GetSlice(__pyx_v_v_config, 0, -1L, &__pyx_cur_scope->__pyx_v_n_helpers, NULL, NULL, 0, 1, 1); if (unlikely(!__pyx_t_4)) __PYX_ERR(0, 74, __pyx_L1_error)
  __Pyx_GOTREF(__pyx_t_4);
  if (PyDict_SetItem(__pyx_t_3, __pyx_n_s_v_ga_bits, __pyx_t_4) < 0) __PYX_ERR(0, 73, __pyx_L1_error)
  __Pyx_DECREF(__pyx_t_4); __pyx_t_4 = 0;
```

```
+075:                                                              d_lambda=v_config[-1])-d_brlx)
```

```
  __pyx_t_4 = __Pyx_GetItemInt(__pyx_v_v_config, -1L, long, 1, __Pyx_PyInt_From_long, 0, 1, 1); if (unlikely(!__pyx_t_4)) __PYX_ERR(0, 75, __pyx_L1_error)
  __Pyx_GOTREF(__pyx_t_4);
  if (PyDict_SetItem(__pyx_t_3, __pyx_n_s_d_lambda, __pyx_t_4) < 0) __PYX_ERR(0, 73, __pyx_L1_error)
  __Pyx_DECREF(__pyx_t_4); __pyx_t_4 = 0;
/* … */
  if (unlikely(!__pyx_cur_scope->__pyx_v_d_brlx)) { __Pyx_RaiseClosureNameError("d_brlx"); __PYX_ERR(0, 75, __pyx_L1_error) }
  __pyx_t_3 = PyNumber_Subtract(__pyx_t_4, __pyx_cur_scope->__pyx_v_d_brlx); if (unlikely(!__pyx_t_3)) __PYX_ERR(0, 75, __pyx_L1_error)
  __Pyx_GOTREF(__pyx_t_3);
  __Pyx_DECREF(__pyx_t_4); __pyx_t_4 = 0;
```

```
+076:         constraint_dc = {'type': 'ineq', 'fun': fn_dc_bd}
```

```
    __pyx_t_3 = PyDict_New(); if (unlikely(!__pyx_t_3)) __PYX_ERR(0, 76, __pyx_L1_error)
    __Pyx_GOTREF(__pyx_t_3);
    if (PyDict_SetItem(__pyx_t_3, __pyx_n_s_type, __pyx_n_s_ineq) < 0) __PYX_ERR(0, 76, __pyx_L1_error)
    if (PyDict_SetItem(__pyx_t_3, __pyx_n_s_fun, __pyx_v_fn_dc_bd) < 0) __PYX_ERR(0, 76, __pyx_L1_error)
    __Pyx_XDECREF_SET(__pyx_v_constraint_dc, ((PyObject*)__pyx_t_3));
    __pyx_t_3 = 0;
```

```
+077:         v_constraints = [constraint_sum, constraint_dc]
```

```
    __pyx_t_3 = PyList_New(2); if (unlikely(!__pyx_t_3)) __PYX_ERR(0, 77, __pyx_L1_error)
    __Pyx_GOTREF(__pyx_t_3);
    __Pyx_INCREF(__pyx_v_constraint_sum);
    __Pyx_GIVEREF(__pyx_v_constraint_sum);
    PyList_SET_ITEM(__pyx_t_3, 0, __pyx_v_constraint_sum);
    __Pyx_INCREF(__pyx_v_constraint_dc);
    __Pyx_GIVEREF(__pyx_v_constraint_dc);
    PyList_SET_ITEM(__pyx_t_3, 1, __pyx_v_constraint_dc);
    __Pyx_XDECREF_SET(__pyx_v_v_constraints, ((PyObject*)__pyx_t_3));
    __pyx_t_3 = 0;
```

```
 078:
```

```
+079:         opt_result = optimize.minimize(fn_objective,
```

```
    __pyx_t_3 = __Pyx_GetModuleGlobalName(__pyx_n_s_optimize); if (unlikely(!__pyx_t_3)) __PYX_ERR(0, 79, __pyx_L1_error)
    __Pyx_GOTREF(__pyx_t_3);
    __pyx_t_10 = __Pyx_PyObject_GetAttrStr(__pyx_t_3, __pyx_n_s_minimize); if (unlikely(!__pyx_t_10)) __PYX_ERR(0, 79, __pyx_L1_error)
    __Pyx_GOTREF(__pyx_t_10);
    __Pyx_DECREF(__pyx_t_3); __pyx_t_3 = 0;
/* … */
    __pyx_t_3 = PyTuple_New(2); if (unlikely(!__pyx_t_3)) __PYX_ERR(0, 79, __pyx_L1_error)
    __Pyx_GOTREF(__pyx_t_3);
    __Pyx_INCREF(__pyx_v_fn_objective);
    __Pyx_GIVEREF(__pyx_v_fn_objective);
    PyTuple_SET_ITEM(__pyx_t_3, 0, __pyx_v_fn_objective);
    __Pyx_INCREF(__pyx_v_v_best);
    __Pyx_GIVEREF(__pyx_v_v_best);
    PyTuple_SET_ITEM(__pyx_t_3, 1, __pyx_v_v_best);
/* … */
    __pyx_t_6 = __Pyx_PyObject_Call(__pyx_t_10, __pyx_t_3, __pyx_t_8); if (unlikely(!__pyx_t_6)) __PYX_ERR(0, 79, __pyx_L1_error)
    __Pyx_GOTREF(__pyx_t_6);
    __Pyx_DECREF(__pyx_t_10); __pyx_t_10 = 0;
    __Pyx_DECREF(__pyx_t_3); __pyx_t_3 = 0;
    __Pyx_DECREF(__pyx_t_8); __pyx_t_8 = 0;
    __Pyx_DECREF_SET(__pyx_v_opt_result, __pyx_t_6);
    __pyx_t_6 = 0;
```

```
 080:                                        v_best,
```

```
+081:                                        bounds=nda_bounds,
```

```
    __pyx_t_8 = PyDict_New(); if (unlikely(!__pyx_t_8)) __PYX_ERR(0, 81, __pyx_L1_error)
    __Pyx_GOTREF(__pyx_t_8);
    if (PyDict_SetItem(__pyx_t_8, __pyx_n_s_bounds, __pyx_v_nda_bounds) < 0) __PYX_ERR(0, 81, __pyx_L1_error)
```

```
+082:                                        constraints=v_constraints)
```

```
    if (PyDict_SetItem(__pyx_t_8, __pyx_n_s_constraints, __pyx_v_v_constraints) < 0) __PYX_ERR(0, 81, __pyx_L1_error)
```

```
 083:
```

```
 084:         # Only update the initial guess if it's better than GA
```

```
+085:         b_is_worse = rate_opt_dc(v_channel, nda_interf, opt_result) < d_opt_ga
```

```
    __pyx_t_8 = __Pyx_GetModuleGlobalName(__pyx_n_s_rate_opt_dc); if (unlikely(!__pyx_t_8)) __PYX_ERR(0, 85, __pyx_L1_error)
    __Pyx_GOTREF(__pyx_t_8);
    __pyx_t_3 = NULL;
    __pyx_t_5 = 0;
    if (CYTHON_UNPACK_METHODS && unlikely(PyMethod_Check(__pyx_t_8))) {
      __pyx_t_3 = PyMethod_GET_SELF(__pyx_t_8);
      if (likely(__pyx_t_3)) {
        PyObject* function = PyMethod_GET_FUNCTION(__pyx_t_8);
        __Pyx_INCREF(__pyx_t_3);
        __Pyx_INCREF(function);
        __Pyx_DECREF_SET(__pyx_t_8, function);
        __pyx_t_5 = 1;
      }
    }
    #if CYTHON_FAST_PYCALL
    if (PyFunction_Check(__pyx_t_8)) {
      PyObject *__pyx_temp[4] = {__pyx_t_3, __pyx_cur_scope->__pyx_v_v_channel, __pyx_cur_scope->__pyx_v_nda_interf, __pyx_v_opt_result};
      __pyx_t_6 = __Pyx_PyFunction_FastCall(__pyx_t_8, __pyx_temp+1-__pyx_t_5, 3+__pyx_t_5); if (unlikely(!__pyx_t_6)) __PYX_ERR(0, 85, __pyx_L1_error)
      __Pyx_XDECREF(__pyx_t_3); __pyx_t_3 = 0;
      __Pyx_GOTREF(__pyx_t_6);
    } else
    #endif
    #if CYTHON_FAST_PYCCALL
    if (__Pyx_PyFastCFunction_Check(__pyx_t_8)) {
      PyObject *__pyx_temp[4] = {__pyx_t_3, __pyx_cur_scope->__pyx_v_v_channel, __pyx_cur_scope->__pyx_v_nda_interf, __pyx_v_opt_result};
      __pyx_t_6 = __Pyx_PyCFunction_FastCall(__pyx_t_8, __pyx_temp+1-__pyx_t_5, 3+__pyx_t_5); if (unlikely(!__pyx_t_6)) __PYX_ERR(0, 85, __pyx_L1_error)
      __Pyx_XDECREF(__pyx_t_3); __pyx_t_3 = 0;
      __Pyx_GOTREF(__pyx_t_6);
    } else
    #endif
    {
      __pyx_t_10 = PyTuple_New(3+__pyx_t_5); if (unlikely(!__pyx_t_10)) __PYX_ERR(0, 85, __pyx_L1_error)
      __Pyx_GOTREF(__pyx_t_10);
      if (__pyx_t_3) {
        __Pyx_GIVEREF(__pyx_t_3); PyTuple_SET_ITEM(__pyx_t_10, 0, __pyx_t_3); __pyx_t_3 = NULL;
      }
      __Pyx_INCREF(__pyx_cur_scope->__pyx_v_v_channel);
      __Pyx_GIVEREF(__pyx_cur_scope->__pyx_v_v_channel);
      PyTuple_SET_ITEM(__pyx_t_10, 0+__pyx_t_5, __pyx_cur_scope->__pyx_v_v_channel);
      __Pyx_INCREF(__pyx_cur_scope->__pyx_v_nda_interf);
      __Pyx_GIVEREF(__pyx_cur_scope->__pyx_v_nda_interf);
      PyTuple_SET_ITEM(__pyx_t_10, 1+__pyx_t_5, __pyx_cur_scope->__pyx_v_nda_interf);
      __Pyx_INCREF(__pyx_v_opt_result);
      __Pyx_GIVEREF(__pyx_v_opt_result);
      PyTuple_SET_ITEM(__pyx_t_10, 2+__pyx_t_5, __pyx_v_opt_result);
      __pyx_t_6 = __Pyx_PyObject_Call(__pyx_t_8, __pyx_t_10, NULL); if (unlikely(!__pyx_t_6)) __PYX_ERR(0, 85, __pyx_L1_error)
      __Pyx_GOTREF(__pyx_t_6);
      __Pyx_DECREF(__pyx_t_10); __pyx_t_10 = 0;
    }
    __Pyx_DECREF(__pyx_t_8); __pyx_t_8 = 0;
    __pyx_t_8 = PyObject_RichCompare(__pyx_t_6, __pyx_v_d_opt_ga, Py_LT); __Pyx_XGOTREF(__pyx_t_8); if (unlikely(!__pyx_t_8)) __PYX_ERR(0, 85, __pyx_L1_error)
    __Pyx_DECREF(__pyx_t_6); __pyx_t_6 = 0;
    __Pyx_XDECREF_SET(__pyx_v_b_is_worse, __pyx_t_8);
    __pyx_t_8 = 0;
```

```
+086:         if (sum(np.isnan(opt_result.x)) > 0) | (b_is_worse & (d_brlx >= -0.05)):
```

```
    __pyx_t_6 = __Pyx_GetModuleGlobalName(__pyx_n_s_np); if (unlikely(!__pyx_t_6)) __PYX_ERR(0, 86, __pyx_L1_error)
    __Pyx_GOTREF(__pyx_t_6);
    __pyx_t_10 = __Pyx_PyObject_GetAttrStr(__pyx_t_6, __pyx_n_s_isnan); if (unlikely(!__pyx_t_10)) __PYX_ERR(0, 86, __pyx_L1_error)
    __Pyx_GOTREF(__pyx_t_10);
    __Pyx_DECREF(__pyx_t_6); __pyx_t_6 = 0;
    __pyx_t_6 = __Pyx_PyObject_GetAttrStr(__pyx_v_opt_result, __pyx_n_s_x); if (unlikely(!__pyx_t_6)) __PYX_ERR(0, 86, __pyx_L1_error)
    __Pyx_GOTREF(__pyx_t_6);
    __pyx_t_3 = NULL;
    if (CYTHON_UNPACK_METHODS && unlikely(PyMethod_Check(__pyx_t_10))) {
      __pyx_t_3 = PyMethod_GET_SELF(__pyx_t_10);
      if (likely(__pyx_t_3)) {
        PyObject* function = PyMethod_GET_FUNCTION(__pyx_t_10);
        __Pyx_INCREF(__pyx_t_3);
        __Pyx_INCREF(function);
        __Pyx_DECREF_SET(__pyx_t_10, function);
      }
    }
    if (!__pyx_t_3) {
      __pyx_t_8 = __Pyx_PyObject_CallOneArg(__pyx_t_10, __pyx_t_6); if (unlikely(!__pyx_t_8)) __PYX_ERR(0, 86, __pyx_L1_error)
      __Pyx_DECREF(__pyx_t_6); __pyx_t_6 = 0;
      __Pyx_GOTREF(__pyx_t_8);
    } else {
      #if CYTHON_FAST_PYCALL
      if (PyFunction_Check(__pyx_t_10)) {
        PyObject *__pyx_temp[2] = {__pyx_t_3, __pyx_t_6};
        __pyx_t_8 = __Pyx_PyFunction_FastCall(__pyx_t_10, __pyx_temp+1-1, 1+1); if (unlikely(!__pyx_t_8)) __PYX_ERR(0, 86, __pyx_L1_error)
        __Pyx_XDECREF(__pyx_t_3); __pyx_t_3 = 0;
        __Pyx_GOTREF(__pyx_t_8);
        __Pyx_DECREF(__pyx_t_6); __pyx_t_6 = 0;
      } else
      #endif
      #if CYTHON_FAST_PYCCALL
      if (__Pyx_PyFastCFunction_Check(__pyx_t_10)) {
        PyObject *__pyx_temp[2] = {__pyx_t_3, __pyx_t_6};
        __pyx_t_8 = __Pyx_PyCFunction_FastCall(__pyx_t_10, __pyx_temp+1-1, 1+1); if (unlikely(!__pyx_t_8)) __PYX_ERR(0, 86, __pyx_L1_error)
        __Pyx_XDECREF(__pyx_t_3); __pyx_t_3 = 0;
        __Pyx_GOTREF(__pyx_t_8);
        __Pyx_DECREF(__pyx_t_6); __pyx_t_6 = 0;
      } else
      #endif
      {
        __pyx_t_1 = PyTuple_New(1+1); if (unlikely(!__pyx_t_1)) __PYX_ERR(0, 86, __pyx_L1_error)
        __Pyx_GOTREF(__pyx_t_1);
        __Pyx_GIVEREF(__pyx_t_3); PyTuple_SET_ITEM(__pyx_t_1, 0, __pyx_t_3); __pyx_t_3 = NULL;
        __Pyx_GIVEREF(__pyx_t_6);
        PyTuple_SET_ITEM(__pyx_t_1, 0+1, __pyx_t_6);
        __pyx_t_6 = 0;
        __pyx_t_8 = __Pyx_PyObject_Call(__pyx_t_10, __pyx_t_1, NULL); if (unlikely(!__pyx_t_8)) __PYX_ERR(0, 86, __pyx_L1_error)
        __Pyx_GOTREF(__pyx_t_8);
        __Pyx_DECREF(__pyx_t_1); __pyx_t_1 = 0;
      }
    }
    __Pyx_DECREF(__pyx_t_10); __pyx_t_10 = 0;
    __pyx_t_10 = PyTuple_New(1); if (unlikely(!__pyx_t_10)) __PYX_ERR(0, 86, __pyx_L1_error)
    __Pyx_GOTREF(__pyx_t_10);
    __Pyx_GIVEREF(__pyx_t_8);
    PyTuple_SET_ITEM(__pyx_t_10, 0, __pyx_t_8);
    __pyx_t_8 = 0;
    __pyx_t_8 = __Pyx_PyObject_Call(__pyx_builtin_sum, __pyx_t_10, NULL); if (unlikely(!__pyx_t_8)) __PYX_ERR(0, 86, __pyx_L1_error)
    __Pyx_GOTREF(__pyx_t_8);
    __Pyx_DECREF(__pyx_t_10); __pyx_t_10 = 0;
    __pyx_t_10 = PyObject_RichCompare(__pyx_t_8, __pyx_int_0, Py_GT); __Pyx_XGOTREF(__pyx_t_10); if (unlikely(!__pyx_t_10)) __PYX_ERR(0, 86, __pyx_L1_error)
    __Pyx_DECREF(__pyx_t_8); __pyx_t_8 = 0;
    __pyx_t_8 = PyObject_RichCompare(__pyx_cur_scope->__pyx_v_d_brlx, __pyx_float_neg_0_05, Py_GE); __Pyx_XGOTREF(__pyx_t_8); if (unlikely(!__pyx_t_8)) __PYX_ERR(0, 86, __pyx_L1_error)
    __pyx_t_1 = PyNumber_And(__pyx_v_b_is_worse, __pyx_t_8); if (unlikely(!__pyx_t_1)) __PYX_ERR(0, 86, __pyx_L1_error)
    __Pyx_GOTREF(__pyx_t_1);
    __Pyx_DECREF(__pyx_t_8); __pyx_t_8 = 0;
    __pyx_t_8 = PyNumber_Or(__pyx_t_10, __pyx_t_1); if (unlikely(!__pyx_t_8)) __PYX_ERR(0, 86, __pyx_L1_error)
    __Pyx_GOTREF(__pyx_t_8);
    __Pyx_DECREF(__pyx_t_10); __pyx_t_10 = 0;
    __Pyx_DECREF(__pyx_t_1); __pyx_t_1 = 0;
    __pyx_t_11 = __Pyx_PyObject_IsTrue(__pyx_t_8); if (unlikely(__pyx_t_11 < 0)) __PYX_ERR(0, 86, __pyx_L1_error)
    __Pyx_DECREF(__pyx_t_8); __pyx_t_8 = 0;
    if (__pyx_t_11) {
/* … */
      goto __pyx_L5;
    }
```

```
+087:             opt_result.x = np.append([v_intl_dc, v_intl_ga], np.array([d_intl_lambda]))
```

```
      __pyx_t_1 = __Pyx_GetModuleGlobalName(__pyx_n_s_np); if (unlikely(!__pyx_t_1)) __PYX_ERR(0, 87, __pyx_L1_error)
      __Pyx_GOTREF(__pyx_t_1);
      __pyx_t_10 = __Pyx_PyObject_GetAttrStr(__pyx_t_1, __pyx_n_s_append); if (unlikely(!__pyx_t_10)) __PYX_ERR(0, 87, __pyx_L1_error)
      __Pyx_GOTREF(__pyx_t_10);
      __Pyx_DECREF(__pyx_t_1); __pyx_t_1 = 0;
      __pyx_t_1 = PyList_New(2); if (unlikely(!__pyx_t_1)) __PYX_ERR(0, 87, __pyx_L1_error)
      __Pyx_GOTREF(__pyx_t_1);
      __Pyx_INCREF(__pyx_v_v_intl_dc);
      __Pyx_GIVEREF(__pyx_v_v_intl_dc);
      PyList_SET_ITEM(__pyx_t_1, 0, __pyx_v_v_intl_dc);
      __Pyx_INCREF(__pyx_v_v_intl_ga);
      __Pyx_GIVEREF(__pyx_v_v_intl_ga);
      PyList_SET_ITEM(__pyx_t_1, 1, __pyx_v_v_intl_ga);
      __pyx_t_3 = __Pyx_GetModuleGlobalName(__pyx_n_s_np); if (unlikely(!__pyx_t_3)) __PYX_ERR(0, 87, __pyx_L1_error)
      __Pyx_GOTREF(__pyx_t_3);
      __pyx_t_4 = __Pyx_PyObject_GetAttrStr(__pyx_t_3, __pyx_n_s_array); if (unlikely(!__pyx_t_4)) __PYX_ERR(0, 87, __pyx_L1_error)
      __Pyx_GOTREF(__pyx_t_4);
      __Pyx_DECREF(__pyx_t_3); __pyx_t_3 = 0;
      __pyx_t_3 = PyList_New(1); if (unlikely(!__pyx_t_3)) __PYX_ERR(0, 87, __pyx_L1_error)
      __Pyx_GOTREF(__pyx_t_3);
      __Pyx_INCREF(__pyx_v_d_intl_lambda);
      __Pyx_GIVEREF(__pyx_v_d_intl_lambda);
      PyList_SET_ITEM(__pyx_t_3, 0, __pyx_v_d_intl_lambda);
      __pyx_t_7 = NULL;
      if (CYTHON_UNPACK_METHODS && unlikely(PyMethod_Check(__pyx_t_4))) {
        __pyx_t_7 = PyMethod_GET_SELF(__pyx_t_4);
        if (likely(__pyx_t_7)) {
          PyObject* function = PyMethod_GET_FUNCTION(__pyx_t_4);
          __Pyx_INCREF(__pyx_t_7);
          __Pyx_INCREF(function);
          __Pyx_DECREF_SET(__pyx_t_4, function);
        }
      }
      if (!__pyx_t_7) {
        __pyx_t_6 = __Pyx_PyObject_CallOneArg(__pyx_t_4, __pyx_t_3); if (unlikely(!__pyx_t_6)) __PYX_ERR(0, 87, __pyx_L1_error)
        __Pyx_DECREF(__pyx_t_3); __pyx_t_3 = 0;
        __Pyx_GOTREF(__pyx_t_6);
      } else {
        #if CYTHON_FAST_PYCALL
        if (PyFunction_Check(__pyx_t_4)) {
          PyObject *__pyx_temp[2] = {__pyx_t_7, __pyx_t_3};
          __pyx_t_6 = __Pyx_PyFunction_FastCall(__pyx_t_4, __pyx_temp+1-1, 1+1); if (unlikely(!__pyx_t_6)) __PYX_ERR(0, 87, __pyx_L1_error)
          __Pyx_XDECREF(__pyx_t_7); __pyx_t_7 = 0;
          __Pyx_GOTREF(__pyx_t_6);
          __Pyx_DECREF(__pyx_t_3); __pyx_t_3 = 0;
        } else
        #endif
        #if CYTHON_FAST_PYCCALL
        if (__Pyx_PyFastCFunction_Check(__pyx_t_4)) {
          PyObject *__pyx_temp[2] = {__pyx_t_7, __pyx_t_3};
          __pyx_t_6 = __Pyx_PyCFunction_FastCall(__pyx_t_4, __pyx_temp+1-1, 1+1); if (unlikely(!__pyx_t_6)) __PYX_ERR(0, 87, __pyx_L1_error)
          __Pyx_XDECREF(__pyx_t_7); __pyx_t_7 = 0;
          __Pyx_GOTREF(__pyx_t_6);
          __Pyx_DECREF(__pyx_t_3); __pyx_t_3 = 0;
        } else
        #endif
        {
          __pyx_t_9 = PyTuple_New(1+1); if (unlikely(!__pyx_t_9)) __PYX_ERR(0, 87, __pyx_L1_error)
          __Pyx_GOTREF(__pyx_t_9);
          __Pyx_GIVEREF(__pyx_t_7); PyTuple_SET_ITEM(__pyx_t_9, 0, __pyx_t_7); __pyx_t_7 = NULL;
          __Pyx_GIVEREF(__pyx_t_3);
          PyTuple_SET_ITEM(__pyx_t_9, 0+1, __pyx_t_3);
          __pyx_t_3 = 0;
          __pyx_t_6 = __Pyx_PyObject_Call(__pyx_t_4, __pyx_t_9, NULL); if (unlikely(!__pyx_t_6)) __PYX_ERR(0, 87, __pyx_L1_error)
          __Pyx_GOTREF(__pyx_t_6);
          __Pyx_DECREF(__pyx_t_9); __pyx_t_9 = 0;
        }
      }
      __Pyx_DECREF(__pyx_t_4); __pyx_t_4 = 0;
      __pyx_t_4 = NULL;
      __pyx_t_5 = 0;
      if (CYTHON_UNPACK_METHODS && unlikely(PyMethod_Check(__pyx_t_10))) {
        __pyx_t_4 = PyMethod_GET_SELF(__pyx_t_10);
        if (likely(__pyx_t_4)) {
          PyObject* function = PyMethod_GET_FUNCTION(__pyx_t_10);
          __Pyx_INCREF(__pyx_t_4);
          __Pyx_INCREF(function);
          __Pyx_DECREF_SET(__pyx_t_10, function);
          __pyx_t_5 = 1;
        }
      }
      #if CYTHON_FAST_PYCALL
      if (PyFunction_Check(__pyx_t_10)) {
        PyObject *__pyx_temp[3] = {__pyx_t_4, __pyx_t_1, __pyx_t_6};
        __pyx_t_8 = __Pyx_PyFunction_FastCall(__pyx_t_10, __pyx_temp+1-__pyx_t_5, 2+__pyx_t_5); if (unlikely(!__pyx_t_8)) __PYX_ERR(0, 87, __pyx_L1_error)
        __Pyx_XDECREF(__pyx_t_4); __pyx_t_4 = 0;
        __Pyx_GOTREF(__pyx_t_8);
        __Pyx_DECREF(__pyx_t_1); __pyx_t_1 = 0;
        __Pyx_DECREF(__pyx_t_6); __pyx_t_6 = 0;
      } else
      #endif
      #if CYTHON_FAST_PYCCALL
      if (__Pyx_PyFastCFunction_Check(__pyx_t_10)) {
        PyObject *__pyx_temp[3] = {__pyx_t_4, __pyx_t_1, __pyx_t_6};
        __pyx_t_8 = __Pyx_PyCFunction_FastCall(__pyx_t_10, __pyx_temp+1-__pyx_t_5, 2+__pyx_t_5); if (unlikely(!__pyx_t_8)) __PYX_ERR(0, 87, __pyx_L1_error)
        __Pyx_XDECREF(__pyx_t_4); __pyx_t_4 = 0;
        __Pyx_GOTREF(__pyx_t_8);
        __Pyx_DECREF(__pyx_t_1); __pyx_t_1 = 0;
        __Pyx_DECREF(__pyx_t_6); __pyx_t_6 = 0;
      } else
      #endif
      {
        __pyx_t_9 = PyTuple_New(2+__pyx_t_5); if (unlikely(!__pyx_t_9)) __PYX_ERR(0, 87, __pyx_L1_error)
        __Pyx_GOTREF(__pyx_t_9);
        if (__pyx_t_4) {
          __Pyx_GIVEREF(__pyx_t_4); PyTuple_SET_ITEM(__pyx_t_9, 0, __pyx_t_4); __pyx_t_4 = NULL;
        }
        __Pyx_GIVEREF(__pyx_t_1);
        PyTuple_SET_ITEM(__pyx_t_9, 0+__pyx_t_5, __pyx_t_1);
        __Pyx_GIVEREF(__pyx_t_6);
        PyTuple_SET_ITEM(__pyx_t_9, 1+__pyx_t_5, __pyx_t_6);
        __pyx_t_1 = 0;
        __pyx_t_6 = 0;
        __pyx_t_8 = __Pyx_PyObject_Call(__pyx_t_10, __pyx_t_9, NULL); if (unlikely(!__pyx_t_8)) __PYX_ERR(0, 87, __pyx_L1_error)
        __Pyx_GOTREF(__pyx_t_8);
        __Pyx_DECREF(__pyx_t_9); __pyx_t_9 = 0;
      }
      __Pyx_DECREF(__pyx_t_10); __pyx_t_10 = 0;
      if (__Pyx_PyObject_SetAttrStr(__pyx_v_opt_result, __pyx_n_s_x, __pyx_t_8) < 0) __PYX_ERR(0, 87, __pyx_L1_error)
      __Pyx_DECREF(__pyx_t_8); __pyx_t_8 = 0;
```

```
 088:         else:
```

```
+089:             v_intl_dc = opt_result.x[0:n_helpers]
```

```
    /*else*/ {
      __pyx_t_8 = __Pyx_PyObject_GetAttrStr(__pyx_v_opt_result, __pyx_n_s_x); if (unlikely(!__pyx_t_8)) __PYX_ERR(0, 89, __pyx_L1_error)
      __Pyx_GOTREF(__pyx_t_8);
      __pyx_t_10 = __Pyx_PyObject_GetSlice(__pyx_t_8, 0, 0, NULL, &__pyx_cur_scope->__pyx_v_n_helpers, NULL, 1, 0, 1); if (unlikely(!__pyx_t_10)) __PYX_ERR(0, 89, __pyx_L1_error)
      __Pyx_GOTREF(__pyx_t_10);
      __Pyx_DECREF(__pyx_t_8); __pyx_t_8 = 0;
      __Pyx_DECREF_SET(__pyx_v_v_intl_dc, __pyx_t_10);
      __pyx_t_10 = 0;
```

```
+090:             v_intl_ga = opt_result.x[n_helpers:-1]
```

```
      __pyx_t_10 = __Pyx_PyObject_GetAttrStr(__pyx_v_opt_result, __pyx_n_s_x); if (unlikely(!__pyx_t_10)) __PYX_ERR(0, 90, __pyx_L1_error)
      __Pyx_GOTREF(__pyx_t_10);
      __pyx_t_8 = __Pyx_PyObject_GetSlice(__pyx_t_10, 0, -1L, &__pyx_cur_scope->__pyx_v_n_helpers, NULL, NULL, 0, 1, 1); if (unlikely(!__pyx_t_8)) __PYX_ERR(0, 90, __pyx_L1_error)
      __Pyx_GOTREF(__pyx_t_8);
      __Pyx_DECREF(__pyx_t_10); __pyx_t_10 = 0;
      __Pyx_DECREF_SET(__pyx_v_v_intl_ga, __pyx_t_8);
      __pyx_t_8 = 0;
```

```
+091:             d_intl_lambda = opt_result.x[-1]
```

```
      __pyx_t_8 = __Pyx_PyObject_GetAttrStr(__pyx_v_opt_result, __pyx_n_s_x); if (unlikely(!__pyx_t_8)) __PYX_ERR(0, 91, __pyx_L1_error)
      __Pyx_GOTREF(__pyx_t_8);
      __pyx_t_10 = __Pyx_GetItemInt(__pyx_t_8, -1L, long, 1, __Pyx_PyInt_From_long, 0, 1, 1); if (unlikely(!__pyx_t_10)) __PYX_ERR(0, 91, __pyx_L1_error)
      __Pyx_GOTREF(__pyx_t_10);
      __Pyx_DECREF(__pyx_t_8); __pyx_t_8 = 0;
      __Pyx_DECREF_SET(__pyx_v_d_intl_lambda, __pyx_t_10);
      __pyx_t_10 = 0;
```

```
+092:             v_best = np.append([v_intl_dc, v_intl_ga], np.array([d_intl_lambda]))
```

```
      __pyx_t_8 = __Pyx_GetModuleGlobalName(__pyx_n_s_np); if (unlikely(!__pyx_t_8)) __PYX_ERR(0, 92, __pyx_L1_error)
      __Pyx_GOTREF(__pyx_t_8);
      __pyx_t_9 = __Pyx_PyObject_GetAttrStr(__pyx_t_8, __pyx_n_s_append); if (unlikely(!__pyx_t_9)) __PYX_ERR(0, 92, __pyx_L1_error)
      __Pyx_GOTREF(__pyx_t_9);
      __Pyx_DECREF(__pyx_t_8); __pyx_t_8 = 0;
      __pyx_t_8 = PyList_New(2); if (unlikely(!__pyx_t_8)) __PYX_ERR(0, 92, __pyx_L1_error)
      __Pyx_GOTREF(__pyx_t_8);
      __Pyx_INCREF(__pyx_v_v_intl_dc);
      __Pyx_GIVEREF(__pyx_v_v_intl_dc);
      PyList_SET_ITEM(__pyx_t_8, 0, __pyx_v_v_intl_dc);
      __Pyx_INCREF(__pyx_v_v_intl_ga);
      __Pyx_GIVEREF(__pyx_v_v_intl_ga);
      PyList_SET_ITEM(__pyx_t_8, 1, __pyx_v_v_intl_ga);
      __pyx_t_1 = __Pyx_GetModuleGlobalName(__pyx_n_s_np); if (unlikely(!__pyx_t_1)) __PYX_ERR(0, 92, __pyx_L1_error)
      __Pyx_GOTREF(__pyx_t_1);
      __pyx_t_4 = __Pyx_PyObject_GetAttrStr(__pyx_t_1, __pyx_n_s_array); if (unlikely(!__pyx_t_4)) __PYX_ERR(0, 92, __pyx_L1_error)
      __Pyx_GOTREF(__pyx_t_4);
      __Pyx_DECREF(__pyx_t_1); __pyx_t_1 = 0;
      __pyx_t_1 = PyList_New(1); if (unlikely(!__pyx_t_1)) __PYX_ERR(0, 92, __pyx_L1_error)
      __Pyx_GOTREF(__pyx_t_1);
      __Pyx_INCREF(__pyx_v_d_intl_lambda);
      __Pyx_GIVEREF(__pyx_v_d_intl_lambda);
      PyList_SET_ITEM(__pyx_t_1, 0, __pyx_v_d_intl_lambda);
      __pyx_t_3 = NULL;
      if (CYTHON_UNPACK_METHODS && unlikely(PyMethod_Check(__pyx_t_4))) {
        __pyx_t_3 = PyMethod_GET_SELF(__pyx_t_4);
        if (likely(__pyx_t_3)) {
          PyObject* function = PyMethod_GET_FUNCTION(__pyx_t_4);
          __Pyx_INCREF(__pyx_t_3);
          __Pyx_INCREF(function);
          __Pyx_DECREF_SET(__pyx_t_4, function);
        }
      }
      if (!__pyx_t_3) {
        __pyx_t_6 = __Pyx_PyObject_CallOneArg(__pyx_t_4, __pyx_t_1); if (unlikely(!__pyx_t_6)) __PYX_ERR(0, 92, __pyx_L1_error)
        __Pyx_DECREF(__pyx_t_1); __pyx_t_1 = 0;
        __Pyx_GOTREF(__pyx_t_6);
      } else {
        #if CYTHON_FAST_PYCALL
        if (PyFunction_Check(__pyx_t_4)) {
          PyObject *__pyx_temp[2] = {__pyx_t_3, __pyx_t_1};
          __pyx_t_6 = __Pyx_PyFunction_FastCall(__pyx_t_4, __pyx_temp+1-1, 1+1); if (unlikely(!__pyx_t_6)) __PYX_ERR(0, 92, __pyx_L1_error)
          __Pyx_XDECREF(__pyx_t_3); __pyx_t_3 = 0;
          __Pyx_GOTREF(__pyx_t_6);
          __Pyx_DECREF(__pyx_t_1); __pyx_t_1 = 0;
        } else
        #endif
        #if CYTHON_FAST_PYCCALL
        if (__Pyx_PyFastCFunction_Check(__pyx_t_4)) {
          PyObject *__pyx_temp[2] = {__pyx_t_3, __pyx_t_1};
          __pyx_t_6 = __Pyx_PyCFunction_FastCall(__pyx_t_4, __pyx_temp+1-1, 1+1); if (unlikely(!__pyx_t_6)) __PYX_ERR(0, 92, __pyx_L1_error)
          __Pyx_XDECREF(__pyx_t_3); __pyx_t_3 = 0;
          __Pyx_GOTREF(__pyx_t_6);
          __Pyx_DECREF(__pyx_t_1); __pyx_t_1 = 0;
        } else
        #endif
        {
          __pyx_t_7 = PyTuple_New(1+1); if (unlikely(!__pyx_t_7)) __PYX_ERR(0, 92, __pyx_L1_error)
          __Pyx_GOTREF(__pyx_t_7);
          __Pyx_GIVEREF(__pyx_t_3); PyTuple_SET_ITEM(__pyx_t_7, 0, __pyx_t_3); __pyx_t_3 = NULL;
          __Pyx_GIVEREF(__pyx_t_1);
          PyTuple_SET_ITEM(__pyx_t_7, 0+1, __pyx_t_1);
          __pyx_t_1 = 0;
          __pyx_t_6 = __Pyx_PyObject_Call(__pyx_t_4, __pyx_t_7, NULL); if (unlikely(!__pyx_t_6)) __PYX_ERR(0, 92, __pyx_L1_error)
          __Pyx_GOTREF(__pyx_t_6);
          __Pyx_DECREF(__pyx_t_7); __pyx_t_7 = 0;
        }
      }
      __Pyx_DECREF(__pyx_t_4); __pyx_t_4 = 0;
      __pyx_t_4 = NULL;
      __pyx_t_5 = 0;
      if (CYTHON_UNPACK_METHODS && unlikely(PyMethod_Check(__pyx_t_9))) {
        __pyx_t_4 = PyMethod_GET_SELF(__pyx_t_9);
        if (likely(__pyx_t_4)) {
          PyObject* function = PyMethod_GET_FUNCTION(__pyx_t_9);
          __Pyx_INCREF(__pyx_t_4);
          __Pyx_INCREF(function);
          __Pyx_DECREF_SET(__pyx_t_9, function);
          __pyx_t_5 = 1;
        }
      }
      #if CYTHON_FAST_PYCALL
      if (PyFunction_Check(__pyx_t_9)) {
        PyObject *__pyx_temp[3] = {__pyx_t_4, __pyx_t_8, __pyx_t_6};
        __pyx_t_10 = __Pyx_PyFunction_FastCall(__pyx_t_9, __pyx_temp+1-__pyx_t_5, 2+__pyx_t_5); if (unlikely(!__pyx_t_10)) __PYX_ERR(0, 92, __pyx_L1_error)
        __Pyx_XDECREF(__pyx_t_4); __pyx_t_4 = 0;
        __Pyx_GOTREF(__pyx_t_10);
        __Pyx_DECREF(__pyx_t_8); __pyx_t_8 = 0;
        __Pyx_DECREF(__pyx_t_6); __pyx_t_6 = 0;
      } else
      #endif
      #if CYTHON_FAST_PYCCALL
      if (__Pyx_PyFastCFunction_Check(__pyx_t_9)) {
        PyObject *__pyx_temp[3] = {__pyx_t_4, __pyx_t_8, __pyx_t_6};
        __pyx_t_10 = __Pyx_PyCFunction_FastCall(__pyx_t_9, __pyx_temp+1-__pyx_t_5, 2+__pyx_t_5); if (unlikely(!__pyx_t_10)) __PYX_ERR(0, 92, __pyx_L1_error)
        __Pyx_XDECREF(__pyx_t_4); __pyx_t_4 = 0;
        __Pyx_GOTREF(__pyx_t_10);
        __Pyx_DECREF(__pyx_t_8); __pyx_t_8 = 0;
        __Pyx_DECREF(__pyx_t_6); __pyx_t_6 = 0;
      } else
      #endif
      {
        __pyx_t_7 = PyTuple_New(2+__pyx_t_5); if (unlikely(!__pyx_t_7)) __PYX_ERR(0, 92, __pyx_L1_error)
        __Pyx_GOTREF(__pyx_t_7);
        if (__pyx_t_4) {
          __Pyx_GIVEREF(__pyx_t_4); PyTuple_SET_ITEM(__pyx_t_7, 0, __pyx_t_4); __pyx_t_4 = NULL;
        }
        __Pyx_GIVEREF(__pyx_t_8);
        PyTuple_SET_ITEM(__pyx_t_7, 0+__pyx_t_5, __pyx_t_8);
        __Pyx_GIVEREF(__pyx_t_6);
        PyTuple_SET_ITEM(__pyx_t_7, 1+__pyx_t_5, __pyx_t_6);
        __pyx_t_8 = 0;
        __pyx_t_6 = 0;
        __pyx_t_10 = __Pyx_PyObject_Call(__pyx_t_9, __pyx_t_7, NULL); if (unlikely(!__pyx_t_10)) __PYX_ERR(0, 92, __pyx_L1_error)
        __Pyx_GOTREF(__pyx_t_10);
        __Pyx_DECREF(__pyx_t_7); __pyx_t_7 = 0;
      }
      __Pyx_DECREF(__pyx_t_9); __pyx_t_9 = 0;
      __Pyx_DECREF_SET(__pyx_v_v_best, __pyx_t_10);
      __pyx_t_10 = 0;
    }
    __pyx_L5:;
```

```
 093:
```

```
+094:         d_brlx = d_brlx/4
```

```
    __pyx_t_10 = __Pyx_PyNumber_Divide(__pyx_cur_scope->__pyx_v_d_brlx, __pyx_int_4); if (unlikely(!__pyx_t_10)) __PYX_ERR(0, 94, __pyx_L1_error)
    __Pyx_GOTREF(__pyx_t_10);
    __Pyx_GOTREF(__pyx_cur_scope->__pyx_v_d_brlx);
    __Pyx_DECREF_SET(__pyx_cur_scope->__pyx_v_d_brlx, __pyx_t_10);
    __Pyx_GIVEREF(__pyx_t_10);
    __pyx_t_10 = 0;
```

```
+095:         if d_brlx > -0.02:
```

```
    __pyx_t_10 = PyObject_RichCompare(__pyx_cur_scope->__pyx_v_d_brlx, __pyx_float_neg_0_02, Py_GT); __Pyx_XGOTREF(__pyx_t_10); if (unlikely(!__pyx_t_10)) __PYX_ERR(0, 95, __pyx_L1_error)
    __pyx_t_11 = __Pyx_PyObject_IsTrue(__pyx_t_10); if (unlikely(__pyx_t_11 < 0)) __PYX_ERR(0, 95, __pyx_L1_error)
    __Pyx_DECREF(__pyx_t_10); __pyx_t_10 = 0;
    if (__pyx_t_11) {
/* … */
    }
  }
  __pyx_L4_break:;
```

```
+096:             break
```

```
      goto __pyx_L4_break;
```

```
 097:
```

```
 098:     # Give up
```

```
+099:     b_is_worse = rate_opt_dc(v_channel, nda_interf, opt_result) < d_opt_ga
```

```
  __pyx_t_9 = __Pyx_GetModuleGlobalName(__pyx_n_s_rate_opt_dc); if (unlikely(!__pyx_t_9)) __PYX_ERR(0, 99, __pyx_L1_error)
  __Pyx_GOTREF(__pyx_t_9);
  __pyx_t_7 = NULL;
  __pyx_t_5 = 0;
  if (CYTHON_UNPACK_METHODS && unlikely(PyMethod_Check(__pyx_t_9))) {
    __pyx_t_7 = PyMethod_GET_SELF(__pyx_t_9);
    if (likely(__pyx_t_7)) {
      PyObject* function = PyMethod_GET_FUNCTION(__pyx_t_9);
      __Pyx_INCREF(__pyx_t_7);
      __Pyx_INCREF(function);
      __Pyx_DECREF_SET(__pyx_t_9, function);
      __pyx_t_5 = 1;
    }
  }
  #if CYTHON_FAST_PYCALL
  if (PyFunction_Check(__pyx_t_9)) {
    PyObject *__pyx_temp[4] = {__pyx_t_7, __pyx_cur_scope->__pyx_v_v_channel, __pyx_cur_scope->__pyx_v_nda_interf, __pyx_v_opt_result};
    __pyx_t_10 = __Pyx_PyFunction_FastCall(__pyx_t_9, __pyx_temp+1-__pyx_t_5, 3+__pyx_t_5); if (unlikely(!__pyx_t_10)) __PYX_ERR(0, 99, __pyx_L1_error)
    __Pyx_XDECREF(__pyx_t_7); __pyx_t_7 = 0;
    __Pyx_GOTREF(__pyx_t_10);
  } else
  #endif
  #if CYTHON_FAST_PYCCALL
  if (__Pyx_PyFastCFunction_Check(__pyx_t_9)) {
    PyObject *__pyx_temp[4] = {__pyx_t_7, __pyx_cur_scope->__pyx_v_v_channel, __pyx_cur_scope->__pyx_v_nda_interf, __pyx_v_opt_result};
    __pyx_t_10 = __Pyx_PyCFunction_FastCall(__pyx_t_9, __pyx_temp+1-__pyx_t_5, 3+__pyx_t_5); if (unlikely(!__pyx_t_10)) __PYX_ERR(0, 99, __pyx_L1_error)
    __Pyx_XDECREF(__pyx_t_7); __pyx_t_7 = 0;
    __Pyx_GOTREF(__pyx_t_10);
  } else
  #endif
  {
    __pyx_t_6 = PyTuple_New(3+__pyx_t_5); if (unlikely(!__pyx_t_6)) __PYX_ERR(0, 99, __pyx_L1_error)
    __Pyx_GOTREF(__pyx_t_6);
    if (__pyx_t_7) {
      __Pyx_GIVEREF(__pyx_t_7); PyTuple_SET_ITEM(__pyx_t_6, 0, __pyx_t_7); __pyx_t_7 = NULL;
    }
    __Pyx_INCREF(__pyx_cur_scope->__pyx_v_v_channel);
    __Pyx_GIVEREF(__pyx_cur_scope->__pyx_v_v_channel);
    PyTuple_SET_ITEM(__pyx_t_6, 0+__pyx_t_5, __pyx_cur_scope->__pyx_v_v_channel);
    __Pyx_INCREF(__pyx_cur_scope->__pyx_v_nda_interf);
    __Pyx_GIVEREF(__pyx_cur_scope->__pyx_v_nda_interf);
    PyTuple_SET_ITEM(__pyx_t_6, 1+__pyx_t_5, __pyx_cur_scope->__pyx_v_nda_interf);
    __Pyx_INCREF(__pyx_v_opt_result);
    __Pyx_GIVEREF(__pyx_v_opt_result);
    PyTuple_SET_ITEM(__pyx_t_6, 2+__pyx_t_5, __pyx_v_opt_result);
    __pyx_t_10 = __Pyx_PyObject_Call(__pyx_t_9, __pyx_t_6, NULL); if (unlikely(!__pyx_t_10)) __PYX_ERR(0, 99, __pyx_L1_error)
    __Pyx_GOTREF(__pyx_t_10);
    __Pyx_DECREF(__pyx_t_6); __pyx_t_6 = 0;
  }
  __Pyx_DECREF(__pyx_t_9); __pyx_t_9 = 0;
  __pyx_t_9 = PyObject_RichCompare(__pyx_t_10, __pyx_v_d_opt_ga, Py_LT); __Pyx_XGOTREF(__pyx_t_9); if (unlikely(!__pyx_t_9)) __PYX_ERR(0, 99, __pyx_L1_error)
  __Pyx_DECREF(__pyx_t_10); __pyx_t_10 = 0;
  __Pyx_XDECREF_SET(__pyx_v_b_is_worse, __pyx_t_9);
  __pyx_t_9 = 0;
```

```
+100:     if (sum(np.isnan(opt_result.x)) > 0) | b_is_worse:
```

```
  __pyx_t_10 = __Pyx_GetModuleGlobalName(__pyx_n_s_np); if (unlikely(!__pyx_t_10)) __PYX_ERR(0, 100, __pyx_L1_error)
  __Pyx_GOTREF(__pyx_t_10);
  __pyx_t_6 = __Pyx_PyObject_GetAttrStr(__pyx_t_10, __pyx_n_s_isnan); if (unlikely(!__pyx_t_6)) __PYX_ERR(0, 100, __pyx_L1_error)
  __Pyx_GOTREF(__pyx_t_6);
  __Pyx_DECREF(__pyx_t_10); __pyx_t_10 = 0;
  __pyx_t_10 = __Pyx_PyObject_GetAttrStr(__pyx_v_opt_result, __pyx_n_s_x); if (unlikely(!__pyx_t_10)) __PYX_ERR(0, 100, __pyx_L1_error)
  __Pyx_GOTREF(__pyx_t_10);
  __pyx_t_7 = NULL;
  if (CYTHON_UNPACK_METHODS && unlikely(PyMethod_Check(__pyx_t_6))) {
    __pyx_t_7 = PyMethod_GET_SELF(__pyx_t_6);
    if (likely(__pyx_t_7)) {
      PyObject* function = PyMethod_GET_FUNCTION(__pyx_t_6);
      __Pyx_INCREF(__pyx_t_7);
      __Pyx_INCREF(function);
      __Pyx_DECREF_SET(__pyx_t_6, function);
    }
  }
  if (!__pyx_t_7) {
    __pyx_t_9 = __Pyx_PyObject_CallOneArg(__pyx_t_6, __pyx_t_10); if (unlikely(!__pyx_t_9)) __PYX_ERR(0, 100, __pyx_L1_error)
    __Pyx_DECREF(__pyx_t_10); __pyx_t_10 = 0;
    __Pyx_GOTREF(__pyx_t_9);
  } else {
    #if CYTHON_FAST_PYCALL
    if (PyFunction_Check(__pyx_t_6)) {
      PyObject *__pyx_temp[2] = {__pyx_t_7, __pyx_t_10};
      __pyx_t_9 = __Pyx_PyFunction_FastCall(__pyx_t_6, __pyx_temp+1-1, 1+1); if (unlikely(!__pyx_t_9)) __PYX_ERR(0, 100, __pyx_L1_error)
      __Pyx_XDECREF(__pyx_t_7); __pyx_t_7 = 0;
      __Pyx_GOTREF(__pyx_t_9);
      __Pyx_DECREF(__pyx_t_10); __pyx_t_10 = 0;
    } else
    #endif
    #if CYTHON_FAST_PYCCALL
    if (__Pyx_PyFastCFunction_Check(__pyx_t_6)) {
      PyObject *__pyx_temp[2] = {__pyx_t_7, __pyx_t_10};
      __pyx_t_9 = __Pyx_PyCFunction_FastCall(__pyx_t_6, __pyx_temp+1-1, 1+1); if (unlikely(!__pyx_t_9)) __PYX_ERR(0, 100, __pyx_L1_error)
      __Pyx_XDECREF(__pyx_t_7); __pyx_t_7 = 0;
      __Pyx_GOTREF(__pyx_t_9);
      __Pyx_DECREF(__pyx_t_10); __pyx_t_10 = 0;
    } else
    #endif
    {
      __pyx_t_8 = PyTuple_New(1+1); if (unlikely(!__pyx_t_8)) __PYX_ERR(0, 100, __pyx_L1_error)
      __Pyx_GOTREF(__pyx_t_8);
      __Pyx_GIVEREF(__pyx_t_7); PyTuple_SET_ITEM(__pyx_t_8, 0, __pyx_t_7); __pyx_t_7 = NULL;
      __Pyx_GIVEREF(__pyx_t_10);
      PyTuple_SET_ITEM(__pyx_t_8, 0+1, __pyx_t_10);
      __pyx_t_10 = 0;
      __pyx_t_9 = __Pyx_PyObject_Call(__pyx_t_6, __pyx_t_8, NULL); if (unlikely(!__pyx_t_9)) __PYX_ERR(0, 100, __pyx_L1_error)
      __Pyx_GOTREF(__pyx_t_9);
      __Pyx_DECREF(__pyx_t_8); __pyx_t_8 = 0;
    }
  }
  __Pyx_DECREF(__pyx_t_6); __pyx_t_6 = 0;
  __pyx_t_6 = PyTuple_New(1); if (unlikely(!__pyx_t_6)) __PYX_ERR(0, 100, __pyx_L1_error)
  __Pyx_GOTREF(__pyx_t_6);
  __Pyx_GIVEREF(__pyx_t_9);
  PyTuple_SET_ITEM(__pyx_t_6, 0, __pyx_t_9);
  __pyx_t_9 = 0;
  __pyx_t_9 = __Pyx_PyObject_Call(__pyx_builtin_sum, __pyx_t_6, NULL); if (unlikely(!__pyx_t_9)) __PYX_ERR(0, 100, __pyx_L1_error)
  __Pyx_GOTREF(__pyx_t_9);
  __Pyx_DECREF(__pyx_t_6); __pyx_t_6 = 0;
  __pyx_t_6 = PyObject_RichCompare(__pyx_t_9, __pyx_int_0, Py_GT); __Pyx_XGOTREF(__pyx_t_6); if (unlikely(!__pyx_t_6)) __PYX_ERR(0, 100, __pyx_L1_error)
  __Pyx_DECREF(__pyx_t_9); __pyx_t_9 = 0;
  __pyx_t_9 = PyNumber_Or(__pyx_t_6, __pyx_v_b_is_worse); if (unlikely(!__pyx_t_9)) __PYX_ERR(0, 100, __pyx_L1_error)
  __Pyx_GOTREF(__pyx_t_9);
  __Pyx_DECREF(__pyx_t_6); __pyx_t_6 = 0;
  __pyx_t_11 = __Pyx_PyObject_IsTrue(__pyx_t_9); if (unlikely(__pyx_t_11 < 0)) __PYX_ERR(0, 100, __pyx_L1_error)
  __Pyx_DECREF(__pyx_t_9); __pyx_t_9 = 0;
  if (__pyx_t_11) {
/* … */
  }
```

```
+101:         opt_result.x = np.append([v_opt_ga, v_opt_ga], np.array([0]))
```

```
    __pyx_t_6 = __Pyx_GetModuleGlobalName(__pyx_n_s_np); if (unlikely(!__pyx_t_6)) __PYX_ERR(0, 101, __pyx_L1_error)
    __Pyx_GOTREF(__pyx_t_6);
    __pyx_t_8 = __Pyx_PyObject_GetAttrStr(__pyx_t_6, __pyx_n_s_append); if (unlikely(!__pyx_t_8)) __PYX_ERR(0, 101, __pyx_L1_error)
    __Pyx_GOTREF(__pyx_t_8);
    __Pyx_DECREF(__pyx_t_6); __pyx_t_6 = 0;
    __pyx_t_6 = PyList_New(2); if (unlikely(!__pyx_t_6)) __PYX_ERR(0, 101, __pyx_L1_error)
    __Pyx_GOTREF(__pyx_t_6);
    __Pyx_INCREF(__pyx_v_v_opt_ga);
    __Pyx_GIVEREF(__pyx_v_v_opt_ga);
    PyList_SET_ITEM(__pyx_t_6, 0, __pyx_v_v_opt_ga);
    __Pyx_INCREF(__pyx_v_v_opt_ga);
    __Pyx_GIVEREF(__pyx_v_v_opt_ga);
    PyList_SET_ITEM(__pyx_t_6, 1, __pyx_v_v_opt_ga);
    __pyx_t_7 = __Pyx_GetModuleGlobalName(__pyx_n_s_np); if (unlikely(!__pyx_t_7)) __PYX_ERR(0, 101, __pyx_L1_error)
    __Pyx_GOTREF(__pyx_t_7);
    __pyx_t_4 = __Pyx_PyObject_GetAttrStr(__pyx_t_7, __pyx_n_s_array); if (unlikely(!__pyx_t_4)) __PYX_ERR(0, 101, __pyx_L1_error)
    __Pyx_GOTREF(__pyx_t_4);
    __Pyx_DECREF(__pyx_t_7); __pyx_t_7 = 0;
    __pyx_t_7 = PyList_New(1); if (unlikely(!__pyx_t_7)) __PYX_ERR(0, 101, __pyx_L1_error)
    __Pyx_GOTREF(__pyx_t_7);
    __Pyx_INCREF(__pyx_int_0);
    __Pyx_GIVEREF(__pyx_int_0);
    PyList_SET_ITEM(__pyx_t_7, 0, __pyx_int_0);
    __pyx_t_1 = NULL;
    if (CYTHON_UNPACK_METHODS && unlikely(PyMethod_Check(__pyx_t_4))) {
      __pyx_t_1 = PyMethod_GET_SELF(__pyx_t_4);
      if (likely(__pyx_t_1)) {
        PyObject* function = PyMethod_GET_FUNCTION(__pyx_t_4);
        __Pyx_INCREF(__pyx_t_1);
        __Pyx_INCREF(function);
        __Pyx_DECREF_SET(__pyx_t_4, function);
      }
    }
    if (!__pyx_t_1) {
      __pyx_t_10 = __Pyx_PyObject_CallOneArg(__pyx_t_4, __pyx_t_7); if (unlikely(!__pyx_t_10)) __PYX_ERR(0, 101, __pyx_L1_error)
      __Pyx_DECREF(__pyx_t_7); __pyx_t_7 = 0;
      __Pyx_GOTREF(__pyx_t_10);
    } else {
      #if CYTHON_FAST_PYCALL
      if (PyFunction_Check(__pyx_t_4)) {
        PyObject *__pyx_temp[2] = {__pyx_t_1, __pyx_t_7};
        __pyx_t_10 = __Pyx_PyFunction_FastCall(__pyx_t_4, __pyx_temp+1-1, 1+1); if (unlikely(!__pyx_t_10)) __PYX_ERR(0, 101, __pyx_L1_error)
        __Pyx_XDECREF(__pyx_t_1); __pyx_t_1 = 0;
        __Pyx_GOTREF(__pyx_t_10);
        __Pyx_DECREF(__pyx_t_7); __pyx_t_7 = 0;
      } else
      #endif
      #if CYTHON_FAST_PYCCALL
      if (__Pyx_PyFastCFunction_Check(__pyx_t_4)) {
        PyObject *__pyx_temp[2] = {__pyx_t_1, __pyx_t_7};
        __pyx_t_10 = __Pyx_PyCFunction_FastCall(__pyx_t_4, __pyx_temp+1-1, 1+1); if (unlikely(!__pyx_t_10)) __PYX_ERR(0, 101, __pyx_L1_error)
        __Pyx_XDECREF(__pyx_t_1); __pyx_t_1 = 0;
        __Pyx_GOTREF(__pyx_t_10);
        __Pyx_DECREF(__pyx_t_7); __pyx_t_7 = 0;
      } else
      #endif
      {
        __pyx_t_3 = PyTuple_New(1+1); if (unlikely(!__pyx_t_3)) __PYX_ERR(0, 101, __pyx_L1_error)
        __Pyx_GOTREF(__pyx_t_3);
        __Pyx_GIVEREF(__pyx_t_1); PyTuple_SET_ITEM(__pyx_t_3, 0, __pyx_t_1); __pyx_t_1 = NULL;
        __Pyx_GIVEREF(__pyx_t_7);
        PyTuple_SET_ITEM(__pyx_t_3, 0+1, __pyx_t_7);
        __pyx_t_7 = 0;
        __pyx_t_10 = __Pyx_PyObject_Call(__pyx_t_4, __pyx_t_3, NULL); if (unlikely(!__pyx_t_10)) __PYX_ERR(0, 101, __pyx_L1_error)
        __Pyx_GOTREF(__pyx_t_10);
        __Pyx_DECREF(__pyx_t_3); __pyx_t_3 = 0;
      }
    }
    __Pyx_DECREF(__pyx_t_4); __pyx_t_4 = 0;
    __pyx_t_4 = NULL;
    __pyx_t_5 = 0;
    if (CYTHON_UNPACK_METHODS && unlikely(PyMethod_Check(__pyx_t_8))) {
      __pyx_t_4 = PyMethod_GET_SELF(__pyx_t_8);
      if (likely(__pyx_t_4)) {
        PyObject* function = PyMethod_GET_FUNCTION(__pyx_t_8);
        __Pyx_INCREF(__pyx_t_4);
        __Pyx_INCREF(function);
        __Pyx_DECREF_SET(__pyx_t_8, function);
        __pyx_t_5 = 1;
      }
    }
    #if CYTHON_FAST_PYCALL
    if (PyFunction_Check(__pyx_t_8)) {
      PyObject *__pyx_temp[3] = {__pyx_t_4, __pyx_t_6, __pyx_t_10};
      __pyx_t_9 = __Pyx_PyFunction_FastCall(__pyx_t_8, __pyx_temp+1-__pyx_t_5, 2+__pyx_t_5); if (unlikely(!__pyx_t_9)) __PYX_ERR(0, 101, __pyx_L1_error)
      __Pyx_XDECREF(__pyx_t_4); __pyx_t_4 = 0;
      __Pyx_GOTREF(__pyx_t_9);
      __Pyx_DECREF(__pyx_t_6); __pyx_t_6 = 0;
      __Pyx_DECREF(__pyx_t_10); __pyx_t_10 = 0;
    } else
    #endif
    #if CYTHON_FAST_PYCCALL
    if (__Pyx_PyFastCFunction_Check(__pyx_t_8)) {
      PyObject *__pyx_temp[3] = {__pyx_t_4, __pyx_t_6, __pyx_t_10};
      __pyx_t_9 = __Pyx_PyCFunction_FastCall(__pyx_t_8, __pyx_temp+1-__pyx_t_5, 2+__pyx_t_5); if (unlikely(!__pyx_t_9)) __PYX_ERR(0, 101, __pyx_L1_error)
      __Pyx_XDECREF(__pyx_t_4); __pyx_t_4 = 0;
      __Pyx_GOTREF(__pyx_t_9);
      __Pyx_DECREF(__pyx_t_6); __pyx_t_6 = 0;
      __Pyx_DECREF(__pyx_t_10); __pyx_t_10 = 0;
    } else
    #endif
    {
      __pyx_t_3 = PyTuple_New(2+__pyx_t_5); if (unlikely(!__pyx_t_3)) __PYX_ERR(0, 101, __pyx_L1_error)
      __Pyx_GOTREF(__pyx_t_3);
      if (__pyx_t_4) {
        __Pyx_GIVEREF(__pyx_t_4); PyTuple_SET_ITEM(__pyx_t_3, 0, __pyx_t_4); __pyx_t_4 = NULL;
      }
      __Pyx_GIVEREF(__pyx_t_6);
      PyTuple_SET_ITEM(__pyx_t_3, 0+__pyx_t_5, __pyx_t_6);
      __Pyx_GIVEREF(__pyx_t_10);
      PyTuple_SET_ITEM(__pyx_t_3, 1+__pyx_t_5, __pyx_t_10);
      __pyx_t_6 = 0;
      __pyx_t_10 = 0;
      __pyx_t_9 = __Pyx_PyObject_Call(__pyx_t_8, __pyx_t_3, NULL); if (unlikely(!__pyx_t_9)) __PYX_ERR(0, 101, __pyx_L1_error)
      __Pyx_GOTREF(__pyx_t_9);
      __Pyx_DECREF(__pyx_t_3); __pyx_t_3 = 0;
    }
    __Pyx_DECREF(__pyx_t_8); __pyx_t_8 = 0;
    if (__Pyx_PyObject_SetAttrStr(__pyx_v_opt_result, __pyx_n_s_x, __pyx_t_9) < 0) __PYX_ERR(0, 101, __pyx_L1_error)
    __Pyx_DECREF(__pyx_t_9); __pyx_t_9 = 0;
```

```
 102:         # print('Optimization failed; using GA')
```

```
 103:
```

```
+104:     return opt_result
```

```
  __Pyx_XDECREF(__pyx_r);
  __Pyx_INCREF(__pyx_v_opt_result);
  __pyx_r = __pyx_v_opt_result;
  goto __pyx_L0;
```

```
 105:
```

```
 106:
```

```
+107: def rate_opt_dc(v_channel, nda_interf, opt_result_dc):
```

```
/* Python wrapper */
static PyObject *__pyx_pw_9dist_recv_9opt_rates_7rate_opt_dc(PyObject *__pyx_self, PyObject *__pyx_args, PyObject *__pyx_kwds); /*proto*/
static char __pyx_doc_9dist_recv_9opt_rates_6rate_opt_dc[] = "Given an opt_dc optimization result, return the associated dc rate it achieves";
static PyMethodDef __pyx_mdef_9dist_recv_9opt_rates_7rate_opt_dc = {"rate_opt_dc", (PyCFunction)__pyx_pw_9dist_recv_9opt_rates_7rate_opt_dc, METH_VARARGS|METH_KEYWORDS, __pyx_doc_9dist_recv_9opt_rates_6rate_opt_dc};
static PyObject *__pyx_pw_9dist_recv_9opt_rates_7rate_opt_dc(PyObject *__pyx_self, PyObject *__pyx_args, PyObject *__pyx_kwds) {
  PyObject *__pyx_v_v_channel = 0;
  PyObject *__pyx_v_nda_interf = 0;
  PyObject *__pyx_v_opt_result_dc = 0;
  PyObject *__pyx_r = 0;
  __Pyx_RefNannyDeclarations
  __Pyx_RefNannySetupContext("rate_opt_dc (wrapper)", 0);
  {
    static PyObject **__pyx_pyargnames[] = {&__pyx_n_s_v_channel,&__pyx_n_s_nda_interf,&__pyx_n_s_opt_result_dc,0};
    PyObject* values[3] = {0,0,0};
    if (unlikely(__pyx_kwds)) {
      Py_ssize_t kw_args;
      const Py_ssize_t pos_args = PyTuple_GET_SIZE(__pyx_args);
      switch (pos_args) {
        case  3: values[2] = PyTuple_GET_ITEM(__pyx_args, 2);
        case  2: values[1] = PyTuple_GET_ITEM(__pyx_args, 1);
        case  1: values[0] = PyTuple_GET_ITEM(__pyx_args, 0);
        case  0: break;
        default: goto __pyx_L5_argtuple_error;
      }
      kw_args = PyDict_Size(__pyx_kwds);
      switch (pos_args) {
        case  0:
        if (likely((values[0] = PyDict_GetItem(__pyx_kwds, __pyx_n_s_v_channel)) != 0)) kw_args--;
        else goto __pyx_L5_argtuple_error;
        case  1:
        if (likely((values[1] = PyDict_GetItem(__pyx_kwds, __pyx_n_s_nda_interf)) != 0)) kw_args--;
        else {
          __Pyx_RaiseArgtupleInvalid("rate_opt_dc", 1, 3, 3, 1); __PYX_ERR(0, 107, __pyx_L3_error)
        }
        case  2:
        if (likely((values[2] = PyDict_GetItem(__pyx_kwds, __pyx_n_s_opt_result_dc)) != 0)) kw_args--;
        else {
          __Pyx_RaiseArgtupleInvalid("rate_opt_dc", 1, 3, 3, 2); __PYX_ERR(0, 107, __pyx_L3_error)
        }
      }
      if (unlikely(kw_args > 0)) {
        if (unlikely(__Pyx_ParseOptionalKeywords(__pyx_kwds, __pyx_pyargnames, 0, values, pos_args, "rate_opt_dc") < 0)) __PYX_ERR(0, 107, __pyx_L3_error)
      }
    } else if (PyTuple_GET_SIZE(__pyx_args) != 3) {
      goto __pyx_L5_argtuple_error;
    } else {
      values[0] = PyTuple_GET_ITEM(__pyx_args, 0);
      values[1] = PyTuple_GET_ITEM(__pyx_args, 1);
      values[2] = PyTuple_GET_ITEM(__pyx_args, 2);
    }
    __pyx_v_v_channel = values[0];
    __pyx_v_nda_interf = values[1];
    __pyx_v_opt_result_dc = values[2];
  }
  goto __pyx_L4_argument_unpacking_done;
  __pyx_L5_argtuple_error:;
  __Pyx_RaiseArgtupleInvalid("rate_opt_dc", 1, 3, 3, PyTuple_GET_SIZE(__pyx_args)); __PYX_ERR(0, 107, __pyx_L3_error)
  __pyx_L3_error:;
  __Pyx_AddTraceback("dist_recv.opt_rates.rate_opt_dc", __pyx_clineno, __pyx_lineno, __pyx_filename);
  __Pyx_RefNannyFinishContext();
  return NULL;
  __pyx_L4_argument_unpacking_done:;
  __pyx_r = __pyx_pf_9dist_recv_9opt_rates_6rate_opt_dc(__pyx_self, __pyx_v_v_channel, __pyx_v_nda_interf, __pyx_v_opt_result_dc);

  /* function exit code */
  __Pyx_RefNannyFinishContext();
  return __pyx_r;
}

static PyObject *__pyx_pf_9dist_recv_9opt_rates_6rate_opt_dc(CYTHON_UNUSED PyObject *__pyx_self, PyObject *__pyx_v_v_channel, PyObject *__pyx_v_nda_interf, PyObject *__pyx_v_opt_result_dc) {
  Py_ssize_t __pyx_v_n_helpers;
  PyObject *__pyx_v_v_config = NULL;
  PyObject *__pyx_r = NULL;
  __Pyx_TraceDeclarations
  __Pyx_TraceFrameInit(__pyx_codeobj__5)
  __Pyx_RefNannyDeclarations
  __Pyx_RefNannySetupContext("rate_opt_dc", 0);
  __Pyx_TraceCall("rate_opt_dc", __pyx_f[0], 107, 0, __PYX_ERR(0, 107, __pyx_L1_error));
/* … */
  /* function exit code */
  __pyx_L1_error:;
  __Pyx_XDECREF(__pyx_t_2);
  __Pyx_XDECREF(__pyx_t_3);
  __Pyx_XDECREF(__pyx_t_4);
  __Pyx_AddTraceback("dist_recv.opt_rates.rate_opt_dc", __pyx_clineno, __pyx_lineno, __pyx_filename);
  __pyx_r = NULL;
  __pyx_L0:;
  __Pyx_XDECREF(__pyx_v_v_config);
  __Pyx_XGIVEREF(__pyx_r);
  __Pyx_TraceReturn(__pyx_r, 0);
  __Pyx_RefNannyFinishContext();
  return __pyx_r;
}
/* … */
  __pyx_tuple__10 = PyTuple_Pack(5, __pyx_n_s_v_channel, __pyx_n_s_nda_interf, __pyx_n_s_opt_result_dc, __pyx_n_s_n_helpers, __pyx_n_s_v_config); if (unlikely(!__pyx_tuple__10)) __PYX_ERR(0, 107, __pyx_L1_error)
  __Pyx_GOTREF(__pyx_tuple__10);
  __Pyx_GIVEREF(__pyx_tuple__10);
/* … */
  __pyx_t_2 = PyCFunction_NewEx(&__pyx_mdef_9dist_recv_9opt_rates_7rate_opt_dc, NULL, __pyx_n_s_dist_recv_opt_rates); if (unlikely(!__pyx_t_2)) __PYX_ERR(0, 107, __pyx_L1_error)
  __Pyx_GOTREF(__pyx_t_2);
  if (PyDict_SetItem(__pyx_d, __pyx_n_s_rate_opt_dc, __pyx_t_2) < 0) __PYX_ERR(0, 107, __pyx_L1_error)
  __Pyx_DECREF(__pyx_t_2); __pyx_t_2 = 0;
  __pyx_codeobj__5 = (PyObject*)__Pyx_PyCode_New(3, 0, 5, 0, 0, __pyx_empty_bytes, __pyx_empty_tuple, __pyx_empty_tuple, __pyx_tuple__10, __pyx_empty_tuple, __pyx_empty_tuple, __pyx_kp_s_Users_cdchapm2_Dropbox_Personal, __pyx_n_s_rate_opt_dc, 107, __pyx_empty_bytes); if (unlikely(!__pyx_codeobj__5)) __PYX_ERR(0, 107, __pyx_L1_error)
```

```
 108:     """Given an opt_dc optimization result, return the associated dc rate it achieves"""
```

```
+109:     n_helpers = len(v_channel)-1
```

```
  __pyx_t_1 = PyObject_Length(__pyx_v_v_channel); if (unlikely(__pyx_t_1 == -1)) __PYX_ERR(0, 109, __pyx_L1_error)
  __pyx_v_n_helpers = (__pyx_t_1 - 1);
```

```
+110:     v_config = opt_result_dc.x
```

```
  __pyx_t_2 = __Pyx_PyObject_GetAttrStr(__pyx_v_opt_result_dc, __pyx_n_s_x); if (unlikely(!__pyx_t_2)) __PYX_ERR(0, 110, __pyx_L1_error)
  __Pyx_GOTREF(__pyx_t_2);
  __pyx_v_v_config = __pyx_t_2;
  __pyx_t_2 = 0;
```

```
+111:     return rates.rate_dc(v_channel=v_channel,
```

```
  __Pyx_XDECREF(__pyx_r);
  __pyx_t_2 = __Pyx_GetModuleGlobalName(__pyx_n_s_rates); if (unlikely(!__pyx_t_2)) __PYX_ERR(0, 111, __pyx_L1_error)
  __Pyx_GOTREF(__pyx_t_2);
  __pyx_t_3 = __Pyx_PyObject_GetAttrStr(__pyx_t_2, __pyx_n_s_rate_dc); if (unlikely(!__pyx_t_3)) __PYX_ERR(0, 111, __pyx_L1_error)
  __Pyx_GOTREF(__pyx_t_3);
  __Pyx_DECREF(__pyx_t_2); __pyx_t_2 = 0;
  __pyx_t_2 = PyDict_New(); if (unlikely(!__pyx_t_2)) __PYX_ERR(0, 111, __pyx_L1_error)
  __Pyx_GOTREF(__pyx_t_2);
  if (PyDict_SetItem(__pyx_t_2, __pyx_n_s_v_channel, __pyx_v_v_channel) < 0) __PYX_ERR(0, 111, __pyx_L1_error)
/* … */
  __pyx_t_4 = __Pyx_PyObject_Call(__pyx_t_3, __pyx_empty_tuple, __pyx_t_2); if (unlikely(!__pyx_t_4)) __PYX_ERR(0, 111, __pyx_L1_error)
  __Pyx_GOTREF(__pyx_t_4);
  __Pyx_DECREF(__pyx_t_3); __pyx_t_3 = 0;
  __Pyx_DECREF(__pyx_t_2); __pyx_t_2 = 0;
  __pyx_r = __pyx_t_4;
  __pyx_t_4 = 0;
  goto __pyx_L0;
```

```
+112:                          nda_interf=nda_interf,
```

```
  if (PyDict_SetItem(__pyx_t_2, __pyx_n_s_nda_interf, __pyx_v_nda_interf) < 0) __PYX_ERR(0, 111, __pyx_L1_error)
```

```
+113:                          v_dc_bits=v_config[0:n_helpers],
```

```
  __pyx_t_4 = __Pyx_PyObject_GetSlice(__pyx_v_v_config, 0, __pyx_v_n_helpers, NULL, NULL, NULL, 1, 1, 1); if (unlikely(!__pyx_t_4)) __PYX_ERR(0, 113, __pyx_L1_error)
  __Pyx_GOTREF(__pyx_t_4);
  if (PyDict_SetItem(__pyx_t_2, __pyx_n_s_v_dc_bits, __pyx_t_4) < 0) __PYX_ERR(0, 111, __pyx_L1_error)
  __Pyx_DECREF(__pyx_t_4); __pyx_t_4 = 0;
```

```
+114:                          v_ga_bits=v_config[n_helpers:-1],
```

```
  __pyx_t_4 = __Pyx_PyObject_GetSlice(__pyx_v_v_config, __pyx_v_n_helpers, -1L, NULL, NULL, NULL, 1, 1, 1); if (unlikely(!__pyx_t_4)) __PYX_ERR(0, 114, __pyx_L1_error)
  __Pyx_GOTREF(__pyx_t_4);
  if (PyDict_SetItem(__pyx_t_2, __pyx_n_s_v_ga_bits, __pyx_t_4) < 0) __PYX_ERR(0, 111, __pyx_L1_error)
  __Pyx_DECREF(__pyx_t_4); __pyx_t_4 = 0;
```

```
+115:                          d_lambda=v_config[-1])
```

```
  __pyx_t_4 = __Pyx_GetItemInt(__pyx_v_v_config, -1L, long, 1, __Pyx_PyInt_From_long, 0, 1, 1); if (unlikely(!__pyx_t_4)) __PYX_ERR(0, 115, __pyx_L1_error)
  __Pyx_GOTREF(__pyx_t_4);
  if (PyDict_SetItem(__pyx_t_2, __pyx_n_s_d_lambda, __pyx_t_4) < 0) __PYX_ERR(0, 111, __pyx_L1_error)
  __Pyx_DECREF(__pyx_t_4); __pyx_t_4 = 0;
```

```
 116:
```

```
 117:
```

```
+118: def fn_dc_objective(v_channel, nda_interf, v_config):
```

```
/* Python wrapper */
static PyObject *__pyx_pw_9dist_recv_9opt_rates_9fn_dc_objective(PyObject *__pyx_self, PyObject *__pyx_args, PyObject *__pyx_kwds); /*proto*/
static PyMethodDef __pyx_mdef_9dist_recv_9opt_rates_9fn_dc_objective = {"fn_dc_objective", (PyCFunction)__pyx_pw_9dist_recv_9opt_rates_9fn_dc_objective, METH_VARARGS|METH_KEYWORDS, 0};
static PyObject *__pyx_pw_9dist_recv_9opt_rates_9fn_dc_objective(PyObject *__pyx_self, PyObject *__pyx_args, PyObject *__pyx_kwds) {
  PyObject *__pyx_v_v_channel = 0;
  PyObject *__pyx_v_nda_interf = 0;
  PyObject *__pyx_v_v_config = 0;
  PyObject *__pyx_r = 0;
  __Pyx_RefNannyDeclarations
  __Pyx_RefNannySetupContext("fn_dc_objective (wrapper)", 0);
  {
    static PyObject **__pyx_pyargnames[] = {&__pyx_n_s_v_channel,&__pyx_n_s_nda_interf,&__pyx_n_s_v_config,0};
    PyObject* values[3] = {0,0,0};
    if (unlikely(__pyx_kwds)) {
      Py_ssize_t kw_args;
      const Py_ssize_t pos_args = PyTuple_GET_SIZE(__pyx_args);
      switch (pos_args) {
        case  3: values[2] = PyTuple_GET_ITEM(__pyx_args, 2);
        case  2: values[1] = PyTuple_GET_ITEM(__pyx_args, 1);
        case  1: values[0] = PyTuple_GET_ITEM(__pyx_args, 0);
        case  0: break;
        default: goto __pyx_L5_argtuple_error;
      }
      kw_args = PyDict_Size(__pyx_kwds);
      switch (pos_args) {
        case  0:
        if (likely((values[0] = PyDict_GetItem(__pyx_kwds, __pyx_n_s_v_channel)) != 0)) kw_args--;
        else goto __pyx_L5_argtuple_error;
        case  1:
        if (likely((values[1] = PyDict_GetItem(__pyx_kwds, __pyx_n_s_nda_interf)) != 0)) kw_args--;
        else {
          __Pyx_RaiseArgtupleInvalid("fn_dc_objective", 1, 3, 3, 1); __PYX_ERR(0, 118, __pyx_L3_error)
        }
        case  2:
        if (likely((values[2] = PyDict_GetItem(__pyx_kwds, __pyx_n_s_v_config)) != 0)) kw_args--;
        else {
          __Pyx_RaiseArgtupleInvalid("fn_dc_objective", 1, 3, 3, 2); __PYX_ERR(0, 118, __pyx_L3_error)
        }
      }
      if (unlikely(kw_args > 0)) {
        if (unlikely(__Pyx_ParseOptionalKeywords(__pyx_kwds, __pyx_pyargnames, 0, values, pos_args, "fn_dc_objective") < 0)) __PYX_ERR(0, 118, __pyx_L3_error)
      }
    } else if (PyTuple_GET_SIZE(__pyx_args) != 3) {
      goto __pyx_L5_argtuple_error;
    } else {
      values[0] = PyTuple_GET_ITEM(__pyx_args, 0);
      values[1] = PyTuple_GET_ITEM(__pyx_args, 1);
      values[2] = PyTuple_GET_ITEM(__pyx_args, 2);
    }
    __pyx_v_v_channel = values[0];
    __pyx_v_nda_interf = values[1];
    __pyx_v_v_config = values[2];
  }
  goto __pyx_L4_argument_unpacking_done;
  __pyx_L5_argtuple_error:;
  __Pyx_RaiseArgtupleInvalid("fn_dc_objective", 1, 3, 3, PyTuple_GET_SIZE(__pyx_args)); __PYX_ERR(0, 118, __pyx_L3_error)
  __pyx_L3_error:;
  __Pyx_AddTraceback("dist_recv.opt_rates.fn_dc_objective", __pyx_clineno, __pyx_lineno, __pyx_filename);
  __Pyx_RefNannyFinishContext();
  return NULL;
  __pyx_L4_argument_unpacking_done:;
  __pyx_r = __pyx_pf_9dist_recv_9opt_rates_8fn_dc_objective(__pyx_self, __pyx_v_v_channel, __pyx_v_nda_interf, __pyx_v_v_config);

  /* function exit code */
  __Pyx_RefNannyFinishContext();
  return __pyx_r;
}

static PyObject *__pyx_pf_9dist_recv_9opt_rates_8fn_dc_objective(CYTHON_UNUSED PyObject *__pyx_self, PyObject *__pyx_v_v_channel, PyObject *__pyx_v_nda_interf, PyObject *__pyx_v_v_config) {
  PyObject *__pyx_v_n_helpers = NULL;
  PyObject *__pyx_v_d_rate = NULL;
  PyObject *__pyx_v_d_dc_obj = NULL;
  PyObject *__pyx_r = NULL;
  __Pyx_TraceDeclarations
  __Pyx_TraceFrameInit(__pyx_codeobj__6)
  __Pyx_RefNannyDeclarations
  __Pyx_RefNannySetupContext("fn_dc_objective", 0);
  __Pyx_TraceCall("fn_dc_objective", __pyx_f[0], 118, 0, __PYX_ERR(0, 118, __pyx_L1_error));
/* … */
  /* function exit code */
  __pyx_L1_error:;
  __Pyx_XDECREF(__pyx_t_1);
  __Pyx_XDECREF(__pyx_t_2);
  __Pyx_XDECREF(__pyx_t_3);
  __Pyx_XDECREF(__pyx_t_4);
  __Pyx_AddTraceback("dist_recv.opt_rates.fn_dc_objective", __pyx_clineno, __pyx_lineno, __pyx_filename);
  __pyx_r = NULL;
  __pyx_L0:;
  __Pyx_XDECREF(__pyx_v_n_helpers);
  __Pyx_XDECREF(__pyx_v_d_rate);
  __Pyx_XDECREF(__pyx_v_d_dc_obj);
  __Pyx_XGIVEREF(__pyx_r);
  __Pyx_TraceReturn(__pyx_r, 0);
  __Pyx_RefNannyFinishContext();
  return __pyx_r;
}
/* … */
  __pyx_tuple__11 = PyTuple_Pack(6, __pyx_n_s_v_channel, __pyx_n_s_nda_interf, __pyx_n_s_v_config, __pyx_n_s_n_helpers, __pyx_n_s_d_rate, __pyx_n_s_d_dc_obj); if (unlikely(!__pyx_tuple__11)) __PYX_ERR(0, 118, __pyx_L1_error)
  __Pyx_GOTREF(__pyx_tuple__11);
  __Pyx_GIVEREF(__pyx_tuple__11);
/* … */
  __pyx_t_2 = PyCFunction_NewEx(&__pyx_mdef_9dist_recv_9opt_rates_9fn_dc_objective, NULL, __pyx_n_s_dist_recv_opt_rates); if (unlikely(!__pyx_t_2)) __PYX_ERR(0, 118, __pyx_L1_error)
  __Pyx_GOTREF(__pyx_t_2);
  if (PyDict_SetItem(__pyx_d, __pyx_n_s_fn_dc_objective, __pyx_t_2) < 0) __PYX_ERR(0, 118, __pyx_L1_error)
  __Pyx_DECREF(__pyx_t_2); __pyx_t_2 = 0;
```

```
+119:     if(sum(np.isnan(v_config))) > 0:
```

```
  __pyx_t_2 = __Pyx_GetModuleGlobalName(__pyx_n_s_np); if (unlikely(!__pyx_t_2)) __PYX_ERR(0, 119, __pyx_L1_error)
  __Pyx_GOTREF(__pyx_t_2);
  __pyx_t_3 = __Pyx_PyObject_GetAttrStr(__pyx_t_2, __pyx_n_s_isnan); if (unlikely(!__pyx_t_3)) __PYX_ERR(0, 119, __pyx_L1_error)
  __Pyx_GOTREF(__pyx_t_3);
  __Pyx_DECREF(__pyx_t_2); __pyx_t_2 = 0;
  __pyx_t_2 = NULL;
  if (CYTHON_UNPACK_METHODS && unlikely(PyMethod_Check(__pyx_t_3))) {
    __pyx_t_2 = PyMethod_GET_SELF(__pyx_t_3);
    if (likely(__pyx_t_2)) {
      PyObject* function = PyMethod_GET_FUNCTION(__pyx_t_3);
      __Pyx_INCREF(__pyx_t_2);
      __Pyx_INCREF(function);
      __Pyx_DECREF_SET(__pyx_t_3, function);
    }
  }
  if (!__pyx_t_2) {
    __pyx_t_1 = __Pyx_PyObject_CallOneArg(__pyx_t_3, __pyx_v_v_config); if (unlikely(!__pyx_t_1)) __PYX_ERR(0, 119, __pyx_L1_error)
    __Pyx_GOTREF(__pyx_t_1);
  } else {
    #if CYTHON_FAST_PYCALL
    if (PyFunction_Check(__pyx_t_3)) {
      PyObject *__pyx_temp[2] = {__pyx_t_2, __pyx_v_v_config};
      __pyx_t_1 = __Pyx_PyFunction_FastCall(__pyx_t_3, __pyx_temp+1-1, 1+1); if (unlikely(!__pyx_t_1)) __PYX_ERR(0, 119, __pyx_L1_error)
      __Pyx_XDECREF(__pyx_t_2); __pyx_t_2 = 0;
      __Pyx_GOTREF(__pyx_t_1);
    } else
    #endif
    #if CYTHON_FAST_PYCCALL
    if (__Pyx_PyFastCFunction_Check(__pyx_t_3)) {
      PyObject *__pyx_temp[2] = {__pyx_t_2, __pyx_v_v_config};
      __pyx_t_1 = __Pyx_PyCFunction_FastCall(__pyx_t_3, __pyx_temp+1-1, 1+1); if (unlikely(!__pyx_t_1)) __PYX_ERR(0, 119, __pyx_L1_error)
      __Pyx_XDECREF(__pyx_t_2); __pyx_t_2 = 0;
      __Pyx_GOTREF(__pyx_t_1);
    } else
    #endif
    {
      __pyx_t_4 = PyTuple_New(1+1); if (unlikely(!__pyx_t_4)) __PYX_ERR(0, 119, __pyx_L1_error)
      __Pyx_GOTREF(__pyx_t_4);
      __Pyx_GIVEREF(__pyx_t_2); PyTuple_SET_ITEM(__pyx_t_4, 0, __pyx_t_2); __pyx_t_2 = NULL;
      __Pyx_INCREF(__pyx_v_v_config);
      __Pyx_GIVEREF(__pyx_v_v_config);
      PyTuple_SET_ITEM(__pyx_t_4, 0+1, __pyx_v_v_config);
      __pyx_t_1 = __Pyx_PyObject_Call(__pyx_t_3, __pyx_t_4, NULL); if (unlikely(!__pyx_t_1)) __PYX_ERR(0, 119, __pyx_L1_error)
      __Pyx_GOTREF(__pyx_t_1);
      __Pyx_DECREF(__pyx_t_4); __pyx_t_4 = 0;
    }
  }
  __Pyx_DECREF(__pyx_t_3); __pyx_t_3 = 0;
  __pyx_t_3 = PyTuple_New(1); if (unlikely(!__pyx_t_3)) __PYX_ERR(0, 119, __pyx_L1_error)
  __Pyx_GOTREF(__pyx_t_3);
  __Pyx_GIVEREF(__pyx_t_1);
  PyTuple_SET_ITEM(__pyx_t_3, 0, __pyx_t_1);
  __pyx_t_1 = 0;
  __pyx_t_1 = __Pyx_PyObject_Call(__pyx_builtin_sum, __pyx_t_3, NULL); if (unlikely(!__pyx_t_1)) __PYX_ERR(0, 119, __pyx_L1_error)
  __Pyx_GOTREF(__pyx_t_1);
  __Pyx_DECREF(__pyx_t_3); __pyx_t_3 = 0;
  __pyx_t_3 = PyObject_RichCompare(__pyx_t_1, __pyx_int_0, Py_GT); __Pyx_XGOTREF(__pyx_t_3); if (unlikely(!__pyx_t_3)) __PYX_ERR(0, 119, __pyx_L1_error)
  __Pyx_DECREF(__pyx_t_1); __pyx_t_1 = 0;
  __pyx_t_5 = __Pyx_PyObject_IsTrue(__pyx_t_3); if (unlikely(__pyx_t_5 < 0)) __PYX_ERR(0, 119, __pyx_L1_error)
  __Pyx_DECREF(__pyx_t_3); __pyx_t_3 = 0;
  if (__pyx_t_5) {
/* … */
  }
```

```
+120:         return float('inf')
```

```
    __Pyx_XDECREF(__pyx_r);
    __pyx_t_3 = __Pyx_PyNumber_Float(__pyx_n_s_inf); if (unlikely(!__pyx_t_3)) __PYX_ERR(0, 120, __pyx_L1_error)
    __Pyx_GOTREF(__pyx_t_3);
    __pyx_r = __pyx_t_3;
    __pyx_t_3 = 0;
    goto __pyx_L0;
```

```
+121:     n_helpers = v_channel.shape[0]-1
```

```
  __pyx_t_3 = __Pyx_PyObject_GetAttrStr(__pyx_v_v_channel, __pyx_n_s_shape); if (unlikely(!__pyx_t_3)) __PYX_ERR(0, 121, __pyx_L1_error)
  __Pyx_GOTREF(__pyx_t_3);
  __pyx_t_1 = __Pyx_GetItemInt(__pyx_t_3, 0, long, 1, __Pyx_PyInt_From_long, 0, 0, 1); if (unlikely(!__pyx_t_1)) __PYX_ERR(0, 121, __pyx_L1_error)
  __Pyx_GOTREF(__pyx_t_1);
  __Pyx_DECREF(__pyx_t_3); __pyx_t_3 = 0;
  __pyx_t_3 = __Pyx_PyInt_SubtractObjC(__pyx_t_1, __pyx_int_1, 1, 0); if (unlikely(!__pyx_t_3)) __PYX_ERR(0, 121, __pyx_L1_error)
  __Pyx_GOTREF(__pyx_t_3);
  __Pyx_DECREF(__pyx_t_1); __pyx_t_1 = 0;
  __pyx_v_n_helpers = __pyx_t_3;
  __pyx_t_3 = 0;
```

```
+122:     d_rate = rates.rate_ga(v_channel, nda_interf, v_bits=v_config[n_helpers:-1])
```

```
  __pyx_t_3 = __Pyx_GetModuleGlobalName(__pyx_n_s_rates); if (unlikely(!__pyx_t_3)) __PYX_ERR(0, 122, __pyx_L1_error)
  __Pyx_GOTREF(__pyx_t_3);
  __pyx_t_1 = __Pyx_PyObject_GetAttrStr(__pyx_t_3, __pyx_n_s_rate_ga); if (unlikely(!__pyx_t_1)) __PYX_ERR(0, 122, __pyx_L1_error)
  __Pyx_GOTREF(__pyx_t_1);
  __Pyx_DECREF(__pyx_t_3); __pyx_t_3 = 0;
  __pyx_t_3 = PyTuple_New(2); if (unlikely(!__pyx_t_3)) __PYX_ERR(0, 122, __pyx_L1_error)
  __Pyx_GOTREF(__pyx_t_3);
  __Pyx_INCREF(__pyx_v_v_channel);
  __Pyx_GIVEREF(__pyx_v_v_channel);
  PyTuple_SET_ITEM(__pyx_t_3, 0, __pyx_v_v_channel);
  __Pyx_INCREF(__pyx_v_nda_interf);
  __Pyx_GIVEREF(__pyx_v_nda_interf);
  PyTuple_SET_ITEM(__pyx_t_3, 1, __pyx_v_nda_interf);
  __pyx_t_4 = PyDict_New(); if (unlikely(!__pyx_t_4)) __PYX_ERR(0, 122, __pyx_L1_error)
  __Pyx_GOTREF(__pyx_t_4);
  __pyx_t_2 = __Pyx_PyObject_GetSlice(__pyx_v_v_config, 0, -1L, &__pyx_v_n_helpers, NULL, NULL, 0, 1, 1); if (unlikely(!__pyx_t_2)) __PYX_ERR(0, 122, __pyx_L1_error)
  __Pyx_GOTREF(__pyx_t_2);
  if (PyDict_SetItem(__pyx_t_4, __pyx_n_s_v_bits, __pyx_t_2) < 0) __PYX_ERR(0, 122, __pyx_L1_error)
  __Pyx_DECREF(__pyx_t_2); __pyx_t_2 = 0;
  __pyx_t_2 = __Pyx_PyObject_Call(__pyx_t_1, __pyx_t_3, __pyx_t_4); if (unlikely(!__pyx_t_2)) __PYX_ERR(0, 122, __pyx_L1_error)
  __Pyx_GOTREF(__pyx_t_2);
  __Pyx_DECREF(__pyx_t_1); __pyx_t_1 = 0;
  __Pyx_DECREF(__pyx_t_3); __pyx_t_3 = 0;
  __Pyx_DECREF(__pyx_t_4); __pyx_t_4 = 0;
  __pyx_v_d_rate = __pyx_t_2;
  __pyx_t_2 = 0;
```

```
+123:     d_dc_obj = -(d_rate - v_config[-1])
```

```
  __pyx_t_2 = __Pyx_GetItemInt(__pyx_v_v_config, -1L, long, 1, __Pyx_PyInt_From_long, 0, 1, 1); if (unlikely(!__pyx_t_2)) __PYX_ERR(0, 123, __pyx_L1_error)
  __Pyx_GOTREF(__pyx_t_2);
  __pyx_t_4 = PyNumber_Subtract(__pyx_v_d_rate, __pyx_t_2); if (unlikely(!__pyx_t_4)) __PYX_ERR(0, 123, __pyx_L1_error)
  __Pyx_GOTREF(__pyx_t_4);
  __Pyx_DECREF(__pyx_t_2); __pyx_t_2 = 0;
  __pyx_t_2 = PyNumber_Negative(__pyx_t_4); if (unlikely(!__pyx_t_2)) __PYX_ERR(0, 123, __pyx_L1_error)
  __Pyx_GOTREF(__pyx_t_2);
  __Pyx_DECREF(__pyx_t_4); __pyx_t_4 = 0;
  __pyx_v_d_dc_obj = __pyx_t_2;
  __pyx_t_2 = 0;
```

```
+124:     return d_dc_obj
```

```
  __Pyx_XDECREF(__pyx_r);
  __Pyx_INCREF(__pyx_v_d_dc_obj);
  __pyx_r = __pyx_v_d_dc_obj;
  goto __pyx_L0;
```
