# Supplementary material for: A Decentralized Receiver in Gaussian Interference
Source: Entropy (Basel). 2018 Apr 11;20(4):269. doi: 10.3390/e20040269 (PMC7512784; doi:10.3390/e20040269)
Supplement: Supplementary file 1 [file entropy-20-00269-s001.zip › dist_recv/rates.html]

Cython: rates.pyx


Generated by Cython 0.25.2

Yellow lines hint at Python interaction.  
Click on a line that starts with a "`+`" to see the C code that Cython generated for it.

Raw output: rates.c

```
 001: """Rates for Distributed Receive"""
```

```
+002: import numpy as np
```

```
  __pyx_t_1 = __Pyx_Import(__pyx_n_s_numpy, 0, -1); if (unlikely(!__pyx_t_1)) __PYX_ERR(0, 2, __pyx_L1_error)
  __Pyx_GOTREF(__pyx_t_1);
  if (PyDict_SetItem(__pyx_d, __pyx_n_s_np, __pyx_t_1) < 0) __PYX_ERR(0, 2, __pyx_L1_error)
  __Pyx_DECREF(__pyx_t_1); __pyx_t_1 = 0;
```

```
 003:
```

```
+004: from dist_recv import checkdc as dc
```

```
  __pyx_t_1 = PyList_New(1); if (unlikely(!__pyx_t_1)) __PYX_ERR(0, 4, __pyx_L1_error)
  __Pyx_GOTREF(__pyx_t_1);
  __Pyx_INCREF(__pyx_n_s_checkdc);
  __Pyx_GIVEREF(__pyx_n_s_checkdc);
  PyList_SET_ITEM(__pyx_t_1, 0, __pyx_n_s_checkdc);
  __pyx_t_2 = __Pyx_Import(__pyx_n_s_dist_recv, __pyx_t_1, -1); if (unlikely(!__pyx_t_2)) __PYX_ERR(0, 4, __pyx_L1_error)
  __Pyx_GOTREF(__pyx_t_2);
  __Pyx_DECREF(__pyx_t_1); __pyx_t_1 = 0;
  __pyx_t_1 = __Pyx_ImportFrom(__pyx_t_2, __pyx_n_s_checkdc); if (unlikely(!__pyx_t_1)) __PYX_ERR(0, 4, __pyx_L1_error)
  __Pyx_GOTREF(__pyx_t_1);
  if (PyDict_SetItem(__pyx_d, __pyx_n_s_dc, __pyx_t_1) < 0) __PYX_ERR(0, 4, __pyx_L1_error)
  __Pyx_DECREF(__pyx_t_1); __pyx_t_1 = 0;
  __Pyx_DECREF(__pyx_t_2); __pyx_t_2 = 0;
```

```
 005:
```

```
+006: D_MIN_USEFUL_BITRATE = 0.01
```

```
  if (PyDict_SetItem(__pyx_d, __pyx_n_s_D_MIN_USEFUL_BITRATE, __pyx_float_0_01) < 0) __PYX_ERR(0, 6, __pyx_L1_error)
```

```
 007:
```

```
 008:
```

```
+009: def rate_ub(v_channel, nda_interf, d_bits):
```

```
/* Python wrapper */
static PyObject *__pyx_pw_9dist_recv_5rates_1rate_ub(PyObject *__pyx_self, PyObject *__pyx_args, PyObject *__pyx_kwds); /*proto*/
static char __pyx_doc_9dist_recv_5rates_rate_ub[] = " Upper bound on rate for distributed receive with reception at base\n        Input:\n            v_channel = Channel vector h (size N+1 x 1)\n            nda_interf = Interference covariance matrix Sigma (size N+1 x N+1)\n            d_bits = Total amount of helper bits\n        Output:\n            d_rate = Rate (in bits)\n    ";
static PyMethodDef __pyx_mdef_9dist_recv_5rates_1rate_ub = {"rate_ub", (PyCFunction)__pyx_pw_9dist_recv_5rates_1rate_ub, METH_VARARGS|METH_KEYWORDS, __pyx_doc_9dist_recv_5rates_rate_ub};
static PyObject *__pyx_pw_9dist_recv_5rates_1rate_ub(PyObject *__pyx_self, PyObject *__pyx_args, PyObject *__pyx_kwds) {
  PyObject *__pyx_v_v_channel = 0;
  PyObject *__pyx_v_nda_interf = 0;
  PyObject *__pyx_v_d_bits = 0;
  PyObject *__pyx_r = 0;
  __Pyx_RefNannyDeclarations
  __Pyx_RefNannySetupContext("rate_ub (wrapper)", 0);
  {
    static PyObject **__pyx_pyargnames[] = {&__pyx_n_s_v_channel,&__pyx_n_s_nda_interf,&__pyx_n_s_d_bits,0};
    PyObject* values[3] = {0,0,0};
    if (unlikely(__pyx_kwds)) {
      Py_ssize_t kw_args;
      const Py_ssize_t pos_args = PyTuple_GET_SIZE(__pyx_args);
      switch (pos_args) {
        case  3: values[2] = PyTuple_GET_ITEM(__pyx_args, 2);
        case  2: values[1] = PyTuple_GET_ITEM(__pyx_args, 1);
        case  1: values[0] = PyTuple_GET_ITEM(__pyx_args, 0);
        case  0: break;
        default: goto __pyx_L5_argtuple_error;
      }
      kw_args = PyDict_Size(__pyx_kwds);
      switch (pos_args) {
        case  0:
        if (likely((values[0] = PyDict_GetItem(__pyx_kwds, __pyx_n_s_v_channel)) != 0)) kw_args--;
        else goto __pyx_L5_argtuple_error;
        case  1:
        if (likely((values[1] = PyDict_GetItem(__pyx_kwds, __pyx_n_s_nda_interf)) != 0)) kw_args--;
        else {
          __Pyx_RaiseArgtupleInvalid("rate_ub", 1, 3, 3, 1); __PYX_ERR(0, 9, __pyx_L3_error)
        }
        case  2:
        if (likely((values[2] = PyDict_GetItem(__pyx_kwds, __pyx_n_s_d_bits)) != 0)) kw_args--;
        else {
          __Pyx_RaiseArgtupleInvalid("rate_ub", 1, 3, 3, 2); __PYX_ERR(0, 9, __pyx_L3_error)
        }
      }
      if (unlikely(kw_args > 0)) {
        if (unlikely(__Pyx_ParseOptionalKeywords(__pyx_kwds, __pyx_pyargnames, 0, values, pos_args, "rate_ub") < 0)) __PYX_ERR(0, 9, __pyx_L3_error)
      }
    } else if (PyTuple_GET_SIZE(__pyx_args) != 3) {
      goto __pyx_L5_argtuple_error;
    } else {
      values[0] = PyTuple_GET_ITEM(__pyx_args, 0);
      values[1] = PyTuple_GET_ITEM(__pyx_args, 1);
      values[2] = PyTuple_GET_ITEM(__pyx_args, 2);
    }
    __pyx_v_v_channel = values[0];
    __pyx_v_nda_interf = values[1];
    __pyx_v_d_bits = values[2];
  }
  goto __pyx_L4_argument_unpacking_done;
  __pyx_L5_argtuple_error:;
  __Pyx_RaiseArgtupleInvalid("rate_ub", 1, 3, 3, PyTuple_GET_SIZE(__pyx_args)); __PYX_ERR(0, 9, __pyx_L3_error)
  __pyx_L3_error:;
  __Pyx_AddTraceback("dist_recv.rates.rate_ub", __pyx_clineno, __pyx_lineno, __pyx_filename);
  __Pyx_RefNannyFinishContext();
  return NULL;
  __pyx_L4_argument_unpacking_done:;
  __pyx_r = __pyx_pf_9dist_recv_5rates_rate_ub(__pyx_self, __pyx_v_v_channel, __pyx_v_nda_interf, __pyx_v_d_bits);

  /* function exit code */
  __Pyx_RefNannyFinishContext();
  return __pyx_r;
}

static PyObject *__pyx_pf_9dist_recv_5rates_rate_ub(CYTHON_UNUSED PyObject *__pyx_self, PyObject *__pyx_v_v_channel, PyObject *__pyx_v_nda_interf, PyObject *__pyx_v_d_bits) {
  PyObject *__pyx_v_n_recv = NULL;
  PyObject *__pyx_v_nda_receiver_noise = NULL;
  PyObject *__pyx_v_nda_receiver_signal = NULL;
  PyObject *__pyx_v_d_rate = NULL;
  PyObject *__pyx_v_d_rate_0 = NULL;
  PyObject *__pyx_r = NULL;
  __Pyx_TraceDeclarations
  __Pyx_TraceFrameInit(__pyx_codeobj_)
  __Pyx_RefNannyDeclarations
  __Pyx_RefNannySetupContext("rate_ub", 0);
  __Pyx_TraceCall("rate_ub", __pyx_f[0], 9, 0, __PYX_ERR(0, 9, __pyx_L1_error));
/* … */
  /* function exit code */
  __pyx_L1_error:;
  __Pyx_XDECREF(__pyx_t_1);
  __Pyx_XDECREF(__pyx_t_2);
  __Pyx_XDECREF(__pyx_t_3);
  __Pyx_XDECREF(__pyx_t_4);
  __Pyx_XDECREF(__pyx_t_5);
  __Pyx_XDECREF(__pyx_t_7);
  __Pyx_XDECREF(__pyx_t_8);
  __Pyx_XDECREF(__pyx_t_9);
  __Pyx_XDECREF(__pyx_t_10);
  __Pyx_XDECREF(__pyx_t_11);
  __Pyx_AddTraceback("dist_recv.rates.rate_ub", __pyx_clineno, __pyx_lineno, __pyx_filename);
  __pyx_r = NULL;
  __pyx_L0:;
  __Pyx_XDECREF(__pyx_v_n_recv);
  __Pyx_XDECREF(__pyx_v_nda_receiver_noise);
  __Pyx_XDECREF(__pyx_v_nda_receiver_signal);
  __Pyx_XDECREF(__pyx_v_d_rate);
  __Pyx_XDECREF(__pyx_v_d_rate_0);
  __Pyx_XGIVEREF(__pyx_r);
  __Pyx_TraceReturn(__pyx_r, 0);
  __Pyx_RefNannyFinishContext();
  return __pyx_r;
}
/* … */
  __pyx_tuple__12 = PyTuple_Pack(8, __pyx_n_s_v_channel, __pyx_n_s_nda_interf, __pyx_n_s_d_bits, __pyx_n_s_n_recv, __pyx_n_s_nda_receiver_noise, __pyx_n_s_nda_receiver_signal, __pyx_n_s_d_rate, __pyx_n_s_d_rate_0); if (unlikely(!__pyx_tuple__12)) __PYX_ERR(0, 9, __pyx_L1_error)
  __Pyx_GOTREF(__pyx_tuple__12);
  __Pyx_GIVEREF(__pyx_tuple__12);
/* … */
  __pyx_t_2 = PyCFunction_NewEx(&__pyx_mdef_9dist_recv_5rates_1rate_ub, NULL, __pyx_n_s_dist_recv_rates); if (unlikely(!__pyx_t_2)) __PYX_ERR(0, 9, __pyx_L1_error)
  __Pyx_GOTREF(__pyx_t_2);
  if (PyDict_SetItem(__pyx_d, __pyx_n_s_rate_ub, __pyx_t_2) < 0) __PYX_ERR(0, 9, __pyx_L1_error)
  __Pyx_DECREF(__pyx_t_2); __pyx_t_2 = 0;
  __pyx_codeobj_ = (PyObject*)__Pyx_PyCode_New(3, 0, 8, 0, 0, __pyx_empty_bytes, __pyx_empty_tuple, __pyx_empty_tuple, __pyx_tuple__12, __pyx_empty_tuple, __pyx_empty_tuple, __pyx_kp_s_Users_cdchapm2_Dropbox_Personal, __pyx_n_s_rate_ub, 9, __pyx_empty_bytes); if (unlikely(!__pyx_codeobj_)) __PYX_ERR(0, 9, __pyx_L1_error)
```

```
 010:     """ Upper bound on rate for distributed receive with reception at base
```

```
 011:         Input:
```

```
 012:             v_channel = Channel vector h (size N+1 x 1)
```

```
 013:             nda_interf = Interference covariance matrix Sigma (size N+1 x N+1)
```

```
 014:             d_bits = Total amount of helper bits
```

```
 015:         Output:
```

```
 016:             d_rate = Rate (in bits)
```

```
 017:     """
```

```
+018:     n_recv = v_channel.shape[0]
```

```
  __pyx_t_1 = __Pyx_PyObject_GetAttrStr(__pyx_v_v_channel, __pyx_n_s_shape); if (unlikely(!__pyx_t_1)) __PYX_ERR(0, 18, __pyx_L1_error)
  __Pyx_GOTREF(__pyx_t_1);
  __pyx_t_2 = __Pyx_GetItemInt(__pyx_t_1, 0, long, 1, __Pyx_PyInt_From_long, 0, 0, 1); if (unlikely(!__pyx_t_2)) __PYX_ERR(0, 18, __pyx_L1_error)
  __Pyx_GOTREF(__pyx_t_2);
  __Pyx_DECREF(__pyx_t_1); __pyx_t_1 = 0;
  __pyx_v_n_recv = __pyx_t_2;
  __pyx_t_2 = 0;
```

```
 019:
```

```
 020:     # Receiver thermal noise + interference covariance
```

```
+021:     nda_receiver_noise = nda_interf + np.identity(n_recv)
```

```
  __pyx_t_1 = __Pyx_GetModuleGlobalName(__pyx_n_s_np); if (unlikely(!__pyx_t_1)) __PYX_ERR(0, 21, __pyx_L1_error)
  __Pyx_GOTREF(__pyx_t_1);
  __pyx_t_3 = __Pyx_PyObject_GetAttrStr(__pyx_t_1, __pyx_n_s_identity); if (unlikely(!__pyx_t_3)) __PYX_ERR(0, 21, __pyx_L1_error)
  __Pyx_GOTREF(__pyx_t_3);
  __Pyx_DECREF(__pyx_t_1); __pyx_t_1 = 0;
  __pyx_t_1 = NULL;
  if (CYTHON_UNPACK_METHODS && unlikely(PyMethod_Check(__pyx_t_3))) {
    __pyx_t_1 = PyMethod_GET_SELF(__pyx_t_3);
    if (likely(__pyx_t_1)) {
      PyObject* function = PyMethod_GET_FUNCTION(__pyx_t_3);
      __Pyx_INCREF(__pyx_t_1);
      __Pyx_INCREF(function);
      __Pyx_DECREF_SET(__pyx_t_3, function);
    }
  }
  if (!__pyx_t_1) {
    __pyx_t_2 = __Pyx_PyObject_CallOneArg(__pyx_t_3, __pyx_v_n_recv); if (unlikely(!__pyx_t_2)) __PYX_ERR(0, 21, __pyx_L1_error)
    __Pyx_GOTREF(__pyx_t_2);
  } else {
    #if CYTHON_FAST_PYCALL
    if (PyFunction_Check(__pyx_t_3)) {
      PyObject *__pyx_temp[2] = {__pyx_t_1, __pyx_v_n_recv};
      __pyx_t_2 = __Pyx_PyFunction_FastCall(__pyx_t_3, __pyx_temp+1-1, 1+1); if (unlikely(!__pyx_t_2)) __PYX_ERR(0, 21, __pyx_L1_error)
      __Pyx_XDECREF(__pyx_t_1); __pyx_t_1 = 0;
      __Pyx_GOTREF(__pyx_t_2);
    } else
    #endif
    #if CYTHON_FAST_PYCCALL
    if (__Pyx_PyFastCFunction_Check(__pyx_t_3)) {
      PyObject *__pyx_temp[2] = {__pyx_t_1, __pyx_v_n_recv};
      __pyx_t_2 = __Pyx_PyCFunction_FastCall(__pyx_t_3, __pyx_temp+1-1, 1+1); if (unlikely(!__pyx_t_2)) __PYX_ERR(0, 21, __pyx_L1_error)
      __Pyx_XDECREF(__pyx_t_1); __pyx_t_1 = 0;
      __Pyx_GOTREF(__pyx_t_2);
    } else
    #endif
    {
      __pyx_t_4 = PyTuple_New(1+1); if (unlikely(!__pyx_t_4)) __PYX_ERR(0, 21, __pyx_L1_error)
      __Pyx_GOTREF(__pyx_t_4);
      __Pyx_GIVEREF(__pyx_t_1); PyTuple_SET_ITEM(__pyx_t_4, 0, __pyx_t_1); __pyx_t_1 = NULL;
      __Pyx_INCREF(__pyx_v_n_recv);
      __Pyx_GIVEREF(__pyx_v_n_recv);
      PyTuple_SET_ITEM(__pyx_t_4, 0+1, __pyx_v_n_recv);
      __pyx_t_2 = __Pyx_PyObject_Call(__pyx_t_3, __pyx_t_4, NULL); if (unlikely(!__pyx_t_2)) __PYX_ERR(0, 21, __pyx_L1_error)
      __Pyx_GOTREF(__pyx_t_2);
      __Pyx_DECREF(__pyx_t_4); __pyx_t_4 = 0;
    }
  }
  __Pyx_DECREF(__pyx_t_3); __pyx_t_3 = 0;
  __pyx_t_3 = PyNumber_Add(__pyx_v_nda_interf, __pyx_t_2); if (unlikely(!__pyx_t_3)) __PYX_ERR(0, 21, __pyx_L1_error)
  __Pyx_GOTREF(__pyx_t_3);
  __Pyx_DECREF(__pyx_t_2); __pyx_t_2 = 0;
  __pyx_v_nda_receiver_noise = __pyx_t_3;
  __pyx_t_3 = 0;
```

```
 022:
```

```
 023:     # Receive signal covariance
```

```
+024:     nda_receiver_signal = np.outer(v_channel, v_channel.conj())
```

```
  __pyx_t_2 = __Pyx_GetModuleGlobalName(__pyx_n_s_np); if (unlikely(!__pyx_t_2)) __PYX_ERR(0, 24, __pyx_L1_error)
  __Pyx_GOTREF(__pyx_t_2);
  __pyx_t_4 = __Pyx_PyObject_GetAttrStr(__pyx_t_2, __pyx_n_s_outer); if (unlikely(!__pyx_t_4)) __PYX_ERR(0, 24, __pyx_L1_error)
  __Pyx_GOTREF(__pyx_t_4);
  __Pyx_DECREF(__pyx_t_2); __pyx_t_2 = 0;
  __pyx_t_1 = __Pyx_PyObject_GetAttrStr(__pyx_v_v_channel, __pyx_n_s_conj); if (unlikely(!__pyx_t_1)) __PYX_ERR(0, 24, __pyx_L1_error)
  __Pyx_GOTREF(__pyx_t_1);
  __pyx_t_5 = NULL;
  if (CYTHON_UNPACK_METHODS && likely(PyMethod_Check(__pyx_t_1))) {
    __pyx_t_5 = PyMethod_GET_SELF(__pyx_t_1);
    if (likely(__pyx_t_5)) {
      PyObject* function = PyMethod_GET_FUNCTION(__pyx_t_1);
      __Pyx_INCREF(__pyx_t_5);
      __Pyx_INCREF(function);
      __Pyx_DECREF_SET(__pyx_t_1, function);
    }
  }
  if (__pyx_t_5) {
    __pyx_t_2 = __Pyx_PyObject_CallOneArg(__pyx_t_1, __pyx_t_5); if (unlikely(!__pyx_t_2)) __PYX_ERR(0, 24, __pyx_L1_error)
    __Pyx_DECREF(__pyx_t_5); __pyx_t_5 = 0;
  } else {
    __pyx_t_2 = __Pyx_PyObject_CallNoArg(__pyx_t_1); if (unlikely(!__pyx_t_2)) __PYX_ERR(0, 24, __pyx_L1_error)
  }
  __Pyx_GOTREF(__pyx_t_2);
  __Pyx_DECREF(__pyx_t_1); __pyx_t_1 = 0;
  __pyx_t_1 = NULL;
  __pyx_t_6 = 0;
  if (CYTHON_UNPACK_METHODS && unlikely(PyMethod_Check(__pyx_t_4))) {
    __pyx_t_1 = PyMethod_GET_SELF(__pyx_t_4);
    if (likely(__pyx_t_1)) {
      PyObject* function = PyMethod_GET_FUNCTION(__pyx_t_4);
      __Pyx_INCREF(__pyx_t_1);
      __Pyx_INCREF(function);
      __Pyx_DECREF_SET(__pyx_t_4, function);
      __pyx_t_6 = 1;
    }
  }
  #if CYTHON_FAST_PYCALL
  if (PyFunction_Check(__pyx_t_4)) {
    PyObject *__pyx_temp[3] = {__pyx_t_1, __pyx_v_v_channel, __pyx_t_2};
    __pyx_t_3 = __Pyx_PyFunction_FastCall(__pyx_t_4, __pyx_temp+1-__pyx_t_6, 2+__pyx_t_6); if (unlikely(!__pyx_t_3)) __PYX_ERR(0, 24, __pyx_L1_error)
    __Pyx_XDECREF(__pyx_t_1); __pyx_t_1 = 0;
    __Pyx_GOTREF(__pyx_t_3);
    __Pyx_DECREF(__pyx_t_2); __pyx_t_2 = 0;
  } else
  #endif
  #if CYTHON_FAST_PYCCALL
  if (__Pyx_PyFastCFunction_Check(__pyx_t_4)) {
    PyObject *__pyx_temp[3] = {__pyx_t_1, __pyx_v_v_channel, __pyx_t_2};
    __pyx_t_3 = __Pyx_PyCFunction_FastCall(__pyx_t_4, __pyx_temp+1-__pyx_t_6, 2+__pyx_t_6); if (unlikely(!__pyx_t_3)) __PYX_ERR(0, 24, __pyx_L1_error)
    __Pyx_XDECREF(__pyx_t_1); __pyx_t_1 = 0;
    __Pyx_GOTREF(__pyx_t_3);
    __Pyx_DECREF(__pyx_t_2); __pyx_t_2 = 0;
  } else
  #endif
  {
    __pyx_t_5 = PyTuple_New(2+__pyx_t_6); if (unlikely(!__pyx_t_5)) __PYX_ERR(0, 24, __pyx_L1_error)
    __Pyx_GOTREF(__pyx_t_5);
    if (__pyx_t_1) {
      __Pyx_GIVEREF(__pyx_t_1); PyTuple_SET_ITEM(__pyx_t_5, 0, __pyx_t_1); __pyx_t_1 = NULL;
    }
    __Pyx_INCREF(__pyx_v_v_channel);
    __Pyx_GIVEREF(__pyx_v_v_channel);
    PyTuple_SET_ITEM(__pyx_t_5, 0+__pyx_t_6, __pyx_v_v_channel);
    __Pyx_GIVEREF(__pyx_t_2);
    PyTuple_SET_ITEM(__pyx_t_5, 1+__pyx_t_6, __pyx_t_2);
    __pyx_t_2 = 0;
    __pyx_t_3 = __Pyx_PyObject_Call(__pyx_t_4, __pyx_t_5, NULL); if (unlikely(!__pyx_t_3)) __PYX_ERR(0, 24, __pyx_L1_error)
    __Pyx_GOTREF(__pyx_t_3);
    __Pyx_DECREF(__pyx_t_5); __pyx_t_5 = 0;
  }
  __Pyx_DECREF(__pyx_t_4); __pyx_t_4 = 0;
  __pyx_v_nda_receiver_signal = __pyx_t_3;
  __pyx_t_3 = 0;
```

```
 025:
```

```
+026:     d_rate = np.log2(np.real(np.linalg.det(nda_receiver_signal + nda_receiver_noise)) /
```

```
  __pyx_t_4 = __Pyx_GetModuleGlobalName(__pyx_n_s_np); if (unlikely(!__pyx_t_4)) __PYX_ERR(0, 26, __pyx_L1_error)
  __Pyx_GOTREF(__pyx_t_4);
  __pyx_t_5 = __Pyx_PyObject_GetAttrStr(__pyx_t_4, __pyx_n_s_log2); if (unlikely(!__pyx_t_5)) __PYX_ERR(0, 26, __pyx_L1_error)
  __Pyx_GOTREF(__pyx_t_5);
  __Pyx_DECREF(__pyx_t_4); __pyx_t_4 = 0;
  __pyx_t_2 = __Pyx_GetModuleGlobalName(__pyx_n_s_np); if (unlikely(!__pyx_t_2)) __PYX_ERR(0, 26, __pyx_L1_error)
  __Pyx_GOTREF(__pyx_t_2);
  __pyx_t_1 = __Pyx_PyObject_GetAttrStr(__pyx_t_2, __pyx_n_s_real); if (unlikely(!__pyx_t_1)) __PYX_ERR(0, 26, __pyx_L1_error)
  __Pyx_GOTREF(__pyx_t_1);
  __Pyx_DECREF(__pyx_t_2); __pyx_t_2 = 0;
  __pyx_t_7 = __Pyx_GetModuleGlobalName(__pyx_n_s_np); if (unlikely(!__pyx_t_7)) __PYX_ERR(0, 26, __pyx_L1_error)
  __Pyx_GOTREF(__pyx_t_7);
  __pyx_t_8 = __Pyx_PyObject_GetAttrStr(__pyx_t_7, __pyx_n_s_linalg); if (unlikely(!__pyx_t_8)) __PYX_ERR(0, 26, __pyx_L1_error)
  __Pyx_GOTREF(__pyx_t_8);
  __Pyx_DECREF(__pyx_t_7); __pyx_t_7 = 0;
  __pyx_t_7 = __Pyx_PyObject_GetAttrStr(__pyx_t_8, __pyx_n_s_det); if (unlikely(!__pyx_t_7)) __PYX_ERR(0, 26, __pyx_L1_error)
  __Pyx_GOTREF(__pyx_t_7);
  __Pyx_DECREF(__pyx_t_8); __pyx_t_8 = 0;
  __pyx_t_8 = PyNumber_Add(__pyx_v_nda_receiver_signal, __pyx_v_nda_receiver_noise); if (unlikely(!__pyx_t_8)) __PYX_ERR(0, 26, __pyx_L1_error)
  __Pyx_GOTREF(__pyx_t_8);
  __pyx_t_9 = NULL;
  if (CYTHON_UNPACK_METHODS && likely(PyMethod_Check(__pyx_t_7))) {
    __pyx_t_9 = PyMethod_GET_SELF(__pyx_t_7);
    if (likely(__pyx_t_9)) {
      PyObject* function = PyMethod_GET_FUNCTION(__pyx_t_7);
      __Pyx_INCREF(__pyx_t_9);
      __Pyx_INCREF(function);
      __Pyx_DECREF_SET(__pyx_t_7, function);
    }
  }
  if (!__pyx_t_9) {
    __pyx_t_2 = __Pyx_PyObject_CallOneArg(__pyx_t_7, __pyx_t_8); if (unlikely(!__pyx_t_2)) __PYX_ERR(0, 26, __pyx_L1_error)
    __Pyx_DECREF(__pyx_t_8); __pyx_t_8 = 0;
    __Pyx_GOTREF(__pyx_t_2);
  } else {
    #if CYTHON_FAST_PYCALL
    if (PyFunction_Check(__pyx_t_7)) {
      PyObject *__pyx_temp[2] = {__pyx_t_9, __pyx_t_8};
      __pyx_t_2 = __Pyx_PyFunction_FastCall(__pyx_t_7, __pyx_temp+1-1, 1+1); if (unlikely(!__pyx_t_2)) __PYX_ERR(0, 26, __pyx_L1_error)
      __Pyx_XDECREF(__pyx_t_9); __pyx_t_9 = 0;
      __Pyx_GOTREF(__pyx_t_2);
      __Pyx_DECREF(__pyx_t_8); __pyx_t_8 = 0;
    } else
    #endif
    #if CYTHON_FAST_PYCCALL
    if (__Pyx_PyFastCFunction_Check(__pyx_t_7)) {
      PyObject *__pyx_temp[2] = {__pyx_t_9, __pyx_t_8};
      __pyx_t_2 = __Pyx_PyCFunction_FastCall(__pyx_t_7, __pyx_temp+1-1, 1+1); if (unlikely(!__pyx_t_2)) __PYX_ERR(0, 26, __pyx_L1_error)
      __Pyx_XDECREF(__pyx_t_9); __pyx_t_9 = 0;
      __Pyx_GOTREF(__pyx_t_2);
      __Pyx_DECREF(__pyx_t_8); __pyx_t_8 = 0;
    } else
    #endif
    {
      __pyx_t_10 = PyTuple_New(1+1); if (unlikely(!__pyx_t_10)) __PYX_ERR(0, 26, __pyx_L1_error)
      __Pyx_GOTREF(__pyx_t_10);
      __Pyx_GIVEREF(__pyx_t_9); PyTuple_SET_ITEM(__pyx_t_10, 0, __pyx_t_9); __pyx_t_9 = NULL;
      __Pyx_GIVEREF(__pyx_t_8);
      PyTuple_SET_ITEM(__pyx_t_10, 0+1, __pyx_t_8);
      __pyx_t_8 = 0;
      __pyx_t_2 = __Pyx_PyObject_Call(__pyx_t_7, __pyx_t_10, NULL); if (unlikely(!__pyx_t_2)) __PYX_ERR(0, 26, __pyx_L1_error)
      __Pyx_GOTREF(__pyx_t_2);
      __Pyx_DECREF(__pyx_t_10); __pyx_t_10 = 0;
    }
  }
  __Pyx_DECREF(__pyx_t_7); __pyx_t_7 = 0;
  __pyx_t_7 = NULL;
  if (CYTHON_UNPACK_METHODS && unlikely(PyMethod_Check(__pyx_t_1))) {
    __pyx_t_7 = PyMethod_GET_SELF(__pyx_t_1);
    if (likely(__pyx_t_7)) {
      PyObject* function = PyMethod_GET_FUNCTION(__pyx_t_1);
      __Pyx_INCREF(__pyx_t_7);
      __Pyx_INCREF(function);
      __Pyx_DECREF_SET(__pyx_t_1, function);
    }
  }
  if (!__pyx_t_7) {
    __pyx_t_4 = __Pyx_PyObject_CallOneArg(__pyx_t_1, __pyx_t_2); if (unlikely(!__pyx_t_4)) __PYX_ERR(0, 26, __pyx_L1_error)
    __Pyx_DECREF(__pyx_t_2); __pyx_t_2 = 0;
    __Pyx_GOTREF(__pyx_t_4);
  } else {
    #if CYTHON_FAST_PYCALL
    if (PyFunction_Check(__pyx_t_1)) {
      PyObject *__pyx_temp[2] = {__pyx_t_7, __pyx_t_2};
      __pyx_t_4 = __Pyx_PyFunction_FastCall(__pyx_t_1, __pyx_temp+1-1, 1+1); if (unlikely(!__pyx_t_4)) __PYX_ERR(0, 26, __pyx_L1_error)
      __Pyx_XDECREF(__pyx_t_7); __pyx_t_7 = 0;
      __Pyx_GOTREF(__pyx_t_4);
      __Pyx_DECREF(__pyx_t_2); __pyx_t_2 = 0;
    } else
    #endif
    #if CYTHON_FAST_PYCCALL
    if (__Pyx_PyFastCFunction_Check(__pyx_t_1)) {
      PyObject *__pyx_temp[2] = {__pyx_t_7, __pyx_t_2};
      __pyx_t_4 = __Pyx_PyCFunction_FastCall(__pyx_t_1, __pyx_temp+1-1, 1+1); if (unlikely(!__pyx_t_4)) __PYX_ERR(0, 26, __pyx_L1_error)
      __Pyx_XDECREF(__pyx_t_7); __pyx_t_7 = 0;
      __Pyx_GOTREF(__pyx_t_4);
      __Pyx_DECREF(__pyx_t_2); __pyx_t_2 = 0;
    } else
    #endif
    {
      __pyx_t_10 = PyTuple_New(1+1); if (unlikely(!__pyx_t_10)) __PYX_ERR(0, 26, __pyx_L1_error)
      __Pyx_GOTREF(__pyx_t_10);
      __Pyx_GIVEREF(__pyx_t_7); PyTuple_SET_ITEM(__pyx_t_10, 0, __pyx_t_7); __pyx_t_7 = NULL;
      __Pyx_GIVEREF(__pyx_t_2);
      PyTuple_SET_ITEM(__pyx_t_10, 0+1, __pyx_t_2);
      __pyx_t_2 = 0;
      __pyx_t_4 = __Pyx_PyObject_Call(__pyx_t_1, __pyx_t_10, NULL); if (unlikely(!__pyx_t_4)) __PYX_ERR(0, 26, __pyx_L1_error)
      __Pyx_GOTREF(__pyx_t_4);
      __Pyx_DECREF(__pyx_t_10); __pyx_t_10 = 0;
    }
  }
  __Pyx_DECREF(__pyx_t_1); __pyx_t_1 = 0;
/* … */
  __pyx_t_2 = __Pyx_PyNumber_Divide(__pyx_t_4, __pyx_t_1); if (unlikely(!__pyx_t_2)) __PYX_ERR(0, 26, __pyx_L1_error)
  __Pyx_GOTREF(__pyx_t_2);
  __Pyx_DECREF(__pyx_t_4); __pyx_t_4 = 0;
  __Pyx_DECREF(__pyx_t_1); __pyx_t_1 = 0;
  __pyx_t_1 = NULL;
  if (CYTHON_UNPACK_METHODS && unlikely(PyMethod_Check(__pyx_t_5))) {
    __pyx_t_1 = PyMethod_GET_SELF(__pyx_t_5);
    if (likely(__pyx_t_1)) {
      PyObject* function = PyMethod_GET_FUNCTION(__pyx_t_5);
      __Pyx_INCREF(__pyx_t_1);
      __Pyx_INCREF(function);
      __Pyx_DECREF_SET(__pyx_t_5, function);
    }
  }
  if (!__pyx_t_1) {
    __pyx_t_3 = __Pyx_PyObject_CallOneArg(__pyx_t_5, __pyx_t_2); if (unlikely(!__pyx_t_3)) __PYX_ERR(0, 26, __pyx_L1_error)
    __Pyx_DECREF(__pyx_t_2); __pyx_t_2 = 0;
    __Pyx_GOTREF(__pyx_t_3);
  } else {
    #if CYTHON_FAST_PYCALL
    if (PyFunction_Check(__pyx_t_5)) {
      PyObject *__pyx_temp[2] = {__pyx_t_1, __pyx_t_2};
      __pyx_t_3 = __Pyx_PyFunction_FastCall(__pyx_t_5, __pyx_temp+1-1, 1+1); if (unlikely(!__pyx_t_3)) __PYX_ERR(0, 26, __pyx_L1_error)
      __Pyx_XDECREF(__pyx_t_1); __pyx_t_1 = 0;
      __Pyx_GOTREF(__pyx_t_3);
      __Pyx_DECREF(__pyx_t_2); __pyx_t_2 = 0;
    } else
    #endif
    #if CYTHON_FAST_PYCCALL
    if (__Pyx_PyFastCFunction_Check(__pyx_t_5)) {
      PyObject *__pyx_temp[2] = {__pyx_t_1, __pyx_t_2};
      __pyx_t_3 = __Pyx_PyCFunction_FastCall(__pyx_t_5, __pyx_temp+1-1, 1+1); if (unlikely(!__pyx_t_3)) __PYX_ERR(0, 26, __pyx_L1_error)
      __Pyx_XDECREF(__pyx_t_1); __pyx_t_1 = 0;
      __Pyx_GOTREF(__pyx_t_3);
      __Pyx_DECREF(__pyx_t_2); __pyx_t_2 = 0;
    } else
    #endif
    {
      __pyx_t_4 = PyTuple_New(1+1); if (unlikely(!__pyx_t_4)) __PYX_ERR(0, 26, __pyx_L1_error)
      __Pyx_GOTREF(__pyx_t_4);
      __Pyx_GIVEREF(__pyx_t_1); PyTuple_SET_ITEM(__pyx_t_4, 0, __pyx_t_1); __pyx_t_1 = NULL;
      __Pyx_GIVEREF(__pyx_t_2);
      PyTuple_SET_ITEM(__pyx_t_4, 0+1, __pyx_t_2);
      __pyx_t_2 = 0;
      __pyx_t_3 = __Pyx_PyObject_Call(__pyx_t_5, __pyx_t_4, NULL); if (unlikely(!__pyx_t_3)) __PYX_ERR(0, 26, __pyx_L1_error)
      __Pyx_GOTREF(__pyx_t_3);
      __Pyx_DECREF(__pyx_t_4); __pyx_t_4 = 0;
    }
  }
  __Pyx_DECREF(__pyx_t_5); __pyx_t_5 = 0;
  __pyx_v_d_rate = __pyx_t_3;
  __pyx_t_3 = 0;
```

```
+027:                      np.real(np.linalg.det(nda_receiver_noise)))
```

```
  __pyx_t_10 = __Pyx_GetModuleGlobalName(__pyx_n_s_np); if (unlikely(!__pyx_t_10)) __PYX_ERR(0, 27, __pyx_L1_error)
  __Pyx_GOTREF(__pyx_t_10);
  __pyx_t_2 = __Pyx_PyObject_GetAttrStr(__pyx_t_10, __pyx_n_s_real); if (unlikely(!__pyx_t_2)) __PYX_ERR(0, 27, __pyx_L1_error)
  __Pyx_GOTREF(__pyx_t_2);
  __Pyx_DECREF(__pyx_t_10); __pyx_t_10 = 0;
  __pyx_t_7 = __Pyx_GetModuleGlobalName(__pyx_n_s_np); if (unlikely(!__pyx_t_7)) __PYX_ERR(0, 27, __pyx_L1_error)
  __Pyx_GOTREF(__pyx_t_7);
  __pyx_t_8 = __Pyx_PyObject_GetAttrStr(__pyx_t_7, __pyx_n_s_linalg); if (unlikely(!__pyx_t_8)) __PYX_ERR(0, 27, __pyx_L1_error)
  __Pyx_GOTREF(__pyx_t_8);
  __Pyx_DECREF(__pyx_t_7); __pyx_t_7 = 0;
  __pyx_t_7 = __Pyx_PyObject_GetAttrStr(__pyx_t_8, __pyx_n_s_det); if (unlikely(!__pyx_t_7)) __PYX_ERR(0, 27, __pyx_L1_error)
  __Pyx_GOTREF(__pyx_t_7);
  __Pyx_DECREF(__pyx_t_8); __pyx_t_8 = 0;
  __pyx_t_8 = NULL;
  if (CYTHON_UNPACK_METHODS && likely(PyMethod_Check(__pyx_t_7))) {
    __pyx_t_8 = PyMethod_GET_SELF(__pyx_t_7);
    if (likely(__pyx_t_8)) {
      PyObject* function = PyMethod_GET_FUNCTION(__pyx_t_7);
      __Pyx_INCREF(__pyx_t_8);
      __Pyx_INCREF(function);
      __Pyx_DECREF_SET(__pyx_t_7, function);
    }
  }
  if (!__pyx_t_8) {
    __pyx_t_10 = __Pyx_PyObject_CallOneArg(__pyx_t_7, __pyx_v_nda_receiver_noise); if (unlikely(!__pyx_t_10)) __PYX_ERR(0, 27, __pyx_L1_error)
    __Pyx_GOTREF(__pyx_t_10);
  } else {
    #if CYTHON_FAST_PYCALL
    if (PyFunction_Check(__pyx_t_7)) {
      PyObject *__pyx_temp[2] = {__pyx_t_8, __pyx_v_nda_receiver_noise};
      __pyx_t_10 = __Pyx_PyFunction_FastCall(__pyx_t_7, __pyx_temp+1-1, 1+1); if (unlikely(!__pyx_t_10)) __PYX_ERR(0, 27, __pyx_L1_error)
      __Pyx_XDECREF(__pyx_t_8); __pyx_t_8 = 0;
      __Pyx_GOTREF(__pyx_t_10);
    } else
    #endif
    #if CYTHON_FAST_PYCCALL
    if (__Pyx_PyFastCFunction_Check(__pyx_t_7)) {
      PyObject *__pyx_temp[2] = {__pyx_t_8, __pyx_v_nda_receiver_noise};
      __pyx_t_10 = __Pyx_PyCFunction_FastCall(__pyx_t_7, __pyx_temp+1-1, 1+1); if (unlikely(!__pyx_t_10)) __PYX_ERR(0, 27, __pyx_L1_error)
      __Pyx_XDECREF(__pyx_t_8); __pyx_t_8 = 0;
      __Pyx_GOTREF(__pyx_t_10);
    } else
    #endif
    {
      __pyx_t_9 = PyTuple_New(1+1); if (unlikely(!__pyx_t_9)) __PYX_ERR(0, 27, __pyx_L1_error)
      __Pyx_GOTREF(__pyx_t_9);
      __Pyx_GIVEREF(__pyx_t_8); PyTuple_SET_ITEM(__pyx_t_9, 0, __pyx_t_8); __pyx_t_8 = NULL;
      __Pyx_INCREF(__pyx_v_nda_receiver_noise);
      __Pyx_GIVEREF(__pyx_v_nda_receiver_noise);
      PyTuple_SET_ITEM(__pyx_t_9, 0+1, __pyx_v_nda_receiver_noise);
      __pyx_t_10 = __Pyx_PyObject_Call(__pyx_t_7, __pyx_t_9, NULL); if (unlikely(!__pyx_t_10)) __PYX_ERR(0, 27, __pyx_L1_error)
      __Pyx_GOTREF(__pyx_t_10);
      __Pyx_DECREF(__pyx_t_9); __pyx_t_9 = 0;
    }
  }
  __Pyx_DECREF(__pyx_t_7); __pyx_t_7 = 0;
  __pyx_t_7 = NULL;
  if (CYTHON_UNPACK_METHODS && unlikely(PyMethod_Check(__pyx_t_2))) {
    __pyx_t_7 = PyMethod_GET_SELF(__pyx_t_2);
    if (likely(__pyx_t_7)) {
      PyObject* function = PyMethod_GET_FUNCTION(__pyx_t_2);
      __Pyx_INCREF(__pyx_t_7);
      __Pyx_INCREF(function);
      __Pyx_DECREF_SET(__pyx_t_2, function);
    }
  }
  if (!__pyx_t_7) {
    __pyx_t_1 = __Pyx_PyObject_CallOneArg(__pyx_t_2, __pyx_t_10); if (unlikely(!__pyx_t_1)) __PYX_ERR(0, 27, __pyx_L1_error)
    __Pyx_DECREF(__pyx_t_10); __pyx_t_10 = 0;
    __Pyx_GOTREF(__pyx_t_1);
  } else {
    #if CYTHON_FAST_PYCALL
    if (PyFunction_Check(__pyx_t_2)) {
      PyObject *__pyx_temp[2] = {__pyx_t_7, __pyx_t_10};
      __pyx_t_1 = __Pyx_PyFunction_FastCall(__pyx_t_2, __pyx_temp+1-1, 1+1); if (unlikely(!__pyx_t_1)) __PYX_ERR(0, 27, __pyx_L1_error)
      __Pyx_XDECREF(__pyx_t_7); __pyx_t_7 = 0;
      __Pyx_GOTREF(__pyx_t_1);
      __Pyx_DECREF(__pyx_t_10); __pyx_t_10 = 0;
    } else
    #endif
    #if CYTHON_FAST_PYCCALL
    if (__Pyx_PyFastCFunction_Check(__pyx_t_2)) {
      PyObject *__pyx_temp[2] = {__pyx_t_7, __pyx_t_10};
      __pyx_t_1 = __Pyx_PyCFunction_FastCall(__pyx_t_2, __pyx_temp+1-1, 1+1); if (unlikely(!__pyx_t_1)) __PYX_ERR(0, 27, __pyx_L1_error)
      __Pyx_XDECREF(__pyx_t_7); __pyx_t_7 = 0;
      __Pyx_GOTREF(__pyx_t_1);
      __Pyx_DECREF(__pyx_t_10); __pyx_t_10 = 0;
    } else
    #endif
    {
      __pyx_t_9 = PyTuple_New(1+1); if (unlikely(!__pyx_t_9)) __PYX_ERR(0, 27, __pyx_L1_error)
      __Pyx_GOTREF(__pyx_t_9);
      __Pyx_GIVEREF(__pyx_t_7); PyTuple_SET_ITEM(__pyx_t_9, 0, __pyx_t_7); __pyx_t_7 = NULL;
      __Pyx_GIVEREF(__pyx_t_10);
      PyTuple_SET_ITEM(__pyx_t_9, 0+1, __pyx_t_10);
      __pyx_t_10 = 0;
      __pyx_t_1 = __Pyx_PyObject_Call(__pyx_t_2, __pyx_t_9, NULL); if (unlikely(!__pyx_t_1)) __PYX_ERR(0, 27, __pyx_L1_error)
      __Pyx_GOTREF(__pyx_t_1);
      __Pyx_DECREF(__pyx_t_9); __pyx_t_9 = 0;
    }
  }
  __Pyx_DECREF(__pyx_t_2); __pyx_t_2 = 0;
```

```
 028:
```

```
+029:     d_rate_0 = np.log2(np.real(1+nda_interf[0, 0] + np.abs(v_channel[0])**2) /
```

```
  __pyx_t_5 = __Pyx_GetModuleGlobalName(__pyx_n_s_np); if (unlikely(!__pyx_t_5)) __PYX_ERR(0, 29, __pyx_L1_error)
  __Pyx_GOTREF(__pyx_t_5);
  __pyx_t_4 = __Pyx_PyObject_GetAttrStr(__pyx_t_5, __pyx_n_s_log2); if (unlikely(!__pyx_t_4)) __PYX_ERR(0, 29, __pyx_L1_error)
  __Pyx_GOTREF(__pyx_t_4);
  __Pyx_DECREF(__pyx_t_5); __pyx_t_5 = 0;
  __pyx_t_2 = __Pyx_GetModuleGlobalName(__pyx_n_s_np); if (unlikely(!__pyx_t_2)) __PYX_ERR(0, 29, __pyx_L1_error)
  __Pyx_GOTREF(__pyx_t_2);
  __pyx_t_1 = __Pyx_PyObject_GetAttrStr(__pyx_t_2, __pyx_n_s_real); if (unlikely(!__pyx_t_1)) __PYX_ERR(0, 29, __pyx_L1_error)
  __Pyx_GOTREF(__pyx_t_1);
  __Pyx_DECREF(__pyx_t_2); __pyx_t_2 = 0;
  __pyx_t_2 = PyObject_GetItem(__pyx_v_nda_interf, __pyx_tuple__2); if (unlikely(!__pyx_t_2)) __PYX_ERR(0, 29, __pyx_L1_error)
  __Pyx_GOTREF(__pyx_t_2);
  __pyx_t_9 = __Pyx_PyInt_AddCObj(__pyx_int_1, __pyx_t_2, 1, 0); if (unlikely(!__pyx_t_9)) __PYX_ERR(0, 29, __pyx_L1_error)
  __Pyx_GOTREF(__pyx_t_9);
  __Pyx_DECREF(__pyx_t_2); __pyx_t_2 = 0;
  __pyx_t_10 = __Pyx_GetModuleGlobalName(__pyx_n_s_np); if (unlikely(!__pyx_t_10)) __PYX_ERR(0, 29, __pyx_L1_error)
  __Pyx_GOTREF(__pyx_t_10);
  __pyx_t_7 = __Pyx_PyObject_GetAttrStr(__pyx_t_10, __pyx_n_s_abs); if (unlikely(!__pyx_t_7)) __PYX_ERR(0, 29, __pyx_L1_error)
  __Pyx_GOTREF(__pyx_t_7);
  __Pyx_DECREF(__pyx_t_10); __pyx_t_10 = 0;
  __pyx_t_10 = __Pyx_GetItemInt(__pyx_v_v_channel, 0, long, 1, __Pyx_PyInt_From_long, 0, 0, 1); if (unlikely(!__pyx_t_10)) __PYX_ERR(0, 29, __pyx_L1_error)
  __Pyx_GOTREF(__pyx_t_10);
  __pyx_t_8 = NULL;
  if (CYTHON_UNPACK_METHODS && unlikely(PyMethod_Check(__pyx_t_7))) {
    __pyx_t_8 = PyMethod_GET_SELF(__pyx_t_7);
    if (likely(__pyx_t_8)) {
      PyObject* function = PyMethod_GET_FUNCTION(__pyx_t_7);
      __Pyx_INCREF(__pyx_t_8);
      __Pyx_INCREF(function);
      __Pyx_DECREF_SET(__pyx_t_7, function);
    }
  }
  if (!__pyx_t_8) {
    __pyx_t_2 = __Pyx_PyObject_CallOneArg(__pyx_t_7, __pyx_t_10); if (unlikely(!__pyx_t_2)) __PYX_ERR(0, 29, __pyx_L1_error)
    __Pyx_DECREF(__pyx_t_10); __pyx_t_10 = 0;
    __Pyx_GOTREF(__pyx_t_2);
  } else {
    #if CYTHON_FAST_PYCALL
    if (PyFunction_Check(__pyx_t_7)) {
      PyObject *__pyx_temp[2] = {__pyx_t_8, __pyx_t_10};
      __pyx_t_2 = __Pyx_PyFunction_FastCall(__pyx_t_7, __pyx_temp+1-1, 1+1); if (unlikely(!__pyx_t_2)) __PYX_ERR(0, 29, __pyx_L1_error)
      __Pyx_XDECREF(__pyx_t_8); __pyx_t_8 = 0;
      __Pyx_GOTREF(__pyx_t_2);
      __Pyx_DECREF(__pyx_t_10); __pyx_t_10 = 0;
    } else
    #endif
    #if CYTHON_FAST_PYCCALL
    if (__Pyx_PyFastCFunction_Check(__pyx_t_7)) {
      PyObject *__pyx_temp[2] = {__pyx_t_8, __pyx_t_10};
      __pyx_t_2 = __Pyx_PyCFunction_FastCall(__pyx_t_7, __pyx_temp+1-1, 1+1); if (unlikely(!__pyx_t_2)) __PYX_ERR(0, 29, __pyx_L1_error)
      __Pyx_XDECREF(__pyx_t_8); __pyx_t_8 = 0;
      __Pyx_GOTREF(__pyx_t_2);
      __Pyx_DECREF(__pyx_t_10); __pyx_t_10 = 0;
    } else
    #endif
    {
      __pyx_t_11 = PyTuple_New(1+1); if (unlikely(!__pyx_t_11)) __PYX_ERR(0, 29, __pyx_L1_error)
      __Pyx_GOTREF(__pyx_t_11);
      __Pyx_GIVEREF(__pyx_t_8); PyTuple_SET_ITEM(__pyx_t_11, 0, __pyx_t_8); __pyx_t_8 = NULL;
      __Pyx_GIVEREF(__pyx_t_10);
      PyTuple_SET_ITEM(__pyx_t_11, 0+1, __pyx_t_10);
      __pyx_t_10 = 0;
      __pyx_t_2 = __Pyx_PyObject_Call(__pyx_t_7, __pyx_t_11, NULL); if (unlikely(!__pyx_t_2)) __PYX_ERR(0, 29, __pyx_L1_error)
      __Pyx_GOTREF(__pyx_t_2);
      __Pyx_DECREF(__pyx_t_11); __pyx_t_11 = 0;
    }
  }
  __Pyx_DECREF(__pyx_t_7); __pyx_t_7 = 0;
  __pyx_t_7 = PyNumber_Power(__pyx_t_2, __pyx_int_2, Py_None); if (unlikely(!__pyx_t_7)) __PYX_ERR(0, 29, __pyx_L1_error)
  __Pyx_GOTREF(__pyx_t_7);
  __Pyx_DECREF(__pyx_t_2); __pyx_t_2 = 0;
  __pyx_t_2 = PyNumber_Add(__pyx_t_9, __pyx_t_7); if (unlikely(!__pyx_t_2)) __PYX_ERR(0, 29, __pyx_L1_error)
  __Pyx_GOTREF(__pyx_t_2);
  __Pyx_DECREF(__pyx_t_9); __pyx_t_9 = 0;
  __Pyx_DECREF(__pyx_t_7); __pyx_t_7 = 0;
  __pyx_t_7 = NULL;
  if (CYTHON_UNPACK_METHODS && unlikely(PyMethod_Check(__pyx_t_1))) {
    __pyx_t_7 = PyMethod_GET_SELF(__pyx_t_1);
    if (likely(__pyx_t_7)) {
      PyObject* function = PyMethod_GET_FUNCTION(__pyx_t_1);
      __Pyx_INCREF(__pyx_t_7);
      __Pyx_INCREF(function);
      __Pyx_DECREF_SET(__pyx_t_1, function);
    }
  }
  if (!__pyx_t_7) {
    __pyx_t_5 = __Pyx_PyObject_CallOneArg(__pyx_t_1, __pyx_t_2); if (unlikely(!__pyx_t_5)) __PYX_ERR(0, 29, __pyx_L1_error)
    __Pyx_DECREF(__pyx_t_2); __pyx_t_2 = 0;
    __Pyx_GOTREF(__pyx_t_5);
  } else {
    #if CYTHON_FAST_PYCALL
    if (PyFunction_Check(__pyx_t_1)) {
      PyObject *__pyx_temp[2] = {__pyx_t_7, __pyx_t_2};
      __pyx_t_5 = __Pyx_PyFunction_FastCall(__pyx_t_1, __pyx_temp+1-1, 1+1); if (unlikely(!__pyx_t_5)) __PYX_ERR(0, 29, __pyx_L1_error)
      __Pyx_XDECREF(__pyx_t_7); __pyx_t_7 = 0;
      __Pyx_GOTREF(__pyx_t_5);
      __Pyx_DECREF(__pyx_t_2); __pyx_t_2 = 0;
    } else
    #endif
    #if CYTHON_FAST_PYCCALL
    if (__Pyx_PyFastCFunction_Check(__pyx_t_1)) {
      PyObject *__pyx_temp[2] = {__pyx_t_7, __pyx_t_2};
      __pyx_t_5 = __Pyx_PyCFunction_FastCall(__pyx_t_1, __pyx_temp+1-1, 1+1); if (unlikely(!__pyx_t_5)) __PYX_ERR(0, 29, __pyx_L1_error)
      __Pyx_XDECREF(__pyx_t_7); __pyx_t_7 = 0;
      __Pyx_GOTREF(__pyx_t_5);
      __Pyx_DECREF(__pyx_t_2); __pyx_t_2 = 0;
    } else
    #endif
    {
      __pyx_t_9 = PyTuple_New(1+1); if (unlikely(!__pyx_t_9)) __PYX_ERR(0, 29, __pyx_L1_error)
      __Pyx_GOTREF(__pyx_t_9);
      __Pyx_GIVEREF(__pyx_t_7); PyTuple_SET_ITEM(__pyx_t_9, 0, __pyx_t_7); __pyx_t_7 = NULL;
      __Pyx_GIVEREF(__pyx_t_2);
      PyTuple_SET_ITEM(__pyx_t_9, 0+1, __pyx_t_2);
      __pyx_t_2 = 0;
      __pyx_t_5 = __Pyx_PyObject_Call(__pyx_t_1, __pyx_t_9, NULL); if (unlikely(!__pyx_t_5)) __PYX_ERR(0, 29, __pyx_L1_error)
      __Pyx_GOTREF(__pyx_t_5);
      __Pyx_DECREF(__pyx_t_9); __pyx_t_9 = 0;
    }
  }
  __Pyx_DECREF(__pyx_t_1); __pyx_t_1 = 0;
/* … */
  __pyx_tuple__2 = PyTuple_Pack(2, __pyx_int_0, __pyx_int_0); if (unlikely(!__pyx_tuple__2)) __PYX_ERR(0, 29, __pyx_L1_error)
  __Pyx_GOTREF(__pyx_tuple__2);
  __Pyx_GIVEREF(__pyx_tuple__2);
/* … */
  __pyx_t_2 = __Pyx_PyNumber_Divide(__pyx_t_5, __pyx_t_1); if (unlikely(!__pyx_t_2)) __PYX_ERR(0, 29, __pyx_L1_error)
  __Pyx_GOTREF(__pyx_t_2);
  __Pyx_DECREF(__pyx_t_5); __pyx_t_5 = 0;
  __Pyx_DECREF(__pyx_t_1); __pyx_t_1 = 0;
  __pyx_t_1 = NULL;
  if (CYTHON_UNPACK_METHODS && unlikely(PyMethod_Check(__pyx_t_4))) {
    __pyx_t_1 = PyMethod_GET_SELF(__pyx_t_4);
    if (likely(__pyx_t_1)) {
      PyObject* function = PyMethod_GET_FUNCTION(__pyx_t_4);
      __Pyx_INCREF(__pyx_t_1);
      __Pyx_INCREF(function);
      __Pyx_DECREF_SET(__pyx_t_4, function);
    }
  }
  if (!__pyx_t_1) {
    __pyx_t_3 = __Pyx_PyObject_CallOneArg(__pyx_t_4, __pyx_t_2); if (unlikely(!__pyx_t_3)) __PYX_ERR(0, 29, __pyx_L1_error)
    __Pyx_DECREF(__pyx_t_2); __pyx_t_2 = 0;
    __Pyx_GOTREF(__pyx_t_3);
  } else {
    #if CYTHON_FAST_PYCALL
    if (PyFunction_Check(__pyx_t_4)) {
      PyObject *__pyx_temp[2] = {__pyx_t_1, __pyx_t_2};
      __pyx_t_3 = __Pyx_PyFunction_FastCall(__pyx_t_4, __pyx_temp+1-1, 1+1); if (unlikely(!__pyx_t_3)) __PYX_ERR(0, 29, __pyx_L1_error)
      __Pyx_XDECREF(__pyx_t_1); __pyx_t_1 = 0;
      __Pyx_GOTREF(__pyx_t_3);
      __Pyx_DECREF(__pyx_t_2); __pyx_t_2 = 0;
    } else
    #endif
    #if CYTHON_FAST_PYCCALL
    if (__Pyx_PyFastCFunction_Check(__pyx_t_4)) {
      PyObject *__pyx_temp[2] = {__pyx_t_1, __pyx_t_2};
      __pyx_t_3 = __Pyx_PyCFunction_FastCall(__pyx_t_4, __pyx_temp+1-1, 1+1); if (unlikely(!__pyx_t_3)) __PYX_ERR(0, 29, __pyx_L1_error)
      __Pyx_XDECREF(__pyx_t_1); __pyx_t_1 = 0;
      __Pyx_GOTREF(__pyx_t_3);
      __Pyx_DECREF(__pyx_t_2); __pyx_t_2 = 0;
    } else
    #endif
    {
      __pyx_t_5 = PyTuple_New(1+1); if (unlikely(!__pyx_t_5)) __PYX_ERR(0, 29, __pyx_L1_error)
      __Pyx_GOTREF(__pyx_t_5);
      __Pyx_GIVEREF(__pyx_t_1); PyTuple_SET_ITEM(__pyx_t_5, 0, __pyx_t_1); __pyx_t_1 = NULL;
      __Pyx_GIVEREF(__pyx_t_2);
      PyTuple_SET_ITEM(__pyx_t_5, 0+1, __pyx_t_2);
      __pyx_t_2 = 0;
      __pyx_t_3 = __Pyx_PyObject_Call(__pyx_t_4, __pyx_t_5, NULL); if (unlikely(!__pyx_t_3)) __PYX_ERR(0, 29, __pyx_L1_error)
      __Pyx_GOTREF(__pyx_t_3);
      __Pyx_DECREF(__pyx_t_5); __pyx_t_5 = 0;
    }
  }
  __Pyx_DECREF(__pyx_t_4); __pyx_t_4 = 0;
  __pyx_v_d_rate_0 = __pyx_t_3;
  __pyx_t_3 = 0;
```

```
+030:                        np.real(1+nda_interf[0, 0]))
```

```
  __pyx_t_9 = __Pyx_GetModuleGlobalName(__pyx_n_s_np); if (unlikely(!__pyx_t_9)) __PYX_ERR(0, 30, __pyx_L1_error)
  __Pyx_GOTREF(__pyx_t_9);
  __pyx_t_2 = __Pyx_PyObject_GetAttrStr(__pyx_t_9, __pyx_n_s_real); if (unlikely(!__pyx_t_2)) __PYX_ERR(0, 30, __pyx_L1_error)
  __Pyx_GOTREF(__pyx_t_2);
  __Pyx_DECREF(__pyx_t_9); __pyx_t_9 = 0;
  __pyx_t_9 = PyObject_GetItem(__pyx_v_nda_interf, __pyx_tuple__3); if (unlikely(!__pyx_t_9)) __PYX_ERR(0, 30, __pyx_L1_error)
  __Pyx_GOTREF(__pyx_t_9);
  __pyx_t_7 = __Pyx_PyInt_AddCObj(__pyx_int_1, __pyx_t_9, 1, 0); if (unlikely(!__pyx_t_7)) __PYX_ERR(0, 30, __pyx_L1_error)
  __Pyx_GOTREF(__pyx_t_7);
  __Pyx_DECREF(__pyx_t_9); __pyx_t_9 = 0;
  __pyx_t_9 = NULL;
  if (CYTHON_UNPACK_METHODS && unlikely(PyMethod_Check(__pyx_t_2))) {
    __pyx_t_9 = PyMethod_GET_SELF(__pyx_t_2);
    if (likely(__pyx_t_9)) {
      PyObject* function = PyMethod_GET_FUNCTION(__pyx_t_2);
      __Pyx_INCREF(__pyx_t_9);
      __Pyx_INCREF(function);
      __Pyx_DECREF_SET(__pyx_t_2, function);
    }
  }
  if (!__pyx_t_9) {
    __pyx_t_1 = __Pyx_PyObject_CallOneArg(__pyx_t_2, __pyx_t_7); if (unlikely(!__pyx_t_1)) __PYX_ERR(0, 30, __pyx_L1_error)
    __Pyx_DECREF(__pyx_t_7); __pyx_t_7 = 0;
    __Pyx_GOTREF(__pyx_t_1);
  } else {
    #if CYTHON_FAST_PYCALL
    if (PyFunction_Check(__pyx_t_2)) {
      PyObject *__pyx_temp[2] = {__pyx_t_9, __pyx_t_7};
      __pyx_t_1 = __Pyx_PyFunction_FastCall(__pyx_t_2, __pyx_temp+1-1, 1+1); if (unlikely(!__pyx_t_1)) __PYX_ERR(0, 30, __pyx_L1_error)
      __Pyx_XDECREF(__pyx_t_9); __pyx_t_9 = 0;
      __Pyx_GOTREF(__pyx_t_1);
      __Pyx_DECREF(__pyx_t_7); __pyx_t_7 = 0;
    } else
    #endif
    #if CYTHON_FAST_PYCCALL
    if (__Pyx_PyFastCFunction_Check(__pyx_t_2)) {
      PyObject *__pyx_temp[2] = {__pyx_t_9, __pyx_t_7};
      __pyx_t_1 = __Pyx_PyCFunction_FastCall(__pyx_t_2, __pyx_temp+1-1, 1+1); if (unlikely(!__pyx_t_1)) __PYX_ERR(0, 30, __pyx_L1_error)
      __Pyx_XDECREF(__pyx_t_9); __pyx_t_9 = 0;
      __Pyx_GOTREF(__pyx_t_1);
      __Pyx_DECREF(__pyx_t_7); __pyx_t_7 = 0;
    } else
    #endif
    {
      __pyx_t_11 = PyTuple_New(1+1); if (unlikely(!__pyx_t_11)) __PYX_ERR(0, 30, __pyx_L1_error)
      __Pyx_GOTREF(__pyx_t_11);
      __Pyx_GIVEREF(__pyx_t_9); PyTuple_SET_ITEM(__pyx_t_11, 0, __pyx_t_9); __pyx_t_9 = NULL;
      __Pyx_GIVEREF(__pyx_t_7);
      PyTuple_SET_ITEM(__pyx_t_11, 0+1, __pyx_t_7);
      __pyx_t_7 = 0;
      __pyx_t_1 = __Pyx_PyObject_Call(__pyx_t_2, __pyx_t_11, NULL); if (unlikely(!__pyx_t_1)) __PYX_ERR(0, 30, __pyx_L1_error)
      __Pyx_GOTREF(__pyx_t_1);
      __Pyx_DECREF(__pyx_t_11); __pyx_t_11 = 0;
    }
  }
  __Pyx_DECREF(__pyx_t_2); __pyx_t_2 = 0;
/* … */
  __pyx_tuple__3 = PyTuple_Pack(2, __pyx_int_0, __pyx_int_0); if (unlikely(!__pyx_tuple__3)) __PYX_ERR(0, 30, __pyx_L1_error)
  __Pyx_GOTREF(__pyx_tuple__3);
  __Pyx_GIVEREF(__pyx_tuple__3);
```

```
+031:     if not (d_rate_0 > 0):
```

```
  __pyx_t_3 = PyObject_RichCompare(__pyx_v_d_rate_0, __pyx_int_0, Py_GT); __Pyx_XGOTREF(__pyx_t_3); if (unlikely(!__pyx_t_3)) __PYX_ERR(0, 31, __pyx_L1_error)
  __pyx_t_12 = __Pyx_PyObject_IsTrue(__pyx_t_3); if (unlikely(__pyx_t_12 < 0)) __PYX_ERR(0, 31, __pyx_L1_error)
  __Pyx_DECREF(__pyx_t_3); __pyx_t_3 = 0;
  __pyx_t_13 = ((!__pyx_t_12) != 0);
  if (__pyx_t_13) {
/* … */
  }
```

```
+032:         d_rate_0 = 0
```

```
    __Pyx_INCREF(__pyx_int_0);
    __Pyx_DECREF_SET(__pyx_v_d_rate_0, __pyx_int_0);
```

```
 033:
```

```
+034:     d_rate = min(d_rate, d_bits + d_rate_0)
```

```
  __pyx_t_3 = PyNumber_Add(__pyx_v_d_bits, __pyx_v_d_rate_0); if (unlikely(!__pyx_t_3)) __PYX_ERR(0, 34, __pyx_L1_error)
  __Pyx_GOTREF(__pyx_t_3);
  __Pyx_INCREF(__pyx_v_d_rate);
  __pyx_t_4 = __pyx_v_d_rate;
  __pyx_t_2 = PyObject_RichCompare(__pyx_t_3, __pyx_t_4, Py_LT); __Pyx_XGOTREF(__pyx_t_2); if (unlikely(!__pyx_t_2)) __PYX_ERR(0, 34, __pyx_L1_error)
  __pyx_t_13 = __Pyx_PyObject_IsTrue(__pyx_t_2); if (unlikely(__pyx_t_13 < 0)) __PYX_ERR(0, 34, __pyx_L1_error)
  __Pyx_DECREF(__pyx_t_2); __pyx_t_2 = 0;
  if (__pyx_t_13) {
    __Pyx_INCREF(__pyx_t_3);
    __pyx_t_5 = __pyx_t_3;
  } else {
    __Pyx_INCREF(__pyx_t_4);
    __pyx_t_5 = __pyx_t_4;
  }
  __Pyx_DECREF(__pyx_t_4); __pyx_t_4 = 0;
  __Pyx_DECREF(__pyx_t_3); __pyx_t_3 = 0;
  __pyx_t_3 = __pyx_t_5;
  __Pyx_INCREF(__pyx_t_3);
  __Pyx_DECREF(__pyx_t_5); __pyx_t_5 = 0;
  __Pyx_DECREF_SET(__pyx_v_d_rate, __pyx_t_3);
  __pyx_t_3 = 0;
```

```
+035:     return d_rate
```

```
  __Pyx_XDECREF(__pyx_r);
  __Pyx_INCREF(__pyx_v_d_rate);
  __pyx_r = __pyx_v_d_rate;
  goto __pyx_L0;
```

```
 036:
```

```
 037:
```

```
+038: def rate_bc(v_channel, nda_interf, d_bits):
```

```
/* Python wrapper */
static PyObject *__pyx_pw_9dist_recv_5rates_3rate_bc(PyObject *__pyx_self, PyObject *__pyx_args, PyObject *__pyx_kwds); /*proto*/
static char __pyx_doc_9dist_recv_5rates_2rate_bc[] = " Rate for Broadcast distributed receive with reception at base\n        Input:\n            v_channel = Channel vector h (size N+1 x 1)\n            nda_interf = Interference covariance matrix Sigma (size N+1 x N+1)\n            d_bits = Total number of bits used by helpers\n        Output:\n            d_rate = Rate (in bits)\n    ";
static PyMethodDef __pyx_mdef_9dist_recv_5rates_3rate_bc = {"rate_bc", (PyCFunction)__pyx_pw_9dist_recv_5rates_3rate_bc, METH_VARARGS|METH_KEYWORDS, __pyx_doc_9dist_recv_5rates_2rate_bc};
static PyObject *__pyx_pw_9dist_recv_5rates_3rate_bc(PyObject *__pyx_self, PyObject *__pyx_args, PyObject *__pyx_kwds) {
  PyObject *__pyx_v_v_channel = 0;
  PyObject *__pyx_v_nda_interf = 0;
  PyObject *__pyx_v_d_bits = 0;
  PyObject *__pyx_r = 0;
  __Pyx_RefNannyDeclarations
  __Pyx_RefNannySetupContext("rate_bc (wrapper)", 0);
  {
    static PyObject **__pyx_pyargnames[] = {&__pyx_n_s_v_channel,&__pyx_n_s_nda_interf,&__pyx_n_s_d_bits,0};
    PyObject* values[3] = {0,0,0};
    if (unlikely(__pyx_kwds)) {
      Py_ssize_t kw_args;
      const Py_ssize_t pos_args = PyTuple_GET_SIZE(__pyx_args);
      switch (pos_args) {
        case  3: values[2] = PyTuple_GET_ITEM(__pyx_args, 2);
        case  2: values[1] = PyTuple_GET_ITEM(__pyx_args, 1);
        case  1: values[0] = PyTuple_GET_ITEM(__pyx_args, 0);
        case  0: break;
        default: goto __pyx_L5_argtuple_error;
      }
      kw_args = PyDict_Size(__pyx_kwds);
      switch (pos_args) {
        case  0:
        if (likely((values[0] = PyDict_GetItem(__pyx_kwds, __pyx_n_s_v_channel)) != 0)) kw_args--;
        else goto __pyx_L5_argtuple_error;
        case  1:
        if (likely((values[1] = PyDict_GetItem(__pyx_kwds, __pyx_n_s_nda_interf)) != 0)) kw_args--;
        else {
          __Pyx_RaiseArgtupleInvalid("rate_bc", 1, 3, 3, 1); __PYX_ERR(0, 38, __pyx_L3_error)
        }
        case  2:
        if (likely((values[2] = PyDict_GetItem(__pyx_kwds, __pyx_n_s_d_bits)) != 0)) kw_args--;
        else {
          __Pyx_RaiseArgtupleInvalid("rate_bc", 1, 3, 3, 2); __PYX_ERR(0, 38, __pyx_L3_error)
        }
      }
      if (unlikely(kw_args > 0)) {
        if (unlikely(__Pyx_ParseOptionalKeywords(__pyx_kwds, __pyx_pyargnames, 0, values, pos_args, "rate_bc") < 0)) __PYX_ERR(0, 38, __pyx_L3_error)
      }
    } else if (PyTuple_GET_SIZE(__pyx_args) != 3) {
      goto __pyx_L5_argtuple_error;
    } else {
      values[0] = PyTuple_GET_ITEM(__pyx_args, 0);
      values[1] = PyTuple_GET_ITEM(__pyx_args, 1);
      values[2] = PyTuple_GET_ITEM(__pyx_args, 2);
    }
    __pyx_v_v_channel = values[0];
    __pyx_v_nda_interf = values[1];
    __pyx_v_d_bits = values[2];
  }
  goto __pyx_L4_argument_unpacking_done;
  __pyx_L5_argtuple_error:;
  __Pyx_RaiseArgtupleInvalid("rate_bc", 1, 3, 3, PyTuple_GET_SIZE(__pyx_args)); __PYX_ERR(0, 38, __pyx_L3_error)
  __pyx_L3_error:;
  __Pyx_AddTraceback("dist_recv.rates.rate_bc", __pyx_clineno, __pyx_lineno, __pyx_filename);
  __Pyx_RefNannyFinishContext();
  return NULL;
  __pyx_L4_argument_unpacking_done:;
  __pyx_r = __pyx_pf_9dist_recv_5rates_2rate_bc(__pyx_self, __pyx_v_v_channel, __pyx_v_nda_interf, __pyx_v_d_bits);

  /* function exit code */
  __Pyx_RefNannyFinishContext();
  return __pyx_r;
}

static PyObject *__pyx_pf_9dist_recv_5rates_2rate_bc(CYTHON_UNUSED PyObject *__pyx_self, PyObject *__pyx_v_v_channel, PyObject *__pyx_v_nda_interf, PyObject *__pyx_v_d_bits) {
  PyObject *__pyx_v_d_rate_0 = NULL;
  PyObject *__pyx_v_idx_n = NULL;
  PyObject *__pyx_v_d_rate_n = NULL;
  PyObject *__pyx_v_d_rate_bc = NULL;
  PyObject *__pyx_r = NULL;
  __Pyx_TraceDeclarations
  __Pyx_TraceFrameInit(__pyx_codeobj__4)
  __Pyx_RefNannyDeclarations
  __Pyx_RefNannySetupContext("rate_bc", 0);
  __Pyx_TraceCall("rate_bc", __pyx_f[0], 38, 0, __PYX_ERR(0, 38, __pyx_L1_error));
/* … */
  /* function exit code */
  __pyx_L1_error:;
  __Pyx_XDECREF(__pyx_t_1);
  __Pyx_XDECREF(__pyx_t_2);
  __Pyx_XDECREF(__pyx_t_3);
  __Pyx_XDECREF(__pyx_t_4);
  __Pyx_XDECREF(__pyx_t_5);
  __Pyx_XDECREF(__pyx_t_6);
  __Pyx_XDECREF(__pyx_t_7);
  __Pyx_XDECREF(__pyx_t_8);
  __Pyx_XDECREF(__pyx_t_9);
  __Pyx_XDECREF(__pyx_t_10);
  __Pyx_AddTraceback("dist_recv.rates.rate_bc", __pyx_clineno, __pyx_lineno, __pyx_filename);
  __pyx_r = NULL;
  __pyx_L0:;
  __Pyx_XDECREF(__pyx_v_d_rate_0);
  __Pyx_XDECREF(__pyx_v_idx_n);
  __Pyx_XDECREF(__pyx_v_d_rate_n);
  __Pyx_XDECREF(__pyx_v_d_rate_bc);
  __Pyx_XGIVEREF(__pyx_r);
  __Pyx_TraceReturn(__pyx_r, 0);
  __Pyx_RefNannyFinishContext();
  return __pyx_r;
}
/* … */
  __pyx_tuple__13 = PyTuple_Pack(7, __pyx_n_s_v_channel, __pyx_n_s_nda_interf, __pyx_n_s_d_bits, __pyx_n_s_d_rate_0, __pyx_n_s_idx_n, __pyx_n_s_d_rate_n, __pyx_n_s_d_rate_bc); if (unlikely(!__pyx_tuple__13)) __PYX_ERR(0, 38, __pyx_L1_error)
  __Pyx_GOTREF(__pyx_tuple__13);
  __Pyx_GIVEREF(__pyx_tuple__13);
/* … */
  __pyx_t_2 = PyCFunction_NewEx(&__pyx_mdef_9dist_recv_5rates_3rate_bc, NULL, __pyx_n_s_dist_recv_rates); if (unlikely(!__pyx_t_2)) __PYX_ERR(0, 38, __pyx_L1_error)
  __Pyx_GOTREF(__pyx_t_2);
  if (PyDict_SetItem(__pyx_d, __pyx_n_s_rate_bc, __pyx_t_2) < 0) __PYX_ERR(0, 38, __pyx_L1_error)
  __Pyx_DECREF(__pyx_t_2); __pyx_t_2 = 0;
  __pyx_codeobj__4 = (PyObject*)__Pyx_PyCode_New(3, 0, 7, 0, 0, __pyx_empty_bytes, __pyx_empty_tuple, __pyx_empty_tuple, __pyx_tuple__13, __pyx_empty_tuple, __pyx_empty_tuple, __pyx_kp_s_Users_cdchapm2_Dropbox_Personal, __pyx_n_s_rate_bc, 38, __pyx_empty_bytes); if (unlikely(!__pyx_codeobj__4)) __PYX_ERR(0, 38, __pyx_L1_error)
```

```
 039:     """ Rate for Broadcast distributed receive with reception at base
```

```
 040:         Input:
```

```
 041:             v_channel = Channel vector h (size N+1 x 1)
```

```
 042:             nda_interf = Interference covariance matrix Sigma (size N+1 x N+1)
```

```
 043:             d_bits = Total number of bits used by helpers
```

```
 044:         Output:
```

```
 045:             d_rate = Rate (in bits)
```

```
 046:     """
```

```
+047:     d_rate_0 = np.log2(np.real(1+nda_interf[0, 0] + np.abs(v_channel[0])**2) /
```

```
  __pyx_t_2 = __Pyx_GetModuleGlobalName(__pyx_n_s_np); if (unlikely(!__pyx_t_2)) __PYX_ERR(0, 47, __pyx_L1_error)
  __Pyx_GOTREF(__pyx_t_2);
  __pyx_t_3 = __Pyx_PyObject_GetAttrStr(__pyx_t_2, __pyx_n_s_log2); if (unlikely(!__pyx_t_3)) __PYX_ERR(0, 47, __pyx_L1_error)
  __Pyx_GOTREF(__pyx_t_3);
  __Pyx_DECREF(__pyx_t_2); __pyx_t_2 = 0;
  __pyx_t_4 = __Pyx_GetModuleGlobalName(__pyx_n_s_np); if (unlikely(!__pyx_t_4)) __PYX_ERR(0, 47, __pyx_L1_error)
  __Pyx_GOTREF(__pyx_t_4);
  __pyx_t_5 = __Pyx_PyObject_GetAttrStr(__pyx_t_4, __pyx_n_s_real); if (unlikely(!__pyx_t_5)) __PYX_ERR(0, 47, __pyx_L1_error)
  __Pyx_GOTREF(__pyx_t_5);
  __Pyx_DECREF(__pyx_t_4); __pyx_t_4 = 0;
  __pyx_t_4 = PyObject_GetItem(__pyx_v_nda_interf, __pyx_tuple__5); if (unlikely(!__pyx_t_4)) __PYX_ERR(0, 47, __pyx_L1_error)
  __Pyx_GOTREF(__pyx_t_4);
  __pyx_t_6 = __Pyx_PyInt_AddCObj(__pyx_int_1, __pyx_t_4, 1, 0); if (unlikely(!__pyx_t_6)) __PYX_ERR(0, 47, __pyx_L1_error)
  __Pyx_GOTREF(__pyx_t_6);
  __Pyx_DECREF(__pyx_t_4); __pyx_t_4 = 0;
  __pyx_t_7 = __Pyx_GetModuleGlobalName(__pyx_n_s_np); if (unlikely(!__pyx_t_7)) __PYX_ERR(0, 47, __pyx_L1_error)
  __Pyx_GOTREF(__pyx_t_7);
  __pyx_t_8 = __Pyx_PyObject_GetAttrStr(__pyx_t_7, __pyx_n_s_abs); if (unlikely(!__pyx_t_8)) __PYX_ERR(0, 47, __pyx_L1_error)
  __Pyx_GOTREF(__pyx_t_8);
  __Pyx_DECREF(__pyx_t_7); __pyx_t_7 = 0;
  __pyx_t_7 = __Pyx_GetItemInt(__pyx_v_v_channel, 0, long, 1, __Pyx_PyInt_From_long, 0, 0, 1); if (unlikely(!__pyx_t_7)) __PYX_ERR(0, 47, __pyx_L1_error)
  __Pyx_GOTREF(__pyx_t_7);
  __pyx_t_9 = NULL;
  if (CYTHON_UNPACK_METHODS && unlikely(PyMethod_Check(__pyx_t_8))) {
    __pyx_t_9 = PyMethod_GET_SELF(__pyx_t_8);
    if (likely(__pyx_t_9)) {
      PyObject* function = PyMethod_GET_FUNCTION(__pyx_t_8);
      __Pyx_INCREF(__pyx_t_9);
      __Pyx_INCREF(function);
      __Pyx_DECREF_SET(__pyx_t_8, function);
    }
  }
  if (!__pyx_t_9) {
    __pyx_t_4 = __Pyx_PyObject_CallOneArg(__pyx_t_8, __pyx_t_7); if (unlikely(!__pyx_t_4)) __PYX_ERR(0, 47, __pyx_L1_error)
    __Pyx_DECREF(__pyx_t_7); __pyx_t_7 = 0;
    __Pyx_GOTREF(__pyx_t_4);
  } else {
    #if CYTHON_FAST_PYCALL
    if (PyFunction_Check(__pyx_t_8)) {
      PyObject *__pyx_temp[2] = {__pyx_t_9, __pyx_t_7};
      __pyx_t_4 = __Pyx_PyFunction_FastCall(__pyx_t_8, __pyx_temp+1-1, 1+1); if (unlikely(!__pyx_t_4)) __PYX_ERR(0, 47, __pyx_L1_error)
      __Pyx_XDECREF(__pyx_t_9); __pyx_t_9 = 0;
      __Pyx_GOTREF(__pyx_t_4);
      __Pyx_DECREF(__pyx_t_7); __pyx_t_7 = 0;
    } else
    #endif
    #if CYTHON_FAST_PYCCALL
    if (__Pyx_PyFastCFunction_Check(__pyx_t_8)) {
      PyObject *__pyx_temp[2] = {__pyx_t_9, __pyx_t_7};
      __pyx_t_4 = __Pyx_PyCFunction_FastCall(__pyx_t_8, __pyx_temp+1-1, 1+1); if (unlikely(!__pyx_t_4)) __PYX_ERR(0, 47, __pyx_L1_error)
      __Pyx_XDECREF(__pyx_t_9); __pyx_t_9 = 0;
      __Pyx_GOTREF(__pyx_t_4);
      __Pyx_DECREF(__pyx_t_7); __pyx_t_7 = 0;
    } else
    #endif
    {
      __pyx_t_10 = PyTuple_New(1+1); if (unlikely(!__pyx_t_10)) __PYX_ERR(0, 47, __pyx_L1_error)
      __Pyx_GOTREF(__pyx_t_10);
      __Pyx_GIVEREF(__pyx_t_9); PyTuple_SET_ITEM(__pyx_t_10, 0, __pyx_t_9); __pyx_t_9 = NULL;
      __Pyx_GIVEREF(__pyx_t_7);
      PyTuple_SET_ITEM(__pyx_t_10, 0+1, __pyx_t_7);
      __pyx_t_7 = 0;
      __pyx_t_4 = __Pyx_PyObject_Call(__pyx_t_8, __pyx_t_10, NULL); if (unlikely(!__pyx_t_4)) __PYX_ERR(0, 47, __pyx_L1_error)
      __Pyx_GOTREF(__pyx_t_4);
      __Pyx_DECREF(__pyx_t_10); __pyx_t_10 = 0;
    }
  }
  __Pyx_DECREF(__pyx_t_8); __pyx_t_8 = 0;
  __pyx_t_8 = PyNumber_Power(__pyx_t_4, __pyx_int_2, Py_None); if (unlikely(!__pyx_t_8)) __PYX_ERR(0, 47, __pyx_L1_error)
  __Pyx_GOTREF(__pyx_t_8);
  __Pyx_DECREF(__pyx_t_4); __pyx_t_4 = 0;
  __pyx_t_4 = PyNumber_Add(__pyx_t_6, __pyx_t_8); if (unlikely(!__pyx_t_4)) __PYX_ERR(0, 47, __pyx_L1_error)
  __Pyx_GOTREF(__pyx_t_4);
  __Pyx_DECREF(__pyx_t_6); __pyx_t_6 = 0;
  __Pyx_DECREF(__pyx_t_8); __pyx_t_8 = 0;
  __pyx_t_8 = NULL;
  if (CYTHON_UNPACK_METHODS && unlikely(PyMethod_Check(__pyx_t_5))) {
    __pyx_t_8 = PyMethod_GET_SELF(__pyx_t_5);
    if (likely(__pyx_t_8)) {
      PyObject* function = PyMethod_GET_FUNCTION(__pyx_t_5);
      __Pyx_INCREF(__pyx_t_8);
      __Pyx_INCREF(function);
      __Pyx_DECREF_SET(__pyx_t_5, function);
    }
  }
  if (!__pyx_t_8) {
    __pyx_t_2 = __Pyx_PyObject_CallOneArg(__pyx_t_5, __pyx_t_4); if (unlikely(!__pyx_t_2)) __PYX_ERR(0, 47, __pyx_L1_error)
    __Pyx_DECREF(__pyx_t_4); __pyx_t_4 = 0;
    __Pyx_GOTREF(__pyx_t_2);
  } else {
    #if CYTHON_FAST_PYCALL
    if (PyFunction_Check(__pyx_t_5)) {
      PyObject *__pyx_temp[2] = {__pyx_t_8, __pyx_t_4};
      __pyx_t_2 = __Pyx_PyFunction_FastCall(__pyx_t_5, __pyx_temp+1-1, 1+1); if (unlikely(!__pyx_t_2)) __PYX_ERR(0, 47, __pyx_L1_error)
      __Pyx_XDECREF(__pyx_t_8); __pyx_t_8 = 0;
      __Pyx_GOTREF(__pyx_t_2);
      __Pyx_DECREF(__pyx_t_4); __pyx_t_4 = 0;
    } else
    #endif
    #if CYTHON_FAST_PYCCALL
    if (__Pyx_PyFastCFunction_Check(__pyx_t_5)) {
      PyObject *__pyx_temp[2] = {__pyx_t_8, __pyx_t_4};
      __pyx_t_2 = __Pyx_PyCFunction_FastCall(__pyx_t_5, __pyx_temp+1-1, 1+1); if (unlikely(!__pyx_t_2)) __PYX_ERR(0, 47, __pyx_L1_error)
      __Pyx_XDECREF(__pyx_t_8); __pyx_t_8 = 0;
      __Pyx_GOTREF(__pyx_t_2);
      __Pyx_DECREF(__pyx_t_4); __pyx_t_4 = 0;
    } else
    #endif
    {
      __pyx_t_6 = PyTuple_New(1+1); if (unlikely(!__pyx_t_6)) __PYX_ERR(0, 47, __pyx_L1_error)
      __Pyx_GOTREF(__pyx_t_6);
      __Pyx_GIVEREF(__pyx_t_8); PyTuple_SET_ITEM(__pyx_t_6, 0, __pyx_t_8); __pyx_t_8 = NULL;
      __Pyx_GIVEREF(__pyx_t_4);
      PyTuple_SET_ITEM(__pyx_t_6, 0+1, __pyx_t_4);
      __pyx_t_4 = 0;
      __pyx_t_2 = __Pyx_PyObject_Call(__pyx_t_5, __pyx_t_6, NULL); if (unlikely(!__pyx_t_2)) __PYX_ERR(0, 47, __pyx_L1_error)
      __Pyx_GOTREF(__pyx_t_2);
      __Pyx_DECREF(__pyx_t_6); __pyx_t_6 = 0;
    }
  }
  __Pyx_DECREF(__pyx_t_5); __pyx_t_5 = 0;
/* … */
  __pyx_tuple__5 = PyTuple_Pack(2, __pyx_int_0, __pyx_int_0); if (unlikely(!__pyx_tuple__5)) __PYX_ERR(0, 47, __pyx_L1_error)
  __Pyx_GOTREF(__pyx_tuple__5);
  __Pyx_GIVEREF(__pyx_tuple__5);
/* … */
  __pyx_t_4 = __Pyx_PyNumber_Divide(__pyx_t_2, __pyx_t_5); if (unlikely(!__pyx_t_4)) __PYX_ERR(0, 47, __pyx_L1_error)
  __Pyx_GOTREF(__pyx_t_4);
  __Pyx_DECREF(__pyx_t_2); __pyx_t_2 = 0;
  __Pyx_DECREF(__pyx_t_5); __pyx_t_5 = 0;
  __pyx_t_5 = NULL;
  if (CYTHON_UNPACK_METHODS && unlikely(PyMethod_Check(__pyx_t_3))) {
    __pyx_t_5 = PyMethod_GET_SELF(__pyx_t_3);
    if (likely(__pyx_t_5)) {
      PyObject* function = PyMethod_GET_FUNCTION(__pyx_t_3);
      __Pyx_INCREF(__pyx_t_5);
      __Pyx_INCREF(function);
      __Pyx_DECREF_SET(__pyx_t_3, function);
    }
  }
  if (!__pyx_t_5) {
    __pyx_t_1 = __Pyx_PyObject_CallOneArg(__pyx_t_3, __pyx_t_4); if (unlikely(!__pyx_t_1)) __PYX_ERR(0, 47, __pyx_L1_error)
    __Pyx_DECREF(__pyx_t_4); __pyx_t_4 = 0;
    __Pyx_GOTREF(__pyx_t_1);
  } else {
    #if CYTHON_FAST_PYCALL
    if (PyFunction_Check(__pyx_t_3)) {
      PyObject *__pyx_temp[2] = {__pyx_t_5, __pyx_t_4};
      __pyx_t_1 = __Pyx_PyFunction_FastCall(__pyx_t_3, __pyx_temp+1-1, 1+1); if (unlikely(!__pyx_t_1)) __PYX_ERR(0, 47, __pyx_L1_error)
      __Pyx_XDECREF(__pyx_t_5); __pyx_t_5 = 0;
      __Pyx_GOTREF(__pyx_t_1);
      __Pyx_DECREF(__pyx_t_4); __pyx_t_4 = 0;
    } else
    #endif
    #if CYTHON_FAST_PYCCALL
    if (__Pyx_PyFastCFunction_Check(__pyx_t_3)) {
      PyObject *__pyx_temp[2] = {__pyx_t_5, __pyx_t_4};
      __pyx_t_1 = __Pyx_PyCFunction_FastCall(__pyx_t_3, __pyx_temp+1-1, 1+1); if (unlikely(!__pyx_t_1)) __PYX_ERR(0, 47, __pyx_L1_error)
      __Pyx_XDECREF(__pyx_t_5); __pyx_t_5 = 0;
      __Pyx_GOTREF(__pyx_t_1);
      __Pyx_DECREF(__pyx_t_4); __pyx_t_4 = 0;
    } else
    #endif
    {
      __pyx_t_2 = PyTuple_New(1+1); if (unlikely(!__pyx_t_2)) __PYX_ERR(0, 47, __pyx_L1_error)
      __Pyx_GOTREF(__pyx_t_2);
      __Pyx_GIVEREF(__pyx_t_5); PyTuple_SET_ITEM(__pyx_t_2, 0, __pyx_t_5); __pyx_t_5 = NULL;
      __Pyx_GIVEREF(__pyx_t_4);
      PyTuple_SET_ITEM(__pyx_t_2, 0+1, __pyx_t_4);
      __pyx_t_4 = 0;
      __pyx_t_1 = __Pyx_PyObject_Call(__pyx_t_3, __pyx_t_2, NULL); if (unlikely(!__pyx_t_1)) __PYX_ERR(0, 47, __pyx_L1_error)
      __Pyx_GOTREF(__pyx_t_1);
      __Pyx_DECREF(__pyx_t_2); __pyx_t_2 = 0;
    }
  }
  __Pyx_DECREF(__pyx_t_3); __pyx_t_3 = 0;
  __pyx_v_d_rate_0 = __pyx_t_1;
  __pyx_t_1 = 0;
```

```
+048:                        np.real(1+nda_interf[0, 0]))
```

```
  __pyx_t_6 = __Pyx_GetModuleGlobalName(__pyx_n_s_np); if (unlikely(!__pyx_t_6)) __PYX_ERR(0, 48, __pyx_L1_error)
  __Pyx_GOTREF(__pyx_t_6);
  __pyx_t_4 = __Pyx_PyObject_GetAttrStr(__pyx_t_6, __pyx_n_s_real); if (unlikely(!__pyx_t_4)) __PYX_ERR(0, 48, __pyx_L1_error)
  __Pyx_GOTREF(__pyx_t_4);
  __Pyx_DECREF(__pyx_t_6); __pyx_t_6 = 0;
  __pyx_t_6 = PyObject_GetItem(__pyx_v_nda_interf, __pyx_tuple__6); if (unlikely(!__pyx_t_6)) __PYX_ERR(0, 48, __pyx_L1_error)
  __Pyx_GOTREF(__pyx_t_6);
  __pyx_t_8 = __Pyx_PyInt_AddCObj(__pyx_int_1, __pyx_t_6, 1, 0); if (unlikely(!__pyx_t_8)) __PYX_ERR(0, 48, __pyx_L1_error)
  __Pyx_GOTREF(__pyx_t_8);
  __Pyx_DECREF(__pyx_t_6); __pyx_t_6 = 0;
  __pyx_t_6 = NULL;
  if (CYTHON_UNPACK_METHODS && unlikely(PyMethod_Check(__pyx_t_4))) {
    __pyx_t_6 = PyMethod_GET_SELF(__pyx_t_4);
    if (likely(__pyx_t_6)) {
      PyObject* function = PyMethod_GET_FUNCTION(__pyx_t_4);
      __Pyx_INCREF(__pyx_t_6);
      __Pyx_INCREF(function);
      __Pyx_DECREF_SET(__pyx_t_4, function);
    }
  }
  if (!__pyx_t_6) {
    __pyx_t_5 = __Pyx_PyObject_CallOneArg(__pyx_t_4, __pyx_t_8); if (unlikely(!__pyx_t_5)) __PYX_ERR(0, 48, __pyx_L1_error)
    __Pyx_DECREF(__pyx_t_8); __pyx_t_8 = 0;
    __Pyx_GOTREF(__pyx_t_5);
  } else {
    #if CYTHON_FAST_PYCALL
    if (PyFunction_Check(__pyx_t_4)) {
      PyObject *__pyx_temp[2] = {__pyx_t_6, __pyx_t_8};
      __pyx_t_5 = __Pyx_PyFunction_FastCall(__pyx_t_4, __pyx_temp+1-1, 1+1); if (unlikely(!__pyx_t_5)) __PYX_ERR(0, 48, __pyx_L1_error)
      __Pyx_XDECREF(__pyx_t_6); __pyx_t_6 = 0;
      __Pyx_GOTREF(__pyx_t_5);
      __Pyx_DECREF(__pyx_t_8); __pyx_t_8 = 0;
    } else
    #endif
    #if CYTHON_FAST_PYCCALL
    if (__Pyx_PyFastCFunction_Check(__pyx_t_4)) {
      PyObject *__pyx_temp[2] = {__pyx_t_6, __pyx_t_8};
      __pyx_t_5 = __Pyx_PyCFunction_FastCall(__pyx_t_4, __pyx_temp+1-1, 1+1); if (unlikely(!__pyx_t_5)) __PYX_ERR(0, 48, __pyx_L1_error)
      __Pyx_XDECREF(__pyx_t_6); __pyx_t_6 = 0;
      __Pyx_GOTREF(__pyx_t_5);
      __Pyx_DECREF(__pyx_t_8); __pyx_t_8 = 0;
    } else
    #endif
    {
      __pyx_t_10 = PyTuple_New(1+1); if (unlikely(!__pyx_t_10)) __PYX_ERR(0, 48, __pyx_L1_error)
      __Pyx_GOTREF(__pyx_t_10);
      __Pyx_GIVEREF(__pyx_t_6); PyTuple_SET_ITEM(__pyx_t_10, 0, __pyx_t_6); __pyx_t_6 = NULL;
      __Pyx_GIVEREF(__pyx_t_8);
      PyTuple_SET_ITEM(__pyx_t_10, 0+1, __pyx_t_8);
      __pyx_t_8 = 0;
      __pyx_t_5 = __Pyx_PyObject_Call(__pyx_t_4, __pyx_t_10, NULL); if (unlikely(!__pyx_t_5)) __PYX_ERR(0, 48, __pyx_L1_error)
      __Pyx_GOTREF(__pyx_t_5);
      __Pyx_DECREF(__pyx_t_10); __pyx_t_10 = 0;
    }
  }
  __Pyx_DECREF(__pyx_t_4); __pyx_t_4 = 0;
/* … */
  __pyx_tuple__6 = PyTuple_Pack(2, __pyx_int_0, __pyx_int_0); if (unlikely(!__pyx_tuple__6)) __PYX_ERR(0, 48, __pyx_L1_error)
  __Pyx_GOTREF(__pyx_tuple__6);
  __Pyx_GIVEREF(__pyx_tuple__6);
```

```
+049:     if not (d_rate_0 > 0):
```

```
  __pyx_t_1 = PyObject_RichCompare(__pyx_v_d_rate_0, __pyx_int_0, Py_GT); __Pyx_XGOTREF(__pyx_t_1); if (unlikely(!__pyx_t_1)) __PYX_ERR(0, 49, __pyx_L1_error)
  __pyx_t_11 = __Pyx_PyObject_IsTrue(__pyx_t_1); if (unlikely(__pyx_t_11 < 0)) __PYX_ERR(0, 49, __pyx_L1_error)
  __Pyx_DECREF(__pyx_t_1); __pyx_t_1 = 0;
  __pyx_t_12 = ((!__pyx_t_11) != 0);
  if (__pyx_t_12) {
/* … */
  }
```

```
+050:         d_rate_0 = 0
```

```
    __Pyx_INCREF(__pyx_int_0);
    __Pyx_DECREF_SET(__pyx_v_d_rate_0, __pyx_int_0);
```

```
+051:     idx_n = np.argmax(np.abs(v_channel))
```

```
  __pyx_t_3 = __Pyx_GetModuleGlobalName(__pyx_n_s_np); if (unlikely(!__pyx_t_3)) __PYX_ERR(0, 51, __pyx_L1_error)
  __Pyx_GOTREF(__pyx_t_3);
  __pyx_t_2 = __Pyx_PyObject_GetAttrStr(__pyx_t_3, __pyx_n_s_argmax); if (unlikely(!__pyx_t_2)) __PYX_ERR(0, 51, __pyx_L1_error)
  __Pyx_GOTREF(__pyx_t_2);
  __Pyx_DECREF(__pyx_t_3); __pyx_t_3 = 0;
  __pyx_t_4 = __Pyx_GetModuleGlobalName(__pyx_n_s_np); if (unlikely(!__pyx_t_4)) __PYX_ERR(0, 51, __pyx_L1_error)
  __Pyx_GOTREF(__pyx_t_4);
  __pyx_t_5 = __Pyx_PyObject_GetAttrStr(__pyx_t_4, __pyx_n_s_abs); if (unlikely(!__pyx_t_5)) __PYX_ERR(0, 51, __pyx_L1_error)
  __Pyx_GOTREF(__pyx_t_5);
  __Pyx_DECREF(__pyx_t_4); __pyx_t_4 = 0;
  __pyx_t_4 = NULL;
  if (CYTHON_UNPACK_METHODS && unlikely(PyMethod_Check(__pyx_t_5))) {
    __pyx_t_4 = PyMethod_GET_SELF(__pyx_t_5);
    if (likely(__pyx_t_4)) {
      PyObject* function = PyMethod_GET_FUNCTION(__pyx_t_5);
      __Pyx_INCREF(__pyx_t_4);
      __Pyx_INCREF(function);
      __Pyx_DECREF_SET(__pyx_t_5, function);
    }
  }
  if (!__pyx_t_4) {
    __pyx_t_3 = __Pyx_PyObject_CallOneArg(__pyx_t_5, __pyx_v_v_channel); if (unlikely(!__pyx_t_3)) __PYX_ERR(0, 51, __pyx_L1_error)
    __Pyx_GOTREF(__pyx_t_3);
  } else {
    #if CYTHON_FAST_PYCALL
    if (PyFunction_Check(__pyx_t_5)) {
      PyObject *__pyx_temp[2] = {__pyx_t_4, __pyx_v_v_channel};
      __pyx_t_3 = __Pyx_PyFunction_FastCall(__pyx_t_5, __pyx_temp+1-1, 1+1); if (unlikely(!__pyx_t_3)) __PYX_ERR(0, 51, __pyx_L1_error)
      __Pyx_XDECREF(__pyx_t_4); __pyx_t_4 = 0;
      __Pyx_GOTREF(__pyx_t_3);
    } else
    #endif
    #if CYTHON_FAST_PYCCALL
    if (__Pyx_PyFastCFunction_Check(__pyx_t_5)) {
      PyObject *__pyx_temp[2] = {__pyx_t_4, __pyx_v_v_channel};
      __pyx_t_3 = __Pyx_PyCFunction_FastCall(__pyx_t_5, __pyx_temp+1-1, 1+1); if (unlikely(!__pyx_t_3)) __PYX_ERR(0, 51, __pyx_L1_error)
      __Pyx_XDECREF(__pyx_t_4); __pyx_t_4 = 0;
      __Pyx_GOTREF(__pyx_t_3);
    } else
    #endif
    {
      __pyx_t_10 = PyTuple_New(1+1); if (unlikely(!__pyx_t_10)) __PYX_ERR(0, 51, __pyx_L1_error)
      __Pyx_GOTREF(__pyx_t_10);
      __Pyx_GIVEREF(__pyx_t_4); PyTuple_SET_ITEM(__pyx_t_10, 0, __pyx_t_4); __pyx_t_4 = NULL;
      __Pyx_INCREF(__pyx_v_v_channel);
      __Pyx_GIVEREF(__pyx_v_v_channel);
      PyTuple_SET_ITEM(__pyx_t_10, 0+1, __pyx_v_v_channel);
      __pyx_t_3 = __Pyx_PyObject_Call(__pyx_t_5, __pyx_t_10, NULL); if (unlikely(!__pyx_t_3)) __PYX_ERR(0, 51, __pyx_L1_error)
      __Pyx_GOTREF(__pyx_t_3);
      __Pyx_DECREF(__pyx_t_10); __pyx_t_10 = 0;
    }
  }
  __Pyx_DECREF(__pyx_t_5); __pyx_t_5 = 0;
  __pyx_t_5 = NULL;
  if (CYTHON_UNPACK_METHODS && unlikely(PyMethod_Check(__pyx_t_2))) {
    __pyx_t_5 = PyMethod_GET_SELF(__pyx_t_2);
    if (likely(__pyx_t_5)) {
      PyObject* function = PyMethod_GET_FUNCTION(__pyx_t_2);
      __Pyx_INCREF(__pyx_t_5);
      __Pyx_INCREF(function);
      __Pyx_DECREF_SET(__pyx_t_2, function);
    }
  }
  if (!__pyx_t_5) {
    __pyx_t_1 = __Pyx_PyObject_CallOneArg(__pyx_t_2, __pyx_t_3); if (unlikely(!__pyx_t_1)) __PYX_ERR(0, 51, __pyx_L1_error)
    __Pyx_DECREF(__pyx_t_3); __pyx_t_3 = 0;
    __Pyx_GOTREF(__pyx_t_1);
  } else {
    #if CYTHON_FAST_PYCALL
    if (PyFunction_Check(__pyx_t_2)) {
      PyObject *__pyx_temp[2] = {__pyx_t_5, __pyx_t_3};
      __pyx_t_1 = __Pyx_PyFunction_FastCall(__pyx_t_2, __pyx_temp+1-1, 1+1); if (unlikely(!__pyx_t_1)) __PYX_ERR(0, 51, __pyx_L1_error)
      __Pyx_XDECREF(__pyx_t_5); __pyx_t_5 = 0;
      __Pyx_GOTREF(__pyx_t_1);
      __Pyx_DECREF(__pyx_t_3); __pyx_t_3 = 0;
    } else
    #endif
    #if CYTHON_FAST_PYCCALL
    if (__Pyx_PyFastCFunction_Check(__pyx_t_2)) {
      PyObject *__pyx_temp[2] = {__pyx_t_5, __pyx_t_3};
      __pyx_t_1 = __Pyx_PyCFunction_FastCall(__pyx_t_2, __pyx_temp+1-1, 1+1); if (unlikely(!__pyx_t_1)) __PYX_ERR(0, 51, __pyx_L1_error)
      __Pyx_XDECREF(__pyx_t_5); __pyx_t_5 = 0;
      __Pyx_GOTREF(__pyx_t_1);
      __Pyx_DECREF(__pyx_t_3); __pyx_t_3 = 0;
    } else
    #endif
    {
      __pyx_t_10 = PyTuple_New(1+1); if (unlikely(!__pyx_t_10)) __PYX_ERR(0, 51, __pyx_L1_error)
      __Pyx_GOTREF(__pyx_t_10);
      __Pyx_GIVEREF(__pyx_t_5); PyTuple_SET_ITEM(__pyx_t_10, 0, __pyx_t_5); __pyx_t_5 = NULL;
      __Pyx_GIVEREF(__pyx_t_3);
      PyTuple_SET_ITEM(__pyx_t_10, 0+1, __pyx_t_3);
      __pyx_t_3 = 0;
      __pyx_t_1 = __Pyx_PyObject_Call(__pyx_t_2, __pyx_t_10, NULL); if (unlikely(!__pyx_t_1)) __PYX_ERR(0, 51, __pyx_L1_error)
      __Pyx_GOTREF(__pyx_t_1);
      __Pyx_DECREF(__pyx_t_10); __pyx_t_10 = 0;
    }
  }
  __Pyx_DECREF(__pyx_t_2); __pyx_t_2 = 0;
  __pyx_v_idx_n = __pyx_t_1;
  __pyx_t_1 = 0;
```

```
+052:     d_rate_n = np.log2(np.real(1+nda_interf[idx_n, idx_n] + np.abs(v_channel[idx_n])**2) /
```

```
  __pyx_t_2 = __Pyx_GetModuleGlobalName(__pyx_n_s_np); if (unlikely(!__pyx_t_2)) __PYX_ERR(0, 52, __pyx_L1_error)
  __Pyx_GOTREF(__pyx_t_2);
  __pyx_t_10 = __Pyx_PyObject_GetAttrStr(__pyx_t_2, __pyx_n_s_log2); if (unlikely(!__pyx_t_10)) __PYX_ERR(0, 52, __pyx_L1_error)
  __Pyx_GOTREF(__pyx_t_10);
  __Pyx_DECREF(__pyx_t_2); __pyx_t_2 = 0;
  __pyx_t_3 = __Pyx_GetModuleGlobalName(__pyx_n_s_np); if (unlikely(!__pyx_t_3)) __PYX_ERR(0, 52, __pyx_L1_error)
  __Pyx_GOTREF(__pyx_t_3);
  __pyx_t_5 = __Pyx_PyObject_GetAttrStr(__pyx_t_3, __pyx_n_s_real); if (unlikely(!__pyx_t_5)) __PYX_ERR(0, 52, __pyx_L1_error)
  __Pyx_GOTREF(__pyx_t_5);
  __Pyx_DECREF(__pyx_t_3); __pyx_t_3 = 0;
  __pyx_t_3 = PyTuple_New(2); if (unlikely(!__pyx_t_3)) __PYX_ERR(0, 52, __pyx_L1_error)
  __Pyx_GOTREF(__pyx_t_3);
  __Pyx_INCREF(__pyx_v_idx_n);
  __Pyx_GIVEREF(__pyx_v_idx_n);
  PyTuple_SET_ITEM(__pyx_t_3, 0, __pyx_v_idx_n);
  __Pyx_INCREF(__pyx_v_idx_n);
  __Pyx_GIVEREF(__pyx_v_idx_n);
  PyTuple_SET_ITEM(__pyx_t_3, 1, __pyx_v_idx_n);
  __pyx_t_4 = PyObject_GetItem(__pyx_v_nda_interf, __pyx_t_3); if (unlikely(!__pyx_t_4)) __PYX_ERR(0, 52, __pyx_L1_error)
  __Pyx_GOTREF(__pyx_t_4);
  __Pyx_DECREF(__pyx_t_3); __pyx_t_3 = 0;
  __pyx_t_3 = __Pyx_PyInt_AddCObj(__pyx_int_1, __pyx_t_4, 1, 0); if (unlikely(!__pyx_t_3)) __PYX_ERR(0, 52, __pyx_L1_error)
  __Pyx_GOTREF(__pyx_t_3);
  __Pyx_DECREF(__pyx_t_4); __pyx_t_4 = 0;
  __pyx_t_8 = __Pyx_GetModuleGlobalName(__pyx_n_s_np); if (unlikely(!__pyx_t_8)) __PYX_ERR(0, 52, __pyx_L1_error)
  __Pyx_GOTREF(__pyx_t_8);
  __pyx_t_6 = __Pyx_PyObject_GetAttrStr(__pyx_t_8, __pyx_n_s_abs); if (unlikely(!__pyx_t_6)) __PYX_ERR(0, 52, __pyx_L1_error)
  __Pyx_GOTREF(__pyx_t_6);
  __Pyx_DECREF(__pyx_t_8); __pyx_t_8 = 0;
  __pyx_t_8 = PyObject_GetItem(__pyx_v_v_channel, __pyx_v_idx_n); if (unlikely(!__pyx_t_8)) __PYX_ERR(0, 52, __pyx_L1_error)
  __Pyx_GOTREF(__pyx_t_8);
  __pyx_t_7 = NULL;
  if (CYTHON_UNPACK_METHODS && unlikely(PyMethod_Check(__pyx_t_6))) {
    __pyx_t_7 = PyMethod_GET_SELF(__pyx_t_6);
    if (likely(__pyx_t_7)) {
      PyObject* function = PyMethod_GET_FUNCTION(__pyx_t_6);
      __Pyx_INCREF(__pyx_t_7);
      __Pyx_INCREF(function);
      __Pyx_DECREF_SET(__pyx_t_6, function);
    }
  }
  if (!__pyx_t_7) {
    __pyx_t_4 = __Pyx_PyObject_CallOneArg(__pyx_t_6, __pyx_t_8); if (unlikely(!__pyx_t_4)) __PYX_ERR(0, 52, __pyx_L1_error)
    __Pyx_DECREF(__pyx_t_8); __pyx_t_8 = 0;
    __Pyx_GOTREF(__pyx_t_4);
  } else {
    #if CYTHON_FAST_PYCALL
    if (PyFunction_Check(__pyx_t_6)) {
      PyObject *__pyx_temp[2] = {__pyx_t_7, __pyx_t_8};
      __pyx_t_4 = __Pyx_PyFunction_FastCall(__pyx_t_6, __pyx_temp+1-1, 1+1); if (unlikely(!__pyx_t_4)) __PYX_ERR(0, 52, __pyx_L1_error)
      __Pyx_XDECREF(__pyx_t_7); __pyx_t_7 = 0;
      __Pyx_GOTREF(__pyx_t_4);
      __Pyx_DECREF(__pyx_t_8); __pyx_t_8 = 0;
    } else
    #endif
    #if CYTHON_FAST_PYCCALL
    if (__Pyx_PyFastCFunction_Check(__pyx_t_6)) {
      PyObject *__pyx_temp[2] = {__pyx_t_7, __pyx_t_8};
      __pyx_t_4 = __Pyx_PyCFunction_FastCall(__pyx_t_6, __pyx_temp+1-1, 1+1); if (unlikely(!__pyx_t_4)) __PYX_ERR(0, 52, __pyx_L1_error)
      __Pyx_XDECREF(__pyx_t_7); __pyx_t_7 = 0;
      __Pyx_GOTREF(__pyx_t_4);
      __Pyx_DECREF(__pyx_t_8); __pyx_t_8 = 0;
    } else
    #endif
    {
      __pyx_t_9 = PyTuple_New(1+1); if (unlikely(!__pyx_t_9)) __PYX_ERR(0, 52, __pyx_L1_error)
      __Pyx_GOTREF(__pyx_t_9);
      __Pyx_GIVEREF(__pyx_t_7); PyTuple_SET_ITEM(__pyx_t_9, 0, __pyx_t_7); __pyx_t_7 = NULL;
      __Pyx_GIVEREF(__pyx_t_8);
      PyTuple_SET_ITEM(__pyx_t_9, 0+1, __pyx_t_8);
      __pyx_t_8 = 0;
      __pyx_t_4 = __Pyx_PyObject_Call(__pyx_t_6, __pyx_t_9, NULL); if (unlikely(!__pyx_t_4)) __PYX_ERR(0, 52, __pyx_L1_error)
      __Pyx_GOTREF(__pyx_t_4);
      __Pyx_DECREF(__pyx_t_9); __pyx_t_9 = 0;
    }
  }
  __Pyx_DECREF(__pyx_t_6); __pyx_t_6 = 0;
  __pyx_t_6 = PyNumber_Power(__pyx_t_4, __pyx_int_2, Py_None); if (unlikely(!__pyx_t_6)) __PYX_ERR(0, 52, __pyx_L1_error)
  __Pyx_GOTREF(__pyx_t_6);
  __Pyx_DECREF(__pyx_t_4); __pyx_t_4 = 0;
  __pyx_t_4 = PyNumber_Add(__pyx_t_3, __pyx_t_6); if (unlikely(!__pyx_t_4)) __PYX_ERR(0, 52, __pyx_L1_error)
  __Pyx_GOTREF(__pyx_t_4);
  __Pyx_DECREF(__pyx_t_3); __pyx_t_3 = 0;
  __Pyx_DECREF(__pyx_t_6); __pyx_t_6 = 0;
  __pyx_t_6 = NULL;
  if (CYTHON_UNPACK_METHODS && unlikely(PyMethod_Check(__pyx_t_5))) {
    __pyx_t_6 = PyMethod_GET_SELF(__pyx_t_5);
    if (likely(__pyx_t_6)) {
      PyObject* function = PyMethod_GET_FUNCTION(__pyx_t_5);
      __Pyx_INCREF(__pyx_t_6);
      __Pyx_INCREF(function);
      __Pyx_DECREF_SET(__pyx_t_5, function);
    }
  }
  if (!__pyx_t_6) {
    __pyx_t_2 = __Pyx_PyObject_CallOneArg(__pyx_t_5, __pyx_t_4); if (unlikely(!__pyx_t_2)) __PYX_ERR(0, 52, __pyx_L1_error)
    __Pyx_DECREF(__pyx_t_4); __pyx_t_4 = 0;
    __Pyx_GOTREF(__pyx_t_2);
  } else {
    #if CYTHON_FAST_PYCALL
    if (PyFunction_Check(__pyx_t_5)) {
      PyObject *__pyx_temp[2] = {__pyx_t_6, __pyx_t_4};
      __pyx_t_2 = __Pyx_PyFunction_FastCall(__pyx_t_5, __pyx_temp+1-1, 1+1); if (unlikely(!__pyx_t_2)) __PYX_ERR(0, 52, __pyx_L1_error)
      __Pyx_XDECREF(__pyx_t_6); __pyx_t_6 = 0;
      __Pyx_GOTREF(__pyx_t_2);
      __Pyx_DECREF(__pyx_t_4); __pyx_t_4 = 0;
    } else
    #endif
    #if CYTHON_FAST_PYCCALL
    if (__Pyx_PyFastCFunction_Check(__pyx_t_5)) {
      PyObject *__pyx_temp[2] = {__pyx_t_6, __pyx_t_4};
      __pyx_t_2 = __Pyx_PyCFunction_FastCall(__pyx_t_5, __pyx_temp+1-1, 1+1); if (unlikely(!__pyx_t_2)) __PYX_ERR(0, 52, __pyx_L1_error)
      __Pyx_XDECREF(__pyx_t_6); __pyx_t_6 = 0;
      __Pyx_GOTREF(__pyx_t_2);
      __Pyx_DECREF(__pyx_t_4); __pyx_t_4 = 0;
    } else
    #endif
    {
      __pyx_t_3 = PyTuple_New(1+1); if (unlikely(!__pyx_t_3)) __PYX_ERR(0, 52, __pyx_L1_error)
      __Pyx_GOTREF(__pyx_t_3);
      __Pyx_GIVEREF(__pyx_t_6); PyTuple_SET_ITEM(__pyx_t_3, 0, __pyx_t_6); __pyx_t_6 = NULL;
      __Pyx_GIVEREF(__pyx_t_4);
      PyTuple_SET_ITEM(__pyx_t_3, 0+1, __pyx_t_4);
      __pyx_t_4 = 0;
      __pyx_t_2 = __Pyx_PyObject_Call(__pyx_t_5, __pyx_t_3, NULL); if (unlikely(!__pyx_t_2)) __PYX_ERR(0, 52, __pyx_L1_error)
      __Pyx_GOTREF(__pyx_t_2);
      __Pyx_DECREF(__pyx_t_3); __pyx_t_3 = 0;
    }
  }
  __Pyx_DECREF(__pyx_t_5); __pyx_t_5 = 0;
/* … */
  __pyx_t_4 = __Pyx_PyNumber_Divide(__pyx_t_2, __pyx_t_5); if (unlikely(!__pyx_t_4)) __PYX_ERR(0, 52, __pyx_L1_error)
  __Pyx_GOTREF(__pyx_t_4);
  __Pyx_DECREF(__pyx_t_2); __pyx_t_2 = 0;
  __Pyx_DECREF(__pyx_t_5); __pyx_t_5 = 0;
  __pyx_t_5 = NULL;
  if (CYTHON_UNPACK_METHODS && unlikely(PyMethod_Check(__pyx_t_10))) {
    __pyx_t_5 = PyMethod_GET_SELF(__pyx_t_10);
    if (likely(__pyx_t_5)) {
      PyObject* function = PyMethod_GET_FUNCTION(__pyx_t_10);
      __Pyx_INCREF(__pyx_t_5);
      __Pyx_INCREF(function);
      __Pyx_DECREF_SET(__pyx_t_10, function);
    }
  }
  if (!__pyx_t_5) {
    __pyx_t_1 = __Pyx_PyObject_CallOneArg(__pyx_t_10, __pyx_t_4); if (unlikely(!__pyx_t_1)) __PYX_ERR(0, 52, __pyx_L1_error)
    __Pyx_DECREF(__pyx_t_4); __pyx_t_4 = 0;
    __Pyx_GOTREF(__pyx_t_1);
  } else {
    #if CYTHON_FAST_PYCALL
    if (PyFunction_Check(__pyx_t_10)) {
      PyObject *__pyx_temp[2] = {__pyx_t_5, __pyx_t_4};
      __pyx_t_1 = __Pyx_PyFunction_FastCall(__pyx_t_10, __pyx_temp+1-1, 1+1); if (unlikely(!__pyx_t_1)) __PYX_ERR(0, 52, __pyx_L1_error)
      __Pyx_XDECREF(__pyx_t_5); __pyx_t_5 = 0;
      __Pyx_GOTREF(__pyx_t_1);
      __Pyx_DECREF(__pyx_t_4); __pyx_t_4 = 0;
    } else
    #endif
    #if CYTHON_FAST_PYCCALL
    if (__Pyx_PyFastCFunction_Check(__pyx_t_10)) {
      PyObject *__pyx_temp[2] = {__pyx_t_5, __pyx_t_4};
      __pyx_t_1 = __Pyx_PyCFunction_FastCall(__pyx_t_10, __pyx_temp+1-1, 1+1); if (unlikely(!__pyx_t_1)) __PYX_ERR(0, 52, __pyx_L1_error)
      __Pyx_XDECREF(__pyx_t_5); __pyx_t_5 = 0;
      __Pyx_GOTREF(__pyx_t_1);
      __Pyx_DECREF(__pyx_t_4); __pyx_t_4 = 0;
    } else
    #endif
    {
      __pyx_t_2 = PyTuple_New(1+1); if (unlikely(!__pyx_t_2)) __PYX_ERR(0, 52, __pyx_L1_error)
      __Pyx_GOTREF(__pyx_t_2);
      __Pyx_GIVEREF(__pyx_t_5); PyTuple_SET_ITEM(__pyx_t_2, 0, __pyx_t_5); __pyx_t_5 = NULL;
      __Pyx_GIVEREF(__pyx_t_4);
      PyTuple_SET_ITEM(__pyx_t_2, 0+1, __pyx_t_4);
      __pyx_t_4 = 0;
      __pyx_t_1 = __Pyx_PyObject_Call(__pyx_t_10, __pyx_t_2, NULL); if (unlikely(!__pyx_t_1)) __PYX_ERR(0, 52, __pyx_L1_error)
      __Pyx_GOTREF(__pyx_t_1);
      __Pyx_DECREF(__pyx_t_2); __pyx_t_2 = 0;
    }
  }
  __Pyx_DECREF(__pyx_t_10); __pyx_t_10 = 0;
  __pyx_v_d_rate_n = __pyx_t_1;
  __pyx_t_1 = 0;
```

```
+053:                        np.real(1+nda_interf[idx_n, idx_n]))
```

```
  __pyx_t_3 = __Pyx_GetModuleGlobalName(__pyx_n_s_np); if (unlikely(!__pyx_t_3)) __PYX_ERR(0, 53, __pyx_L1_error)
  __Pyx_GOTREF(__pyx_t_3);
  __pyx_t_4 = __Pyx_PyObject_GetAttrStr(__pyx_t_3, __pyx_n_s_real); if (unlikely(!__pyx_t_4)) __PYX_ERR(0, 53, __pyx_L1_error)
  __Pyx_GOTREF(__pyx_t_4);
  __Pyx_DECREF(__pyx_t_3); __pyx_t_3 = 0;
  __pyx_t_3 = PyTuple_New(2); if (unlikely(!__pyx_t_3)) __PYX_ERR(0, 53, __pyx_L1_error)
  __Pyx_GOTREF(__pyx_t_3);
  __Pyx_INCREF(__pyx_v_idx_n);
  __Pyx_GIVEREF(__pyx_v_idx_n);
  PyTuple_SET_ITEM(__pyx_t_3, 0, __pyx_v_idx_n);
  __Pyx_INCREF(__pyx_v_idx_n);
  __Pyx_GIVEREF(__pyx_v_idx_n);
  PyTuple_SET_ITEM(__pyx_t_3, 1, __pyx_v_idx_n);
  __pyx_t_6 = PyObject_GetItem(__pyx_v_nda_interf, __pyx_t_3); if (unlikely(!__pyx_t_6)) __PYX_ERR(0, 53, __pyx_L1_error)
  __Pyx_GOTREF(__pyx_t_6);
  __Pyx_DECREF(__pyx_t_3); __pyx_t_3 = 0;
  __pyx_t_3 = __Pyx_PyInt_AddCObj(__pyx_int_1, __pyx_t_6, 1, 0); if (unlikely(!__pyx_t_3)) __PYX_ERR(0, 53, __pyx_L1_error)
  __Pyx_GOTREF(__pyx_t_3);
  __Pyx_DECREF(__pyx_t_6); __pyx_t_6 = 0;
  __pyx_t_6 = NULL;
  if (CYTHON_UNPACK_METHODS && unlikely(PyMethod_Check(__pyx_t_4))) {
    __pyx_t_6 = PyMethod_GET_SELF(__pyx_t_4);
    if (likely(__pyx_t_6)) {
      PyObject* function = PyMethod_GET_FUNCTION(__pyx_t_4);
      __Pyx_INCREF(__pyx_t_6);
      __Pyx_INCREF(function);
      __Pyx_DECREF_SET(__pyx_t_4, function);
    }
  }
  if (!__pyx_t_6) {
    __pyx_t_5 = __Pyx_PyObject_CallOneArg(__pyx_t_4, __pyx_t_3); if (unlikely(!__pyx_t_5)) __PYX_ERR(0, 53, __pyx_L1_error)
    __Pyx_DECREF(__pyx_t_3); __pyx_t_3 = 0;
    __Pyx_GOTREF(__pyx_t_5);
  } else {
    #if CYTHON_FAST_PYCALL
    if (PyFunction_Check(__pyx_t_4)) {
      PyObject *__pyx_temp[2] = {__pyx_t_6, __pyx_t_3};
      __pyx_t_5 = __Pyx_PyFunction_FastCall(__pyx_t_4, __pyx_temp+1-1, 1+1); if (unlikely(!__pyx_t_5)) __PYX_ERR(0, 53, __pyx_L1_error)
      __Pyx_XDECREF(__pyx_t_6); __pyx_t_6 = 0;
      __Pyx_GOTREF(__pyx_t_5);
      __Pyx_DECREF(__pyx_t_3); __pyx_t_3 = 0;
    } else
    #endif
    #if CYTHON_FAST_PYCCALL
    if (__Pyx_PyFastCFunction_Check(__pyx_t_4)) {
      PyObject *__pyx_temp[2] = {__pyx_t_6, __pyx_t_3};
      __pyx_t_5 = __Pyx_PyCFunction_FastCall(__pyx_t_4, __pyx_temp+1-1, 1+1); if (unlikely(!__pyx_t_5)) __PYX_ERR(0, 53, __pyx_L1_error)
      __Pyx_XDECREF(__pyx_t_6); __pyx_t_6 = 0;
      __Pyx_GOTREF(__pyx_t_5);
      __Pyx_DECREF(__pyx_t_3); __pyx_t_3 = 0;
    } else
    #endif
    {
      __pyx_t_9 = PyTuple_New(1+1); if (unlikely(!__pyx_t_9)) __PYX_ERR(0, 53, __pyx_L1_error)
      __Pyx_GOTREF(__pyx_t_9);
      __Pyx_GIVEREF(__pyx_t_6); PyTuple_SET_ITEM(__pyx_t_9, 0, __pyx_t_6); __pyx_t_6 = NULL;
      __Pyx_GIVEREF(__pyx_t_3);
      PyTuple_SET_ITEM(__pyx_t_9, 0+1, __pyx_t_3);
      __pyx_t_3 = 0;
      __pyx_t_5 = __Pyx_PyObject_Call(__pyx_t_4, __pyx_t_9, NULL); if (unlikely(!__pyx_t_5)) __PYX_ERR(0, 53, __pyx_L1_error)
      __Pyx_GOTREF(__pyx_t_5);
      __Pyx_DECREF(__pyx_t_9); __pyx_t_9 = 0;
    }
  }
  __Pyx_DECREF(__pyx_t_4); __pyx_t_4 = 0;
```

```
+054:     if not (d_rate_n > 0):
```

```
  __pyx_t_1 = PyObject_RichCompare(__pyx_v_d_rate_n, __pyx_int_0, Py_GT); __Pyx_XGOTREF(__pyx_t_1); if (unlikely(!__pyx_t_1)) __PYX_ERR(0, 54, __pyx_L1_error)
  __pyx_t_12 = __Pyx_PyObject_IsTrue(__pyx_t_1); if (unlikely(__pyx_t_12 < 0)) __PYX_ERR(0, 54, __pyx_L1_error)
  __Pyx_DECREF(__pyx_t_1); __pyx_t_1 = 0;
  __pyx_t_11 = ((!__pyx_t_12) != 0);
  if (__pyx_t_11) {
/* … */
  }
```

```
+055:         d_rate_n = 0
```

```
    __Pyx_INCREF(__pyx_int_0);
    __Pyx_DECREF_SET(__pyx_v_d_rate_n, __pyx_int_0);
```

```
+056:     d_rate_bc = max([d_rate_0, min([d_rate_n, d_bits])])
```

```
  __Pyx_INCREF(__pyx_v_d_bits);
  __pyx_t_1 = __pyx_v_d_bits;
  __Pyx_INCREF(__pyx_v_d_rate_n);
  __pyx_t_10 = __pyx_v_d_rate_n;
  __pyx_t_4 = PyObject_RichCompare(__pyx_t_1, __pyx_t_10, Py_LT); __Pyx_XGOTREF(__pyx_t_4); if (unlikely(!__pyx_t_4)) __PYX_ERR(0, 56, __pyx_L1_error)
  __pyx_t_11 = __Pyx_PyObject_IsTrue(__pyx_t_4); if (unlikely(__pyx_t_11 < 0)) __PYX_ERR(0, 56, __pyx_L1_error)
  __Pyx_DECREF(__pyx_t_4); __pyx_t_4 = 0;
  if (__pyx_t_11) {
    __Pyx_INCREF(__pyx_t_1);
    __pyx_t_2 = __pyx_t_1;
  } else {
    __Pyx_INCREF(__pyx_t_10);
    __pyx_t_2 = __pyx_t_10;
  }
  __Pyx_DECREF(__pyx_t_10); __pyx_t_10 = 0;
  __Pyx_DECREF(__pyx_t_1); __pyx_t_1 = 0;
  __Pyx_INCREF(__pyx_t_2);
  __pyx_t_1 = __pyx_t_2;
  __Pyx_DECREF(__pyx_t_2); __pyx_t_2 = 0;
  __Pyx_INCREF(__pyx_v_d_rate_0);
  __pyx_t_2 = __pyx_v_d_rate_0;
  __pyx_t_4 = PyObject_RichCompare(__pyx_t_1, __pyx_t_2, Py_GT); __Pyx_XGOTREF(__pyx_t_4); if (unlikely(!__pyx_t_4)) __PYX_ERR(0, 56, __pyx_L1_error)
  __pyx_t_11 = __Pyx_PyObject_IsTrue(__pyx_t_4); if (unlikely(__pyx_t_11 < 0)) __PYX_ERR(0, 56, __pyx_L1_error)
  __Pyx_DECREF(__pyx_t_4); __pyx_t_4 = 0;
  if (__pyx_t_11) {
    __Pyx_INCREF(__pyx_t_1);
    __pyx_t_10 = __pyx_t_1;
  } else {
    __Pyx_INCREF(__pyx_t_2);
    __pyx_t_10 = __pyx_t_2;
  }
  __Pyx_DECREF(__pyx_t_2); __pyx_t_2 = 0;
  __Pyx_DECREF(__pyx_t_1); __pyx_t_1 = 0;
  __pyx_t_1 = __pyx_t_10;
  __Pyx_INCREF(__pyx_t_1);
  __Pyx_DECREF(__pyx_t_10); __pyx_t_10 = 0;
  __pyx_v_d_rate_bc = __pyx_t_1;
  __pyx_t_1 = 0;
```

```
+057:     return d_rate_bc
```

```
  __Pyx_XDECREF(__pyx_r);
  __Pyx_INCREF(__pyx_v_d_rate_bc);
  __pyx_r = __pyx_v_d_rate_bc;
  goto __pyx_L0;
```

```
 058:
```

```
 059:
```

```
+060: def rate_ga(v_channel, nda_interf, v_bits):
```

```
/* Python wrapper */
static PyObject *__pyx_pw_9dist_recv_5rates_5rate_ga(PyObject *__pyx_self, PyObject *__pyx_args, PyObject *__pyx_kwds); /*proto*/
static char __pyx_doc_9dist_recv_5rates_4rate_ga[] = " Rate for Gaussian approximation to distributed receive with reception at base\n        Input:\n            v_channel = Channel vector h (size N+1 x 1)\n            nda_interf = Interference covariance matrix Sigma (size N+1 x N+1)\n            v_bits = Helper encoding rates. (size N x 1)\n        Output:\n            d_rate = Rate (in bits)\n    ";
static PyMethodDef __pyx_mdef_9dist_recv_5rates_5rate_ga = {"rate_ga", (PyCFunction)__pyx_pw_9dist_recv_5rates_5rate_ga, METH_VARARGS|METH_KEYWORDS, __pyx_doc_9dist_recv_5rates_4rate_ga};
static PyObject *__pyx_pw_9dist_recv_5rates_5rate_ga(PyObject *__pyx_self, PyObject *__pyx_args, PyObject *__pyx_kwds) {
  PyObject *__pyx_v_v_channel = 0;
  PyObject *__pyx_v_nda_interf = 0;
  PyObject *__pyx_v_v_bits = 0;
  PyObject *__pyx_r = 0;
  __Pyx_RefNannyDeclarations
  __Pyx_RefNannySetupContext("rate_ga (wrapper)", 0);
  {
    static PyObject **__pyx_pyargnames[] = {&__pyx_n_s_v_channel,&__pyx_n_s_nda_interf,&__pyx_n_s_v_bits,0};
    PyObject* values[3] = {0,0,0};
    if (unlikely(__pyx_kwds)) {
      Py_ssize_t kw_args;
      const Py_ssize_t pos_args = PyTuple_GET_SIZE(__pyx_args);
      switch (pos_args) {
        case  3: values[2] = PyTuple_GET_ITEM(__pyx_args, 2);
        case  2: values[1] = PyTuple_GET_ITEM(__pyx_args, 1);
        case  1: values[0] = PyTuple_GET_ITEM(__pyx_args, 0);
        case  0: break;
        default: goto __pyx_L5_argtuple_error;
      }
      kw_args = PyDict_Size(__pyx_kwds);
      switch (pos_args) {
        case  0:
        if (likely((values[0] = PyDict_GetItem(__pyx_kwds, __pyx_n_s_v_channel)) != 0)) kw_args--;
        else goto __pyx_L5_argtuple_error;
        case  1:
        if (likely((values[1] = PyDict_GetItem(__pyx_kwds, __pyx_n_s_nda_interf)) != 0)) kw_args--;
        else {
          __Pyx_RaiseArgtupleInvalid("rate_ga", 1, 3, 3, 1); __PYX_ERR(0, 60, __pyx_L3_error)
        }
        case  2:
        if (likely((values[2] = PyDict_GetItem(__pyx_kwds, __pyx_n_s_v_bits)) != 0)) kw_args--;
        else {
          __Pyx_RaiseArgtupleInvalid("rate_ga", 1, 3, 3, 2); __PYX_ERR(0, 60, __pyx_L3_error)
        }
      }
      if (unlikely(kw_args > 0)) {
        if (unlikely(__Pyx_ParseOptionalKeywords(__pyx_kwds, __pyx_pyargnames, 0, values, pos_args, "rate_ga") < 0)) __PYX_ERR(0, 60, __pyx_L3_error)
      }
    } else if (PyTuple_GET_SIZE(__pyx_args) != 3) {
      goto __pyx_L5_argtuple_error;
    } else {
      values[0] = PyTuple_GET_ITEM(__pyx_args, 0);
      values[1] = PyTuple_GET_ITEM(__pyx_args, 1);
      values[2] = PyTuple_GET_ITEM(__pyx_args, 2);
    }
    __pyx_v_v_channel = values[0];
    __pyx_v_nda_interf = values[1];
    __pyx_v_v_bits = values[2];
  }
  goto __pyx_L4_argument_unpacking_done;
  __pyx_L5_argtuple_error:;
  __Pyx_RaiseArgtupleInvalid("rate_ga", 1, 3, 3, PyTuple_GET_SIZE(__pyx_args)); __PYX_ERR(0, 60, __pyx_L3_error)
  __pyx_L3_error:;
  __Pyx_AddTraceback("dist_recv.rates.rate_ga", __pyx_clineno, __pyx_lineno, __pyx_filename);
  __Pyx_RefNannyFinishContext();
  return NULL;
  __pyx_L4_argument_unpacking_done:;
  __pyx_r = __pyx_pf_9dist_recv_5rates_4rate_ga(__pyx_self, __pyx_v_v_channel, __pyx_v_nda_interf, __pyx_v_v_bits);

  /* function exit code */
  __Pyx_RefNannyFinishContext();
  return __pyx_r;
}

static PyObject *__pyx_pf_9dist_recv_5rates_4rate_ga(CYTHON_UNUSED PyObject *__pyx_self, PyObject *__pyx_v_v_channel, PyObject *__pyx_v_nda_interf, PyObject *__pyx_v_v_bits) {
  PyObject *__pyx_v_v_bits_u = NULL;
  PyObject *__pyx_v_v_using = NULL;
  PyObject *__pyx_v_n_using = NULL;
  PyObject *__pyx_v_v_channel_u = NULL;
  PyObject *__pyx_v_nda_interf_u = NULL;
  PyObject *__pyx_v_nda_receiver_noise = NULL;
  PyObject *__pyx_v_nda_receiver_signal = NULL;
  PyObject *__pyx_v_v_helper_distortion = NULL;
  PyObject *__pyx_v_nda_total_noise = NULL;
  PyObject *__pyx_v_d_rate = NULL;
  PyObject *__pyx_r = NULL;
  __Pyx_TraceDeclarations
  __Pyx_TraceFrameInit(__pyx_codeobj__7)
  __Pyx_RefNannyDeclarations
  __Pyx_RefNannySetupContext("rate_ga", 0);
  __Pyx_TraceCall("rate_ga", __pyx_f[0], 60, 0, __PYX_ERR(0, 60, __pyx_L1_error));
/* … */
  /* function exit code */
  __pyx_L1_error:;
  __Pyx_XDECREF(__pyx_t_1);
  __Pyx_XDECREF(__pyx_t_2);
  __Pyx_XDECREF(__pyx_t_3);
  __Pyx_XDECREF(__pyx_t_4);
  __Pyx_XDECREF(__pyx_t_7);
  __Pyx_XDECREF(__pyx_t_8);
  __Pyx_XDECREF(__pyx_t_9);
  __Pyx_XDECREF(__pyx_t_10);
  __Pyx_XDECREF(__pyx_t_11);
  __Pyx_AddTraceback("dist_recv.rates.rate_ga", __pyx_clineno, __pyx_lineno, __pyx_filename);
  __pyx_r = NULL;
  __pyx_L0:;
  __Pyx_XDECREF(__pyx_v_v_bits_u);
  __Pyx_XDECREF(__pyx_v_v_using);
  __Pyx_XDECREF(__pyx_v_n_using);
  __Pyx_XDECREF(__pyx_v_v_channel_u);
  __Pyx_XDECREF(__pyx_v_nda_interf_u);
  __Pyx_XDECREF(__pyx_v_nda_receiver_noise);
  __Pyx_XDECREF(__pyx_v_nda_receiver_signal);
  __Pyx_XDECREF(__pyx_v_v_helper_distortion);
  __Pyx_XDECREF(__pyx_v_nda_total_noise);
  __Pyx_XDECREF(__pyx_v_d_rate);
  __Pyx_XGIVEREF(__pyx_r);
  __Pyx_TraceReturn(__pyx_r, 0);
  __Pyx_RefNannyFinishContext();
  return __pyx_r;
}
/* … */
  __pyx_tuple__14 = PyTuple_Pack(13, __pyx_n_s_v_channel, __pyx_n_s_nda_interf, __pyx_n_s_v_bits, __pyx_n_s_v_bits_u, __pyx_n_s_v_using, __pyx_n_s_n_using, __pyx_n_s_v_channel_u, __pyx_n_s_nda_interf_u, __pyx_n_s_nda_receiver_noise, __pyx_n_s_nda_receiver_signal, __pyx_n_s_v_helper_distortion, __pyx_n_s_nda_total_noise, __pyx_n_s_d_rate); if (unlikely(!__pyx_tuple__14)) __PYX_ERR(0, 60, __pyx_L1_error)
  __Pyx_GOTREF(__pyx_tuple__14);
  __Pyx_GIVEREF(__pyx_tuple__14);
/* … */
  __pyx_t_2 = PyCFunction_NewEx(&__pyx_mdef_9dist_recv_5rates_5rate_ga, NULL, __pyx_n_s_dist_recv_rates); if (unlikely(!__pyx_t_2)) __PYX_ERR(0, 60, __pyx_L1_error)
  __Pyx_GOTREF(__pyx_t_2);
  if (PyDict_SetItem(__pyx_d, __pyx_n_s_rate_ga, __pyx_t_2) < 0) __PYX_ERR(0, 60, __pyx_L1_error)
  __Pyx_DECREF(__pyx_t_2); __pyx_t_2 = 0;
  __pyx_codeobj__7 = (PyObject*)__Pyx_PyCode_New(3, 0, 13, 0, 0, __pyx_empty_bytes, __pyx_empty_tuple, __pyx_empty_tuple, __pyx_tuple__14, __pyx_empty_tuple, __pyx_empty_tuple, __pyx_kp_s_Users_cdchapm2_Dropbox_Personal, __pyx_n_s_rate_ga, 60, __pyx_empty_bytes); if (unlikely(!__pyx_codeobj__7)) __PYX_ERR(0, 60, __pyx_L1_error)
```

```
 061:     """ Rate for Gaussian approximation to distributed receive with reception at base
```

```
 062:         Input:
```

```
 063:             v_channel = Channel vector h (size N+1 x 1)
```

```
 064:             nda_interf = Interference covariance matrix Sigma (size N+1 x N+1)
```

```
 065:             v_bits = Helper encoding rates. (size N x 1)
```

```
 066:         Output:
```

```
 067:             d_rate = Rate (in bits)
```

```
 068:     """
```

```
 069:
```

```
 070:     # Give up if there are any nans
```

```
+071:     if sum(np.isnan(v_bits)) > 0:
```

```
  __pyx_t_2 = __Pyx_GetModuleGlobalName(__pyx_n_s_np); if (unlikely(!__pyx_t_2)) __PYX_ERR(0, 71, __pyx_L1_error)
  __Pyx_GOTREF(__pyx_t_2);
  __pyx_t_3 = __Pyx_PyObject_GetAttrStr(__pyx_t_2, __pyx_n_s_isnan); if (unlikely(!__pyx_t_3)) __PYX_ERR(0, 71, __pyx_L1_error)
  __Pyx_GOTREF(__pyx_t_3);
  __Pyx_DECREF(__pyx_t_2); __pyx_t_2 = 0;
  __pyx_t_2 = NULL;
  if (CYTHON_UNPACK_METHODS && unlikely(PyMethod_Check(__pyx_t_3))) {
    __pyx_t_2 = PyMethod_GET_SELF(__pyx_t_3);
    if (likely(__pyx_t_2)) {
      PyObject* function = PyMethod_GET_FUNCTION(__pyx_t_3);
      __Pyx_INCREF(__pyx_t_2);
      __Pyx_INCREF(function);
      __Pyx_DECREF_SET(__pyx_t_3, function);
    }
  }
  if (!__pyx_t_2) {
    __pyx_t_1 = __Pyx_PyObject_CallOneArg(__pyx_t_3, __pyx_v_v_bits); if (unlikely(!__pyx_t_1)) __PYX_ERR(0, 71, __pyx_L1_error)
    __Pyx_GOTREF(__pyx_t_1);
  } else {
    #if CYTHON_FAST_PYCALL
    if (PyFunction_Check(__pyx_t_3)) {
      PyObject *__pyx_temp[2] = {__pyx_t_2, __pyx_v_v_bits};
      __pyx_t_1 = __Pyx_PyFunction_FastCall(__pyx_t_3, __pyx_temp+1-1, 1+1); if (unlikely(!__pyx_t_1)) __PYX_ERR(0, 71, __pyx_L1_error)
      __Pyx_XDECREF(__pyx_t_2); __pyx_t_2 = 0;
      __Pyx_GOTREF(__pyx_t_1);
    } else
    #endif
    #if CYTHON_FAST_PYCCALL
    if (__Pyx_PyFastCFunction_Check(__pyx_t_3)) {
      PyObject *__pyx_temp[2] = {__pyx_t_2, __pyx_v_v_bits};
      __pyx_t_1 = __Pyx_PyCFunction_FastCall(__pyx_t_3, __pyx_temp+1-1, 1+1); if (unlikely(!__pyx_t_1)) __PYX_ERR(0, 71, __pyx_L1_error)
      __Pyx_XDECREF(__pyx_t_2); __pyx_t_2 = 0;
      __Pyx_GOTREF(__pyx_t_1);
    } else
    #endif
    {
      __pyx_t_4 = PyTuple_New(1+1); if (unlikely(!__pyx_t_4)) __PYX_ERR(0, 71, __pyx_L1_error)
      __Pyx_GOTREF(__pyx_t_4);
      __Pyx_GIVEREF(__pyx_t_2); PyTuple_SET_ITEM(__pyx_t_4, 0, __pyx_t_2); __pyx_t_2 = NULL;
      __Pyx_INCREF(__pyx_v_v_bits);
      __Pyx_GIVEREF(__pyx_v_v_bits);
      PyTuple_SET_ITEM(__pyx_t_4, 0+1, __pyx_v_v_bits);
      __pyx_t_1 = __Pyx_PyObject_Call(__pyx_t_3, __pyx_t_4, NULL); if (unlikely(!__pyx_t_1)) __PYX_ERR(0, 71, __pyx_L1_error)
      __Pyx_GOTREF(__pyx_t_1);
      __Pyx_DECREF(__pyx_t_4); __pyx_t_4 = 0;
    }
  }
  __Pyx_DECREF(__pyx_t_3); __pyx_t_3 = 0;
  __pyx_t_3 = PyTuple_New(1); if (unlikely(!__pyx_t_3)) __PYX_ERR(0, 71, __pyx_L1_error)
  __Pyx_GOTREF(__pyx_t_3);
  __Pyx_GIVEREF(__pyx_t_1);
  PyTuple_SET_ITEM(__pyx_t_3, 0, __pyx_t_1);
  __pyx_t_1 = 0;
  __pyx_t_1 = __Pyx_PyObject_Call(__pyx_builtin_sum, __pyx_t_3, NULL); if (unlikely(!__pyx_t_1)) __PYX_ERR(0, 71, __pyx_L1_error)
  __Pyx_GOTREF(__pyx_t_1);
  __Pyx_DECREF(__pyx_t_3); __pyx_t_3 = 0;
  __pyx_t_3 = PyObject_RichCompare(__pyx_t_1, __pyx_int_0, Py_GT); __Pyx_XGOTREF(__pyx_t_3); if (unlikely(!__pyx_t_3)) __PYX_ERR(0, 71, __pyx_L1_error)
  __Pyx_DECREF(__pyx_t_1); __pyx_t_1 = 0;
  __pyx_t_5 = __Pyx_PyObject_IsTrue(__pyx_t_3); if (unlikely(__pyx_t_5 < 0)) __PYX_ERR(0, 71, __pyx_L1_error)
  __Pyx_DECREF(__pyx_t_3); __pyx_t_3 = 0;
  if (__pyx_t_5) {
/* … */
  }
```

```
+072:         return 0
```

```
    __Pyx_XDECREF(__pyx_r);
    __Pyx_INCREF(__pyx_int_0);
    __pyx_r = __pyx_int_0;
    goto __pyx_L0;
```

```
 073:
```

```
 074:     # Clip to useful receivers
```

```
+075:     v_bits_u = np.append(float('inf'), v_bits)
```

```
  __pyx_t_1 = __Pyx_GetModuleGlobalName(__pyx_n_s_np); if (unlikely(!__pyx_t_1)) __PYX_ERR(0, 75, __pyx_L1_error)
  __Pyx_GOTREF(__pyx_t_1);
  __pyx_t_4 = __Pyx_PyObject_GetAttrStr(__pyx_t_1, __pyx_n_s_append); if (unlikely(!__pyx_t_4)) __PYX_ERR(0, 75, __pyx_L1_error)
  __Pyx_GOTREF(__pyx_t_4);
  __Pyx_DECREF(__pyx_t_1); __pyx_t_1 = 0;
  __pyx_t_1 = __Pyx_PyNumber_Float(__pyx_n_s_inf); if (unlikely(!__pyx_t_1)) __PYX_ERR(0, 75, __pyx_L1_error)
  __Pyx_GOTREF(__pyx_t_1);
  __pyx_t_2 = NULL;
  __pyx_t_6 = 0;
  if (CYTHON_UNPACK_METHODS && unlikely(PyMethod_Check(__pyx_t_4))) {
    __pyx_t_2 = PyMethod_GET_SELF(__pyx_t_4);
    if (likely(__pyx_t_2)) {
      PyObject* function = PyMethod_GET_FUNCTION(__pyx_t_4);
      __Pyx_INCREF(__pyx_t_2);
      __Pyx_INCREF(function);
      __Pyx_DECREF_SET(__pyx_t_4, function);
      __pyx_t_6 = 1;
    }
  }
  #if CYTHON_FAST_PYCALL
  if (PyFunction_Check(__pyx_t_4)) {
    PyObject *__pyx_temp[3] = {__pyx_t_2, __pyx_t_1, __pyx_v_v_bits};
    __pyx_t_3 = __Pyx_PyFunction_FastCall(__pyx_t_4, __pyx_temp+1-__pyx_t_6, 2+__pyx_t_6); if (unlikely(!__pyx_t_3)) __PYX_ERR(0, 75, __pyx_L1_error)
    __Pyx_XDECREF(__pyx_t_2); __pyx_t_2 = 0;
    __Pyx_GOTREF(__pyx_t_3);
    __Pyx_DECREF(__pyx_t_1); __pyx_t_1 = 0;
  } else
  #endif
  #if CYTHON_FAST_PYCCALL
  if (__Pyx_PyFastCFunction_Check(__pyx_t_4)) {
    PyObject *__pyx_temp[3] = {__pyx_t_2, __pyx_t_1, __pyx_v_v_bits};
    __pyx_t_3 = __Pyx_PyCFunction_FastCall(__pyx_t_4, __pyx_temp+1-__pyx_t_6, 2+__pyx_t_6); if (unlikely(!__pyx_t_3)) __PYX_ERR(0, 75, __pyx_L1_error)
    __Pyx_XDECREF(__pyx_t_2); __pyx_t_2 = 0;
    __Pyx_GOTREF(__pyx_t_3);
    __Pyx_DECREF(__pyx_t_1); __pyx_t_1 = 0;
  } else
  #endif
  {
    __pyx_t_7 = PyTuple_New(2+__pyx_t_6); if (unlikely(!__pyx_t_7)) __PYX_ERR(0, 75, __pyx_L1_error)
    __Pyx_GOTREF(__pyx_t_7);
    if (__pyx_t_2) {
      __Pyx_GIVEREF(__pyx_t_2); PyTuple_SET_ITEM(__pyx_t_7, 0, __pyx_t_2); __pyx_t_2 = NULL;
    }
    __Pyx_GIVEREF(__pyx_t_1);
    PyTuple_SET_ITEM(__pyx_t_7, 0+__pyx_t_6, __pyx_t_1);
    __Pyx_INCREF(__pyx_v_v_bits);
    __Pyx_GIVEREF(__pyx_v_v_bits);
    PyTuple_SET_ITEM(__pyx_t_7, 1+__pyx_t_6, __pyx_v_v_bits);
    __pyx_t_1 = 0;
    __pyx_t_3 = __Pyx_PyObject_Call(__pyx_t_4, __pyx_t_7, NULL); if (unlikely(!__pyx_t_3)) __PYX_ERR(0, 75, __pyx_L1_error)
    __Pyx_GOTREF(__pyx_t_3);
    __Pyx_DECREF(__pyx_t_7); __pyx_t_7 = 0;
  }
  __Pyx_DECREF(__pyx_t_4); __pyx_t_4 = 0;
  __pyx_v_v_bits_u = __pyx_t_3;
  __pyx_t_3 = 0;
```

```
+076:     v_using = v_bits_u > D_MIN_USEFUL_BITRATE
```

```
  __pyx_t_3 = __Pyx_GetModuleGlobalName(__pyx_n_s_D_MIN_USEFUL_BITRATE); if (unlikely(!__pyx_t_3)) __PYX_ERR(0, 76, __pyx_L1_error)
  __Pyx_GOTREF(__pyx_t_3);
  __pyx_t_4 = PyObject_RichCompare(__pyx_v_v_bits_u, __pyx_t_3, Py_GT); __Pyx_XGOTREF(__pyx_t_4); if (unlikely(!__pyx_t_4)) __PYX_ERR(0, 76, __pyx_L1_error)
  __Pyx_DECREF(__pyx_t_3); __pyx_t_3 = 0;
  __pyx_v_v_using = __pyx_t_4;
  __pyx_t_4 = 0;
```

```
+077:     n_using = sum(v_using)
```

```
  __pyx_t_4 = PyTuple_New(1); if (unlikely(!__pyx_t_4)) __PYX_ERR(0, 77, __pyx_L1_error)
  __Pyx_GOTREF(__pyx_t_4);
  __Pyx_INCREF(__pyx_v_v_using);
  __Pyx_GIVEREF(__pyx_v_v_using);
  PyTuple_SET_ITEM(__pyx_t_4, 0, __pyx_v_v_using);
  __pyx_t_3 = __Pyx_PyObject_Call(__pyx_builtin_sum, __pyx_t_4, NULL); if (unlikely(!__pyx_t_3)) __PYX_ERR(0, 77, __pyx_L1_error)
  __Pyx_GOTREF(__pyx_t_3);
  __Pyx_DECREF(__pyx_t_4); __pyx_t_4 = 0;
  __pyx_v_n_using = __pyx_t_3;
  __pyx_t_3 = 0;
```

```
+078:     if n_using == 0:
```

```
  __pyx_t_3 = __Pyx_PyInt_EqObjC(__pyx_v_n_using, __pyx_int_0, 0, 0); if (unlikely(!__pyx_t_3)) __PYX_ERR(0, 78, __pyx_L1_error)
  __Pyx_GOTREF(__pyx_t_3);
  __pyx_t_5 = __Pyx_PyObject_IsTrue(__pyx_t_3); if (unlikely(__pyx_t_5 < 0)) __PYX_ERR(0, 78, __pyx_L1_error)
  __Pyx_DECREF(__pyx_t_3); __pyx_t_3 = 0;
  if (__pyx_t_5) {
/* … */
  }
```

```
+079:         return 0
```

```
    __Pyx_XDECREF(__pyx_r);
    __Pyx_INCREF(__pyx_int_0);
    __pyx_r = __pyx_int_0;
    goto __pyx_L0;
```

```
 080:
```

```
+081:     v_channel_u = v_channel[v_using]
```

```
  __pyx_t_3 = PyObject_GetItem(__pyx_v_v_channel, __pyx_v_v_using); if (unlikely(!__pyx_t_3)) __PYX_ERR(0, 81, __pyx_L1_error)
  __Pyx_GOTREF(__pyx_t_3);
  __pyx_v_v_channel_u = __pyx_t_3;
  __pyx_t_3 = 0;
```

```
+082:     nda_interf_u = nda_interf[v_using][:, v_using]
```

```
  __pyx_t_3 = PyObject_GetItem(__pyx_v_nda_interf, __pyx_v_v_using); if (unlikely(!__pyx_t_3)) __PYX_ERR(0, 82, __pyx_L1_error)
  __Pyx_GOTREF(__pyx_t_3);
  __pyx_t_4 = PyTuple_New(2); if (unlikely(!__pyx_t_4)) __PYX_ERR(0, 82, __pyx_L1_error)
  __Pyx_GOTREF(__pyx_t_4);
  __Pyx_INCREF(__pyx_slice__8);
  __Pyx_GIVEREF(__pyx_slice__8);
  PyTuple_SET_ITEM(__pyx_t_4, 0, __pyx_slice__8);
  __Pyx_INCREF(__pyx_v_v_using);
  __Pyx_GIVEREF(__pyx_v_v_using);
  PyTuple_SET_ITEM(__pyx_t_4, 1, __pyx_v_v_using);
  __pyx_t_7 = PyObject_GetItem(__pyx_t_3, __pyx_t_4); if (unlikely(!__pyx_t_7)) __PYX_ERR(0, 82, __pyx_L1_error)
  __Pyx_GOTREF(__pyx_t_7);
  __Pyx_DECREF(__pyx_t_3); __pyx_t_3 = 0;
  __Pyx_DECREF(__pyx_t_4); __pyx_t_4 = 0;
  __pyx_v_nda_interf_u = __pyx_t_7;
  __pyx_t_7 = 0;
/* … */
  __pyx_slice__8 = PySlice_New(Py_None, Py_None, Py_None); if (unlikely(!__pyx_slice__8)) __PYX_ERR(0, 82, __pyx_L1_error)
  __Pyx_GOTREF(__pyx_slice__8);
  __Pyx_GIVEREF(__pyx_slice__8);
```

```
+083:     v_bits_u = v_bits_u[v_using]
```

```
  __pyx_t_7 = PyObject_GetItem(__pyx_v_v_bits_u, __pyx_v_v_using); if (unlikely(!__pyx_t_7)) __PYX_ERR(0, 83, __pyx_L1_error)
  __Pyx_GOTREF(__pyx_t_7);
  __Pyx_DECREF_SET(__pyx_v_v_bits_u, __pyx_t_7);
  __pyx_t_7 = 0;
```

```
 084:
```

```
 085:     # Receiver thermal noise + interference covariance
```

```
+086:     nda_receiver_noise = nda_interf_u + np.identity(n_using)
```

```
  __pyx_t_4 = __Pyx_GetModuleGlobalName(__pyx_n_s_np); if (unlikely(!__pyx_t_4)) __PYX_ERR(0, 86, __pyx_L1_error)
  __Pyx_GOTREF(__pyx_t_4);
  __pyx_t_3 = __Pyx_PyObject_GetAttrStr(__pyx_t_4, __pyx_n_s_identity); if (unlikely(!__pyx_t_3)) __PYX_ERR(0, 86, __pyx_L1_error)
  __Pyx_GOTREF(__pyx_t_3);
  __Pyx_DECREF(__pyx_t_4); __pyx_t_4 = 0;
  __pyx_t_4 = NULL;
  if (CYTHON_UNPACK_METHODS && unlikely(PyMethod_Check(__pyx_t_3))) {
    __pyx_t_4 = PyMethod_GET_SELF(__pyx_t_3);
    if (likely(__pyx_t_4)) {
      PyObject* function = PyMethod_GET_FUNCTION(__pyx_t_3);
      __Pyx_INCREF(__pyx_t_4);
      __Pyx_INCREF(function);
      __Pyx_DECREF_SET(__pyx_t_3, function);
    }
  }
  if (!__pyx_t_4) {
    __pyx_t_7 = __Pyx_PyObject_CallOneArg(__pyx_t_3, __pyx_v_n_using); if (unlikely(!__pyx_t_7)) __PYX_ERR(0, 86, __pyx_L1_error)
    __Pyx_GOTREF(__pyx_t_7);
  } else {
    #if CYTHON_FAST_PYCALL
    if (PyFunction_Check(__pyx_t_3)) {
      PyObject *__pyx_temp[2] = {__pyx_t_4, __pyx_v_n_using};
      __pyx_t_7 = __Pyx_PyFunction_FastCall(__pyx_t_3, __pyx_temp+1-1, 1+1); if (unlikely(!__pyx_t_7)) __PYX_ERR(0, 86, __pyx_L1_error)
      __Pyx_XDECREF(__pyx_t_4); __pyx_t_4 = 0;
      __Pyx_GOTREF(__pyx_t_7);
    } else
    #endif
    #if CYTHON_FAST_PYCCALL
    if (__Pyx_PyFastCFunction_Check(__pyx_t_3)) {
      PyObject *__pyx_temp[2] = {__pyx_t_4, __pyx_v_n_using};
      __pyx_t_7 = __Pyx_PyCFunction_FastCall(__pyx_t_3, __pyx_temp+1-1, 1+1); if (unlikely(!__pyx_t_7)) __PYX_ERR(0, 86, __pyx_L1_error)
      __Pyx_XDECREF(__pyx_t_4); __pyx_t_4 = 0;
      __Pyx_GOTREF(__pyx_t_7);
    } else
    #endif
    {
      __pyx_t_1 = PyTuple_New(1+1); if (unlikely(!__pyx_t_1)) __PYX_ERR(0, 86, __pyx_L1_error)
      __Pyx_GOTREF(__pyx_t_1);
      __Pyx_GIVEREF(__pyx_t_4); PyTuple_SET_ITEM(__pyx_t_1, 0, __pyx_t_4); __pyx_t_4 = NULL;
      __Pyx_INCREF(__pyx_v_n_using);
      __Pyx_GIVEREF(__pyx_v_n_using);
      PyTuple_SET_ITEM(__pyx_t_1, 0+1, __pyx_v_n_using);
      __pyx_t_7 = __Pyx_PyObject_Call(__pyx_t_3, __pyx_t_1, NULL); if (unlikely(!__pyx_t_7)) __PYX_ERR(0, 86, __pyx_L1_error)
      __Pyx_GOTREF(__pyx_t_7);
      __Pyx_DECREF(__pyx_t_1); __pyx_t_1 = 0;
    }
  }
  __Pyx_DECREF(__pyx_t_3); __pyx_t_3 = 0;
  __pyx_t_3 = PyNumber_Add(__pyx_v_nda_interf_u, __pyx_t_7); if (unlikely(!__pyx_t_3)) __PYX_ERR(0, 86, __pyx_L1_error)
  __Pyx_GOTREF(__pyx_t_3);
  __Pyx_DECREF(__pyx_t_7); __pyx_t_7 = 0;
  __pyx_v_nda_receiver_noise = __pyx_t_3;
  __pyx_t_3 = 0;
```

```
 087:
```

```
 088:     # Receive signal covariance
```

```
+089:     nda_receiver_signal = np.outer(v_channel_u, v_channel_u.conj())
```

```
  __pyx_t_7 = __Pyx_GetModuleGlobalName(__pyx_n_s_np); if (unlikely(!__pyx_t_7)) __PYX_ERR(0, 89, __pyx_L1_error)
  __Pyx_GOTREF(__pyx_t_7);
  __pyx_t_1 = __Pyx_PyObject_GetAttrStr(__pyx_t_7, __pyx_n_s_outer); if (unlikely(!__pyx_t_1)) __PYX_ERR(0, 89, __pyx_L1_error)
  __Pyx_GOTREF(__pyx_t_1);
  __Pyx_DECREF(__pyx_t_7); __pyx_t_7 = 0;
  __pyx_t_4 = __Pyx_PyObject_GetAttrStr(__pyx_v_v_channel_u, __pyx_n_s_conj); if (unlikely(!__pyx_t_4)) __PYX_ERR(0, 89, __pyx_L1_error)
  __Pyx_GOTREF(__pyx_t_4);
  __pyx_t_2 = NULL;
  if (CYTHON_UNPACK_METHODS && likely(PyMethod_Check(__pyx_t_4))) {
    __pyx_t_2 = PyMethod_GET_SELF(__pyx_t_4);
    if (likely(__pyx_t_2)) {
      PyObject* function = PyMethod_GET_FUNCTION(__pyx_t_4);
      __Pyx_INCREF(__pyx_t_2);
      __Pyx_INCREF(function);
      __Pyx_DECREF_SET(__pyx_t_4, function);
    }
  }
  if (__pyx_t_2) {
    __pyx_t_7 = __Pyx_PyObject_CallOneArg(__pyx_t_4, __pyx_t_2); if (unlikely(!__pyx_t_7)) __PYX_ERR(0, 89, __pyx_L1_error)
    __Pyx_DECREF(__pyx_t_2); __pyx_t_2 = 0;
  } else {
    __pyx_t_7 = __Pyx_PyObject_CallNoArg(__pyx_t_4); if (unlikely(!__pyx_t_7)) __PYX_ERR(0, 89, __pyx_L1_error)
  }
  __Pyx_GOTREF(__pyx_t_7);
  __Pyx_DECREF(__pyx_t_4); __pyx_t_4 = 0;
  __pyx_t_4 = NULL;
  __pyx_t_6 = 0;
  if (CYTHON_UNPACK_METHODS && unlikely(PyMethod_Check(__pyx_t_1))) {
    __pyx_t_4 = PyMethod_GET_SELF(__pyx_t_1);
    if (likely(__pyx_t_4)) {
      PyObject* function = PyMethod_GET_FUNCTION(__pyx_t_1);
      __Pyx_INCREF(__pyx_t_4);
      __Pyx_INCREF(function);
      __Pyx_DECREF_SET(__pyx_t_1, function);
      __pyx_t_6 = 1;
    }
  }
  #if CYTHON_FAST_PYCALL
  if (PyFunction_Check(__pyx_t_1)) {
    PyObject *__pyx_temp[3] = {__pyx_t_4, __pyx_v_v_channel_u, __pyx_t_7};
    __pyx_t_3 = __Pyx_PyFunction_FastCall(__pyx_t_1, __pyx_temp+1-__pyx_t_6, 2+__pyx_t_6); if (unlikely(!__pyx_t_3)) __PYX_ERR(0, 89, __pyx_L1_error)
    __Pyx_XDECREF(__pyx_t_4); __pyx_t_4 = 0;
    __Pyx_GOTREF(__pyx_t_3);
    __Pyx_DECREF(__pyx_t_7); __pyx_t_7 = 0;
  } else
  #endif
  #if CYTHON_FAST_PYCCALL
  if (__Pyx_PyFastCFunction_Check(__pyx_t_1)) {
    PyObject *__pyx_temp[3] = {__pyx_t_4, __pyx_v_v_channel_u, __pyx_t_7};
    __pyx_t_3 = __Pyx_PyCFunction_FastCall(__pyx_t_1, __pyx_temp+1-__pyx_t_6, 2+__pyx_t_6); if (unlikely(!__pyx_t_3)) __PYX_ERR(0, 89, __pyx_L1_error)
    __Pyx_XDECREF(__pyx_t_4); __pyx_t_4 = 0;
    __Pyx_GOTREF(__pyx_t_3);
    __Pyx_DECREF(__pyx_t_7); __pyx_t_7 = 0;
  } else
  #endif
  {
    __pyx_t_2 = PyTuple_New(2+__pyx_t_6); if (unlikely(!__pyx_t_2)) __PYX_ERR(0, 89, __pyx_L1_error)
    __Pyx_GOTREF(__pyx_t_2);
    if (__pyx_t_4) {
      __Pyx_GIVEREF(__pyx_t_4); PyTuple_SET_ITEM(__pyx_t_2, 0, __pyx_t_4); __pyx_t_4 = NULL;
    }
    __Pyx_INCREF(__pyx_v_v_channel_u);
    __Pyx_GIVEREF(__pyx_v_v_channel_u);
    PyTuple_SET_ITEM(__pyx_t_2, 0+__pyx_t_6, __pyx_v_v_channel_u);
    __Pyx_GIVEREF(__pyx_t_7);
    PyTuple_SET_ITEM(__pyx_t_2, 1+__pyx_t_6, __pyx_t_7);
    __pyx_t_7 = 0;
    __pyx_t_3 = __Pyx_PyObject_Call(__pyx_t_1, __pyx_t_2, NULL); if (unlikely(!__pyx_t_3)) __PYX_ERR(0, 89, __pyx_L1_error)
    __Pyx_GOTREF(__pyx_t_3);
    __Pyx_DECREF(__pyx_t_2); __pyx_t_2 = 0;
  }
  __Pyx_DECREF(__pyx_t_1); __pyx_t_1 = 0;
  __pyx_v_nda_receiver_signal = __pyx_t_3;
  __pyx_t_3 = 0;
```

```
 090:
```

```
 091:     # Power of distortion due to quantization
```

```
+092:     v_helper_distortion = np.real(np.diag(nda_receiver_signal + nda_receiver_noise))
```

```
  __pyx_t_1 = __Pyx_GetModuleGlobalName(__pyx_n_s_np); if (unlikely(!__pyx_t_1)) __PYX_ERR(0, 92, __pyx_L1_error)
  __Pyx_GOTREF(__pyx_t_1);
  __pyx_t_2 = __Pyx_PyObject_GetAttrStr(__pyx_t_1, __pyx_n_s_real); if (unlikely(!__pyx_t_2)) __PYX_ERR(0, 92, __pyx_L1_error)
  __Pyx_GOTREF(__pyx_t_2);
  __Pyx_DECREF(__pyx_t_1); __pyx_t_1 = 0;
  __pyx_t_7 = __Pyx_GetModuleGlobalName(__pyx_n_s_np); if (unlikely(!__pyx_t_7)) __PYX_ERR(0, 92, __pyx_L1_error)
  __Pyx_GOTREF(__pyx_t_7);
  __pyx_t_4 = __Pyx_PyObject_GetAttrStr(__pyx_t_7, __pyx_n_s_diag); if (unlikely(!__pyx_t_4)) __PYX_ERR(0, 92, __pyx_L1_error)
  __Pyx_GOTREF(__pyx_t_4);
  __Pyx_DECREF(__pyx_t_7); __pyx_t_7 = 0;
  __pyx_t_7 = PyNumber_Add(__pyx_v_nda_receiver_signal, __pyx_v_nda_receiver_noise); if (unlikely(!__pyx_t_7)) __PYX_ERR(0, 92, __pyx_L1_error)
  __Pyx_GOTREF(__pyx_t_7);
  __pyx_t_8 = NULL;
  if (CYTHON_UNPACK_METHODS && unlikely(PyMethod_Check(__pyx_t_4))) {
    __pyx_t_8 = PyMethod_GET_SELF(__pyx_t_4);
    if (likely(__pyx_t_8)) {
      PyObject* function = PyMethod_GET_FUNCTION(__pyx_t_4);
      __Pyx_INCREF(__pyx_t_8);
      __Pyx_INCREF(function);
      __Pyx_DECREF_SET(__pyx_t_4, function);
    }
  }
  if (!__pyx_t_8) {
    __pyx_t_1 = __Pyx_PyObject_CallOneArg(__pyx_t_4, __pyx_t_7); if (unlikely(!__pyx_t_1)) __PYX_ERR(0, 92, __pyx_L1_error)
    __Pyx_DECREF(__pyx_t_7); __pyx_t_7 = 0;
    __Pyx_GOTREF(__pyx_t_1);
  } else {
    #if CYTHON_FAST_PYCALL
    if (PyFunction_Check(__pyx_t_4)) {
      PyObject *__pyx_temp[2] = {__pyx_t_8, __pyx_t_7};
      __pyx_t_1 = __Pyx_PyFunction_FastCall(__pyx_t_4, __pyx_temp+1-1, 1+1); if (unlikely(!__pyx_t_1)) __PYX_ERR(0, 92, __pyx_L1_error)
      __Pyx_XDECREF(__pyx_t_8); __pyx_t_8 = 0;
      __Pyx_GOTREF(__pyx_t_1);
      __Pyx_DECREF(__pyx_t_7); __pyx_t_7 = 0;
    } else
    #endif
    #if CYTHON_FAST_PYCCALL
    if (__Pyx_PyFastCFunction_Check(__pyx_t_4)) {
      PyObject *__pyx_temp[2] = {__pyx_t_8, __pyx_t_7};
      __pyx_t_1 = __Pyx_PyCFunction_FastCall(__pyx_t_4, __pyx_temp+1-1, 1+1); if (unlikely(!__pyx_t_1)) __PYX_ERR(0, 92, __pyx_L1_error)
      __Pyx_XDECREF(__pyx_t_8); __pyx_t_8 = 0;
      __Pyx_GOTREF(__pyx_t_1);
      __Pyx_DECREF(__pyx_t_7); __pyx_t_7 = 0;
    } else
    #endif
    {
      __pyx_t_9 = PyTuple_New(1+1); if (unlikely(!__pyx_t_9)) __PYX_ERR(0, 92, __pyx_L1_error)
      __Pyx_GOTREF(__pyx_t_9);
      __Pyx_GIVEREF(__pyx_t_8); PyTuple_SET_ITEM(__pyx_t_9, 0, __pyx_t_8); __pyx_t_8 = NULL;
      __Pyx_GIVEREF(__pyx_t_7);
      PyTuple_SET_ITEM(__pyx_t_9, 0+1, __pyx_t_7);
      __pyx_t_7 = 0;
      __pyx_t_1 = __Pyx_PyObject_Call(__pyx_t_4, __pyx_t_9, NULL); if (unlikely(!__pyx_t_1)) __PYX_ERR(0, 92, __pyx_L1_error)
      __Pyx_GOTREF(__pyx_t_1);
      __Pyx_DECREF(__pyx_t_9); __pyx_t_9 = 0;
    }
  }
  __Pyx_DECREF(__pyx_t_4); __pyx_t_4 = 0;
  __pyx_t_4 = NULL;
  if (CYTHON_UNPACK_METHODS && unlikely(PyMethod_Check(__pyx_t_2))) {
    __pyx_t_4 = PyMethod_GET_SELF(__pyx_t_2);
    if (likely(__pyx_t_4)) {
      PyObject* function = PyMethod_GET_FUNCTION(__pyx_t_2);
      __Pyx_INCREF(__pyx_t_4);
      __Pyx_INCREF(function);
      __Pyx_DECREF_SET(__pyx_t_2, function);
    }
  }
  if (!__pyx_t_4) {
    __pyx_t_3 = __Pyx_PyObject_CallOneArg(__pyx_t_2, __pyx_t_1); if (unlikely(!__pyx_t_3)) __PYX_ERR(0, 92, __pyx_L1_error)
    __Pyx_DECREF(__pyx_t_1); __pyx_t_1 = 0;
    __Pyx_GOTREF(__pyx_t_3);
  } else {
    #if CYTHON_FAST_PYCALL
    if (PyFunction_Check(__pyx_t_2)) {
      PyObject *__pyx_temp[2] = {__pyx_t_4, __pyx_t_1};
      __pyx_t_3 = __Pyx_PyFunction_FastCall(__pyx_t_2, __pyx_temp+1-1, 1+1); if (unlikely(!__pyx_t_3)) __PYX_ERR(0, 92, __pyx_L1_error)
      __Pyx_XDECREF(__pyx_t_4); __pyx_t_4 = 0;
      __Pyx_GOTREF(__pyx_t_3);
      __Pyx_DECREF(__pyx_t_1); __pyx_t_1 = 0;
    } else
    #endif
    #if CYTHON_FAST_PYCCALL
    if (__Pyx_PyFastCFunction_Check(__pyx_t_2)) {
      PyObject *__pyx_temp[2] = {__pyx_t_4, __pyx_t_1};
      __pyx_t_3 = __Pyx_PyCFunction_FastCall(__pyx_t_2, __pyx_temp+1-1, 1+1); if (unlikely(!__pyx_t_3)) __PYX_ERR(0, 92, __pyx_L1_error)
      __Pyx_XDECREF(__pyx_t_4); __pyx_t_4 = 0;
      __Pyx_GOTREF(__pyx_t_3);
      __Pyx_DECREF(__pyx_t_1); __pyx_t_1 = 0;
    } else
    #endif
    {
      __pyx_t_9 = PyTuple_New(1+1); if (unlikely(!__pyx_t_9)) __PYX_ERR(0, 92, __pyx_L1_error)
      __Pyx_GOTREF(__pyx_t_9);
      __Pyx_GIVEREF(__pyx_t_4); PyTuple_SET_ITEM(__pyx_t_9, 0, __pyx_t_4); __pyx_t_4 = NULL;
      __Pyx_GIVEREF(__pyx_t_1);
      PyTuple_SET_ITEM(__pyx_t_9, 0+1, __pyx_t_1);
      __pyx_t_1 = 0;
      __pyx_t_3 = __Pyx_PyObject_Call(__pyx_t_2, __pyx_t_9, NULL); if (unlikely(!__pyx_t_3)) __PYX_ERR(0, 92, __pyx_L1_error)
      __Pyx_GOTREF(__pyx_t_3);
      __Pyx_DECREF(__pyx_t_9); __pyx_t_9 = 0;
    }
  }
  __Pyx_DECREF(__pyx_t_2); __pyx_t_2 = 0;
  __pyx_v_v_helper_distortion = __pyx_t_3;
  __pyx_t_3 = 0;
```

```
+093:     v_helper_distortion = np.divide(v_helper_distortion, np.power(2, v_bits_u)-1)
```

```
  __pyx_t_2 = __Pyx_GetModuleGlobalName(__pyx_n_s_np); if (unlikely(!__pyx_t_2)) __PYX_ERR(0, 93, __pyx_L1_error)
  __Pyx_GOTREF(__pyx_t_2);
  __pyx_t_9 = __Pyx_PyObject_GetAttrStr(__pyx_t_2, __pyx_n_s_divide); if (unlikely(!__pyx_t_9)) __PYX_ERR(0, 93, __pyx_L1_error)
  __Pyx_GOTREF(__pyx_t_9);
  __Pyx_DECREF(__pyx_t_2); __pyx_t_2 = 0;
  __pyx_t_1 = __Pyx_GetModuleGlobalName(__pyx_n_s_np); if (unlikely(!__pyx_t_1)) __PYX_ERR(0, 93, __pyx_L1_error)
  __Pyx_GOTREF(__pyx_t_1);
  __pyx_t_4 = __Pyx_PyObject_GetAttrStr(__pyx_t_1, __pyx_n_s_power); if (unlikely(!__pyx_t_4)) __PYX_ERR(0, 93, __pyx_L1_error)
  __Pyx_GOTREF(__pyx_t_4);
  __Pyx_DECREF(__pyx_t_1); __pyx_t_1 = 0;
  __pyx_t_1 = NULL;
  __pyx_t_6 = 0;
  if (CYTHON_UNPACK_METHODS && unlikely(PyMethod_Check(__pyx_t_4))) {
    __pyx_t_1 = PyMethod_GET_SELF(__pyx_t_4);
    if (likely(__pyx_t_1)) {
      PyObject* function = PyMethod_GET_FUNCTION(__pyx_t_4);
      __Pyx_INCREF(__pyx_t_1);
      __Pyx_INCREF(function);
      __Pyx_DECREF_SET(__pyx_t_4, function);
      __pyx_t_6 = 1;
    }
  }
  #if CYTHON_FAST_PYCALL
  if (PyFunction_Check(__pyx_t_4)) {
    PyObject *__pyx_temp[3] = {__pyx_t_1, __pyx_int_2, __pyx_v_v_bits_u};
    __pyx_t_2 = __Pyx_PyFunction_FastCall(__pyx_t_4, __pyx_temp+1-__pyx_t_6, 2+__pyx_t_6); if (unlikely(!__pyx_t_2)) __PYX_ERR(0, 93, __pyx_L1_error)
    __Pyx_XDECREF(__pyx_t_1); __pyx_t_1 = 0;
    __Pyx_GOTREF(__pyx_t_2);
  } else
  #endif
  #if CYTHON_FAST_PYCCALL
  if (__Pyx_PyFastCFunction_Check(__pyx_t_4)) {
    PyObject *__pyx_temp[3] = {__pyx_t_1, __pyx_int_2, __pyx_v_v_bits_u};
    __pyx_t_2 = __Pyx_PyCFunction_FastCall(__pyx_t_4, __pyx_temp+1-__pyx_t_6, 2+__pyx_t_6); if (unlikely(!__pyx_t_2)) __PYX_ERR(0, 93, __pyx_L1_error)
    __Pyx_XDECREF(__pyx_t_1); __pyx_t_1 = 0;
    __Pyx_GOTREF(__pyx_t_2);
  } else
  #endif
  {
    __pyx_t_7 = PyTuple_New(2+__pyx_t_6); if (unlikely(!__pyx_t_7)) __PYX_ERR(0, 93, __pyx_L1_error)
    __Pyx_GOTREF(__pyx_t_7);
    if (__pyx_t_1) {
      __Pyx_GIVEREF(__pyx_t_1); PyTuple_SET_ITEM(__pyx_t_7, 0, __pyx_t_1); __pyx_t_1 = NULL;
    }
    __Pyx_INCREF(__pyx_int_2);
    __Pyx_GIVEREF(__pyx_int_2);
    PyTuple_SET_ITEM(__pyx_t_7, 0+__pyx_t_6, __pyx_int_2);
    __Pyx_INCREF(__pyx_v_v_bits_u);
    __Pyx_GIVEREF(__pyx_v_v_bits_u);
    PyTuple_SET_ITEM(__pyx_t_7, 1+__pyx_t_6, __pyx_v_v_bits_u);
    __pyx_t_2 = __Pyx_PyObject_Call(__pyx_t_4, __pyx_t_7, NULL); if (unlikely(!__pyx_t_2)) __PYX_ERR(0, 93, __pyx_L1_error)
    __Pyx_GOTREF(__pyx_t_2);
    __Pyx_DECREF(__pyx_t_7); __pyx_t_7 = 0;
  }
  __Pyx_DECREF(__pyx_t_4); __pyx_t_4 = 0;
  __pyx_t_4 = __Pyx_PyInt_SubtractObjC(__pyx_t_2, __pyx_int_1, 1, 0); if (unlikely(!__pyx_t_4)) __PYX_ERR(0, 93, __pyx_L1_error)
  __Pyx_GOTREF(__pyx_t_4);
  __Pyx_DECREF(__pyx_t_2); __pyx_t_2 = 0;
  __pyx_t_2 = NULL;
  __pyx_t_6 = 0;
  if (CYTHON_UNPACK_METHODS && unlikely(PyMethod_Check(__pyx_t_9))) {
    __pyx_t_2 = PyMethod_GET_SELF(__pyx_t_9);
    if (likely(__pyx_t_2)) {
      PyObject* function = PyMethod_GET_FUNCTION(__pyx_t_9);
      __Pyx_INCREF(__pyx_t_2);
      __Pyx_INCREF(function);
      __Pyx_DECREF_SET(__pyx_t_9, function);
      __pyx_t_6 = 1;
    }
  }
  #if CYTHON_FAST_PYCALL
  if (PyFunction_Check(__pyx_t_9)) {
    PyObject *__pyx_temp[3] = {__pyx_t_2, __pyx_v_v_helper_distortion, __pyx_t_4};
    __pyx_t_3 = __Pyx_PyFunction_FastCall(__pyx_t_9, __pyx_temp+1-__pyx_t_6, 2+__pyx_t_6); if (unlikely(!__pyx_t_3)) __PYX_ERR(0, 93, __pyx_L1_error)
    __Pyx_XDECREF(__pyx_t_2); __pyx_t_2 = 0;
    __Pyx_GOTREF(__pyx_t_3);
    __Pyx_DECREF(__pyx_t_4); __pyx_t_4 = 0;
  } else
  #endif
  #if CYTHON_FAST_PYCCALL
  if (__Pyx_PyFastCFunction_Check(__pyx_t_9)) {
    PyObject *__pyx_temp[3] = {__pyx_t_2, __pyx_v_v_helper_distortion, __pyx_t_4};
    __pyx_t_3 = __Pyx_PyCFunction_FastCall(__pyx_t_9, __pyx_temp+1-__pyx_t_6, 2+__pyx_t_6); if (unlikely(!__pyx_t_3)) __PYX_ERR(0, 93, __pyx_L1_error)
    __Pyx_XDECREF(__pyx_t_2); __pyx_t_2 = 0;
    __Pyx_GOTREF(__pyx_t_3);
    __Pyx_DECREF(__pyx_t_4); __pyx_t_4 = 0;
  } else
  #endif
  {
    __pyx_t_7 = PyTuple_New(2+__pyx_t_6); if (unlikely(!__pyx_t_7)) __PYX_ERR(0, 93, __pyx_L1_error)
    __Pyx_GOTREF(__pyx_t_7);
    if (__pyx_t_2) {
      __Pyx_GIVEREF(__pyx_t_2); PyTuple_SET_ITEM(__pyx_t_7, 0, __pyx_t_2); __pyx_t_2 = NULL;
    }
    __Pyx_INCREF(__pyx_v_v_helper_distortion);
    __Pyx_GIVEREF(__pyx_v_v_helper_distortion);
    PyTuple_SET_ITEM(__pyx_t_7, 0+__pyx_t_6, __pyx_v_v_helper_distortion);
    __Pyx_GIVEREF(__pyx_t_4);
    PyTuple_SET_ITEM(__pyx_t_7, 1+__pyx_t_6, __pyx_t_4);
    __pyx_t_4 = 0;
    __pyx_t_3 = __Pyx_PyObject_Call(__pyx_t_9, __pyx_t_7, NULL); if (unlikely(!__pyx_t_3)) __PYX_ERR(0, 93, __pyx_L1_error)
    __Pyx_GOTREF(__pyx_t_3);
    __Pyx_DECREF(__pyx_t_7); __pyx_t_7 = 0;
  }
  __Pyx_DECREF(__pyx_t_9); __pyx_t_9 = 0;
  __Pyx_DECREF_SET(__pyx_v_v_helper_distortion, __pyx_t_3);
  __pyx_t_3 = 0;
```

```
 094:
```

```
 095:     # Thermal + interference + distortion covariance
```

```
+096:     nda_total_noise = nda_receiver_noise + np.diag(v_helper_distortion)
```

```
  __pyx_t_9 = __Pyx_GetModuleGlobalName(__pyx_n_s_np); if (unlikely(!__pyx_t_9)) __PYX_ERR(0, 96, __pyx_L1_error)
  __Pyx_GOTREF(__pyx_t_9);
  __pyx_t_7 = __Pyx_PyObject_GetAttrStr(__pyx_t_9, __pyx_n_s_diag); if (unlikely(!__pyx_t_7)) __PYX_ERR(0, 96, __pyx_L1_error)
  __Pyx_GOTREF(__pyx_t_7);
  __Pyx_DECREF(__pyx_t_9); __pyx_t_9 = 0;
  __pyx_t_9 = NULL;
  if (CYTHON_UNPACK_METHODS && unlikely(PyMethod_Check(__pyx_t_7))) {
    __pyx_t_9 = PyMethod_GET_SELF(__pyx_t_7);
    if (likely(__pyx_t_9)) {
      PyObject* function = PyMethod_GET_FUNCTION(__pyx_t_7);
      __Pyx_INCREF(__pyx_t_9);
      __Pyx_INCREF(function);
      __Pyx_DECREF_SET(__pyx_t_7, function);
    }
  }
  if (!__pyx_t_9) {
    __pyx_t_3 = __Pyx_PyObject_CallOneArg(__pyx_t_7, __pyx_v_v_helper_distortion); if (unlikely(!__pyx_t_3)) __PYX_ERR(0, 96, __pyx_L1_error)
    __Pyx_GOTREF(__pyx_t_3);
  } else {
    #if CYTHON_FAST_PYCALL
    if (PyFunction_Check(__pyx_t_7)) {
      PyObject *__pyx_temp[2] = {__pyx_t_9, __pyx_v_v_helper_distortion};
      __pyx_t_3 = __Pyx_PyFunction_FastCall(__pyx_t_7, __pyx_temp+1-1, 1+1); if (unlikely(!__pyx_t_3)) __PYX_ERR(0, 96, __pyx_L1_error)
      __Pyx_XDECREF(__pyx_t_9); __pyx_t_9 = 0;
      __Pyx_GOTREF(__pyx_t_3);
    } else
    #endif
    #if CYTHON_FAST_PYCCALL
    if (__Pyx_PyFastCFunction_Check(__pyx_t_7)) {
      PyObject *__pyx_temp[2] = {__pyx_t_9, __pyx_v_v_helper_distortion};
      __pyx_t_3 = __Pyx_PyCFunction_FastCall(__pyx_t_7, __pyx_temp+1-1, 1+1); if (unlikely(!__pyx_t_3)) __PYX_ERR(0, 96, __pyx_L1_error)
      __Pyx_XDECREF(__pyx_t_9); __pyx_t_9 = 0;
      __Pyx_GOTREF(__pyx_t_3);
    } else
    #endif
    {
      __pyx_t_4 = PyTuple_New(1+1); if (unlikely(!__pyx_t_4)) __PYX_ERR(0, 96, __pyx_L1_error)
      __Pyx_GOTREF(__pyx_t_4);
      __Pyx_GIVEREF(__pyx_t_9); PyTuple_SET_ITEM(__pyx_t_4, 0, __pyx_t_9); __pyx_t_9 = NULL;
      __Pyx_INCREF(__pyx_v_v_helper_distortion);
      __Pyx_GIVEREF(__pyx_v_v_helper_distortion);
      PyTuple_SET_ITEM(__pyx_t_4, 0+1, __pyx_v_v_helper_distortion);
      __pyx_t_3 = __Pyx_PyObject_Call(__pyx_t_7, __pyx_t_4, NULL); if (unlikely(!__pyx_t_3)) __PYX_ERR(0, 96, __pyx_L1_error)
      __Pyx_GOTREF(__pyx_t_3);
      __Pyx_DECREF(__pyx_t_4); __pyx_t_4 = 0;
    }
  }
  __Pyx_DECREF(__pyx_t_7); __pyx_t_7 = 0;
  __pyx_t_7 = PyNumber_Add(__pyx_v_nda_receiver_noise, __pyx_t_3); if (unlikely(!__pyx_t_7)) __PYX_ERR(0, 96, __pyx_L1_error)
  __Pyx_GOTREF(__pyx_t_7);
  __Pyx_DECREF(__pyx_t_3); __pyx_t_3 = 0;
  __pyx_v_nda_total_noise = __pyx_t_7;
  __pyx_t_7 = 0;
```

```
 097:
```

```
+098:     d_rate = np.log2(np.real(np.linalg.det(nda_receiver_signal + nda_total_noise)) /
```

```
  __pyx_t_3 = __Pyx_GetModuleGlobalName(__pyx_n_s_np); if (unlikely(!__pyx_t_3)) __PYX_ERR(0, 98, __pyx_L1_error)
  __Pyx_GOTREF(__pyx_t_3);
  __pyx_t_4 = __Pyx_PyObject_GetAttrStr(__pyx_t_3, __pyx_n_s_log2); if (unlikely(!__pyx_t_4)) __PYX_ERR(0, 98, __pyx_L1_error)
  __Pyx_GOTREF(__pyx_t_4);
  __Pyx_DECREF(__pyx_t_3); __pyx_t_3 = 0;
  __pyx_t_9 = __Pyx_GetModuleGlobalName(__pyx_n_s_np); if (unlikely(!__pyx_t_9)) __PYX_ERR(0, 98, __pyx_L1_error)
  __Pyx_GOTREF(__pyx_t_9);
  __pyx_t_2 = __Pyx_PyObject_GetAttrStr(__pyx_t_9, __pyx_n_s_real); if (unlikely(!__pyx_t_2)) __PYX_ERR(0, 98, __pyx_L1_error)
  __Pyx_GOTREF(__pyx_t_2);
  __Pyx_DECREF(__pyx_t_9); __pyx_t_9 = 0;
  __pyx_t_1 = __Pyx_GetModuleGlobalName(__pyx_n_s_np); if (unlikely(!__pyx_t_1)) __PYX_ERR(0, 98, __pyx_L1_error)
  __Pyx_GOTREF(__pyx_t_1);
  __pyx_t_8 = __Pyx_PyObject_GetAttrStr(__pyx_t_1, __pyx_n_s_linalg); if (unlikely(!__pyx_t_8)) __PYX_ERR(0, 98, __pyx_L1_error)
  __Pyx_GOTREF(__pyx_t_8);
  __Pyx_DECREF(__pyx_t_1); __pyx_t_1 = 0;
  __pyx_t_1 = __Pyx_PyObject_GetAttrStr(__pyx_t_8, __pyx_n_s_det); if (unlikely(!__pyx_t_1)) __PYX_ERR(0, 98, __pyx_L1_error)
  __Pyx_GOTREF(__pyx_t_1);
  __Pyx_DECREF(__pyx_t_8); __pyx_t_8 = 0;
  __pyx_t_8 = PyNumber_Add(__pyx_v_nda_receiver_signal, __pyx_v_nda_total_noise); if (unlikely(!__pyx_t_8)) __PYX_ERR(0, 98, __pyx_L1_error)
  __Pyx_GOTREF(__pyx_t_8);
  __pyx_t_10 = NULL;
  if (CYTHON_UNPACK_METHODS && likely(PyMethod_Check(__pyx_t_1))) {
    __pyx_t_10 = PyMethod_GET_SELF(__pyx_t_1);
    if (likely(__pyx_t_10)) {
      PyObject* function = PyMethod_GET_FUNCTION(__pyx_t_1);
      __Pyx_INCREF(__pyx_t_10);
      __Pyx_INCREF(function);
      __Pyx_DECREF_SET(__pyx_t_1, function);
    }
  }
  if (!__pyx_t_10) {
    __pyx_t_9 = __Pyx_PyObject_CallOneArg(__pyx_t_1, __pyx_t_8); if (unlikely(!__pyx_t_9)) __PYX_ERR(0, 98, __pyx_L1_error)
    __Pyx_DECREF(__pyx_t_8); __pyx_t_8 = 0;
    __Pyx_GOTREF(__pyx_t_9);
  } else {
    #if CYTHON_FAST_PYCALL
    if (PyFunction_Check(__pyx_t_1)) {
      PyObject *__pyx_temp[2] = {__pyx_t_10, __pyx_t_8};
      __pyx_t_9 = __Pyx_PyFunction_FastCall(__pyx_t_1, __pyx_temp+1-1, 1+1); if (unlikely(!__pyx_t_9)) __PYX_ERR(0, 98, __pyx_L1_error)
      __Pyx_XDECREF(__pyx_t_10); __pyx_t_10 = 0;
      __Pyx_GOTREF(__pyx_t_9);
      __Pyx_DECREF(__pyx_t_8); __pyx_t_8 = 0;
    } else
    #endif
    #if CYTHON_FAST_PYCCALL
    if (__Pyx_PyFastCFunction_Check(__pyx_t_1)) {
      PyObject *__pyx_temp[2] = {__pyx_t_10, __pyx_t_8};
      __pyx_t_9 = __Pyx_PyCFunction_FastCall(__pyx_t_1, __pyx_temp+1-1, 1+1); if (unlikely(!__pyx_t_9)) __PYX_ERR(0, 98, __pyx_L1_error)
      __Pyx_XDECREF(__pyx_t_10); __pyx_t_10 = 0;
      __Pyx_GOTREF(__pyx_t_9);
      __Pyx_DECREF(__pyx_t_8); __pyx_t_8 = 0;
    } else
    #endif
    {
      __pyx_t_11 = PyTuple_New(1+1); if (unlikely(!__pyx_t_11)) __PYX_ERR(0, 98, __pyx_L1_error)
      __Pyx_GOTREF(__pyx_t_11);
      __Pyx_GIVEREF(__pyx_t_10); PyTuple_SET_ITEM(__pyx_t_11, 0, __pyx_t_10); __pyx_t_10 = NULL;
      __Pyx_GIVEREF(__pyx_t_8);
      PyTuple_SET_ITEM(__pyx_t_11, 0+1, __pyx_t_8);
      __pyx_t_8 = 0;
      __pyx_t_9 = __Pyx_PyObject_Call(__pyx_t_1, __pyx_t_11, NULL); if (unlikely(!__pyx_t_9)) __PYX_ERR(0, 98, __pyx_L1_error)
      __Pyx_GOTREF(__pyx_t_9);
      __Pyx_DECREF(__pyx_t_11); __pyx_t_11 = 0;
    }
  }
  __Pyx_DECREF(__pyx_t_1); __pyx_t_1 = 0;
  __pyx_t_1 = NULL;
  if (CYTHON_UNPACK_METHODS && unlikely(PyMethod_Check(__pyx_t_2))) {
    __pyx_t_1 = PyMethod_GET_SELF(__pyx_t_2);
    if (likely(__pyx_t_1)) {
      PyObject* function = PyMethod_GET_FUNCTION(__pyx_t_2);
      __Pyx_INCREF(__pyx_t_1);
      __Pyx_INCREF(function);
      __Pyx_DECREF_SET(__pyx_t_2, function);
    }
  }
  if (!__pyx_t_1) {
    __pyx_t_3 = __Pyx_PyObject_CallOneArg(__pyx_t_2, __pyx_t_9); if (unlikely(!__pyx_t_3)) __PYX_ERR(0, 98, __pyx_L1_error)
    __Pyx_DECREF(__pyx_t_9); __pyx_t_9 = 0;
    __Pyx_GOTREF(__pyx_t_3);
  } else {
    #if CYTHON_FAST_PYCALL
    if (PyFunction_Check(__pyx_t_2)) {
      PyObject *__pyx_temp[2] = {__pyx_t_1, __pyx_t_9};
      __pyx_t_3 = __Pyx_PyFunction_FastCall(__pyx_t_2, __pyx_temp+1-1, 1+1); if (unlikely(!__pyx_t_3)) __PYX_ERR(0, 98, __pyx_L1_error)
      __Pyx_XDECREF(__pyx_t_1); __pyx_t_1 = 0;
      __Pyx_GOTREF(__pyx_t_3);
      __Pyx_DECREF(__pyx_t_9); __pyx_t_9 = 0;
    } else
    #endif
    #if CYTHON_FAST_PYCCALL
    if (__Pyx_PyFastCFunction_Check(__pyx_t_2)) {
      PyObject *__pyx_temp[2] = {__pyx_t_1, __pyx_t_9};
      __pyx_t_3 = __Pyx_PyCFunction_FastCall(__pyx_t_2, __pyx_temp+1-1, 1+1); if (unlikely(!__pyx_t_3)) __PYX_ERR(0, 98, __pyx_L1_error)
      __Pyx_XDECREF(__pyx_t_1); __pyx_t_1 = 0;
      __Pyx_GOTREF(__pyx_t_3);
      __Pyx_DECREF(__pyx_t_9); __pyx_t_9 = 0;
    } else
    #endif
    {
      __pyx_t_11 = PyTuple_New(1+1); if (unlikely(!__pyx_t_11)) __PYX_ERR(0, 98, __pyx_L1_error)
      __Pyx_GOTREF(__pyx_t_11);
      __Pyx_GIVEREF(__pyx_t_1); PyTuple_SET_ITEM(__pyx_t_11, 0, __pyx_t_1); __pyx_t_1 = NULL;
      __Pyx_GIVEREF(__pyx_t_9);
      PyTuple_SET_ITEM(__pyx_t_11, 0+1, __pyx_t_9);
      __pyx_t_9 = 0;
      __pyx_t_3 = __Pyx_PyObject_Call(__pyx_t_2, __pyx_t_11, NULL); if (unlikely(!__pyx_t_3)) __PYX_ERR(0, 98, __pyx_L1_error)
      __Pyx_GOTREF(__pyx_t_3);
      __Pyx_DECREF(__pyx_t_11); __pyx_t_11 = 0;
    }
  }
  __Pyx_DECREF(__pyx_t_2); __pyx_t_2 = 0;
/* … */
  __pyx_t_9 = __Pyx_PyNumber_Divide(__pyx_t_3, __pyx_t_2); if (unlikely(!__pyx_t_9)) __PYX_ERR(0, 98, __pyx_L1_error)
  __Pyx_GOTREF(__pyx_t_9);
  __Pyx_DECREF(__pyx_t_3); __pyx_t_3 = 0;
  __Pyx_DECREF(__pyx_t_2); __pyx_t_2 = 0;
  __pyx_t_2 = NULL;
  if (CYTHON_UNPACK_METHODS && unlikely(PyMethod_Check(__pyx_t_4))) {
    __pyx_t_2 = PyMethod_GET_SELF(__pyx_t_4);
    if (likely(__pyx_t_2)) {
      PyObject* function = PyMethod_GET_FUNCTION(__pyx_t_4);
      __Pyx_INCREF(__pyx_t_2);
      __Pyx_INCREF(function);
      __Pyx_DECREF_SET(__pyx_t_4, function);
    }
  }
  if (!__pyx_t_2) {
    __pyx_t_7 = __Pyx_PyObject_CallOneArg(__pyx_t_4, __pyx_t_9); if (unlikely(!__pyx_t_7)) __PYX_ERR(0, 98, __pyx_L1_error)
    __Pyx_DECREF(__pyx_t_9); __pyx_t_9 = 0;
    __Pyx_GOTREF(__pyx_t_7);
  } else {
    #if CYTHON_FAST_PYCALL
    if (PyFunction_Check(__pyx_t_4)) {
      PyObject *__pyx_temp[2] = {__pyx_t_2, __pyx_t_9};
      __pyx_t_7 = __Pyx_PyFunction_FastCall(__pyx_t_4, __pyx_temp+1-1, 1+1); if (unlikely(!__pyx_t_7)) __PYX_ERR(0, 98, __pyx_L1_error)
      __Pyx_XDECREF(__pyx_t_2); __pyx_t_2 = 0;
      __Pyx_GOTREF(__pyx_t_7);
      __Pyx_DECREF(__pyx_t_9); __pyx_t_9 = 0;
    } else
    #endif
    #if CYTHON_FAST_PYCCALL
    if (__Pyx_PyFastCFunction_Check(__pyx_t_4)) {
      PyObject *__pyx_temp[2] = {__pyx_t_2, __pyx_t_9};
      __pyx_t_7 = __Pyx_PyCFunction_FastCall(__pyx_t_4, __pyx_temp+1-1, 1+1); if (unlikely(!__pyx_t_7)) __PYX_ERR(0, 98, __pyx_L1_error)
      __Pyx_XDECREF(__pyx_t_2); __pyx_t_2 = 0;
      __Pyx_GOTREF(__pyx_t_7);
      __Pyx_DECREF(__pyx_t_9); __pyx_t_9 = 0;
    } else
    #endif
    {
      __pyx_t_3 = PyTuple_New(1+1); if (unlikely(!__pyx_t_3)) __PYX_ERR(0, 98, __pyx_L1_error)
      __Pyx_GOTREF(__pyx_t_3);
      __Pyx_GIVEREF(__pyx_t_2); PyTuple_SET_ITEM(__pyx_t_3, 0, __pyx_t_2); __pyx_t_2 = NULL;
      __Pyx_GIVEREF(__pyx_t_9);
      PyTuple_SET_ITEM(__pyx_t_3, 0+1, __pyx_t_9);
      __pyx_t_9 = 0;
      __pyx_t_7 = __Pyx_PyObject_Call(__pyx_t_4, __pyx_t_3, NULL); if (unlikely(!__pyx_t_7)) __PYX_ERR(0, 98, __pyx_L1_error)
      __Pyx_GOTREF(__pyx_t_7);
      __Pyx_DECREF(__pyx_t_3); __pyx_t_3 = 0;
    }
  }
  __Pyx_DECREF(__pyx_t_4); __pyx_t_4 = 0;
  __pyx_v_d_rate = __pyx_t_7;
  __pyx_t_7 = 0;
```

```
+099:                      np.real(np.linalg.det(nda_total_noise)))
```

```
  __pyx_t_11 = __Pyx_GetModuleGlobalName(__pyx_n_s_np); if (unlikely(!__pyx_t_11)) __PYX_ERR(0, 99, __pyx_L1_error)
  __Pyx_GOTREF(__pyx_t_11);
  __pyx_t_9 = __Pyx_PyObject_GetAttrStr(__pyx_t_11, __pyx_n_s_real); if (unlikely(!__pyx_t_9)) __PYX_ERR(0, 99, __pyx_L1_error)
  __Pyx_GOTREF(__pyx_t_9);
  __Pyx_DECREF(__pyx_t_11); __pyx_t_11 = 0;
  __pyx_t_1 = __Pyx_GetModuleGlobalName(__pyx_n_s_np); if (unlikely(!__pyx_t_1)) __PYX_ERR(0, 99, __pyx_L1_error)
  __Pyx_GOTREF(__pyx_t_1);
  __pyx_t_8 = __Pyx_PyObject_GetAttrStr(__pyx_t_1, __pyx_n_s_linalg); if (unlikely(!__pyx_t_8)) __PYX_ERR(0, 99, __pyx_L1_error)
  __Pyx_GOTREF(__pyx_t_8);
  __Pyx_DECREF(__pyx_t_1); __pyx_t_1 = 0;
  __pyx_t_1 = __Pyx_PyObject_GetAttrStr(__pyx_t_8, __pyx_n_s_det); if (unlikely(!__pyx_t_1)) __PYX_ERR(0, 99, __pyx_L1_error)
  __Pyx_GOTREF(__pyx_t_1);
  __Pyx_DECREF(__pyx_t_8); __pyx_t_8 = 0;
  __pyx_t_8 = NULL;
  if (CYTHON_UNPACK_METHODS && likely(PyMethod_Check(__pyx_t_1))) {
    __pyx_t_8 = PyMethod_GET_SELF(__pyx_t_1);
    if (likely(__pyx_t_8)) {
      PyObject* function = PyMethod_GET_FUNCTION(__pyx_t_1);
      __Pyx_INCREF(__pyx_t_8);
      __Pyx_INCREF(function);
      __Pyx_DECREF_SET(__pyx_t_1, function);
    }
  }
  if (!__pyx_t_8) {
    __pyx_t_11 = __Pyx_PyObject_CallOneArg(__pyx_t_1, __pyx_v_nda_total_noise); if (unlikely(!__pyx_t_11)) __PYX_ERR(0, 99, __pyx_L1_error)
    __Pyx_GOTREF(__pyx_t_11);
  } else {
    #if CYTHON_FAST_PYCALL
    if (PyFunction_Check(__pyx_t_1)) {
      PyObject *__pyx_temp[2] = {__pyx_t_8, __pyx_v_nda_total_noise};
      __pyx_t_11 = __Pyx_PyFunction_FastCall(__pyx_t_1, __pyx_temp+1-1, 1+1); if (unlikely(!__pyx_t_11)) __PYX_ERR(0, 99, __pyx_L1_error)
      __Pyx_XDECREF(__pyx_t_8); __pyx_t_8 = 0;
      __Pyx_GOTREF(__pyx_t_11);
    } else
    #endif
    #if CYTHON_FAST_PYCCALL
    if (__Pyx_PyFastCFunction_Check(__pyx_t_1)) {
      PyObject *__pyx_temp[2] = {__pyx_t_8, __pyx_v_nda_total_noise};
      __pyx_t_11 = __Pyx_PyCFunction_FastCall(__pyx_t_1, __pyx_temp+1-1, 1+1); if (unlikely(!__pyx_t_11)) __PYX_ERR(0, 99, __pyx_L1_error)
      __Pyx_XDECREF(__pyx_t_8); __pyx_t_8 = 0;
      __Pyx_GOTREF(__pyx_t_11);
    } else
    #endif
    {
      __pyx_t_10 = PyTuple_New(1+1); if (unlikely(!__pyx_t_10)) __PYX_ERR(0, 99, __pyx_L1_error)
      __Pyx_GOTREF(__pyx_t_10);
      __Pyx_GIVEREF(__pyx_t_8); PyTuple_SET_ITEM(__pyx_t_10, 0, __pyx_t_8); __pyx_t_8 = NULL;
      __Pyx_INCREF(__pyx_v_nda_total_noise);
      __Pyx_GIVEREF(__pyx_v_nda_total_noise);
      PyTuple_SET_ITEM(__pyx_t_10, 0+1, __pyx_v_nda_total_noise);
      __pyx_t_11 = __Pyx_PyObject_Call(__pyx_t_1, __pyx_t_10, NULL); if (unlikely(!__pyx_t_11)) __PYX_ERR(0, 99, __pyx_L1_error)
      __Pyx_GOTREF(__pyx_t_11);
      __Pyx_DECREF(__pyx_t_10); __pyx_t_10 = 0;
    }
  }
  __Pyx_DECREF(__pyx_t_1); __pyx_t_1 = 0;
  __pyx_t_1 = NULL;
  if (CYTHON_UNPACK_METHODS && unlikely(PyMethod_Check(__pyx_t_9))) {
    __pyx_t_1 = PyMethod_GET_SELF(__pyx_t_9);
    if (likely(__pyx_t_1)) {
      PyObject* function = PyMethod_GET_FUNCTION(__pyx_t_9);
      __Pyx_INCREF(__pyx_t_1);
      __Pyx_INCREF(function);
      __Pyx_DECREF_SET(__pyx_t_9, function);
    }
  }
  if (!__pyx_t_1) {
    __pyx_t_2 = __Pyx_PyObject_CallOneArg(__pyx_t_9, __pyx_t_11); if (unlikely(!__pyx_t_2)) __PYX_ERR(0, 99, __pyx_L1_error)
    __Pyx_DECREF(__pyx_t_11); __pyx_t_11 = 0;
    __Pyx_GOTREF(__pyx_t_2);
  } else {
    #if CYTHON_FAST_PYCALL
    if (PyFunction_Check(__pyx_t_9)) {
      PyObject *__pyx_temp[2] = {__pyx_t_1, __pyx_t_11};
      __pyx_t_2 = __Pyx_PyFunction_FastCall(__pyx_t_9, __pyx_temp+1-1, 1+1); if (unlikely(!__pyx_t_2)) __PYX_ERR(0, 99, __pyx_L1_error)
      __Pyx_XDECREF(__pyx_t_1); __pyx_t_1 = 0;
      __Pyx_GOTREF(__pyx_t_2);
      __Pyx_DECREF(__pyx_t_11); __pyx_t_11 = 0;
    } else
    #endif
    #if CYTHON_FAST_PYCCALL
    if (__Pyx_PyFastCFunction_Check(__pyx_t_9)) {
      PyObject *__pyx_temp[2] = {__pyx_t_1, __pyx_t_11};
      __pyx_t_2 = __Pyx_PyCFunction_FastCall(__pyx_t_9, __pyx_temp+1-1, 1+1); if (unlikely(!__pyx_t_2)) __PYX_ERR(0, 99, __pyx_L1_error)
      __Pyx_XDECREF(__pyx_t_1); __pyx_t_1 = 0;
      __Pyx_GOTREF(__pyx_t_2);
      __Pyx_DECREF(__pyx_t_11); __pyx_t_11 = 0;
    } else
    #endif
    {
      __pyx_t_10 = PyTuple_New(1+1); if (unlikely(!__pyx_t_10)) __PYX_ERR(0, 99, __pyx_L1_error)
      __Pyx_GOTREF(__pyx_t_10);
      __Pyx_GIVEREF(__pyx_t_1); PyTuple_SET_ITEM(__pyx_t_10, 0, __pyx_t_1); __pyx_t_1 = NULL;
      __Pyx_GIVEREF(__pyx_t_11);
      PyTuple_SET_ITEM(__pyx_t_10, 0+1, __pyx_t_11);
      __pyx_t_11 = 0;
      __pyx_t_2 = __Pyx_PyObject_Call(__pyx_t_9, __pyx_t_10, NULL); if (unlikely(!__pyx_t_2)) __PYX_ERR(0, 99, __pyx_L1_error)
      __Pyx_GOTREF(__pyx_t_2);
      __Pyx_DECREF(__pyx_t_10); __pyx_t_10 = 0;
    }
  }
  __Pyx_DECREF(__pyx_t_9); __pyx_t_9 = 0;
```

```
 100:
```

```
+101:     if not (np.isreal(d_rate)):
```

```
  __pyx_t_4 = __Pyx_GetModuleGlobalName(__pyx_n_s_np); if (unlikely(!__pyx_t_4)) __PYX_ERR(0, 101, __pyx_L1_error)
  __Pyx_GOTREF(__pyx_t_4);
  __pyx_t_3 = __Pyx_PyObject_GetAttrStr(__pyx_t_4, __pyx_n_s_isreal); if (unlikely(!__pyx_t_3)) __PYX_ERR(0, 101, __pyx_L1_error)
  __Pyx_GOTREF(__pyx_t_3);
  __Pyx_DECREF(__pyx_t_4); __pyx_t_4 = 0;
  __pyx_t_4 = NULL;
  if (CYTHON_UNPACK_METHODS && unlikely(PyMethod_Check(__pyx_t_3))) {
    __pyx_t_4 = PyMethod_GET_SELF(__pyx_t_3);
    if (likely(__pyx_t_4)) {
      PyObject* function = PyMethod_GET_FUNCTION(__pyx_t_3);
      __Pyx_INCREF(__pyx_t_4);
      __Pyx_INCREF(function);
      __Pyx_DECREF_SET(__pyx_t_3, function);
    }
  }
  if (!__pyx_t_4) {
    __pyx_t_7 = __Pyx_PyObject_CallOneArg(__pyx_t_3, __pyx_v_d_rate); if (unlikely(!__pyx_t_7)) __PYX_ERR(0, 101, __pyx_L1_error)
    __Pyx_GOTREF(__pyx_t_7);
  } else {
    #if CYTHON_FAST_PYCALL
    if (PyFunction_Check(__pyx_t_3)) {
      PyObject *__pyx_temp[2] = {__pyx_t_4, __pyx_v_d_rate};
      __pyx_t_7 = __Pyx_PyFunction_FastCall(__pyx_t_3, __pyx_temp+1-1, 1+1); if (unlikely(!__pyx_t_7)) __PYX_ERR(0, 101, __pyx_L1_error)
      __Pyx_XDECREF(__pyx_t_4); __pyx_t_4 = 0;
      __Pyx_GOTREF(__pyx_t_7);
    } else
    #endif
    #if CYTHON_FAST_PYCCALL
    if (__Pyx_PyFastCFunction_Check(__pyx_t_3)) {
      PyObject *__pyx_temp[2] = {__pyx_t_4, __pyx_v_d_rate};
      __pyx_t_7 = __Pyx_PyCFunction_FastCall(__pyx_t_3, __pyx_temp+1-1, 1+1); if (unlikely(!__pyx_t_7)) __PYX_ERR(0, 101, __pyx_L1_error)
      __Pyx_XDECREF(__pyx_t_4); __pyx_t_4 = 0;
      __Pyx_GOTREF(__pyx_t_7);
    } else
    #endif
    {
      __pyx_t_9 = PyTuple_New(1+1); if (unlikely(!__pyx_t_9)) __PYX_ERR(0, 101, __pyx_L1_error)
      __Pyx_GOTREF(__pyx_t_9);
      __Pyx_GIVEREF(__pyx_t_4); PyTuple_SET_ITEM(__pyx_t_9, 0, __pyx_t_4); __pyx_t_4 = NULL;
      __Pyx_INCREF(__pyx_v_d_rate);
      __Pyx_GIVEREF(__pyx_v_d_rate);
      PyTuple_SET_ITEM(__pyx_t_9, 0+1, __pyx_v_d_rate);
      __pyx_t_7 = __Pyx_PyObject_Call(__pyx_t_3, __pyx_t_9, NULL); if (unlikely(!__pyx_t_7)) __PYX_ERR(0, 101, __pyx_L1_error)
      __Pyx_GOTREF(__pyx_t_7);
      __Pyx_DECREF(__pyx_t_9); __pyx_t_9 = 0;
    }
  }
  __Pyx_DECREF(__pyx_t_3); __pyx_t_3 = 0;
  __pyx_t_5 = __Pyx_PyObject_IsTrue(__pyx_t_7); if (unlikely(__pyx_t_5 < 0)) __PYX_ERR(0, 101, __pyx_L1_error)
  __Pyx_DECREF(__pyx_t_7); __pyx_t_7 = 0;
  __pyx_t_12 = ((!__pyx_t_5) != 0);
  if (__pyx_t_12) {
/* … */
  }
```

```
+102:         raise ValueError('rate_ga gives complex rate for arguments')
```

```
    __pyx_t_7 = __Pyx_PyObject_Call(__pyx_builtin_ValueError, __pyx_tuple__9, NULL); if (unlikely(!__pyx_t_7)) __PYX_ERR(0, 102, __pyx_L1_error)
    __Pyx_GOTREF(__pyx_t_7);
    __Pyx_Raise(__pyx_t_7, 0, 0, 0);
    __Pyx_DECREF(__pyx_t_7); __pyx_t_7 = 0;
    __PYX_ERR(0, 102, __pyx_L1_error)
/* … */
  __pyx_tuple__9 = PyTuple_Pack(1, __pyx_kp_s_rate_ga_gives_complex_rate_for_a); if (unlikely(!__pyx_tuple__9)) __PYX_ERR(0, 102, __pyx_L1_error)
  __Pyx_GOTREF(__pyx_tuple__9);
  __Pyx_GIVEREF(__pyx_tuple__9);
```

```
+103:     if d_rate < 0:
```

```
  __pyx_t_7 = PyObject_RichCompare(__pyx_v_d_rate, __pyx_int_0, Py_LT); __Pyx_XGOTREF(__pyx_t_7); if (unlikely(!__pyx_t_7)) __PYX_ERR(0, 103, __pyx_L1_error)
  __pyx_t_12 = __Pyx_PyObject_IsTrue(__pyx_t_7); if (unlikely(__pyx_t_12 < 0)) __PYX_ERR(0, 103, __pyx_L1_error)
  __Pyx_DECREF(__pyx_t_7); __pyx_t_7 = 0;
  if (__pyx_t_12) {
/* … */
  }
```

```
+104:         raise ValueError('rate_ga gives negative rate for arguments')
```

```
    __pyx_t_7 = __Pyx_PyObject_Call(__pyx_builtin_ValueError, __pyx_tuple__10, NULL); if (unlikely(!__pyx_t_7)) __PYX_ERR(0, 104, __pyx_L1_error)
    __Pyx_GOTREF(__pyx_t_7);
    __Pyx_Raise(__pyx_t_7, 0, 0, 0);
    __Pyx_DECREF(__pyx_t_7); __pyx_t_7 = 0;
    __PYX_ERR(0, 104, __pyx_L1_error)
/* … */
  __pyx_tuple__10 = PyTuple_Pack(1, __pyx_kp_s_rate_ga_gives_negative_rate_for); if (unlikely(!__pyx_tuple__10)) __PYX_ERR(0, 104, __pyx_L1_error)
  __Pyx_GOTREF(__pyx_tuple__10);
  __Pyx_GIVEREF(__pyx_tuple__10);
```

```
 105:
```

```
+106:     return d_rate
```

```
  __Pyx_XDECREF(__pyx_r);
  __Pyx_INCREF(__pyx_v_d_rate);
  __pyx_r = __pyx_v_d_rate;
  goto __pyx_L0;
```

```
 107:
```

```
 108:
```

```
+109: def rate_dc(v_channel, nda_interf, v_dc_bits, v_ga_bits, d_lambda):
```

```
/* Python wrapper */
static PyObject *__pyx_pw_9dist_recv_5rates_7rate_dc(PyObject *__pyx_self, PyObject *__pyx_args, PyObject *__pyx_kwds); /*proto*/
static char __pyx_doc_9dist_recv_5rates_6rate_dc[] = " Rate for Distributed Compression distributed receive with reception at base\n        Input:\n            v_channel = Channel vector h (size N+1 x 1)\n            nda_interf = Interference covariance matrix Sigma (size N+1 x N+1)\n            v_dc_bits = Post-compression helper encoding rates. (size N x 1)\n            v_ga_bits = Pre-compression Gaussian encoder rates. (size N x 1)\n            d_lambda = Hull parameter\n        Output:\n            d_rate = Rate (in bits), 0 if configuration is not valid\n    ";
static PyMethodDef __pyx_mdef_9dist_recv_5rates_7rate_dc = {"rate_dc", (PyCFunction)__pyx_pw_9dist_recv_5rates_7rate_dc, METH_VARARGS|METH_KEYWORDS, __pyx_doc_9dist_recv_5rates_6rate_dc};
static PyObject *__pyx_pw_9dist_recv_5rates_7rate_dc(PyObject *__pyx_self, PyObject *__pyx_args, PyObject *__pyx_kwds) {
  PyObject *__pyx_v_v_channel = 0;
  PyObject *__pyx_v_nda_interf = 0;
  PyObject *__pyx_v_v_dc_bits = 0;
  PyObject *__pyx_v_v_ga_bits = 0;
  PyObject *__pyx_v_d_lambda = 0;
  PyObject *__pyx_r = 0;
  __Pyx_RefNannyDeclarations
  __Pyx_RefNannySetupContext("rate_dc (wrapper)", 0);
  {
    static PyObject **__pyx_pyargnames[] = {&__pyx_n_s_v_channel,&__pyx_n_s_nda_interf,&__pyx_n_s_v_dc_bits,&__pyx_n_s_v_ga_bits,&__pyx_n_s_d_lambda,0};
    PyObject* values[5] = {0,0,0,0,0};
    if (unlikely(__pyx_kwds)) {
      Py_ssize_t kw_args;
      const Py_ssize_t pos_args = PyTuple_GET_SIZE(__pyx_args);
      switch (pos_args) {
        case  5: values[4] = PyTuple_GET_ITEM(__pyx_args, 4);
        case  4: values[3] = PyTuple_GET_ITEM(__pyx_args, 3);
        case  3: values[2] = PyTuple_GET_ITEM(__pyx_args, 2);
        case  2: values[1] = PyTuple_GET_ITEM(__pyx_args, 1);
        case  1: values[0] = PyTuple_GET_ITEM(__pyx_args, 0);
        case  0: break;
        default: goto __pyx_L5_argtuple_error;
      }
      kw_args = PyDict_Size(__pyx_kwds);
      switch (pos_args) {
        case  0:
        if (likely((values[0] = PyDict_GetItem(__pyx_kwds, __pyx_n_s_v_channel)) != 0)) kw_args--;
        else goto __pyx_L5_argtuple_error;
        case  1:
        if (likely((values[1] = PyDict_GetItem(__pyx_kwds, __pyx_n_s_nda_interf)) != 0)) kw_args--;
        else {
          __Pyx_RaiseArgtupleInvalid("rate_dc", 1, 5, 5, 1); __PYX_ERR(0, 109, __pyx_L3_error)
        }
        case  2:
        if (likely((values[2] = PyDict_GetItem(__pyx_kwds, __pyx_n_s_v_dc_bits)) != 0)) kw_args--;
        else {
          __Pyx_RaiseArgtupleInvalid("rate_dc", 1, 5, 5, 2); __PYX_ERR(0, 109, __pyx_L3_error)
        }
        case  3:
        if (likely((values[3] = PyDict_GetItem(__pyx_kwds, __pyx_n_s_v_ga_bits)) != 0)) kw_args--;
        else {
          __Pyx_RaiseArgtupleInvalid("rate_dc", 1, 5, 5, 3); __PYX_ERR(0, 109, __pyx_L3_error)
        }
        case  4:
        if (likely((values[4] = PyDict_GetItem(__pyx_kwds, __pyx_n_s_d_lambda)) != 0)) kw_args--;
        else {
          __Pyx_RaiseArgtupleInvalid("rate_dc", 1, 5, 5, 4); __PYX_ERR(0, 109, __pyx_L3_error)
        }
      }
      if (unlikely(kw_args > 0)) {
        if (unlikely(__Pyx_ParseOptionalKeywords(__pyx_kwds, __pyx_pyargnames, 0, values, pos_args, "rate_dc") < 0)) __PYX_ERR(0, 109, __pyx_L3_error)
      }
    } else if (PyTuple_GET_SIZE(__pyx_args) != 5) {
      goto __pyx_L5_argtuple_error;
    } else {
      values[0] = PyTuple_GET_ITEM(__pyx_args, 0);
      values[1] = PyTuple_GET_ITEM(__pyx_args, 1);
      values[2] = PyTuple_GET_ITEM(__pyx_args, 2);
      values[3] = PyTuple_GET_ITEM(__pyx_args, 3);
      values[4] = PyTuple_GET_ITEM(__pyx_args, 4);
    }
    __pyx_v_v_channel = values[0];
    __pyx_v_nda_interf = values[1];
    __pyx_v_v_dc_bits = values[2];
    __pyx_v_v_ga_bits = values[3];
    __pyx_v_d_lambda = values[4];
  }
  goto __pyx_L4_argument_unpacking_done;
  __pyx_L5_argtuple_error:;
  __Pyx_RaiseArgtupleInvalid("rate_dc", 1, 5, 5, PyTuple_GET_SIZE(__pyx_args)); __PYX_ERR(0, 109, __pyx_L3_error)
  __pyx_L3_error:;
  __Pyx_AddTraceback("dist_recv.rates.rate_dc", __pyx_clineno, __pyx_lineno, __pyx_filename);
  __Pyx_RefNannyFinishContext();
  return NULL;
  __pyx_L4_argument_unpacking_done:;
  __pyx_r = __pyx_pf_9dist_recv_5rates_6rate_dc(__pyx_self, __pyx_v_v_channel, __pyx_v_nda_interf, __pyx_v_v_dc_bits, __pyx_v_v_ga_bits, __pyx_v_d_lambda);

  /* function exit code */
  __Pyx_RefNannyFinishContext();
  return __pyx_r;
}

static PyObject *__pyx_pf_9dist_recv_5rates_6rate_dc(CYTHON_UNUSED PyObject *__pyx_self, PyObject *__pyx_v_v_channel, PyObject *__pyx_v_nda_interf, PyObject *__pyx_v_v_dc_bits, PyObject *__pyx_v_v_ga_bits, PyObject *__pyx_v_d_lambda) {
  PyObject *__pyx_v_b_is_valid = NULL;
  PyObject *__pyx_r = NULL;
  __Pyx_TraceDeclarations
  __Pyx_TraceFrameInit(__pyx_codeobj__11)
  __Pyx_RefNannyDeclarations
  __Pyx_RefNannySetupContext("rate_dc", 0);
  __Pyx_TraceCall("rate_dc", __pyx_f[0], 109, 0, __PYX_ERR(0, 109, __pyx_L1_error));
/* … */
  /* function exit code */
  __pyx_L1_error:;
  __Pyx_XDECREF(__pyx_t_1);
  __Pyx_XDECREF(__pyx_t_2);
  __Pyx_XDECREF(__pyx_t_3);
  __Pyx_XDECREF(__pyx_t_5);
  __Pyx_AddTraceback("dist_recv.rates.rate_dc", __pyx_clineno, __pyx_lineno, __pyx_filename);
  __pyx_r = NULL;
  __pyx_L0:;
  __Pyx_XDECREF(__pyx_v_b_is_valid);
  __Pyx_XGIVEREF(__pyx_r);
  __Pyx_TraceReturn(__pyx_r, 0);
  __Pyx_RefNannyFinishContext();
  return __pyx_r;
}
/* … */
  __pyx_tuple__15 = PyTuple_Pack(6, __pyx_n_s_v_channel, __pyx_n_s_nda_interf, __pyx_n_s_v_dc_bits, __pyx_n_s_v_ga_bits, __pyx_n_s_d_lambda, __pyx_n_s_b_is_valid); if (unlikely(!__pyx_tuple__15)) __PYX_ERR(0, 109, __pyx_L1_error)
  __Pyx_GOTREF(__pyx_tuple__15);
  __Pyx_GIVEREF(__pyx_tuple__15);
/* … */
  __pyx_t_2 = PyCFunction_NewEx(&__pyx_mdef_9dist_recv_5rates_7rate_dc, NULL, __pyx_n_s_dist_recv_rates); if (unlikely(!__pyx_t_2)) __PYX_ERR(0, 109, __pyx_L1_error)
  __Pyx_GOTREF(__pyx_t_2);
  if (PyDict_SetItem(__pyx_d, __pyx_n_s_rate_dc, __pyx_t_2) < 0) __PYX_ERR(0, 109, __pyx_L1_error)
  __Pyx_DECREF(__pyx_t_2); __pyx_t_2 = 0;
```

```
 110:     """ Rate for Distributed Compression distributed receive with reception at base
```

```
 111:         Input:
```

```
 112:             v_channel = Channel vector h (size N+1 x 1)
```

```
 113:             nda_interf = Interference covariance matrix Sigma (size N+1 x N+1)
```

```
 114:             v_dc_bits = Post-compression helper encoding rates. (size N x 1)
```

```
 115:             v_ga_bits = Pre-compression Gaussian encoder rates. (size N x 1)
```

```
 116:             d_lambda = Hull parameter
```

```
 117:         Output:
```

```
 118:             d_rate = Rate (in bits), 0 if configuration is not valid
```

```
 119:     """
```

```
+120:     b_is_valid = dc.checkdc(v_channel, nda_interf, v_dc_bits, v_ga_bits, d_lambda)
```

```
  __pyx_t_2 = __Pyx_GetModuleGlobalName(__pyx_n_s_dc); if (unlikely(!__pyx_t_2)) __PYX_ERR(0, 120, __pyx_L1_error)
  __Pyx_GOTREF(__pyx_t_2);
  __pyx_t_3 = __Pyx_PyObject_GetAttrStr(__pyx_t_2, __pyx_n_s_checkdc); if (unlikely(!__pyx_t_3)) __PYX_ERR(0, 120, __pyx_L1_error)
  __Pyx_GOTREF(__pyx_t_3);
  __Pyx_DECREF(__pyx_t_2); __pyx_t_2 = 0;
  __pyx_t_2 = NULL;
  __pyx_t_4 = 0;
  if (CYTHON_UNPACK_METHODS && unlikely(PyMethod_Check(__pyx_t_3))) {
    __pyx_t_2 = PyMethod_GET_SELF(__pyx_t_3);
    if (likely(__pyx_t_2)) {
      PyObject* function = PyMethod_GET_FUNCTION(__pyx_t_3);
      __Pyx_INCREF(__pyx_t_2);
      __Pyx_INCREF(function);
      __Pyx_DECREF_SET(__pyx_t_3, function);
      __pyx_t_4 = 1;
    }
  }
  #if CYTHON_FAST_PYCALL
  if (PyFunction_Check(__pyx_t_3)) {
    PyObject *__pyx_temp[6] = {__pyx_t_2, __pyx_v_v_channel, __pyx_v_nda_interf, __pyx_v_v_dc_bits, __pyx_v_v_ga_bits, __pyx_v_d_lambda};
    __pyx_t_1 = __Pyx_PyFunction_FastCall(__pyx_t_3, __pyx_temp+1-__pyx_t_4, 5+__pyx_t_4); if (unlikely(!__pyx_t_1)) __PYX_ERR(0, 120, __pyx_L1_error)
    __Pyx_XDECREF(__pyx_t_2); __pyx_t_2 = 0;
    __Pyx_GOTREF(__pyx_t_1);
  } else
  #endif
  #if CYTHON_FAST_PYCCALL
  if (__Pyx_PyFastCFunction_Check(__pyx_t_3)) {
    PyObject *__pyx_temp[6] = {__pyx_t_2, __pyx_v_v_channel, __pyx_v_nda_interf, __pyx_v_v_dc_bits, __pyx_v_v_ga_bits, __pyx_v_d_lambda};
    __pyx_t_1 = __Pyx_PyCFunction_FastCall(__pyx_t_3, __pyx_temp+1-__pyx_t_4, 5+__pyx_t_4); if (unlikely(!__pyx_t_1)) __PYX_ERR(0, 120, __pyx_L1_error)
    __Pyx_XDECREF(__pyx_t_2); __pyx_t_2 = 0;
    __Pyx_GOTREF(__pyx_t_1);
  } else
  #endif
  {
    __pyx_t_5 = PyTuple_New(5+__pyx_t_4); if (unlikely(!__pyx_t_5)) __PYX_ERR(0, 120, __pyx_L1_error)
    __Pyx_GOTREF(__pyx_t_5);
    if (__pyx_t_2) {
      __Pyx_GIVEREF(__pyx_t_2); PyTuple_SET_ITEM(__pyx_t_5, 0, __pyx_t_2); __pyx_t_2 = NULL;
    }
    __Pyx_INCREF(__pyx_v_v_channel);
    __Pyx_GIVEREF(__pyx_v_v_channel);
    PyTuple_SET_ITEM(__pyx_t_5, 0+__pyx_t_4, __pyx_v_v_channel);
    __Pyx_INCREF(__pyx_v_nda_interf);
    __Pyx_GIVEREF(__pyx_v_nda_interf);
    PyTuple_SET_ITEM(__pyx_t_5, 1+__pyx_t_4, __pyx_v_nda_interf);
    __Pyx_INCREF(__pyx_v_v_dc_bits);
    __Pyx_GIVEREF(__pyx_v_v_dc_bits);
    PyTuple_SET_ITEM(__pyx_t_5, 2+__pyx_t_4, __pyx_v_v_dc_bits);
    __Pyx_INCREF(__pyx_v_v_ga_bits);
    __Pyx_GIVEREF(__pyx_v_v_ga_bits);
    PyTuple_SET_ITEM(__pyx_t_5, 3+__pyx_t_4, __pyx_v_v_ga_bits);
    __Pyx_INCREF(__pyx_v_d_lambda);
    __Pyx_GIVEREF(__pyx_v_d_lambda);
    PyTuple_SET_ITEM(__pyx_t_5, 4+__pyx_t_4, __pyx_v_d_lambda);
    __pyx_t_1 = __Pyx_PyObject_Call(__pyx_t_3, __pyx_t_5, NULL); if (unlikely(!__pyx_t_1)) __PYX_ERR(0, 120, __pyx_L1_error)
    __Pyx_GOTREF(__pyx_t_1);
    __Pyx_DECREF(__pyx_t_5); __pyx_t_5 = 0;
  }
  __Pyx_DECREF(__pyx_t_3); __pyx_t_3 = 0;
  __pyx_v_b_is_valid = __pyx_t_1;
  __pyx_t_1 = 0;
```

```
+121:     if b_is_valid:
```

```
  __pyx_t_6 = __Pyx_PyObject_IsTrue(__pyx_v_b_is_valid); if (unlikely(__pyx_t_6 < 0)) __PYX_ERR(0, 121, __pyx_L1_error)
  if (__pyx_t_6) {
/* … */
  }
```

```
+122:         return rate_ga(v_channel, nda_interf, v_ga_bits) - d_lambda
```

```
    __Pyx_XDECREF(__pyx_r);
    __pyx_t_3 = __Pyx_GetModuleGlobalName(__pyx_n_s_rate_ga); if (unlikely(!__pyx_t_3)) __PYX_ERR(0, 122, __pyx_L1_error)
    __Pyx_GOTREF(__pyx_t_3);
    __pyx_t_5 = NULL;
    __pyx_t_4 = 0;
    if (CYTHON_UNPACK_METHODS && unlikely(PyMethod_Check(__pyx_t_3))) {
      __pyx_t_5 = PyMethod_GET_SELF(__pyx_t_3);
      if (likely(__pyx_t_5)) {
        PyObject* function = PyMethod_GET_FUNCTION(__pyx_t_3);
        __Pyx_INCREF(__pyx_t_5);
        __Pyx_INCREF(function);
        __Pyx_DECREF_SET(__pyx_t_3, function);
        __pyx_t_4 = 1;
      }
    }
    #if CYTHON_FAST_PYCALL
    if (PyFunction_Check(__pyx_t_3)) {
      PyObject *__pyx_temp[4] = {__pyx_t_5, __pyx_v_v_channel, __pyx_v_nda_interf, __pyx_v_v_ga_bits};
      __pyx_t_1 = __Pyx_PyFunction_FastCall(__pyx_t_3, __pyx_temp+1-__pyx_t_4, 3+__pyx_t_4); if (unlikely(!__pyx_t_1)) __PYX_ERR(0, 122, __pyx_L1_error)
      __Pyx_XDECREF(__pyx_t_5); __pyx_t_5 = 0;
      __Pyx_GOTREF(__pyx_t_1);
    } else
    #endif
    #if CYTHON_FAST_PYCCALL
    if (__Pyx_PyFastCFunction_Check(__pyx_t_3)) {
      PyObject *__pyx_temp[4] = {__pyx_t_5, __pyx_v_v_channel, __pyx_v_nda_interf, __pyx_v_v_ga_bits};
      __pyx_t_1 = __Pyx_PyCFunction_FastCall(__pyx_t_3, __pyx_temp+1-__pyx_t_4, 3+__pyx_t_4); if (unlikely(!__pyx_t_1)) __PYX_ERR(0, 122, __pyx_L1_error)
      __Pyx_XDECREF(__pyx_t_5); __pyx_t_5 = 0;
      __Pyx_GOTREF(__pyx_t_1);
    } else
    #endif
    {
      __pyx_t_2 = PyTuple_New(3+__pyx_t_4); if (unlikely(!__pyx_t_2)) __PYX_ERR(0, 122, __pyx_L1_error)
      __Pyx_GOTREF(__pyx_t_2);
      if (__pyx_t_5) {
        __Pyx_GIVEREF(__pyx_t_5); PyTuple_SET_ITEM(__pyx_t_2, 0, __pyx_t_5); __pyx_t_5 = NULL;
      }
      __Pyx_INCREF(__pyx_v_v_channel);
      __Pyx_GIVEREF(__pyx_v_v_channel);
      PyTuple_SET_ITEM(__pyx_t_2, 0+__pyx_t_4, __pyx_v_v_channel);
      __Pyx_INCREF(__pyx_v_nda_interf);
      __Pyx_GIVEREF(__pyx_v_nda_interf);
      PyTuple_SET_ITEM(__pyx_t_2, 1+__pyx_t_4, __pyx_v_nda_interf);
      __Pyx_INCREF(__pyx_v_v_ga_bits);
      __Pyx_GIVEREF(__pyx_v_v_ga_bits);
      PyTuple_SET_ITEM(__pyx_t_2, 2+__pyx_t_4, __pyx_v_v_ga_bits);
      __pyx_t_1 = __Pyx_PyObject_Call(__pyx_t_3, __pyx_t_2, NULL); if (unlikely(!__pyx_t_1)) __PYX_ERR(0, 122, __pyx_L1_error)
      __Pyx_GOTREF(__pyx_t_1);
      __Pyx_DECREF(__pyx_t_2); __pyx_t_2 = 0;
    }
    __Pyx_DECREF(__pyx_t_3); __pyx_t_3 = 0;
    __pyx_t_3 = PyNumber_Subtract(__pyx_t_1, __pyx_v_d_lambda); if (unlikely(!__pyx_t_3)) __PYX_ERR(0, 122, __pyx_L1_error)
    __Pyx_GOTREF(__pyx_t_3);
    __Pyx_DECREF(__pyx_t_1); __pyx_t_1 = 0;
    __pyx_r = __pyx_t_3;
    __pyx_t_3 = 0;
    goto __pyx_L0;
```

```
 123:     else:
```

```
+124:         return 0
```

```
  /*else*/ {
    __Pyx_XDECREF(__pyx_r);
    __Pyx_INCREF(__pyx_int_0);
    __pyx_r = __pyx_int_0;
    goto __pyx_L0;
  }
```
